# Antennal and Abdominal Transcriptomes Reveal Chemosensory Genes in the Asian Citrus Psyllid, *Diaphorina citri*

**DOI:** 10.1371/journal.pone.0159372

**Published:** 2016-07-21

**Authors:** Zhongzhen Wu, He Zhang, Shuying Bin, Lei Chen, Qunxin Han, Jintian Lin

**Affiliations:** Institute for Management of Invasive Alien Species, Zhongkai University of Agriculture and Engineering, Guangzhou, PR China; University of California Davis, UNITED STATES

## Abstract

The Asian citrus psyllid, *Diaphorina citri* is the principal vector of the highly destructive citrus disease called Huanglongbing (HLB) or citrus greening, which is a major threat to citrus cultivation worldwide. More effective pest control strategies against this pest entail the identification of potential chemosensory proteins that could be used in the development of attractants or repellents. However, the molecular basis of olfaction in the Asian citrus psyllid is not completely understood. Therefore, we performed this study to analyze the antennal and abdominal transcriptome of the Asian citrus psyllid. We identified a large number of transcripts belonging to nine chemoreception-related gene families and compared their expression in male and female adult antennae and terminal abdomen. In total, 9 odorant binding proteins (OBPs), 12 chemosensory proteins (CSPs), 46 odorant receptors (ORs), 20 gustatory receptors (GRs), 35 ionotropic receptors (IRs), 4 sensory neuron membrane proteins (SNMPs) and 4 different gene families encoding odorant-degrading enzymes (ODEs): 80 cytochrome P450s (CYPs), 12 esterase (ESTs), and 5 aldehyde dehydrogenases (ADE) were annotated in the *D*. *citri* antennal and abdominal transcriptomes. Our results revealed that a large proportion of chemosensory genes exhibited no distinct differences in their expression patterns in the antennae and terminal abdominal tissues. Notably, RNA sequencing (RNA-seq) data and quantitative real time-PCR (qPCR) analyses showed that 4 DictOBPs, 4 DictCSPs, 4 DictIRs, 1 DictSNMP, and 2 DictCYPs were upregulated in the antennae relative to that in terminal abdominal tissues. Furthermore, 2 DictOBPs (DictOBP8 and DictOBP9), 2 DictCSPs (DictOBP8 and DictOBP12), 4 DictIRs (DictIR3, DictIR6, DictIR10, and DictIR35), and 1 DictCYP (DictCYP57) were expressed at higher levels in the male antennae than in the female antennae. Our study provides the first insights into the molecular basis of chemoreception in this insect pest. Further studies on the identified differentially expressed genes would facilitate the understanding of insect olfaction and their role in the interactions between olfactory system and biological processes.

## Introduction

Insects use chemoreception for a variety of survival and reproductive functions, including the identification of food sources, toxic substances, and mating partners. Previous studies have shown that chemoreception-related genes in insects are highly divergent [1–4], as indicated by the presence of two major chemosensory mechanisms, olfaction and gustation, which include several multigene families. These multigene families consist of odorant-binding proteins (OBPs), chemosensory proteins (CSPs), sensory neuron membrane proteins (SNMPs), olfactory receptors (ORs), ionotropic receptors (IR), and gustatory receptors (GRs) [5], and odorant-degrading enzymes (ODEs) that are involved in peripheral olfactory processes. Specifically, the chemosensory multigene families perform diverse functions, including chemosensory as well as non-sensory functions, and are mainly expressed in the antennae or maxillary palps, which contain chemosensory sensilla with sensory neurons. These include OBPs and CSPs, which are located in the sensillar lymph, and are regarded as solubilizers and carriers of odorants and pheromones, and the chemoreceptor superfamily formed by the OR, IR, and GR families, located on the dendrites of olfactory receptor neurons (ORNs). Moreover, members of the chemosensory multigene families are expressed in other tissues and were shown to be involved in non-sensory functions. Thus far, OBPs and CSPs have been found in pheromone glands where they are involved in pheromone delivery [6–12], and in the reproductive organs and eggs where they regulate development [13–16]. Furthermore, ORs identified in testes are involved in sperm activation [17].

The Asian citrus psyllid, *Diaphorina citri* Kuwayama (Hemiptera: Psyllidae) is the principal vector of the fastidious bacterium *Candidatus* Liberibacter asiaticus (CLas), the causal agent of citrus greening disease or Huanglongbing (HLB), which is a highly destructive disease that is a major threat to citrus cultivation worldwide [18]. The psyllids are specific to citrus, on which they feed, mate, oviposit, and develop on new flush shoots [19, 20]. Currently, the control of *D*. *citri* has been recognized as the key approach to prevent the spread of HLB. Traditional *D*. *citri* management has used broad-spectrum insecticides (pyrethroid, organophosphate, and neonicotinoid classes), which has led to the evolution of pesticide-resistant strains [21–23]. Therefore, it is critical to identify potential molecular targets, particularly chemosensory proteins (binding proteins and receptors) to develop novel control strategies that interfere with olfaction [24–26]. However, the molecular components of psyllid olfaction have not been described. While the molecular basis of insect olfaction has been extensively studied in Lepidopteran and Dipteran insects, elucidation of the molecular components and mechanisms that comprise the hemipteran olfactory system is limited [27–33]. In addition, the molecular basis underlying chemical communication, including olfactory sensing, pheromone biosynthesis, and oviposition in psyllids is lacking.

In the present study, we analyzed the antennal and abdominal transcriptomes of the Asian citrus psyllid. The antenna was chosen because of its obvious involvement in the chemosensory function and the abdomen was chosen due to its role in reproduction and potential sex pheromone production. In addition, these two tissues allowed comparison of the chemosensory gene expression profiles between an olfactory and a non-olfactory tissue in males and females in order to identify olfactory genes in the Asian citrus psyllid. The expression of these chemosensory gene transcripts was analyzed based on transcriptome profiling using RNA sequencing (RNA-seq) data and validated by quantitative real time-PCR (qPCR). This is the first study on the olfactory molecular components of the Asian citrus psyllid, which provides the molecular basis to better understand psyllid chemoreception.

## Methods

### Ethics statement

No specific permission was required to collect insects from the locations mentioned in this article. The locations sampled were not privately owned or protected in any way, and this field study did not involve endangered or protected species.

### Psyllid rearing and collection

The Asian citrus psyllid, *D*. *citri* was originally collected from the citrus groves in Fogang County, Qingyuan City, Guangdong Province, China (E113°31', N23°52'). The collected psyllids were reared in a greenhouse (21°C–26°C, 60%–80% RH) with natural lighting throughout the day. For the past 3 years, the psyllids were fed Citrus Reticulate Blanco Cv. Shatangju. To synchronize the developmental stages, a large number of eggs were collected and moved to artificial climate boxes for mass rearing. The controlled environmental conditions were 26°C ± 1°C, 70% relative humidity, and a 12 h: 12 h dark light cycle. Emerging adult psyllids were segregated by sex under a stereoscope, followed by morphological identification.

### RNA isolation and Illumina sequencing

From the newly emerged adults (2-day-old), about 920 male antennae, 1,000 female antennae, 200 male terminal abdomen (cut from the 5^th^ abdominal segments, including aedaeagus and clasper), and 200 female terminal abdomen (cut from the 5^th^ abdominal segments, including ovipositor) were manually dissected and immediately transferred to a polypropylene tube immersed in liquid nitrogen. All frozen tissues were crushed and ground in liquid nitrogen, and total RNA was extracted using TRIzol (Invitrogen, Carlsbad, CA, USA) according to the manufacturer’s instructions. RNA concentration and quality were assessed using standard procedures as recommended for Illumina (Illumina, San Diego, CA, USA) sequencing.

cDNA library preparation, including fragmentation and barcoding (ligation of specific adapters), was performed following the Illumina protocol. After quality evaluation with an Agilent 2010 Bioanalyzer, the libraries were diluted with an elution buffer and loaded on an Illumina HiSeq2500 for sequencing (paired-end, 2 × 100 bp; total, 200 cycles), and each cDNA library was deep-sequenced for 6 gigabytes. The majority of Illumina library were about 200 bp in size, and both ends were sequenced. The raw data from Illumina deep-sequencing were deposited to the NCBI Short Read Archive (SRA) database as BioProject Accession Number SRP064720.

### *De novo* assembly and sequence annotation

Clean reads were generated from raw reads by removing the adaptor sequences, duplicated sequences, ambiguous reads, and low-quality reads. *De novo* assembly of clean data was accomplished by the Trinity software to generate unigenes [34, 35] with min_kmer_cov set to 2 by default and all other parameters set at default. Using BLASTx, unigenes were first searched against protein databases, including NCBI-Nr, NCBI- Nt, KEGG Orthology (KO), Swiss-Prot, PFAM, Gene Ontology (GO), and euKaryotic Ortholog Groups (KOG) (significant thresholds of E-value < 10^−5^). Proteins with the highest sequence similarity to the given unigenes along with putative functional annotations were retrieved. BLAST results were then imported into Blast2GO pipeline for GO annotation. Protein coding region prediction was performed using the ORF Predictor according to the BLAST results. Then, the Blast2GO program was used to retrieve the GO annotation of unigenes and the GO functional classification was obtained using WEGO (http://wego.genomics.org.cn/) [36].

### Identification of chemosensory genes, sequence alignment and phylogenetic analysis

The open reading frames (ORFs) of putative chemosensory genes were predicted using the ORF finder (http://www.ncbi.nlm.nih.gov/gorf/gorf.html). The signal peptides of OBPs and CSPs were predicted using SignalP 4.1 Server (http://www.cbs.dtu.dk/services/SignalP/). The trans-membrane domains (TMDs) of ORs, IRs, GRs, and SNMPs were predicted using TMHMM 2.0 (http://www.cbs.dtu.dk/services/TMHMM).

Phylogenetic analyses of *D*. *citri* chemosensory genes were performed in conjunction with other insect chemosensory sequences from previously published data (S1 Text). After removal of the signal peptides from the OBP and CSP datasets, the resulting amino acid sequences of all chemosensory genes were aligned using MAFFT v.6 [37] (E-INS-I parameter set for OBPs, CSPs and SNMPs; FFT-NS-2 parameter set for ORs, GRs and IRs). Maximum-likelihood trees were constructed for OBP, CSP, OR, GR, and SNMP using MEGA6 [38] with the corresponding best substitution model, and the phylogenetic tree for IR was constructed using FastTree 2.1.7 [39]. Robustness of the branches was assessed with 1,000 bootstrap pseudo-replicates. Dendrograms were viewed and edited in FigTree (http://tree.bio.ed.ac.uk/software/figtree/). For comparative purposes, multiple putative DcitOBP and DcitCSP protein sequences (without the signal peptides), DcitIR protein sequences and DcitSNMP protein sequences were aligned using the E-INS-I strategy in MAFFT [37] and rendered in Geneious 8.1.7 (http://www.geneious.com) and Jalview 2.0.1[40].

### Gene expression analysis

Clean reads were mapped back onto the assembled transcriptome and read count for each gene was obtained from the mapping results. Gene expression levels for each sample were estimated by RSEM [41] using default parameters in Bowtie2. Additionally, expression levels were assessed in terms of FPKM values (fragments per kilobase per million reads), which were calculated based on the number of mapped transcript fragments corrected for transcript length and sequencing depth [42].

Prior to differential gene expression analysis in the four transcriptomes, the read counts were adjusted by edgeR program package through one scaling normalized factor. Differential expression analysis of two samples was performed using the DEGseq R package [43]. P value was adjusted using q value [44]. A q value < 0.005 & |log2 (foldchange)| > 1 was set as the threshold for significant differential expression. Then, paired comparisons were conducted in the following manner: male antennae *vs*. female antennae, male terminal abdomen *vs*. female terminal abdomen, male antennae *vs*. male terminal abdomen, and female antennae *vs*. female terminal abdomen. Differential expression of genes was processed by following strict criteria: FPKM value differences between the two analyzed transcriptomes > 3-fold, in combination with a significant Bonferroni-corrected p-value at < 2.2 × 10^−4^.

### Quantitative real time-PCR analysis

Quantitative real time-PCR (qPCR) was used to verify expression levels of the selected sex- and tissue-specific chemosensory gene transcripts in a Light Cycler 480 System (Roche, USA) using the SYBR Premix EX Taq (Takara, China). Total RNA isolated from the four tissues described above was used to synthesize first-strand cDNA using the First strand cDNA synthesis kit (Takara, China). Two reference genes (*actin-1* and *GAPDH2*) were used as internal controls. Primers of the target and reference genes were designed using the Primer 3 program (http://frodo.wi.mit.edu/). Negative controls without cDNA template or transcriptase were included in each experiment. Each qPCR reaction was performed with three technical replicates and three biological replicates. All primer sequences are listed in S1 Table. The relative transcript abundance of the genes in each tissue was calculated using the comparative 2^-ΔΔCT^ method [45]. Data analysis was performed using Prism 6.0 (GraphPad Software, CA). Statistical significance was analyzed by ANOVA, followed by a Tukey’s multiple comparison test. A value of P < 0.05 was considered statistically significant.

## Results

### Antennal and abdominal transcriptome

**Fig 1:**
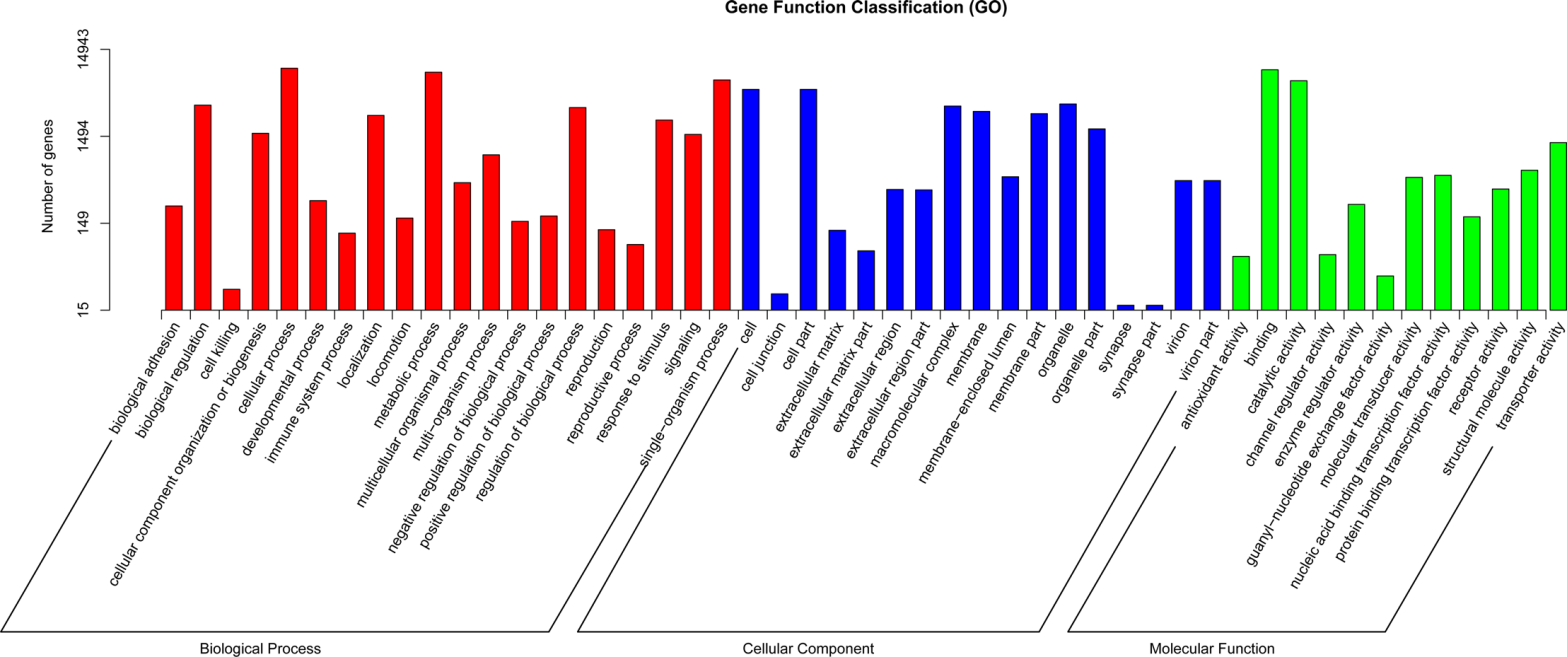
GO classifications of unigenes.

Illumina sequencing generated a total of 51,405,960 (100.00% of the total reads) and 48,894,536 (95.99%) clean reads for the female antennal and abdominal transcriptomes, and 50,686,376 (96.94%) and 55,986,086 (96.28%) clean reads for the male antennal and abdominal transcriptomes, respectively (S2 Table). The combined Trinity assembly of all above transcriptomes resulted in 78,154 unigenes from which 106,743 non-redundant putative transcripts were predicted. For annotations, unigenes longer than 200 bp were used as query in the NCBI, Swiss-PROT, KEGG, COG, and GO databases using BLASTx with a cut-off E value of 10^−5^. Subsequently, 16,967 (21.7%), were annotated using the NCBI-Nr database, 3,041 (3.89%) by NCBI-Nt, 5,813 (7.43%) by KO, 10,824 (13.84%) by Swiss-PROT, 14,834 (18.98%) by Pfam, 14,943 (19.11%) by GO, and 8,348 (10.68%) by KOG (S3 Table). Of the 78,154 unigenes, 14,943 (19.11%) had at least one GO term (Fig 1), and the following categories were well represented: “binding” (24574, 31.4%), “response to stimulus” (3121, 4.0%), “signal transducer activity” (371, 0.5%), “hydrolase activity” (2783, 3.6%), and “transferase activity” (2652, 3.4%).

### Chemosensory gene expression profiles

Based on homology analysis, a total of 223 chemosensory-related transcripts were predicted. These included transcripts putatively involved in odorant binding (OBPs and CSPs, S4 Table), chemosensory reception (ORs, GRs, IRs and SNMPs, S5 Table), and odorant degradation (cytochrome P450s (CYPs), esterases (ESTs), aldehyde dehydrogenases (ADEs), S6 Table).

### Odorant binding proteins

**Fig 2:**
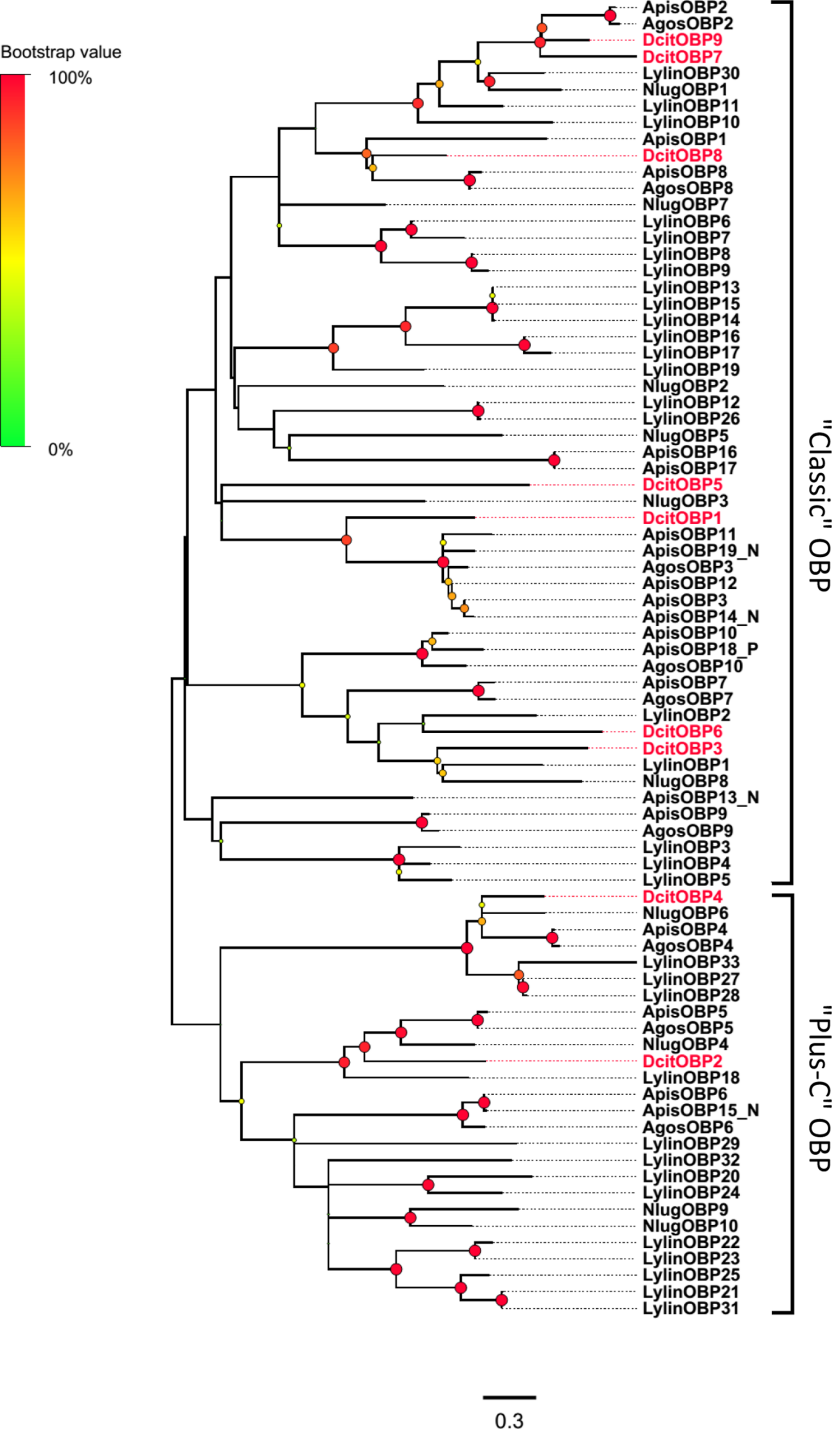
Phylogenetic relationship between DictOBPs.

Nine candidate OBP transcripts were identified based on sequence similarity with annotated orthologous sequences and the presence of conserved cysteine residues characteristic to insect OBPs (S4 Table). Based on the hemipteran ‘Classic’ OBP cysteine motif (C1-X_22–32_-C2-X_3_- C3-X_36–46_-C4-X_8–14_-C5-X_8_-C6) [46], 7 DcitOBP transcripts (DcitOBP1, 3, 5–9) were classified as ‘Classic’ OBPs (S1 Fig). The remaining two DcitOBP transcripts (DcitOBP2, 4) were classified as ‘Plus-C’ (S2 Fig), which is characterized by a cysteine spacing pattern consisting of C1-X_8–41_-C2-X_3_-C3-X_39–47_-C4-X_17–29_-C4a-X_9_-C5-X_8_-C6-P-X_9–11_-C6a [47]. Intriguingly, DcitOBP4 more significantly deviated from the typical ‘Plus-C’ pattern with 52 residues between C1–C2, eight residues between C4–C5, a missing C6 and absence of the conserved proline residue after C6 (S2 Fig). In addition, analysis of the predicted DcitOBP amino acid sequences with signal peptide prediction algorithms suggested that all the DcitOBPs, except three (DcitOBP1, 5, and 9) had defined signal peptide sequences. Maximum-likelihood phylogenetic analysis with hemipteran OBPs showed that all DcitOBP transcripts segregated into the ‘Classic’ and ‘Plus-C’ OBP sub-families (Fig 2) based on the number of cysteines. In the ‘Classic’ OBP clade, 7 ‘Classic’ DcitOBP transcripts clustered with other classic hemipteran OBPs, which were distributed in four well distinct sub-groups.

**Fig 3:**
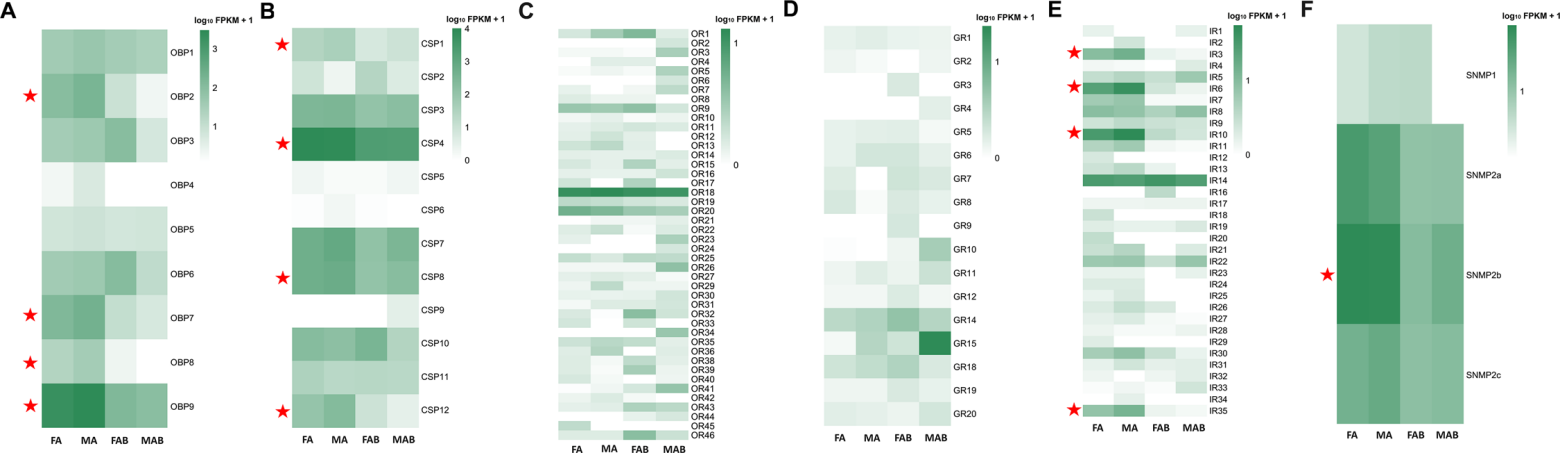
Expression profiles of chemosensory genes. A: OBP; B: CSP; C: OR; D: GR; E: IR; and F: SNMP.

All these OBP transcripts were detected in both the antennae and terminal abdomen of both sexes. Transcripts of three Classic OBPs (OBP7, 8 and 9) and one Plus-C OBP (OBP2) were expressed significantly higher in the antennae when compared to the terminal abdomen (Red stars in Fig 3A; S7 Table). Among these, the abundance of DcitOBP9 transcript was the highest in the antennae (Fig 3A; S4 Table).

### Chemosensory proteins

**Fig 4:**
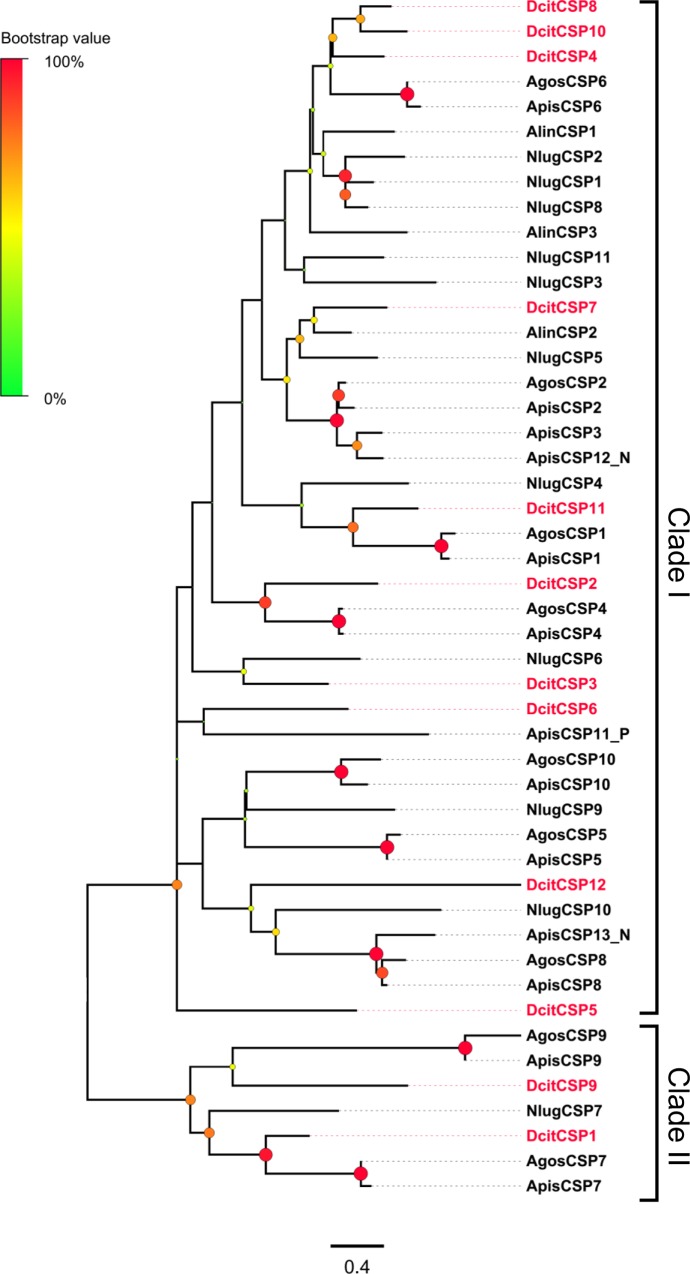
Phylogenetic relationship between DictCSPs.

Twelve transcripts encoding candidate CSPs were identified, and they had an OS-D domain and the typical four cysteine signature (C1-X_6_-C2-X_6-18_-C3-X_2_-C4) of CSPs (S4 Table and S3 Fig). Three of these transcripts encoded partial proteins (DcitCSP5, 6, and 9), whereas the others were full-length CDS. All full-length DcitCSPs, but except DcitCSP4, possessed a signal peptide. Phylogenetic analysis of all DcitCSPs showed two well distinct clades (clades I and II), as seen in CSPs of other hemipteran species. Two DcitCSP transcripts (DcitCSP1 and DcitCSP9) grouped with the “divergent” clade II, whereas the remaining DcitCSP transcripts clustered with the bigger group (Fig 4).These CSP transcripts were detected in at least one of the analyzed tissues (Fig 3B; S4 Table). Transcripts of four CSPs (CSP1, 4, 8 and 12) were upregulated in the antennae when compared to the terminal abdomen (Red stars in Fig 4B; S7 Table). Among all CSP transcripts, DcitCSP4 had the highest expression.

### Odorant receptors

**Fig 5:**
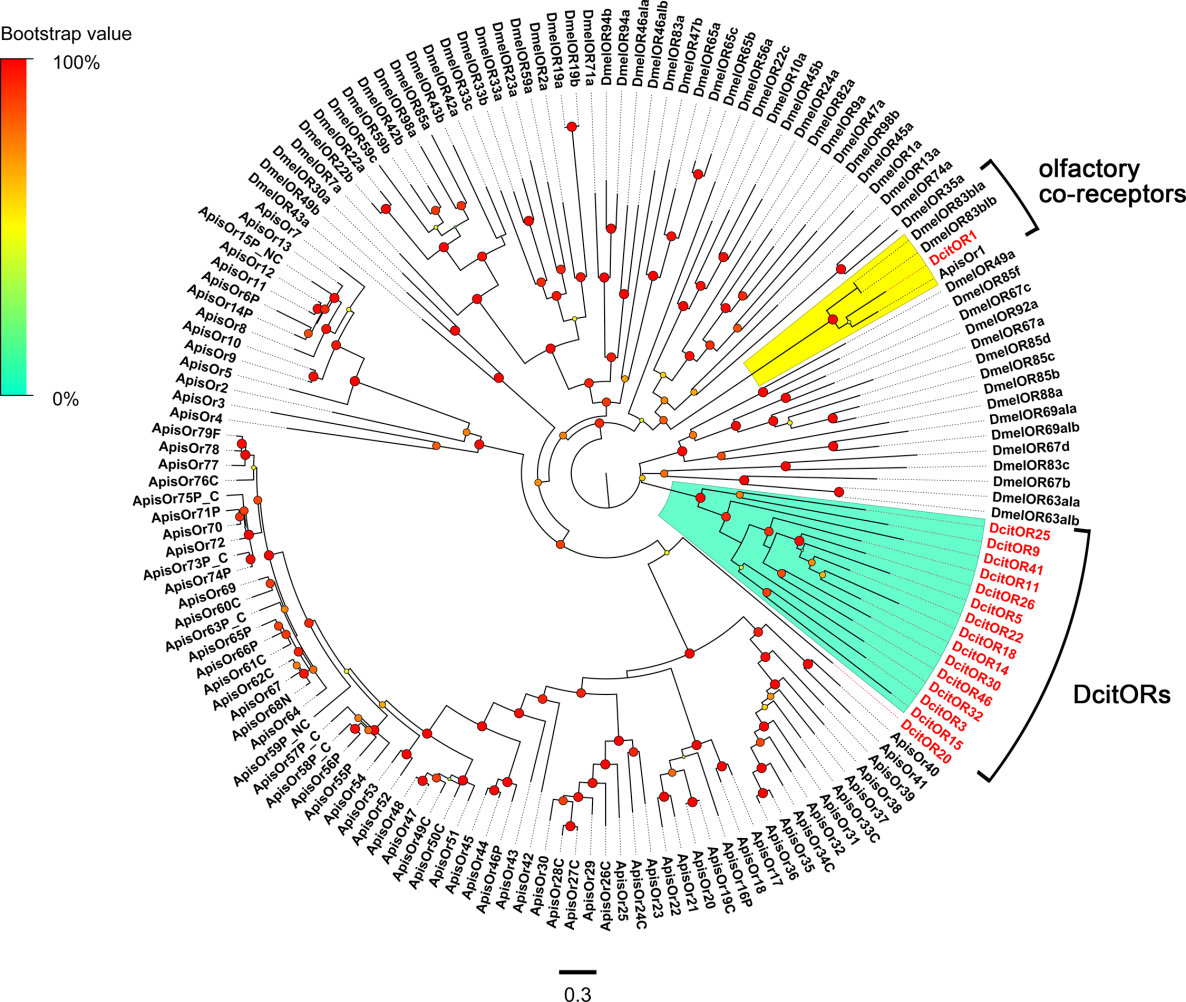
Phylogenetic relationship between DictORs.

Analysis of the combined transcriptomes from all four *D*. *citri* tissues resulted in the identification of 46 candidate OR transcripts (S5 Table). Eleven of these had full-length CDS, and encoded proteins consisting of more than 305 amino acids. Among the full-length DcitOR transcripts, only one transcript (DcitOR1), which was an ortholog of the odorant receptor co-receptor (ORco), had the 7 transmembrane (TM) odorant receptor domains, whereas the others were predicted to have less than 7 TM domains. In the phylogenetic analysis, the co-receptor DcitOR1, grouped into a conserved clade containing ORco from *D*. *melanogaster* and *A*. *pisum*. The remaining DcitOR transcripts, except for DcitOR20, clustered together into independent species-specific clades (Fig 5).

Expression levels of almost all OR transcripts (FPKM of only DcitOR18 was >10) were low in both antennae and terminal abdomen (S5 Table), and were not significantly different between these tissues (Fig 3C; S7 Table). Among them, DcitOR18 had the highest level of expression in the male antennae (FPKM: 10.32) followed by DcitOR20 for which the FPKM value in the male antennae was 4.63 (S5 Table). The Orco transcripts (DcitOR1) with very low expression (FPKM: 0.49–3.69) were observed in the antennae or terminal abdomen of both sexes (S5 Table).

### Gustatory receptors

Twenty candidate GR transcripts were identified in the combined transcriptomes of *D*. *citri* tissues and had the 7TM chemosensory receptor domain (S5 Table). Except for DcitGR14, which was a full-length CDS encoding a protein with 393 amino acids, almost all DcitGR transcripts were partial fragments that encoded overlapping regions with low amino acid sequence identity, thus indicating their origin from separate genes. Interestingly, only one putative CO_2_ receptor (DcitGR14) ortholog [48] was found in our combined transcriptomes. A majority of the partial length DcitGR transcripts also showed high amino acid sequence similarity to known sugar receptors (S5 Table).

The range of GR transcript expression levels varied largely in the analyzed tissues with FPKM values ranging from 0.03–22.52 (S5 Table). Among them, a lower expression (FPKM: 0.03) was observed only for the putative CO_2_ receptor, DcitGR14, found in the antennae and the terminal abdomen (Fig 3D). Expression of DcitGR15 was significantly higher in the male terminal abdomen when compared to the other tissues (FPKM: 22.52) (Fig 3D).

### Ionotropic receptors

**Fig 6:**
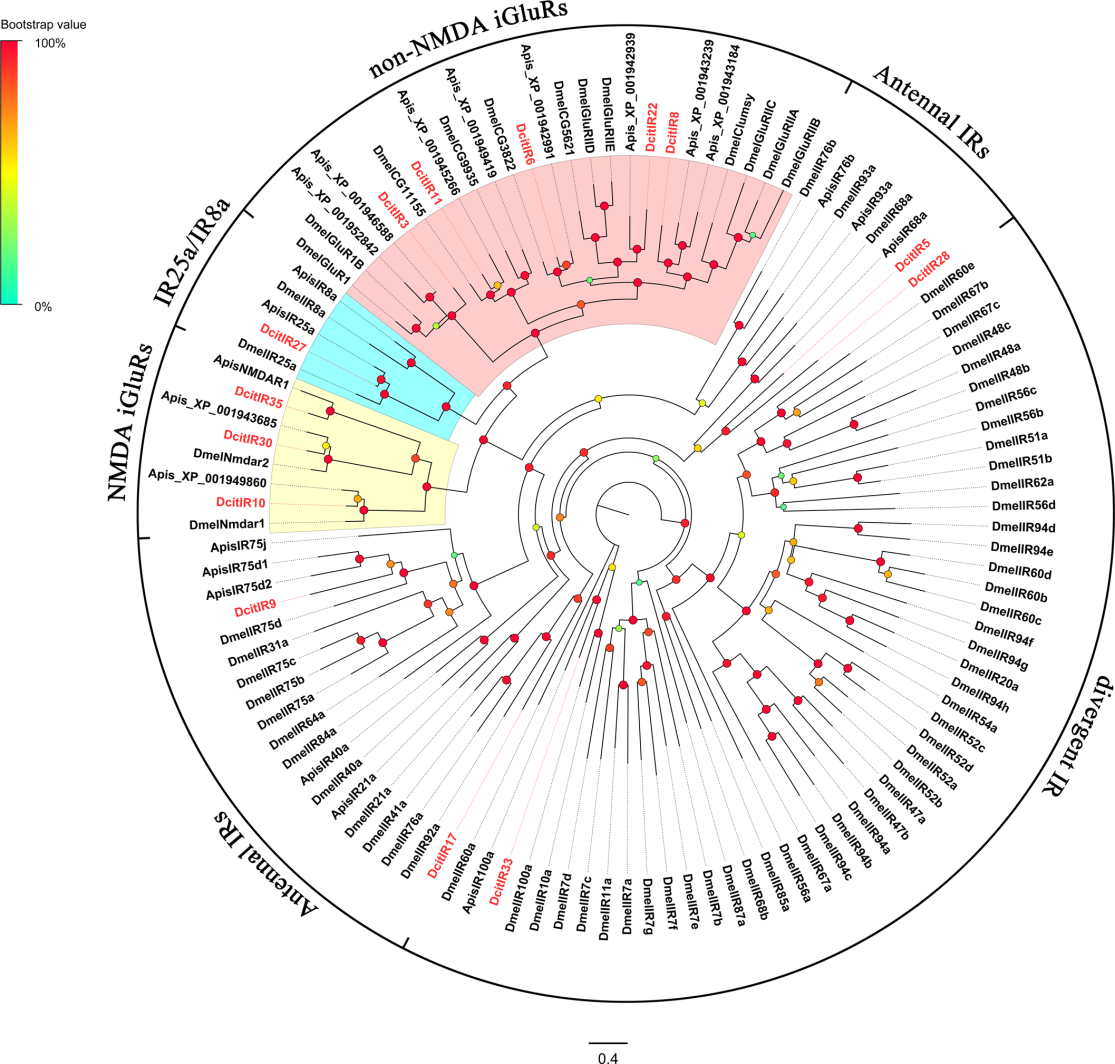
Phylogenetic relationship between DictIRs.

Thirty-five candidate IR transcripts were identified in the combined transcriptomes of *D*. *citri* and were annotated as known insect IRs and ionotropic glutamate receptors (iGluRs), and as the ligand-gated ion channel family of proteins (S5 Table). Eight of the transcripts encoded full-length CDS (DcitIR3, 5, 6, 8, 10, 22, 30, and 35), whereas the remaining were partial CDS. Based on structural analysis and amino acid sequence alignments, 8 iGluRs (DcitIR3, 6, 8, 10, 11, 22, 27, and 30) were identified with all residues typical for iGluRs (R, T, and D/E) [49], and 6 IR transcripts had mismatching amino acids at one or more positions indicating variable ligand binding properties (S4 Fig). According to the ML analysis of DcitIRs with *D*. *melanogaster* IRs, five DcitIRs (DcitIR3, 6, 8, 11, and 22) grouped together with the non-NMDA iGluRs clade; three DcitIRs (DcitIR10, 30, and 35) clustered with the NMDA iGluRs clade; one DcitIR (DcitIR33) was grouped together with the divergent IRs clade; DcitIR27 grouped with the co-receptors, IR8a and IR25a; and four DcitIRs (DcitIR5, 9, 17, and 28) grouped with the antennal IRs clade (Fig 6).

Larger numbers of candidate IR transcripts were expressed in the antennae when compared to the terminal abdomen (S7 Table). Two non-NMDA iGluR orthologs (IR3 and 6) and two NMDA iGluR orthologs (IR10 and 35) were upregulated in the antennae when compared to the terminal abdomen (Fig 3E; S7 Table). Four antennal IR orthologs (IR5, 9, 17, and 28) and IR co-receptor ortholog (IR27) with low expression levels showed no significant difference in expression in the antennae and terminal abdomen.

### Sensory neuron membrane proteins

**Fig 7:**
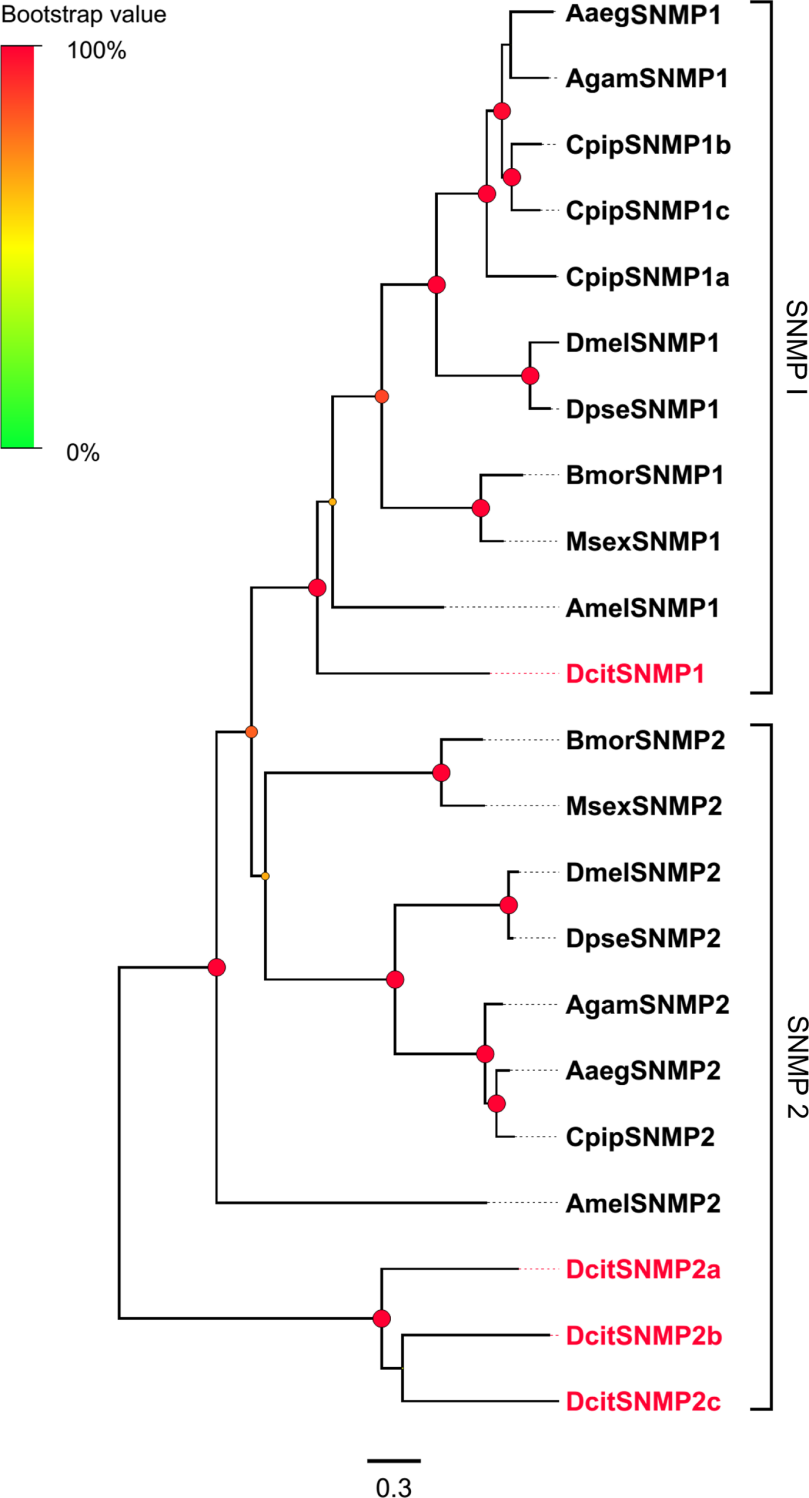
Phylogenetic relationship between DictSNMPs.

Four candidate SNMP transcripts, which were full-length genes, were identified in the combined transcriptomes from the two tissues of *D*. *citri* (S5 Table). Consistent with the characteristics of insect SNMPs, four candidate DcitSNMP transcripts contained two transmembrane domains. Phylogenetic analysis along with sequence alignment revealed the classification of four candidate DcitSNMP transcripts; one was the ortholog of SNMP1 (DcitSNMP1), and three were orthologs of SNMP2 (DcitSNMP2a, DcitSNMP2b, and DcitSNMP2c) (Fig 7).

All these SNMP transcripts were detected in the antennae, and three among them (except DcitSNMP1) were expressed also in male and female terminal abdomen. In addition, the expression of DcitSNMP2b was significantly higher in the male or female antennae than in the terminal abdomen (Red stars in Fig 3F; S7 Table). The remaining showed no significant difference in expression between the antennae and terminal abdomen among the sexes (S7 Table).

### Odorant degrading enzymes

In the olfaction processes, previous studies have implicated odorant degrading enzymes (ODEs) such as cytochrome P450s (CYPs) [50, 51], esterases (ESTs) [52–54], and aldehyde dehydrogenases (ADEs) [55–57] involved in the rapid inactivation of signals. A large number of putative odor/xenobiotic degradation gene transcripts were detected in our transcriptomes, including 80 CYP transcripts, 12 EST transcripts, and 5 ADE transcripts (S6 Table). Additionally, 35 among the 80 CYP transcripts, 10 among the 12 EST transcripts, and three among the five ADE transcripts had full-length CDS.

**Fig 8:**
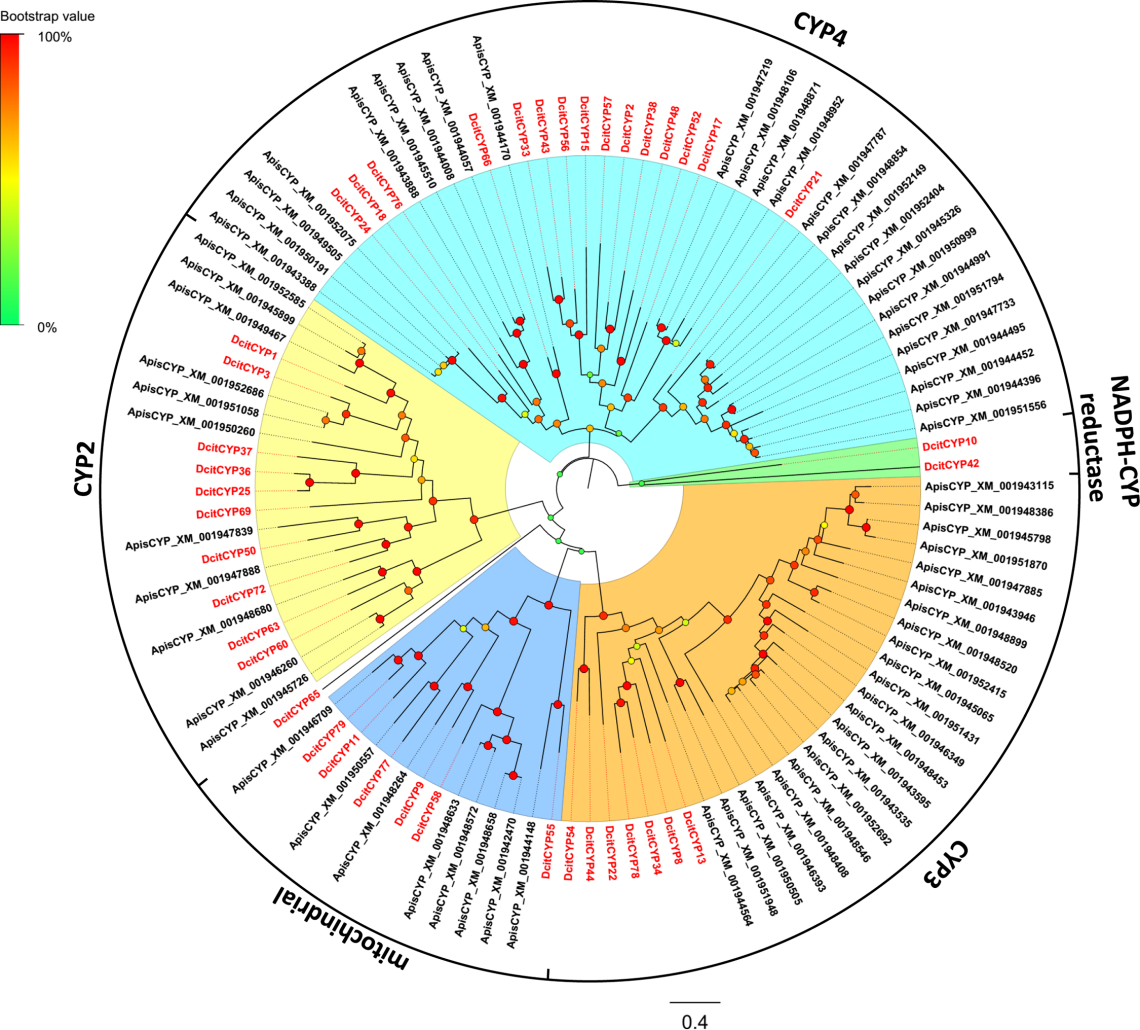
Phylogenetic relationship between DictCYPs.

Phylogenetic analysis revealed that the DictCYP transcripts clustered with four major cytochrome P450 gene families, CYP2, CYP3, CYP4, and mitochondrial clades (Fig 8). Notably, the majority of DictCYP transcripts (15 transcripts) were largely grouped into the CYP4 clade, which has previously been implicated in the metabolism of odorants or pheromones [58]. However, in the CYP4 clade, four CYP4 genes (DcitCYP18, DcitCYP66, DcitCYP76, and DcitCYP24 previously named as CYP4C67, CYP4G70, CYP4DA1, and CYP4DB1, respectively) involved in imidacloprid resistance [21] were clustered into one clade.

**Fig 9:**
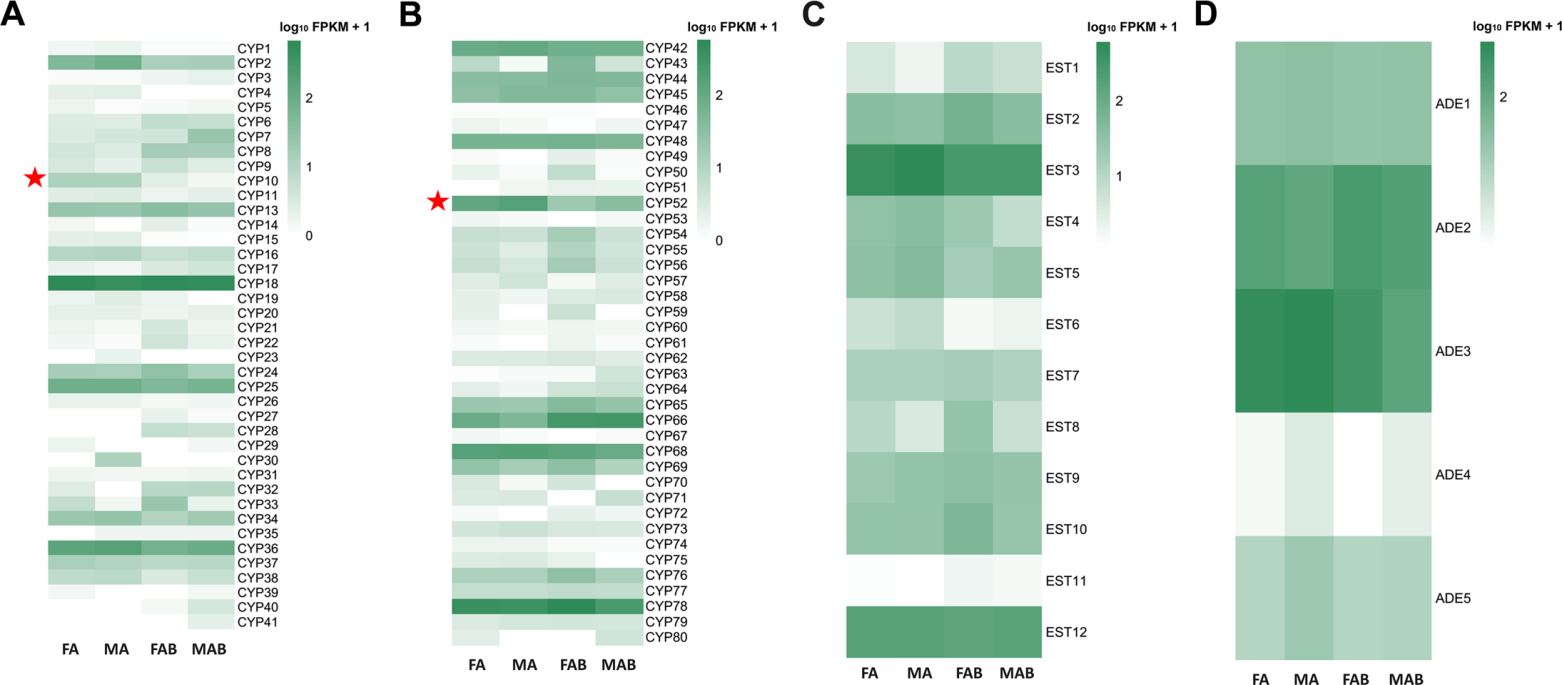
Expression profiles of odorant-degrading enzymes. A: CYP; B: EST; and C: ADE.

In addition, 2 CYPs (CYP10 and 52) had a higher expression level in the antennae than in the terminal abdomen (Fig 9A and 9B). Among them, DcitCYP52 from the CYP4 clade was associated with metabolizing odorants and/or pheromones [58]. The antennal-dominant DcitCYP10 clustered with the NADPH CYP clade, which has been shown to be involved in insecticide resistance [59]. In the other detoxification enzyme classes including ESTs and ADEs, none of the candidates were significantly upregulated in the antennae when compared to the terminal abdomen (Fig 9C and 9D; S7 Table).

### qPCR validation

**Fig 10:**
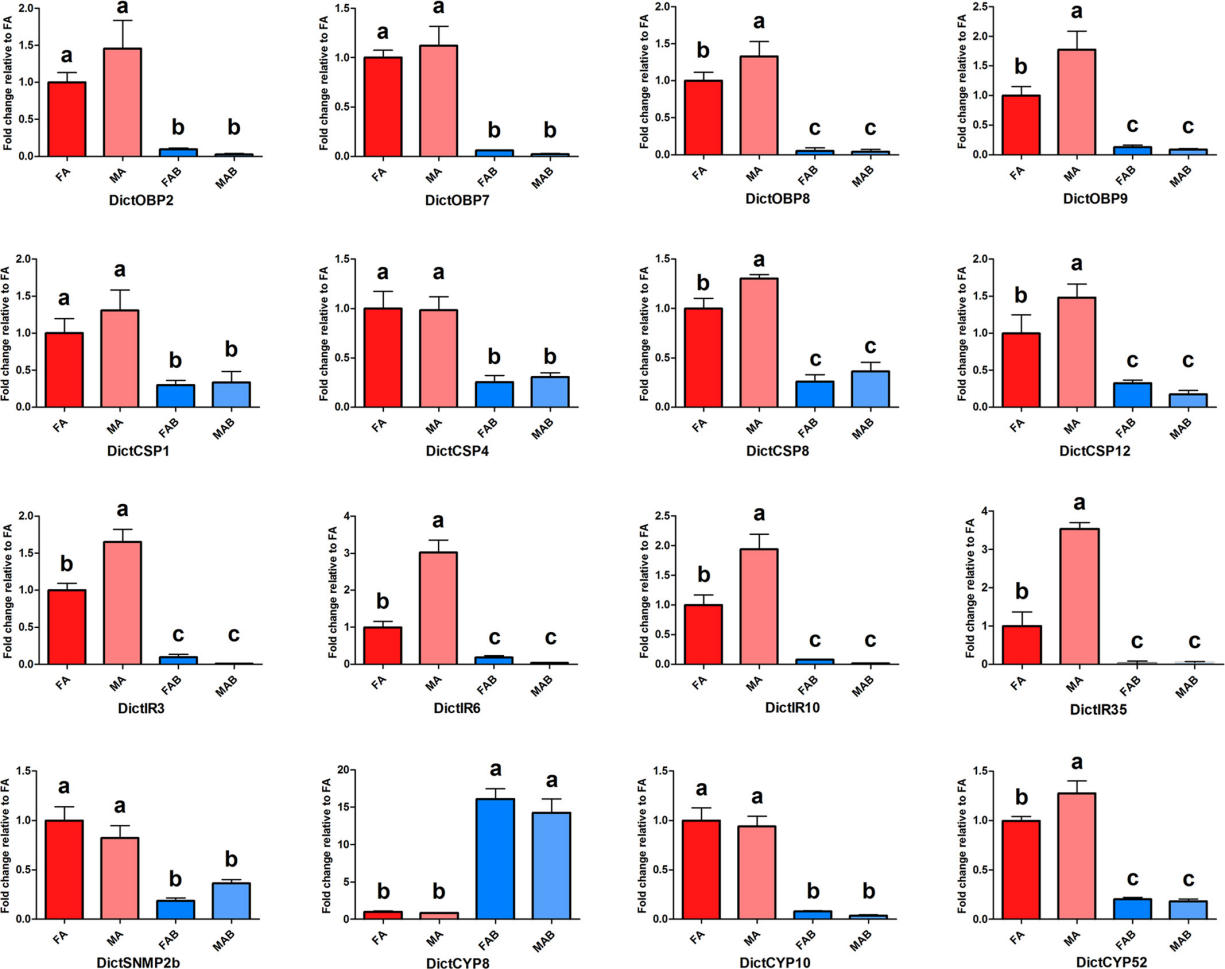
qPCR results of differentially expressed genes in the antennae and terminal abdominal tissues. Standard error is represented by the error bar. Different letters (a, b, c) above each bar denote significant differences (p<0.05).

Referring to the calculated expression values determined based on the FKPM methods, the chemosensory gene transcripts, including 4 DictOBP transcripts, 4 DictCSP transcripts, 4 DictIR transcripts, 1 DictSNMP transcripts, and 3 DictCYP transcripts were differentially expressed in the antennae and terminal abdominal tissues (S7 Table, red font), and were selected for qPCR validation. The qPCR results confirmed the higher expression of the following transcripts in the antennae compared to abdomens: 4 DictOBP transcripts (DictOBP2, 7, 8, 9), 4 DictCSP transcripts (DictCSP1, 4, 8, 12), 4 DictIR transcripts (DictIR3, 6, 10, 35), 1 DictSNMP transcript (DictSNMP2b), and 2 DictCYPs transcripts (DictCYP10, 52), which were found to be enriched in the antennae by RNA-seq (Fig 10). Additionally, two DictCYP transcripts were enriched in the terminal abdominal tissues (Fig 10). In addition, the qPCR data also showed that 2 DictOBP transcripts (DictOBP8 and DictOBP9), 2 DictCSP transcripts (DictOBP8 and DictOBP12), 4 DictIR transcripts (DictIR3, DictIR6, DictIR10, and DictIR35), and 1 DictCYP transcript (DictCYP57) were expressed at higher levels in the male antennae than in the female antennae (p < 0.05; Fig 10).

## Discussion

The Asian citrus psyllid is one of the most important insect pests in the world. Despite its devastating impact on citrus cultivation worldwide, effective control measures including monitoring and annihilation of this insect pest are not currently available. Transcriptome analysis along with genome annotation, has paved the way to identify and characterize multiple chemoreception genes in insects [60, 61]. More importantly, as potential molecular targets, chemosensory proteins can be used to identify novel attractants or repellants for use in environment-friendly pest management strategies [24–26, 62]. However, a lack in the genetic information of chemosensory genes has limited our understanding of the chemosensory mechanisms in this species. Besides, the molecular basis of chemoreception in hemipterans is poorly understood compared to dipterans and lepidopterans. In the present study, we compared the expression profiles of chemosensory genes in an olfactory (antennae) and a non-olfactory tissue (terminal abdomen) from male and female *D*. *citri* to identify olfaction-specific genes for use as novel targets in pest control.

### Odorant binding proteins and chemosensory proteins

Odorants penetrate into the sensillum lymph through pore tubules and bind to OBPs forming the odorant·OBP complex. [63] After OR activation, odorants transported to the OR by OBPs and then released [5]. Similarly, CSP are also found in olfactory and gustatory organs of insects [64–66], and are suggested to play crucial roles in chemo-detection [67]. The 9 candidate OBP transcripts (seven Classic and two Plus-C OBPs) and 12 CSP transcripts identified in *D*. *citri* were more similar to the numbers identified previously in the aphid species (*A*. *pisum*: 13 Classic and two Plus-C OBPs and 13 CSPs; *A*. *gossypii*: 9 OBPs and 9 CSPs) and another hemipteran (*N*. *lugens*: 8 Classic and two Plus-C OBPs and 11 CSPs) [27–31]. Notably, without Minus-C OBP transcripts, only Classic OBP transcripts and Plus-C OBP transcripts were found in the psyllid and other hemipterans. This may indicate their origin from a common ancestor. RNA-seq data and qPCR analyses showed that 4 DictOBP transcripts and 4 DictCSP transcripts were significantly enriched in antennae when compared to the terminal abdomen, suggesting the possible involvement of these antennal OBP and CSP proteins in odor perception. In addition, qPCR data revealed that DictOBP8, DictOBP9, DictCSP8, and DictCSP12 were expressed higher in the male antennae than in the female antennae. Male-biased expression of these genes implicates their importance in the perception of female sex pheromones [64, 68].

### Odorant receptors

Insect ORX/Orco heteromers are known to function as odorant-gated ion channels, and consist of a highly conserved co-receptor (Orco) and odorants-recognizing receptor (ORX) [69–72]. In general, it is known that ORs employ a higher number of receptor protein families to detect a broad range of odors [1], and that the members of insect ORs show lineage specific expansions that might have been driven by ecological adaptation [73]. Compared to two phytophagous hemipterans with known OR sets, the number of *D*. *citri* ORs were close to that reported for the cotton aphid *A*. *gossypii* ORs (45 in genome and 36 in transcriptome of the antennae and decapitated body parts) [33], but lower than in the pea aphid *A*. *pisum* genome (79) [32]. Considering that the host range of *D*. *citri* is relatively narrow and is within the rutaceous subfamily Aurantioideae [18], we presumed that the number of candidate OR transcripts in *D*. *citri* was smaller due to lower “semiochemical diversity”. Although *D*. *citri* is a phytophagous hemipteran, *D*. *citri* ORs share low homology with the two aphids, and all but the conserved co-receptor (DcitOR1) have no homology with their ORs according to phylogeny, indicating a significant species-specific expansion and divergence in *D*. *citri*. Surprisingly, the OR repertoire of *D*. *citri* was not mainly expressed in antennae, which is not consistent with OR expression in other insect species including *D*. *melanogaster* [74], *Anopheles gambiae* [75], *Culex quinquefasciatus* [60], and *Mayetiola destructor* [76]. It is therefore possible that a majority of DictOR transcripts have other non-olfactory functions.

### Gustatory receptors

In mosquito and *Drosophila*, the GR complex consists of two or three GR members that play a crucial role in CO_2_ detection [48, 77–80]_._ Only one GR homolog of insect CO_2_ receptors was found in *D*. *citri* and it had low expression in the antennae. In general, insect CO_2_ receptors are highly expressed in the antennae. We presume that an alternate organ such as the maxillary palp may be responsible for CO_2_ detection in this insect pest [77, 81]. In addition, we cannot rule out the possibility that sequencing depth in this study may have been the reason for identifying low abundance putative CO_2_ receptors in antennal transcriptomes. A large number of putative sugar receptor transcripts were also observed in the antennae and terminal abdomen. Although the exact reason for this is not known, it likely that they evolved to adapt for feeding or egg laying habits. Both male and female *D*. *citri* have a high preference for feeding on tender shoots, and females lay eggs only in the exocuticle of the earliest tender shoots [18].

### Ionotropic receptors

IRs were recently identified as a novel class of insect olfactory receptors [82]. Functional studies of insect IRs expressed in olfactory neurons have demonstrated their role in the detection of amines, acids [49, 83] and DEET [62]. More recently, a few members of IRs expressed in taste neurons have also been confirmed as a new-type of larval taste receptors [84] and were reported to be involved in the detection of pheromones [85]. We identified 6 putative DcitIR transcripts and 8 iGluRs transcripts based on the amino acid sequence alignment (S4 Fig). However, the search for IR co-receptors, IR8a and IR25a, revealed that only DcitIR27 in *D*. *citri* was homologous to insect IR25a receptors. Interestingly, another homologous IR-co-receptor, IR8a, was not discovered in the four transcriptomes assembly. A similar expression pattern was also reported in *Culex quinquefasciatus* [60]. We therefore concluded that it is likely that DcitIR8a is not expressed in *D*. *citri* antennae and terminal abdomen. Also, this lack of identification may be related to the low sequencing depth in this study. In addition, two non-NMDA iGluR orthologs (DcitIR3 and 6), three DcitIRs (DcitIR10, 30 and 35), and two NMDA iGluRs orthologs showed a clear difference in expression level between the sexes (Figs 6, 3E and 10). In *D*. *melanogaster*, male-biased patterns of IR expression as well as functional analyses revealed that both IR52c and IR52d may determine male copulation [84]. Therefore, it is presumed that the DcitIR transcripts that showed sex-biased expression could play a role in sexual behavior.

### Sensory neuron membrane proteins

SNMPs in insects, which are located in the odorant-sensitive ORNs, are a family of membrane proteins with two transmembrane domains and are presumed to be associated with pheromone reception in lepidopteran and dipteran insects [86–89]. In Lepidoptera, the SNMP family consists of two subfamilies, SNMP1 and SNMP2, which are differentially expressed in the cells of pheromone-sensitive sensilla.SNMP1 is expressed in the pheromone-specific olfactory neurons suggesting its involvement in pheromone detection [89, 90] while SNMP2 is expressed in the sensilla support cells [89]. In *D*. *melanogaster*, SNMP1 homolog is essential for the proper olfactory sensory neuron (OSN) responses to the volatile pheromone, 11-cis-vaccenyl acetate [91]. In this study, one SNMP1 and three homologs of SNMP2 were identified *in D*. *citri*, and all these SNMPs were expressed in both the antennae and terminal abdomen. Not surprisingly, the expression of SNMP1 and SNMP2 in the antennae and the remaining body parts including terminal abdominal tissues was also reported in moths and flies [76, 92]. Since SNMP1 homolog has key functions in pheromone detection in *D*. *melanogaster*, we presumed that DictSNMP1 transcripts would be expressed at the maximum levels in the antennae. However, only DictSNMP2b had highest expression in the antennae compared to terminal abdomen. This finding suggests that DictSNMP2b may play a specific role in pheromone detection, or alternatively that the role of DictSNMP1 might not be restricted to detect pheromones in psyllids.

### Odorant degrading enzymes

CYP4s have been linked to odorant or pheromone metabolism in some insect species [50, 51, 93–97]. DictCYP52, which aligned with the known CYP4 clade, was found to be highly expressed in the antennae than in the terminal abdomen, and higher in male antennae based on qPCR data. Thus, we presume that DictCYP52 could play a role in odorant clearance. However, further studies along this line is warranted.

## Conclusions

To better understand the chemosensory genes involved in olfactory sensation, sex pheromone production and host selection behavior in *D*. *citri*, we analyzed its antennal and abdominal transcriptome in both sexes. A total of 126 chemosensory gene transcripts (excluding ODEs), including 9 OBPs, 12 CSPs, 46 ORs, 35 IRs, 20 GRs, and 4 SNMPs were identified in the present study. In addition, a group of detoxification enzymes potentially linked to odorant degradation such as 80 cytochrome P450s, 12 esterases, and 5 aldehyde dehydrogenases were also identified. Furthermore, the expression of all these chemosensory gene transcripts was analyzed based on transcriptome profiling using RNA-seq data. Both RNA-seq data and qPCR analyses revealed that a number of these newly identified genes in *D*. *citri* transcriptomes were differentially expressed in the four tissues, thus indicating their role in the interaction between the olfactory system and biological processes.

## Supporting Information

S1 Fig Amino acid sequence alignment of *D*. *citri* and other hemipteran ‘Classic’ OBPs. (TIF)

S2 Fig Amino acid sequence alignment of *D*. *citri* and other hemipteran ‘Plus-C’ OBPs. (TIF)

S3 Fig Amino acid sequence alignment of *D*. *citri* CSPs. (TIF)

S4 Fig Amino acid sequence alignment of *D*. *citri* IRs. (TIF)

S1 Table Primers used in qPCR analysis. (XLSX)

S2 Table Summary of the *D*. *citri* antennal and abdominal transcriptomes. (XLSX)

S3 Table Annotation statistics of the assembled unigenes. (XLSX)

S4 Table Candidate odorant-binding receptors identified in the combined tissue transcriptomes. (XLSX)

S5 Table List of *D*. *citri* chemosensory genes putatively involved in chemosensory reception. (XLSX)

S6 Table List of *D*. *citri* chemosensory genes putatively involved in odorant degradation. (XLSX)

S7 Table Differentially expressed chemosensory-related genes in antennae and terminal abdominal tissues. (XLSX)

S1 Text Amino acid sequences from published data in phylogenetic analyses. (DOCX)
