# Supplementary figures and images for: Antennal and Abdominal Transcriptomes Reveal Chemosensory Genes in the Asian Citrus Psyllid, Diaphorina citri
Source: PLoS One. 2016 Jul 21;11(7):e0159372. doi: 10.1371/journal.pone.0159372 (PMC4956155; doi:10.1371/journal.pone.0159372)

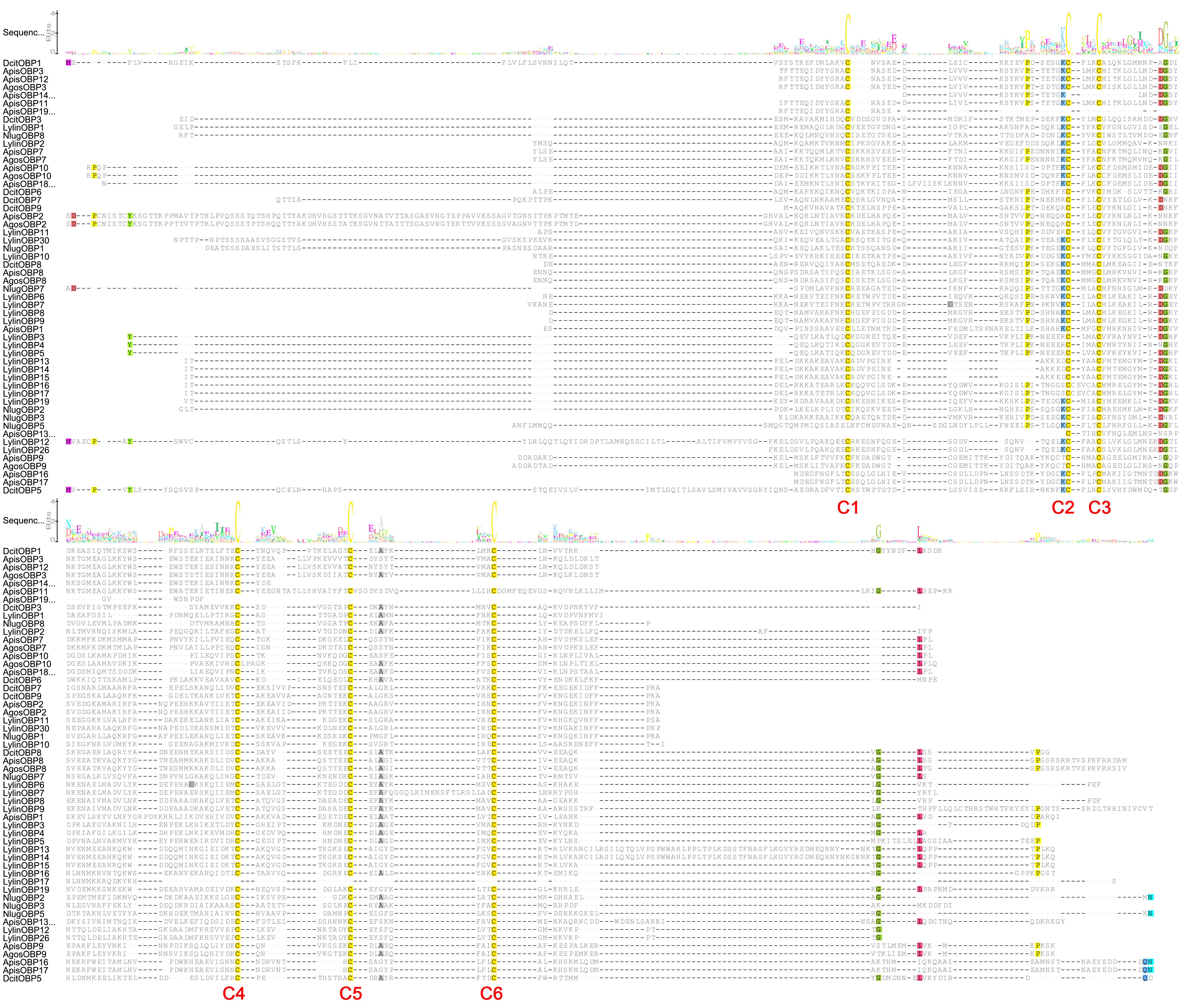

Supplement: S1 Fig — (TIF) [file pone.0159372.s001.tif]

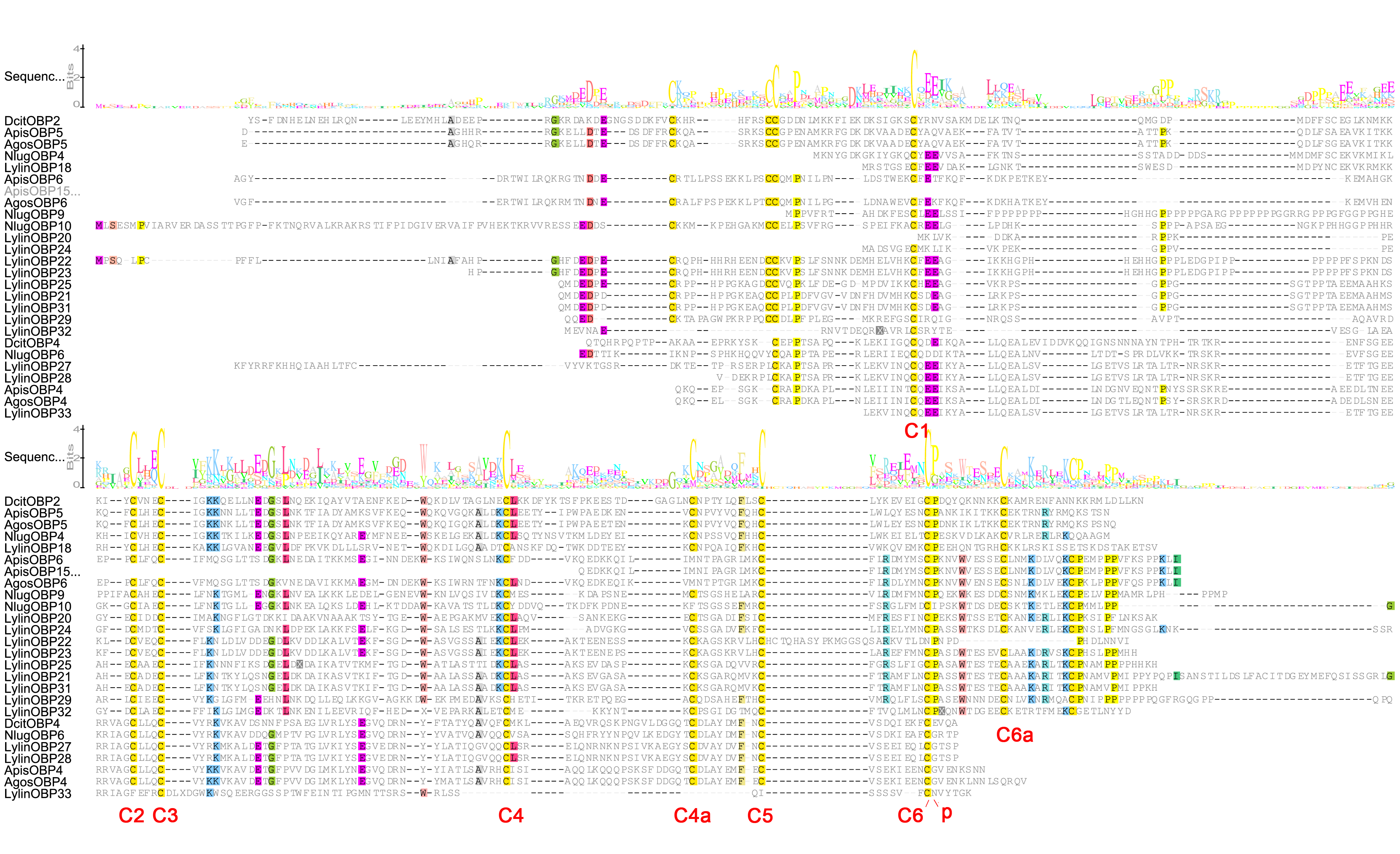

Supplement: S2 Fig — (TIF) [file pone.0159372.s002.tif]

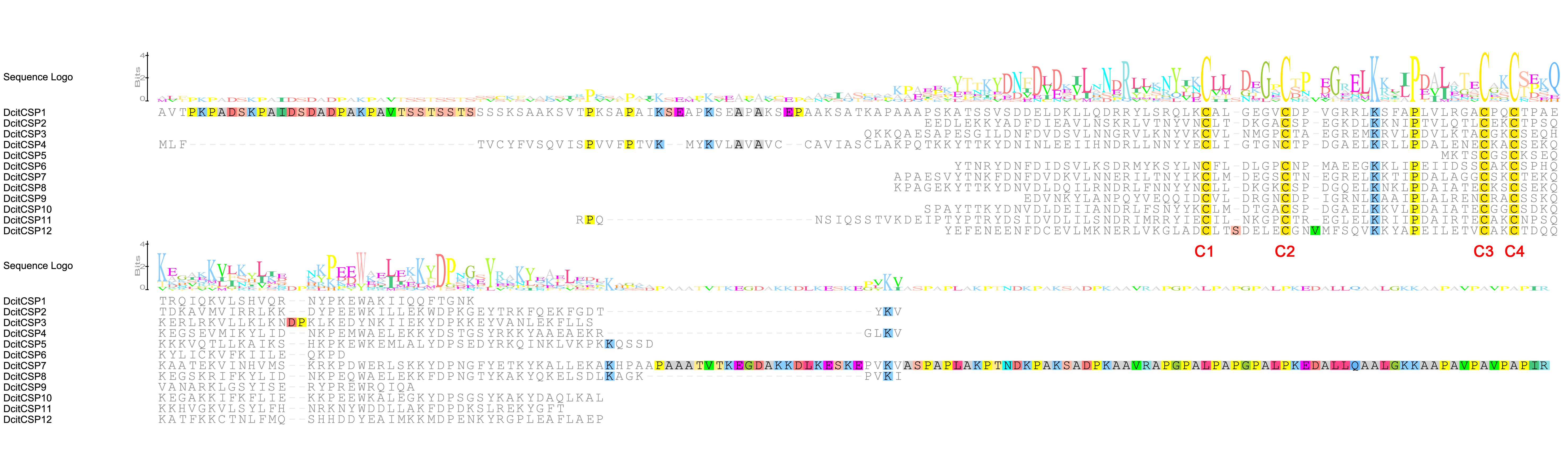

Supplement: S3 Fig — (TIF) [file pone.0159372.s003.tif]

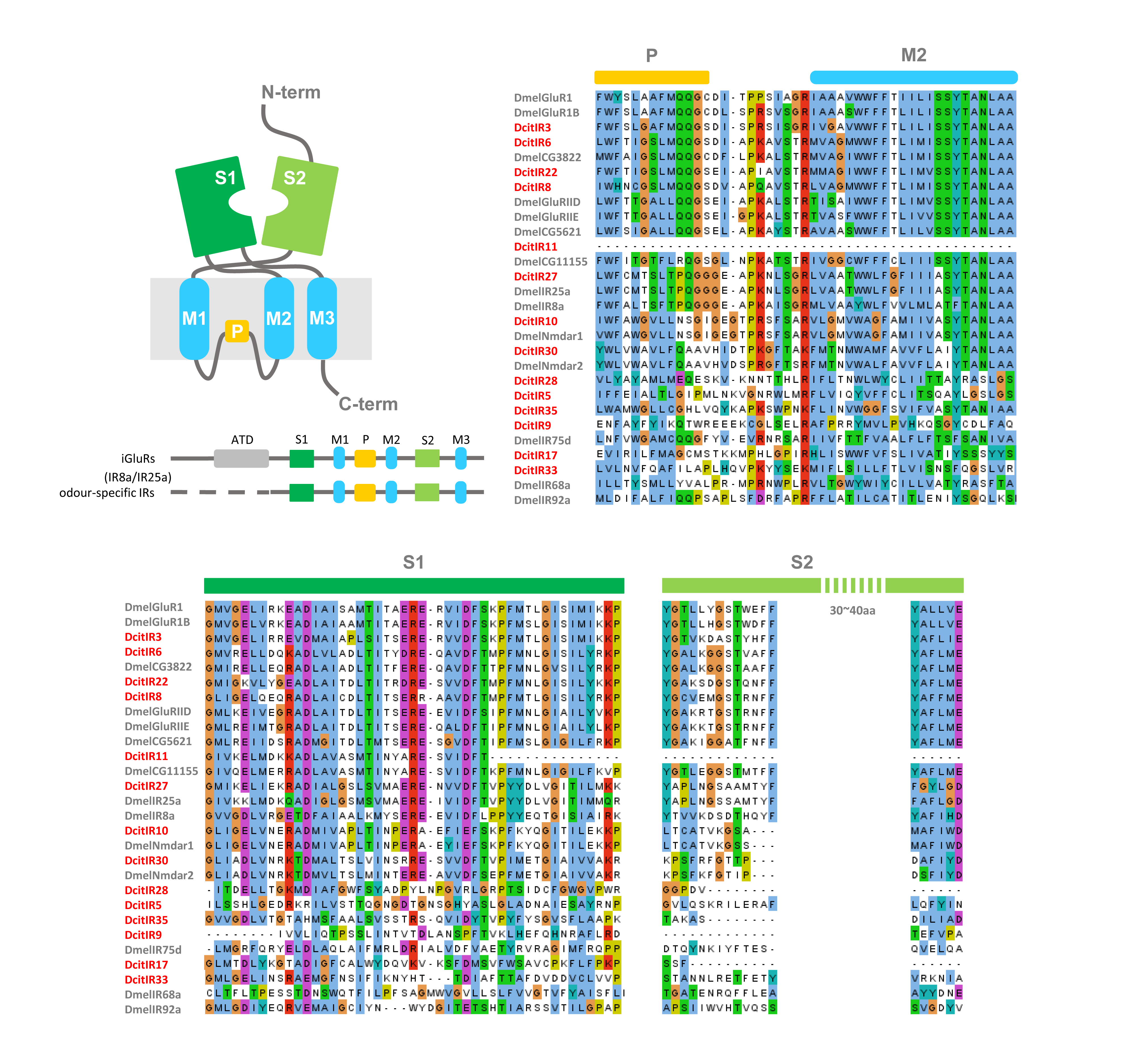

Supplement: S4 Fig — (TIF) [file pone.0159372.s004.tif]
