# Supplementary material for: Antennal and Abdominal Transcriptomes Reveal Chemosensory Genes in the Asian Citrus Psyllid, Diaphorina citri
Source: PLoS One. 2016 Jul 21;11(7):e0159372. doi: 10.1371/journal.pone.0159372 (PMC4956155; doi:10.1371/journal.pone.0159372)
Supplement: S1 Text — (DOCX) [file pone.0159372.s012.docx]

**Amino acid sequences from published data in phylogenetic analyses**

The OBP data set contained 17 sequences from *Acyrthosiphon pisum* [1, 2], 9 sequences from *Aphis gossypii* [3], 33 sequences from *Lygus lineolaris* [4], and 10 sequences from *Nilaparvata lugens* [5-7]. The CSP data set contained 13 sequences from *A. pisum* [1, 2], 9 sequences from *A. gossypii* [3], 11 sequences from *N. lugens* [5, 7], 3 sequences from *Adelphocoris lineolatus* [8].The OR data set contained 63 OR sequences from *Drosophila melanogaster* [9, 10] and 79 sequences from *A. pisum* [11]. The IRs data sets contained 66 IR sequences from *D. melanogaster*. The SNMP data set contained 18 SNMP sequences from Diptera, Lepidoptera and Hymenoptera [12]. The CYP data set contained 67 CYP sequences from *A. pisum* [13].

**References**

1. Zhou JJ, Vieira FG, He XL, Smadja C, Liu R, Rozas J, et al. Genome annotation and comparative analyses of the odorant-binding proteins and chemosensory proteins in the pea aphid *Acyrthosiphon pisum*. Insect Mol Biol. 2010; 19 Suppl 2:113-122.
2. Vieira FG, Rozas J. Comparative Genomics of the Odorant-Binding and Chemosensory Protein Gene Families across the Arthropoda: Origin and Evolutionary History of the Chemosensory System. Genome Biol Evol. 2011; 3:476-490.
3. Gu S, Wu K, Guo Y, Field LM, Pickett JA, Zhang Y, et al. Identification and Expression Profiling of Odorant Binding Proteins and Chemosensory Proteins between Two Wingless Morphs and a Winged Morph of the Cotton Aphid *Aphis gossypii* Glover. PloS one*.* 2013; 8(9):e73524.
4. Hull JJ, Perera OP, Snodgrass GL. Cloning and expression profiling of odorant-binding proteins in the tarnished plant bug, *Lygus lineolaris*. Insect Mol Biol. 2014; 23(1):78-97.
5. Zhou SS, Sun Z, Ma W, Chen W, Wang MQ. De novo analysis of the *Nilaparvata lugens*(Stål) antenna transcriptome and expression patterns of olfactory genes. Comp Biochem Physiol Part D Genomics Proteomics. 2014; 9:31-39.
6. He P, Zhang J, Liu NY, Zhang YN, Yang K, Dong SL. Distinct expression profiles and different functions of odorant binding proteins in *Nilaparvata lugens* Stal. PloS one. 2011; 6(12):e28921.
7. Xu YL, He P, Zhang L, Fang SQ, Dong SL, Zhang YJ, Li F. Large-scale identification of odorant-binding proteins and chemosensory proteins from expressed sequence tags in insects. BMC Genomics. 2009; 10:632.
8. Gu SH, Wang SY, Zhang XY, Ji P, Liu JT, Wang GR, et al. Functional characterizations of chemosensory proteins of the alfalfa plant bug *Adelphocoris lineolatus* indicate their involvement in host recognition. PloS one. 2012; 7(8):e42871.
9. Robertson HM, Warr CG, Carlson JR. Molecular evolution of the insect chemoreceptor gene superfamily in *Drosophila melanogaster*. P Natl Acad Sci USA. 2003; 100 Suppl 2:14537-14542.
10. Gao Q, Chess A. Identification of candidate Drosophila olfactory receptors from genomic DNA sequence. Genomics. 1999; 60(1):31-39.
11. Smadja C, Shi P, Butlin RK, Robertson HM. Large Gene Family Expansions and Adaptive Evolution for Odorant and Gustatory Receptors in the Pea Aphid, *Acyrthosiphon pisum*. Mol Biol Evol. 2009; 26(9):2073-2086.
12. Vogt RG, Miller NE, Litvack R, Fandino RA, Sparks J, Staples J, et al. The insect SNMP gene family. Insect Biochem Molec. 2009; 39(7):448-456.
13. Duvaux L, Geissmann Q, Gharbi K, Zhou JJ, Ferrari J, Smadja CM, Butlin RK. Dynamics of copy number variation in host races of the pea aphid. Mol Biol Evol. 2015, 32(1): 63-80.

**OBP sequences**

>ApisOBP1

MLNLKVMMFLCLSVIVVYCESDQVPINSSAAVESCLLETNMTRDEFEDMLTSPNARELTILKSHAHKCMFGCVMRKNHIVNDGVVSKEVLSKYVLNFYGRPDYKRRLIIKDVEHIVDVCAKKVADESETDECELAATLVTCIVLEANKAGLVDDPARQI

>ApisOBP2

MKVSAATAVLVALVATVQSSDPCNISTCYKSGTTKPPMAVTPTHLPVQSSSTQTSHPQTTYAKDHVHGSTTTKSGVNATVTTASGASVNGTEPPAVVKSSAGVTGNSTTPKPTMTEGHVALKQKLNTIAVKCKDELHAPQEIMALVSNTVVPQNEQQRCYLECVYKNLNLIKNNKFSVEDGKAMARIRFANQPEEHKKAVTIIETCEKEAVIDPKTTEKCAAGRVIRNCFVKNGEKINFFPKA

>ApisOBP3

MNSSTFYITFLFGIAMLISCGYGTFTTEQIDYYGRACNASEDDLVVVKSYKVPSTETGKCLMKCMITKLGLLNDDGSYNKTGMEAGLKKYWSEWSTEKIEAINNKCYEEALLVPKEVVVTCDYSYTVMACLNKQLDLDKLT

>ApisOBP4

MRGNYSSMVFLLFAIGFQDIFCQKQEPSGKCRAPDKAPLNLEIIINTCQEEIKSALLQEALDILNDGNVEQNTPNYSSRSKREAEEDLTNEERRVAGCLLQCVYKKVKAVDETGFPVVDGLMKLYNEGVQDRNYYIATLSAVRHCISIAQQLKQQQPSKSFDDGQTCDLAYEMFECVSEKIEENCGVENKSNN

>ApisOBP5

MSANSATIKCIAVAAILLQISVIFADAGHHRRGKELLDTEDSDFFRCKQASRKSCCGPENAMKRFGDKDKVAADECYAQVAEKFATVTATTPKQDLFSAEAVKITKKKQFCLHECIGKKNNLLTEDGSLNKTFIADYAMKSVFKEQWQKQVGQKALDKCLEETYIPWPAEDKENVCNPVYVQFQHCLWLQYESNCPANKIKITKKCEKTRNRYRMQKSTSN

>ApisOBP6

MQKVVFICIFAIICQTVFTAGYDRTWILRQKRGTNDDECRTLLPSSEKKLPSCCQMPNILPNLDSTWEKCFETFKQFKDKPETKEYKEMAHGKEPPCLFQCIFMQSGLTTSDGKLNEDAITKKMSEGINNDEKWKSIWQNSLNKCFDDVKQEDKKQILIMNTPAGRLMKCFLRDMYMSCPKNVWVESSECLNMKDLVQKCPEMPPPVFKSPPKLI

>ApisOBP7

MVARKRMYNMLPTTVLFAIIAATVLKDCDAYLSEAAIKKTQQMLKTVCSKKHSVEEDVFTNIKKGIFPEDNNNIKCYFACNFKTMQLINQKGVIDKKMFKDKMSMMAPPNVYKILLPVIEQCTGKDKGEELCQSSYNVIKCAHSVDPKSLEFLPL

>ApisOBP8

MFALKVACLCLSVAVVFGENNQQNGPSDRSATIFQSCIAETKLSGDALKGFRSMSIPKTQAEKCMMGCLMRKVNVINKGKFSVEEATKVAQKYYGTNEAMMKKAKDLIDVCAKKAQSTTEECALAGIVTTCIVEEAQKAGLSGGPGSRSRRTVSPKFRRDAM

>ApisOBP9

MIIKKTLLLSVFVLFGCLFSINKADDADAKDKELMSKLFTVVFKCFKDADWGTCGEMITTKYDITQAKYKQCTCHMACAGEELGMINASGQPEPAKFLEYVNKINNPDIKSQLQLIYDKCQNVKGSEKCDLAEQFAICAFKESPALKERVSTLMEMLVKMKPKSK

>ApisOBP10

MEHLRSTNVVFAIVMALLVVQSSTRPQPDEMEEIKRTLYNACAGKFPITEEIKNNAKNSIISDDPTFKCFLKCCFDEMSMIDEDGIIDGDSLKAMAPDHIKPILEQVIPSCTKNVKQDGCEASFEFISCGIKLNPLIVALLPL

>ApisOBP11

MSSSTFYITLLFGIAMLISCGYGIFTTEQIDYYGKACNASEDDLIVLKSYKVPSTETGKCLMKCMITKLGLLNDDGSYNKTGMEAGLKKYWSEWATEKIETINEKCYEEGNTATLLYHVAIYFTCVSGDYSDVQLLIHCDGMFEQEVGSRQVNLKLLIMLKIGLSEPKR

>ApisOBP12

MISSTFYITLVFGIAMLISCGHGRFTTEQIDYYGKACNASEDDLVVVKSYKVPTTETGKCLMKCMITKLGLLNDDGSYNKTGMEAGLKKYWSEWSTEKIESINNKCYEEALLVSKEVVATCNYSYTVMACLNKQLDLDKST

>ApisOBP13_N

CTIHCVFNQLEMLNSNSRPDKYSIVNIMTNQIKDVELKEFIQDSIDECFDTLELDSHNNKCEFSKNFAVCMENKAQRNCDDWDENLSANKINSAGLQDGTNQQDKRKGY

>ApisOBP14_N

DLVVVKSYKVPSTETGKLNDDGSYNKSGMEAGLKKYWLEWSTEKIEAINNKCYDE

>ApisOBP15_N

QEDKKQILIMNIPAGRLMKCFLRDMYMSCPKNVWVESSECLNMKDLVQKCPEMPPPVFKSPPKLI

>ApisOBP16

MDEGFNGFLTCSSQLGLNIEVCSDLLDPNLNSSDTKYDGCKCFLPCMAKIIGTMNTDDGKWNEKRFWEITAMLNVPDWKHEAEVIGNNCNDRVNTHCSAGYPLFLCALKHSKMLQDMAKTHMIQKQAAIEAMNSTNAEYEDDDQN

>ApisOBP17

MDEGFNGFLTCSSQLGLNIEVCSDLLDPNLNSSDTKYDGCKCFLPCMAKIIGTMNTDDGKWNEKRFWEITAMLNVPDWKHEAEVIGNNCNDRVNTHCSAGYPLFLCALKHSKMLQDMAKTHMIQKQAAIEAMNSTNAEYEDDDQN

>AgosOBP2

MKVSAATAVLVALVATVQSSDPCNISTCYKSGTTKPPTTVTPTRLPVQSSSTPTSHQQTTYAKDHVHSSTATKSGVNTTATTTSGASVNGTERTTVVKSSSGVAGNVTTPKPTMTDGHVALKQKLNTIAVKCKDELHAPQEIMALVSNTVVPQNEQQRCYLECVYKNLNLIKNNKFSVDDGKAMAKIRFANQPEEHKKAVTIIETCEKEAIIDPKTTEKCAAGRVIRNCFVKNGEKINFFPKA

>AgosOBP3

MISSTFYTSLMFGIAMLISCSFGRFTTEQIDHYGKACNATEDDLVIVKSYKVPTSDTGKCLMKCMISKLGLLNDDGSYNKTGMEAGLKKYWSEWSTDTIESINNKCYEEALLVSKDIIATCNYAYVVMACLNKQLKLDNST

>AgosOBP4

MRGNYSLVVFLLFGFGLLEIYCQKQELSGKCRAPDKAPLNLEIIINICQEEIKSALLQEALDILNDGTLEQNTPSYSRSKRDADEDLSNEERRVAGCLLQCVYKKVKAVDETGFPVVDGLMKLYNEGVQDRNYYMATLSAVRHCISIAQQLKQQQPSKSFDDGQTCDLAYEMFECVSEKIEENCGVENKLNNLSQRQV

>AgosOBP5

MKMSANGATMKCVAVAVVLFQMSVIFAEAGHQRRGKELLDTEDSDFFRCKQASRKSCCGPENAMKRFGDKDKVAADECYAQVAEKFATVTATTPKQDLFSGEAVKITKKKQFCLHECIGKKNKLLTEDGSLNKTFIADYAMKSVFKEQWQKQIGQKALDKCLEETYIPWPAEETENKCNPVYVQFQHCLWLEYESNCPDNKIKLTKKCEKTRNRYRMQKSPSNQ

>AgosOBP6

MQKVVFLCIFAIICQTVFTVGFERTWILRQKRMTNDNECRALFPSPEKKLPTCCQMPNILPGLDNAWEVCFEKFKQFKDKHATKEYKEMVHENEPPCLFQCVFMQSGLTTSDGKVNEDAVIKKMAEGMDNDEKWKSIWRNTFNKCLNDVKQEDKEQIKVMNTPTGRLMKCFLRDLYMNCPKNVWVENSECSNLKDLVEKCPKLPPPVFQSPPKLI

>AgosOBP7

MNMLPATVLLAVVAATILKDSDAYLSEEAIKKTQKMLKNVCSKKHSVEEEVFTDIKKGIFPENNNNIKCYFACNFKTMQMVNQKGILDKKMFKDKMTMLAPPNVLAILLPPIEQCIGNDKDTEICQSSYNFIKCAHRVDPKSLEFLPL

>AgosOBP8

MFAFKVACLCLSVAVVFGENNQQNSNDRSASIFQSCISETKLSGDALKGFRSMSIPKTQAEKCMMGCLMRKVNVINKGKFSVEEATKVAQKYYGTNESMMKKAKDLIDVCAKKAQSTTEECALAGIVTTCIVEEAQKAGLTGGPGSRSKRTVSPKFRHSIV

>AgosOBP9

MIIKKTLLVSGFVLFGCMFSINKAADDADTADKELMSKLITVAFKCFKDADWGTCGEMITTKYDITQAKYKQCTCHMACAGEDLGLINSNGQPEPAKFLEYVKRINNSVIKSQLQHIYDKCQNVKGTEKCDLAEQFAICAFKESPEMKERVTKLIEMLVKMKPKSK

>AgosOBP10

MEHLRGTNVMFAIVMALLVVQSSTRPQPDEPDDIKKTLYNACSEKFPLTEEIKNNVKNSMVIDDQNFKCFLRCCFDEMSLIDEDGIIDGESLAAMAVDKIKPVAEKIVHDCLPAGKQEKQDGCEAAFKFFSCGMKLNPLTIELLPLQ

>LlinOBP1

MRILVLFTAALTCVMAGELPEEMREMAQGLHDGCVEETGVDNGLIGPCAKGNFADDQKLKCYFKCVFGNLGVISDEGELDAEAFGSILPDNMQELLPTIRGCAGTTGADPCELAMNFNKCLQKVDPVNFMVI

>LlinOBP2

MVLKMSLLLVVFVASQVLISTTEAYMSQAQMKQAMKTVRNMCIPKSGVAKEALAKMVEGEFDDSDQKLKCYLGCVLGMMQAVKNNKINLTMVRNQISKMLAPEQGQRILTAFEGCATVTGDDNCDLAFKFAKCIYDTDKELLFQAFIVP

>LlinOBP3

MAVNAKAVLFLALCGLVYVSAYQEVLKATLQDCKGGKEITQEEVDEFVKPLIPKNEEERCLMACVFRAYNVIVDGHFDPKLAYGVAKNILHENPEKLKHIKETLDYCGHEIPTKMDNECDLAGEVMACRNKYNKDHGYDQDP

>LlinOBP4

MSIKIHFFVFAAIGLACVCAYQEQLKQTIKDCQGGKEVTDDELEEFTKPLIPKNEEERCIMACVMRTYNIINNGHYDPKIAFGILKGILKDHPEKLNKIKEVMDHCGEDVPQHMDNECDLAGEIMQCEVKYQKAMGLA

>LlinOBP5

MTTKLRSVGMIVAVTIAYVCAYQEQLKATIQKCQDGREVTDDEVEEFTKPLIPKNEEERCLVACVFKEYKVIIDGHFDPVNALNVAKMVYKEYPEKWERIRDVIDHCGEDIPTHNDNECDLAGDIMKCEVKYLNSMPKITSLELLAGSIAATEEP

>LlinOBP6

MKFVLSAAVVLLVAAAVKANEKKANEKVTEIFNKCKETWPVTDEEIEQVKQKQSIPESKNVKCILACMLKEAKILRDGEYNKENAELMADVLYKDEPEHAXKSKQIIEMCSAELGTKTEGDDCEYAYKMSVCASKHAKELGVKTPEF

>LlinOBP7

VKANEKKANEKVTEIFNKCKETWPVTRRGNXTSENRSRAFPNPKNVKCILACMLKEAKILRDGEYNKENAELMADVLYKDEPEHAEKSKQIIEMCSAELGTKTEGDDCEFAFQGGQLKIMENSFTLRSLLADCLNRRYPGHVGYRYL

>LlinOBP8

MNPLIPVLLVVCAAATRGDEQTNAMVAKAFNKCHGEFPIGDDEMKGVREKSTVPDSHNAKCLMACMLKEGKILRDGKYEKENAIVMADVLNKDDPAAADKAKQLVETCATQVGSDASADECEFAYKMAVCAAGEAKKLGVRPPDF

>LlinOBP9

MNPLIPVLLVVCAAATRGDEQTNAMVAKAFNKCHGEFPIGDDEMKGVREKSTVPDSHNAKCLMACMLKEGKILRDGKYEKENAIVMADVLNKDDPAAADKAKQLVETCATQVGSDASADECEFAYKMAVCAAAWSSSTRFLKTHPFLLQLCTHRSTWHTFKYEYLPGNTSSRDLTHHINIVCVT

>LlinOBP10

MTPIVAILFALLAAHVKANTKELSPVEVYKHKIHEECIKETKATPEQAKIVFNYKDVPKDDGEKCFMECVYKKSGGIDANGKYSIEGFNKLVDMKYKGEENAGAKMIVKDCSSKVAPKEGEKCSVGRTIRECLSAASKENEFFTI

>LlinOBP11

MHAAFVLIGSALLVAFVSGAPSANVKEIVQNVSKKCVAETKASPEQAKIAVSQHIPKDDVERCYLQCVYTGVGVIKDGKFSEEGGKKLVALRFHDAKEKELANKLIATCAKEIKAKDGEKCSLGRAVRECFVNHGKQVNFFPSA

>LlinOBP12

MVAECPAYSWVCQSTLSYYLHLQQTLQYIDRDPTLAMNQSSCILTLALTIFVMVVVSGFKELDSVLPQAKQEECRKESNFQGELSGDVSQNVTQELKCFAACSLVKLGLMNEKDGTINTTQLDELIAKHTAGKDAADMFKHSVVEPCLKEVNKTADYCEYSFQLVTCGMNKVKPPTTG

>LlinOBP13

MMKIAFVISVLVVLATVSAITPELDKKAKEAVAKCADVPGINEAKKEDCYAACFMTEMGYMTDGKINVENMEEANKQKWDDQQMINKGIEIDKTCAKQVGDTKGKSECAIGYDFGVCKTRLVKANCILRSILQTQLVPSPWWAHLPPLTPLKDSSTFNASFLKGVYREDMEQRNYNKTGLQPPTPLKQ

>LlinOBP14

MKIAFVVSVLVVLATVSAITPELDKKAKEAVAKCADVPEINEAKKEDCYAACFMTEMGYMTDGKINVENMEEANKQKWDDQQMINKGIEIDKTCAKQVGDTKGKSECAIGYDFGVCKTRLVKANCILRSILQXQLVPSPWWAHLPPLTPLKDSSTFNASFLKGVYRGDMEQRNYNKGKNKTGLQPPTPLKQ

>LlinOBP15

MMKIAFVTSVLVVLATVSAITPELDKKAKEAVAKCADVPGINEAKKEDCYAACFMTEMGYMTDGKINVENMEEANRQKWDDQQMINKGIEIDKTCAKQVGDTKGKSECAIGYDFGVCKTRLVKATGLQPPTPLKQ

>LlinOBP16

MKRLVFVLVTLYLLQSASGITDELRKKATEARLKCKQQVGLSDKEYQDWVKGISLPITNGGSCCEVCACWMRELGYMTDGHLNLNNMKNVNTQKWSEKANVEKANQIDTLCTARVVQDGRKECEIALDYRKCKTEMIKQNGGPPKPGST

>LlinOBP17

MKRLVFVLVTLYLLQSASGITDELRKKATETRLKCKQQVGLSDKEYQDWVKGISLPTTNGGSCCEVCACWMRELGYMTDGHLNLNNMKKRQDKYKHS

>LlinOBP18

MRSTGSECFEEVDAKLGNKTSWESDMDPYNCEKVKRMKKRHYCLHECKAKKLGVANEEGVLDFPKVKDLLLSRVNETWQKDILGQAADTCANSKFDQTWKDDTEEYKCNPQAIQFKHCVWKQVEMKCPEEHQNTGRHCKKLRSKISSETSKDSTAKETSV

>LlinOBP19

MKSFVGLIFAVALVEFASAVTKEYHDRAVAAKDKCAKEHNIKESEIQEFVKKHKLPETEDGKCMIACYMEEMKLITDGKVNVDEWKKSNKEKWDEEAHVAMADEIVDKCNEQVSPDGLAKCEYGFKLTECGLKHRLEKGLPAPNMDDVKRR

>LlinOBP20

MKLVKDDKARPPKPEGYECIDDCIMAKNGFLGTDKKIDAAKVNAAAKTSYTGEWAEPGAKMVEKCLAQVSANKEKGECTSGADIFSICMFRESFINCPEKSWTSSETCKANKERLIKCPKSIPFLNKSAK

>LlinOBP21

MLTAYMIVATLSVFFFAVALTQGQMDEDPDCRPPHPPGKEAQCCPLPDFVGVVDNFHDVMHKCSDEAGLRKPSGPPGSGTPPTAEEMAAHMSAHECADECLFKNTKYLQSNGELDKDAIKASVTKIFTGDWAALASSAADKCLASAKSEVGASAKCKSGARQMVKCFTRAMFLNCPASSWTESTECAAAKARITKCPNAMVPMIPPYPQPISANSTILDSLFACITDGEYMEFQSISSGRLG

>LlinOBP22

MPSQLPCPFFLLNIAFAHPGHFDEDPECRQPHHHRHEENDCCKVPSLFSNNKDEMHELVHKCFEEAGIKKHGPHHEHHGPPPLEDGPIPPPPPPPFSPKNDSKLDCVEQCFLKNLDLVDDEGDLKVDDLKALVTEKFSGDWASVGSSAIEKCLEKAKTEENESSKCKAGSKRVLHCHCTQHASYPKMGGSQSARKVTLDNPNPDPHDLNNVI

>LlinOBP23

MYAFTAALSFFLLNIAFAHPGHFDEDPECRQPHHHRHEENDCCKVPSLFSNNKDEMHELVHKCFEEAGIKKHGPHHEHHGPPPLEDGPIPPPPPPPFSPKNDSKFDCVEQCFLKNLDLVDDEGDLKVDDLKALVTEKFSGDWASVGSSAIEKCLEKAKTEENEPSKCKAGSKRVLHCLAREFFMNCPASDWTESEVCLAAKDRVSKCPHSLPPMHH

>LlinOBP24

MADSVGECMKLIKVKPEKGPPVPEGFDCMDTCVFSKLGFIGADNKLDPEKLAKKFSELFKGDWSALSESTLKKCLPMADVGKGVCSSGADVFKFCLIRELYMNCPASSWTKSDLCKANVERLEKCPNSLPFMNGSGLKNKSSR

>LlinOBP25

MFTTATSTIIFLFAVALTRGQMDEDPECRPPHPPGKAGDCCVQPKLFDEGDMPDVIKKCHEEAGVKRPSGPPGSGTPPTAEEMAAHKSAHECAAECIFKNNNFIKSDGELDXDAIKATVTKMFTGDWATLASTTIDKCLASAKSEVDASPKCKSGADQVVRCFGRSLFIGCPASAWTESTECAAEKARLTKCPNAMPPPPHHKH

>LlinOBP26

MNQSSCILTLALTIVAMAVVSGFKELDSVLPQAKQEECRKESNFQGELSGDLSQNVTQELKCFAACSLVKLGLMNEKDGTINTTQLDELIAKHTEGKDAADMFKHSVVEPCLKEVNKTADYCEYSFQLVTCGMNKVKPPTTG

>LlinOBP27

KFYRRFKHHQIAAHLTFCVYVKTGSRDKTETPRSERPLCKAPTSAPRKLEKVINQCQEEIKYALLQEALSVLGETVSLRTALTRNRSKRETFTGEERRIAGCLLQCVYRKMKALDETGFPTATGLVKIYSEGVEDRNYYLATIQGVQQCLSRELQNRNKNPSIVKAEGYSCDVAYDVFNCVSEEIEQLCGTSP

>LlinOBP28

MEVAACLVLLAALAALTSAVDEKRPLCKAPTSAPRKLEKVINQCQEEIKYALLQEALSVLGETVSLRTALTRNRSKRETFTGEERRIAGCLLQCVYRKMKALDETGFPTATGLVKIYSEGVEDRNYYLATIQGVQQCLSRELQNRNKNPSIVKAEGYSCDVAYDVFNCVSEEIEQLCGTSP

>LlinOBP29

MNRPLLLLTAVLAVGSGQQEDCKTAPAGWPKRPPQCCDLPFPLEGMKREFGSCIRQIGNRQSSAVPTAQAVRDARLCIEECVYKGLGFMEEHNLNKDQLLEQLKKGVAGKKDWEKPMEDAVKSCHETITKRETPQEGACQDSAHEFTHCVMRQLFLSCPASEWNNNDECNLVKNRMQACPNIPPPPPPPPQGFRGQGPPQPQ

>LlinOBP30

MNSFTVLCLVASVVALTQGNPTTPNPTSSSHAASVSGGSTVSGVSKSPEEVKQKIKEQVEALTGACKSQTKITGEQAKIVATQAIPKTEAEKCFLECIYTGLQLTKDGKFNEPAARALAQKRFGNAPEDLTKANSMIDTCVKEVVVKDLNEKCALGRLIRECFVKNGAKINFFPKP

>LlinOBP31

MLTAYMIVATLSVFFFAVALTQGQMDEDPDCRPPHPPGKEAQCCPLPDFVGVVDNFHDVMHKCSDEAGLRKPSGPPGSGTPPTAEEMAAHMSAHECADECLFKNTKYLQSNGELDKDAIKASVTKIFTGDWAALASSAADKCLASAKSEVGASAKCKSGARQMVKCFTRAMFLNCPASSWTESTECAAAKARITKCPNAMVPMIPPKH

>LlinOBP32

MEVNAERNVTDEQRXAVRLCSRYTEVESGLAEAGYDCLAECFFIKLGLMGEDKTLNKENILEEVRIQFHEDXVEPARKALETCMEKKYNTKCPSGIDGTMQCFTVQLMLNCPXQNWTDGEECKETRTFMEKCGETLNYYD

>LlinOBP33

LEKVINQCQEEIKYALLQEALSVLGETVSLRTALTRNRSKRETFTGEERRIAGFEFRCDLXDGWKWSQEERGGSSPTWFEINTIPGMNTTSRSWRLSSQISSSSVFCNVYTGK

>NlugOBP1

MKSFIVCIAVSYLLVANIKADEATSSSDAESLITSTTLSPASNESDAARSAIKEQLAKLTESCKTSSQANSDDAKIIGTESVPKTEGEKCFLQCVYTGFGIVKNDQFSVEGARLLAQKRFGAFPEELEKANQLIETCSKEAVKKDSKDKCPMGFLIRQCFVKNGQKINFFPKA

>NlugOBP2

MKCQIVLAALALATICEVSYAGLTPDKLKELKPLIDTCIKQSKVEEDTLGKLHNGHEIPSSQSGKCFIACMAEHMKLMKDGKFEPEMTMEFIDKMVQDKDKAAEIKKSLGECIKSVPEGDKCEMAAGLATCMKDHHAELAGMN

>NlugOBP3

MKASAAITLVFLSLAVFHCSEAKLDKAKKEAAIKKCQAETQATDEDVMKVRKEHIVPDSEEGKCFIACGFNSYDMLKDNRINLEGVNAFFEKLYDEQDKRDIAIKAAASCAATETVSGLNECHYAAKYFACMQRHPDFAKMKDDFDI

>NlugOBP4

MKNYGDKGKIYGKQCYEEVVSAFKTNSSSTADDDDSMMDMFSCEKVKMIKLKHICVHECIGKKTKILKEDGSLNPEEIKQYAREYMFNEEWSKELGEKALDKCLSQTYNSVTKMLDEYEIKCNPSSVQFHHCLWKEIELTCPESKVDLKAKCVRLRERLRKQQAAGM

>NlugOBP5

MERTSVLIVFTFIPFLSSVLGANFLMMQQSMQGTQMPMIQSIASELKFCMDVNAEQNSDGLNDYLPLLFNEELPSTLGQKCFLTCLFNRFGLLKDGFLDTKTAKNLVETFYADKHDEKTMANIAINVCHVAAVPDAMNPCEIGFSLKSCFVDSNKKGKELRGKN

>NlugOBP6

MSTFHKFVISGMVVLAGALFVTAEDTTIKIKNPSPHKHQQVYCQAPPTAPERLERIIEQCQDDIKTALLQEALNVLTDTSPRDLVKKTRSKREVFSGEEKRIAGCLLQCVYRKVKAVDDQGMPTVPGLVRLYSEGVQDRNYYVATVQAVQQCVSASQHFRYYNPQVLKEDGYTCDLAYDMFNCVSDKIEAFCGRTP

>NlugOBP7

MLYLLEFVIFSLALTASIPQIMAADSPDMLAVFNKCREEAGATEDDIKNFRAQQIPSTTTGKCMLACMFNHSGLMKDDKYNSEGALKLVSQVFADNPVNLGKAKQLINGCTDEVKNENDKCEIASKIAHCTVKMTSVVGLS

>NlugOBP8

MLLEVCRFSVFLIALFATVNGRFTEEEKQLMNQVHSQCISETGTSEDLVTKATTGDFADDDNLKCYVKCIWSTLTVMDDEGNFDVGVLEVMLPADMKDTVMKAMNACTGVGGATPCEKAFAMTKCLYKEAPSDFFLP

>NlugOBP9

MPPVFRTAHDKFESCLEELSSIFPPPPPPPHGHHGPPPPPPGARGPPPPPPPGGRRGPPPGFGGPPGHEPPIFACAHECLFNKTGMLENGKLNVEALKKKLEDELGENEVWKNLVQSIVDKCMESKDAPSNEMCTSGSHELARCVLRDMFMNCPQEKWKESDDCSNMKMKLEKCPELVPPMAMRLPHPPMP

>NlugOBP10

MLSESMPVIARVERDASSTTPGFPFKTNQRVALKRAKRSTIFPIDGIVERVAIFPVHEKTKRVVRESSEEDDSCKKMKPEHGAKMCCELPSVFRGSPEIFKACREELGLPDHKSPPPAPSAEGNGKPPHHGGPPHHRGKGCIAECLFNKTGLLEGGKLNKEALQKSLDEHLKTDDAWKAVATSTLDKCYDDVQTKDFKPDNEKFTSGSSEFMRCFSRGLFMDCIPSKWTDSDECSKTKETLEKCPMMLPPG

**CSP sequences**

>ApisCSP1

MNLLAIFCYITMMCDSQFRRLEQPTAIPQVKRIEQPATIATRIGQATIAPKFGQPTIAPRFGQATVAPQIGEAAIGPRIGQSFQSVNNSVSPTTDGRKTTRETSSYPTRYDFIDIEAVMNNERIIKILFNCVMNQGPCTREGLELKRIVPDAIQTECAKCNDRQRKQAGKVLAHLLQYKPEYWNMLVKKFDPNNVYLRKYMADNDDDEKLSLQKLTNNTTK

>ApisCSP2

MAHLNLFVVLVASLVCFTLAEEKYTTKFDNFDVDKVLNNDRILTSYIKCLLDQGNCTNEGRELKKVLPDALKTDCSKCTAVQKDRSEKVIKFLIKNRSKDFDNLTAKYDPSGEYKKKIEKFDAERAAAAKH

>ApisCSP3

MVHLNLFVVVVASLVCFTLAQEKYSTKYENFDEDKVLNNDSLLTSYINCLLDEGNCTEEGQALKRILPDALKTNCGKCTDAQKLKIEKIMKFLIKNRSIDFDRLTAKYDPSGEYKKKLEKFSA

>ApisCSP4

MDSRIAVVCVVLAVFAVDQTVGAPQKDAVAASGTAYTTKYDHIDIDQVLASKRLVNSYVQCLLDKKPCTPEGAELRKILPDALKTQCAKCNATQKNAALKVVDRLQRDYDKEWKQLLDKWDPKREQFQKFQQFLAEEKKKGVVKF

>ApisCSP5

MNCKVLIALCCVAVYAAQANPAGVATATAADDEIKDFPAYMKRFEKLNVEQVLNNDRVLASHLKCFLNEGPCVQQSRDLKRVIPVIANNSCNGCTEKQKTTIKKTLNFLRTKKPDEWARLVKIYDPTGTKLNKFLDA

>ApisCSP6

MNKLLLAVAFFIATTMTMVQAAPAKYTTKYDNVNIDDILNNDRLVASYFKCLMETGKCTPEGEEIKRWLPEAVENKCEDCSEKQKIGSEKIIKFLFEKKNDMWKQLEEKYDPKGLYRQRYSEDAKKLNIDV

>ApisCSP7

MARSSSSVTMKVFVMAVCVCAALARPEEVKMENKPTVVKSETLAAPLPTTIVKRATPQVVSTQKDSSLPNVSEDVLDKALSDRRFVQRQLKCATGEGPCDPIGRKIKAHAPLVLRGMCVKCSQSEIKQIQRVMSHIQKNYPKEYTKMLKQYQSGF

>ApisCSP8

MTNNNMNCPRSRPEIFSLLAVTTIAAVLVHQPAKVYCADGTIYPSQQQQQQTMMFTAPSGYYLSTYDNLDVGHLLRNKKVVSGFVKCFVNEGPCTPDGKLVKAYLLPEIIRTVCGKCTPRQKDMSRAVLRHLYTYRRADFDKIMQIYDTDNKKNEIINFMNQK

>ApisCSP9

MSSFCLNSVILMTVTTVMVALVTFTGCTASDDRPGMSDIRLAKRDADHKENNADSEEGFFFSLTNFFGRSKHDEDDDKPDFITTFDLIRLLDEKYAMKQFYCVINEDPCDSVGMRLKATIPEEINRDCERCTATETNNIRRILNYVKKHYPKFWERVEPIYRDKMTVLTANIVKIQ

>ApisCSP10

MVSKPFVFVFVLMSVVGVSFSVTEENDGTKVVDKEEDHHQNIQEELKKFLSTLEKIDIDQILNNHRLMSNNVKCFLNEGPCTAQLREMKKMLPALVKDSCASCTKEQKNIIKKSMEAIQARRPNEYKQVSKFFDPEGKYQKKFLENLNTD

>ApisCSP11_P

MFKLVFVAIISGFVSMVQCGPMPEQRIDGGQNRRQEQQKYAVDHFTTGLVGNPKIRENYLNCFLDNGPCSPEAKNIKPGMVPEAIQNECEHCTELQRKVIEKMMCYLNNHQPDILKEVAAKFDPNGEYMKQYINTIERNGNEQF

>ApisCSP12_N

VIPDALKTDCSKCTDVQVSKLLKIMKFLMKNRSADFDRLTTNYDPSGEYKKKL

>ApisCSP13_N

MMFTAPSGYYLSTYDNLDVGHLLRNKKVVSGFVKCFVNEGPCTPDGKLVKDTIWEGAWRCLSAPILNLAPPNGNGNGV

>AgosCSP1

MNILTIFCYVTVMCDTQVKPAVSAQRLQSVNQNVTPTNDGRKTIRETSSYPTRYDYIDIEAVMNNERIIKILFNCVMSRGPCTREGLELKRIVPDAIQTECAKCNERQRKQAGKVLAHLLQYKPEYWKMLVQKFDPNNVYLRKYMADNDDDEKLSLQKLSNDTTKKKRNI

>AgosCSP2

MAHLNLFVVLIASLIYFTSAAEEKYTTKFDNFDVDKVLNNNRILTSYIKCLLDEGNCTNEGRELKRVLPDALKTDCSKCTDVQKDRSEKVIKFLIKNRSTDFDRLTAKYDPTGEYKKNLEKFEKERASAKPLKA

>AgosCSP4

MDSRIAVVCVVLAAFAVDQTVGAPQKDAVAASGPAYTTKYDHIDVDQVLASKRLVNSYVQCLLDKKPCTPEGAELRKILPDALKTQCAKCNATQKNAALKVVDRLQKDYDAEWKQLLDKWDPKREHFQKFQQFLAEEKKKGFTKF

>AgosCSP5

MHCKVLIALCCVAVYAVQASPAGTATAAAVSADDEIKDFPAYMKRFDKLNVEQVLNNDRVLASHLKCFLNEGPCVQQSRDLKRVIPVIANNGCNGCTERQMTTIKKSLNFLRTKKPTEWARLVKIYDPSGTKLNKFLDA

>AgosCSP6

MIKLILAIAFCVSITMTVVQTAPAKYTTKYDNVNIDEILNNDRLVASYFKCLMETGKCTPEGEEIKRWLPEAIENKCEDCSEKQKLGSEKIIKFLFEKKNDMWKQLEAKYDPQGTYRQRYAEEAKKLNINV

>AgosCSP7

MSRSSSSVTMKVFVIAICVCAALARPEDVKVENKPAVIKSETLAAPLPTNIVKRATDTIQLDSSLPNVSEDVLDKALSDRRFVQRQLKCATGEGPCDPIGRKIKAHAPLVLRGMCVKCSQSEIKQIQRVMSHIQKNYPKEYTKMLKQYQSGF

>AgosCSP8

MNNIIMNNSRGRYGIFSLLAVTIAAIMLVHQPATVRCADGGIITPQQQQQQTMMFTAPTGYYVSTYDHIDVGRLLRNNKVVSGYVKCFVNEGPCTPDGKLVKAYLLPEIIRTVCGKCTPRQKDMARMVLKHIYTYRQADFEKIMQIYDTDGKRNEILAFMNH

>AgosCSP9

MSAFCLNSFILMTMITVIVTHATFTRSTKFDDRTGIDIHLVKRDTDDVNDDENSVESDEGFFYRFTHFFQDSSDKEDDDDDEKKPDFITTFDIFKLLDEEYAMQQFYCVINEDPCDEVGMRLKATIPEEINRNCERCTSTERNNIRRILNYVKKHYPQFWKRVEPIYKKKI

>AgosCSP10

MINTRPRKLVRCIRGVSISVAKGDDAVNAENKDDDSHLVNREEIQRYMSMMEKINIDQMLNNTRLMSNNVKCFLNEGPCTAHLREMKKMVPMLVKDSCSSCTKEQKIMMKKAMDAVKARRPNDYEKLSKFFDPEGKYEKKFLENLNESK

>NlugCSP1

MFKNVLLVCLLVAVVSAKPKPAEKKQYTTKYDNIDLDEILNNQRLFDNYYKCLLGGKCTPDGQELREALPDALATACSKCTEKQRVGTEKVIKYLIEKKPTEYSELEKKYDPQGNYKRKYQAEAAKRGIKV

>NlugCSP2

MSKLPVTLVLMLAVFSVDCGKLYKDRYTTKFDKIDLDEALNNQRLFESYLKCLMGDKCSPDGYELREALPDALATACAKCSEAQKAGTEKVIRFLIEKRPKEYALLEKKYDPEGIYRDKYKPIAEMKGIKLD

>NlugCSP3

MKFLCVTIFECALIVVAFGMPQDTTYPTTYDDVNVDDILHNDRLFNRYFTCLTKKEGCTPEGKLLAATIPDALATTCAKCSAKQKTAAEKVIKYLYFNKRDKFDELAKIYDPESNYLNKYLVDGFPAKV

>NlugCSP4

MFLIAVWALSPRRLPWGLPWGGLAGVAAQQQAKNTRYTTRFDSIDVEVILKNERIFRRYMDCLLDKGRCTPEARELKRLLPEALKTECLKCSEVQRRQGAKVMAFIIKNKRPSWELLLAKYDPQGIFRAKYMYNENNIEAVLKQLEREQQGIYGTYSSTNSTTSSNSTSIR

>NlugCSP5

MRCLLLVAVVCAALVAVCHAQDSKYTSKYDNIDIDKILKNDRVLSQYIKCLMGEGSCTQEGRELKRLLPDAIQSNCSKCSEKQRSASVKVMRHLRQSRERDWNRLLDKYDPQGDKRKNLKLD

>NlugCSP6

MLWAARFIVLPLLFCVLQVWSAPADEKYTDIDFDSILANRRVLSSYVKCLTDKGPCTPQGKELKKIVPEVIQTSCTKCSPQQKKVVRNVITTMQSKYKDQWDLVVNKYDPKKQRSGELKAFLSGTD

>NlugCSP7

MASASSGTTSTTSAPKTAESASAKSSSKDEIPDQTFDRYINNERYMLMQYECLMGNKPCDHVGRKLKAAVPLVVRGLGCPKCSQREEDQMKRIVSHVQRSYPDKWQKLIKKYGN

>NlugCSP8

MSSTMLVFVAVLCFSAVLAKPADKYTTKYDNIDLDEVLSNQRLFDSYFKCLMGGKCTPDGQELRDALPDALATACEKCSEKQKEGTEKVMKFLIEKKPTEFAELEKKYDPQGTYRQKYKAEADKRGYSV

>NlugCSP9

MKSQQLLVSCLFICTWLVVLMAPSANAAPKEKDPERKALYRLEYIDIEKVLDNNRMLTNFIRCFLRQGPCTPEARDFRKLLPKLAKTMCSDCTARQRYIIKKVFKHLMEERPKEWELLMDRFDPQRKYAERLDTFMVDMTTRAPVTSSPMPSSPVTLTSSSVTMSSTTQRVIEILRTSTDMSNESRPAS

>NlugCSP10

MSEVLVTSLLFMLLACSELGSGQQQQNVDNIEMSIYDKMFENMDVNSLLKNHRLVDSYLKCFLNEGSCTHIGHEVKMMIPEVIKSRCGTCGENQMRALKAGLRLFIVLRPDDWQRFLDVYDPDRKEWPHIKAFMDSDD

>NlugCSP11

MKSIILLVFVSMSAMVYRCRADEPSYPTSWDNVNIDEVLGNERLVQNYAKCLLEKGSCSPEGTELKKAIPDALKTGCTKCSDKQKAGAQKVIKWLVQKKPELWKEVVDKYDPSGEYTKKYEKEYQI

>AlinCSP1

MLKVLVLLAAVVCCVSAAATYTSKYDNIDLDEILSNTRLYKKYFDCLANKGKCTPDGKELKESLPDALKTNCAKCTKKQQEGTDKVFRHVLKNKPNDYKVLESIYDPPGIYRKKYEAEAEKRGIKLPGSH

>AlinCSP2

MKVAVLVLLCVGAALSAEVYTSKYDNIDVDKILSNDRILTRYIKCLMEEGNCTNEGKELKKTLPDALASGCTKCSEKQKAQTEKVLRHLSKNRPRDWALLKTKYDPKGEYSKKYEKEAKALTA

>AlinCSP3

MISKLSMVLLIGAFADVWAAEQYTDKYDNIDIDEILNNDRMYKNYFHCVMGNGKCTPDGLELKAKIPEALQTECAKCTDKQKKEVEKVLRFIINQKKDDYKLLEEKFDPEGVYRKKYEAQKKLVEEGKPIEY

**OR sequences**

>DmelOR83bIa

MTTSMQPSKYTGLVADLMPNIRAMKYSGLFMHNFTGGSAFMKKVYSSVHLVFLLMQFTFILVNMALNAEEVNELSGNTITTLFFTHCITKFIYLAVNQKNFYRTLNIWNQVNTHPLFAESDARYHSIALAKMRKLFFLVMLTTVASATAWTTITFFGDSVKMVVDHETNSSIPVEIPRLPIKSFYPWNASHGMFYMISFAFQIYYVLFSMIHSNLCDVMFCSWLIFACEQLQHLKGIMKPLMELSASLDTYRPNSAALFRSLSANSKSELIHNEEKDPGTDMDMSGIYSSKADWGAQFRAPSTLQSFGGNGGGGNGLVNGANPNGLTKKQEMMVRSAIKYWVERHKHVVRLVAAIGDTYGAALLLHMLTSTIKLTLLAYQATKINGVNVYAFTVVGYLGYALAQVFHFCIFGNRLIEESSSVMEAAYSCHWYDGSEEAKTFVQIVCQQCQKAMSISGAKFFTVSLDLFASVLGAVVTYFMVLVQLK

>DmelOR83bIb

MTTSMQPSKYTGLVADLMPNIRAMKYSGLFMHNFTGGSAFMKKVYSSVHLVFLLMQFTFILVNMALNAEEVNELSGNTITTLFFTHCITKFIYLAVNQKNFYRTLNIWNQVNTHPLFAESDARYHSIALAKMRKLFFLVMLTTVASATAWTTITFFGDSVKMVVDHETNSSIPVEIPRLPIKSFYPWNASHGMFYMISFAFQIYYVLFSMIHSNLCDVMFCSWLIFACEQLQHLKGIMKPLMELSASLDTYRPNSAALFRSLSANSKSELIHNEEKDPGTDMDMSGIYSSKADWGAQFRAPSTLQSFGGNGGGGNGLVNGANPNGLTKKQEMMVRSAIKYWVERHKHVVRLVAAIGDTYGAALLLHMLTSTIKLTLLAYQATKINGVNVYAFTVVGYLGYALAQVFHFCIFGNRLIEESSSVMEAAYSCHWYDGSEEAKTFVQIVCQQCQKAMSISGAKFFTVSLDLFASVLGAVVTYFMVLVQLK

>DmelOR69aIa

MQLHDHMKYIDLGCKMACIPRYQWKGRPTERQFYASEQRIVFLLGTICQIFQITGVLIYWYCNGRLATETGTFVAQLSEMCSSFCLTFVGFCNVYAISTNRNQIETLLEELHQIYPRYRKNHYRCQHYFDMAMTIMRIEFLFYMILYVYYNSAPLWVLLWEHLHEEYDLSFKTQTNTWFPWKVHGSALGFGMAVLSITVGSFVGVGFSIVTQNLICLLTFQLKLHYDGISSQLVSLDCRRPGAHKELSILIAHHSRILQLGDQVNDIMNFVFGSSLVGATIAICMSSVSIMLLDLASAFKYASGLVAFVLYNFVICYMGTEVTLASGKVLPAAFYNNWYEGDLVYRRMLLILMMRATKPYMWKTYKLAPVSITTYMATLKFSYQMFTCVRSLK

>DmelOR69aIb

MQLEDFMRYPDLVCQAAQLPRYTWNGRRSLEVKRNLAKRIIFWLGAVNLVYHNIGCVMYGYFGDGRTKDPIAYLAELASVASMLGFTIVGTLNLWKMLSLKTHFENLLNEFEELFQLIKHRAYRIHHYQEKYTRHIRNTFIFHTSAVVYYNSLPILLMIREHFSNSQQLGYRIQSNTWYPWQVQGSIPGFFAAVACQIFSCQTNMCVNMFIQFLINFFGIQLEIHFDGLARQLETIDARNPHAKDQLKYLIVYHTKLLNLADRVNRSFNFTFLISLSVSMISNCFLAFSMTMFDFGTSLKHLLGLLLFITYNFSMCRSGTHLILTSGKVLPAAFYNNWYEGDLVYRRMLLILMMRATKPYMWKTYKLAPVSITTYMATLKFSYQMFTCVRSLK

>DmelOR46aIa

MSKGVEIFYKGQKAFLNILSLWPQIERRWRIIHQVNYVHVIVFWVLLFDLLLVLHVMANLSYMSEVVKAIFILATSAGHTTKLLSIKANNVQMEELFRRLDNEEFRPRGANEELIFAAACERSRKLRDFYGALSFAALSMILIPQFALDWSHLPLKTYNPLGENTGSPAYWLLYCYQCLALSVSCITNIGFDSLCSSLFIFLKCQLDILAVRLDKIGRLITTSGGTVEQQLKENIRYHMTIVELSKTVERLLCKPISVQIFCSVLVLTANFYAIAVLSDERLELFKYVTYQACMLIQIFILCYYAGEVTQRSLDLPHELYKTSWVDWDYRSRRIALLFMQRLHSTLRIRTLNPSLGFDLMLFSSIVNCSYSYFALLKRVNS

>DmelOR46aIb

MVTEDFYKYQVWYFQILGVWQLPTWAADHQRRFQSMRFGFILVILFIMLLLFSFEMLNNISQVREILKVFFMFATEISCMAKLLHLKLKSRKLAGLVDAMLSPEFGVKSEQEMQMLELDRVAVVRMRNSYGIMSLGAASLILIVPCFDNFGELPLAMLEVCSIEGWICYWSQYLFHSICLLPTCVLNITYDSVAYSLLCFLKVQLQMLVLRLEKLGPVIEPQDNEKIAMELRECAAYYNRIVRFKDLVELFIKGPGSVQLMCSVLVLVSNLYDMSTMSIANGDAIFMLKTCIYQLVMLWQIFIICYASNEVTVQSSRLCHSIYSSQWTGWNRANRRIVLLMMQRFNSPMLLSTFNPTFAFSLEAFGSIVNCSYSYFALLKRVNS

>DmelOR63aIa

MYSPEEAAELKRRNYRSIREMIRLSYTVGFNLLDPSRCGQVLRIWTIVLSVSSLASLYGHWQMLARYIHDIPRIGETAGTALQFLTSIAKMWYFLFAHRQIYELLRKARCHELLQKCELFERMSDLPVIKEIRQQVESTMNRYWASTRRQILIYLYSCICITTNYFINSFVINLYRYFTKPKGSYDIMLPLPSLYPAWEHKGLEFPYYHIQMYLETCSLYICGMCAVSFDGVFIVLCLHSVGLMRSLNQMVEQATSELVPPDRRVEYLRCCIYQYQRVANFATEVNNCFRHITFTQFLLSLFNWGLALFQMSVGLGNNSSITMIRMTMYLVAAGYQIVVYCYNGQRFATASEEIANAFYQVRWYGESREFRHLIRMMLMRTNRGFRLDVSWFMQMSLPTLMAMVRTSGQYFLLLQNVNQK

>DmelOR63aIb

MYSPEEAAELKRRNYRSIREMIRLSYTVGFNLLDPSRCGQVLRIWTIVLSVSSLASLYGHWQMLARYIHDIPRIGETAGTALQFLTSIAKMWYFLFAHRQIYELLRKARCHELLQKCELFERMSDLPVIKEIRQQVESTMNRYWASTRRQILIYLYSCICITTNYFINSFVINLYRYFTKPKGSYDIMLRK

>DmelOR43b

MFGHFKLVYPAPISEPIQSRDSNAYMMETLRNSGLNLKNDFGIGRKIWRVFSFTYNMVILPVSFPINYVIHLAEFPPELLLQSLQLCLNTWCFALKFFTLIVYTHRLELANKHFDELDKYCVKPAEKRKVRDMVATITRLYLTFVVVYVLYATSTLLDGLLHHRVPYNTYYPFINWRVDRTQMYIQSFLEYFTVGYAIYVATATDSYPVIYVAALRTHILLLKDRIIYLGDPSNEGSSDPSYMFKSLVDCIKAHRTMLNFCDAIQPIISGTIFAQFIICGSILGIIMINMVLFADQSTRFGIVIYVMAVLLQTFPLCFYCNAIVDDCKELAHALFHSAWWVQDKRYQRTVIQFLQKLQQPMTFTAMNIFNINLATNINVAKFAFTVYAIASGMNLDQKLSIKE

>DmelOR23a

MKLSETLKIDYFRVQLNAWRICGALDLSEGRYWSWSMLLCILVYLPTPMLLRGVYSFEDPVENNFSLSLTVTSLSNLMKFCMYVAQLTKMVEVQSLIGQLDARVSGESQSERHRNMTEHLLRMSKLFQITYAVVFIIAAVPFVFETELSLPMPMWFPFDWKNSMVAYIGALVFQEIGYVFQIMQCFAADSFPPLVLYLISEQCQLLILRISEIGYGYKTLEENEQDLVNCIRDQNALYRLLDVTKSLVSYPMMVQFMVIGINIAITLFVLIFYVETLYDRIYYLCFLLGITVQTYPLCYYGTMVQESFAELHYAVFCSNWVDQSASYRGHMLILAERTKRMQLLLAGNLVPIHLSTYVACWKGAYSFFTLMADRDGLGS

>DmelOR22b

MLSQFFPHIKEKPLSERVKSRDAFVYLDRVMWSFGWTVPENKRWDLHYKLWSTFVTLLIFILLPISVSVEYIQRFKTFSAGEFLSSIQIGVNMYGSSFKSYLTMMGYKKRQEAKMSLDELDKRCVCDEERTIVHRHVALGNFCYIFYHIAYTSFLISNFLSFIMKRIHAWRMYFPYVDPEKQFYISSIAEVILRGWAVFMDLCTDVCPLISMVIARCHITLLKQRLRNLRSEPGRTEDEYLKELADCVRDHRLILDYVDALRSVFSGTIFVQFLLIGIVLGLSMINIMFFSTLSTGVAVVLFMSCVSMQTFPFCYLCNMIMDDCQEMADSLFQSDWTSADRRYKSTLVYFLHNLQQPIILTAGGVFPISMQTNLNMVKLAFTVVTIVKQFNLAEKFQ

>DmelOR22a

MLSKFFPHIKEKPLSERVKSRDAFIYLDRVMWSFGWTEPENKRWILPYKLWLAFVNIVMLILLPISISIEYLHRFKTFSAGEFLSSLEIGVNMYGSSFKCAFTLIGFKKRQEAKVLLDQLDKRCLSDKERSTVHRYVAMGNFFDILYHIFYSTFVVMNFPYFLLERRHAWRMYFPYIDSDEQFYISSIAECFLMTEAIYMDLCTDVCPLISMLMARCHISLLKQRLRNLRSKPGRTEDEYLEELTECIRDHRLLLDYVDALRPVFSGTIFVQFLLIGTVLGLSMINLMFFSTFWTGVATCLFMFDVSMETFPFCYLCNMIIDDCQEMSNCLFQSDWTSADRRYKSTLVYFLHNLQQPITLTAGGVFPISMQTNLAMVKLAFSVVTVIKQFNLAERFQ

>DmelOR82a

MGRLFQLQEYCLRAMGHKDDMDSTDSTALSLKHISSLIFVISAQYPLISYVAYNRNDMEKVTACLSVVFTNMLTVIKISTFLANRKDFWEMIHRFRKMHEQSASHIPRYREGLDYVAEANKLASFLGRAYCVSCGLTGLYFMLGPIVKIGVCRWHGTTCDKELPMPMKFPFNDLESPGYEVCFLYTVLVTVVVVAYASAVDGLFISFAINLRAHFQTLQRQIENWEFPSSEPDTQIRLKSIVEYHVLLLSLSRKLRSIYTPTVMGQFVITSLQVGVIIYQLVTNMDSVMDLLLYASFFGSIMLQLFIYCYGGEIIKAESLQVDTAVRLSNWHLASPKTRTSLSLIILQSQKEVLIRAGFFVASLANFVGICRTALSLITLIKSIE

>DmelOR43a

MTIEDIGLVGINVRMWRHLAVLYPTPGSSWRKFAFVLPVTAMNLMQFVYLLRMWGDLPAFILNMFFFSAIFNALMRTWLVIIKRRQFEEFLGQLATLFHSILDSTDEWGRGILRRAEREARNLAILNLSASFLDIVGALVSPLFREERAHPFGLALPGVSMTSSPVYEVIYLAQLPTPLLLSMMYMPFVSLFAGLAIFGKAMLQILVHRLGQIGGEEQSEEERFQRLASCIAYHTQVMRYVWQLNKLVANIVAVEAIIFGSIICSLLFCLNIITSPTQVISIVMYILTMLYVLFTYYNRANEICLENNRVAEAVYNVPWYEAGTRFRKTLLIFLMQTQHPMEIRVGNVYPMTLAMFQSLLN ASYSYFTMLRGVTG

>DmelOR2a

MEKQEDFKLNTHSAVYYHWRVWELTGLMRPPGVSSLLYVVYSITVNLVVTVLFPLSLLARLLFTTNMAGLCENLTITITDIVANLKFANVYMVRKQLHEIRSLLRLMDARARLVGDPEEISALRKEVNIAQGTFRTFASIFVFGTTLSCVRVVVRPDRELLYPAWFGVDWMHSTRNYVLINIYQLFGLIVQAIQNCASDSYPPAFLCLLTGHMRALELRVRRIGCRTEKSNKGQTYEAWREEVYQELIECIRDLARVHRLREIIQRVLSVPCMAQFVCSAAVQCTVAMHFLYVADDHDHTAMIISIVFFSAVTLEVFVICYFGDRMRTQSEALCDAFYDCNWIEQLPKFKRELLFTLARTQRPSLIYAGNYIALSLETFEQVMRFTYSVFTLLLRAK

>DmelOR67d

MLKMAKVEPVERYCKVIRMIRFCVGFCGNDVADPNFRMWWLTYAVMAAIAFFFACTGYTIYVGVVINGDLTIILQALAMVGSAVQGLTKLLVTANNASHMREVQNTYEDIYREYGSKGDEYAKCLEKRIRITWTLLIGFMLVYIILLGLVITFPIFYLLILHQKVLVMQFLIPFLDHTTDGGHLILTAAHVILITFGGFGNYGGDMYLFLFVTHVPLIKDIFCVKLTEFNELVMKRNDFPKVRAMLCDLLVWHQLYTRMLQTTKKIYSIVLFVQLSTTCVGLLCTISCIFMKAWPAAPLYLLYAAITLYTFCGLGTLVENSNEDFLSVIYTNCLWYELPVKEEKLIIMMLAKAQNEVVLTAADMAPLSMNTALQLTKGIYSFSMMLMNYLG

>DmelOR59c

MTKFFFKRLQTAPLDQEVSSLDASDYYYRIAFFLGWTPPKGALLRWIYSLWTLTTMWLGIVYLPLGLSLTYVKHFDRFTPTEFLTSLQVDINCIGNVIKSCVTYSQMWRFRRMNELISSLDKRCVTTTQRRIFHKMVARVNLIVILFLSTYLGFCFLTLFTSVFAGKAPWQLYNPLVDWRKGHWQLWIASILEYCVVSIGTMQELMSDTYAIVFISLFRCHLAILRDRIANLRQDPKLSEMEHYEQMVACIQDHRTIIQCSQIIRPILSITIFAQFMLVGIDLGLAAISILFFPNTIWTIMANVSFIVAICTESFPCCMLCEHLIEDSVHVSNALFHSNWITADRSYKSAVLYFLHRAQQPIQFTAGSIFPISVQSNIAVAKFAFTIITIVNQMNLGEKFFSDRSNGDINP

>DmelOR59b

MAVFKLIKPAPLTEKVQSRQGNIYLYRAMWLIGWIPPKEGVLRYVYLFWTCVPFAFGVFYLPVGFIISYVQEFKNFTPGEFLTSLQVCINVYGASVKSTITYLFLWRLRKTEILLDSLDKRLANDSDRERIHNMVARCNYAFLIYSFIYCGYAGSTFLSYALSGRPPWSVYNPFIDWRDGMGSLWIQAIFEYITMSFAVLQDQLSDTYPLMFTIMFRAHMEVLKDHVRSLRMDPERSEADNYQDLVNCVLDHKTILKCCDMIRPMISRTIFVQFALIGSVLGLTLVNVFFFSNFWKGVASLLFVITILLQTFPFCYTCNMLIDDAQDLSNEIFQSNWVDAEPRYKATLVLFMHHVQQPIIFIAGGIFPISMNSNITVAKFAFSIITIVRQMNLAEQFQ

>DmelOR59a

MAEVRVDSLEFFKSHWTAWRYLGVAHFRVENWKNLYVFYSIVSNLLVTLCYPVHLGISLFRNRTITEDILNLTTFATCTACSVKCLLYAYNIKDVLEMERLLRLLDERVVGPEQRSIYGQVRVQLRNVLYVFIGIYMPCALFAELSFLFKEERGLMYPAWFPFDWLHSTRNYYIANAYQIVGISFQLLQNYVSDCFPAVVLCLISSHIKMLYNRFEEVGLDPARDAEKDLEACITDHKHILELFRRIEAFISLPMLIQFTVTALNVCIGLAALVFFVSEPMARMYFIFYSLAMPLQIFPSCFFGTDNEYWFGRLHYAAFSCNWHTQNRSFKRKMMLFVEQSLKKSTAVAGGMMRIHLDTFFSTLKGAYSLFTIIIRMRK

>DmelOR88a

MKPTEIKKPYRMEEFLRPQMFQEVAQMVHFQWRRNPVDNSMVNASMVPFCLSAFLNVLFFGCNGWDIIGHFWLGHPANQNPPVLSITIYFSIRGLMLYLKRKEIVEFVNDLDRECPRDLVSQLDMQMDETYRNFWQRYRFIRIYSHLGGPMFCVVPLALFLLTHEGKDTPVAQHEQLLGGWLPCGVRKDPNFYLLVWSFDLMCTTCGVSFFVTFDNLFNVMQGHLVMHLGHLARQFSAIDPRQSLTDEKRFFVDLRLLVQRQQLLNGLCRKYNDIFKVAFLVSNFVGAGSLCFYLFMLSETSDVLIIAQYILPTLVLVGFTFEICLRGTQLEKASEGLESSLRSQEWYLGSRRYRKFYLLWTQYCQRTQQLGAFGLIQVNMVHFTEIMQLAYRLFTFLKSH

>DmelOR22c

MTDSGQPAIADHFYRIPRISGLIVGLWPQRIRGGGGRPWHAHLLFVFAFAMVVVGAVGEVSYGCVHLDNLVVALEAFCPGTTKAVCVLKLWVFFRSNRRWAELVQRLRAILWESRRQEAQRMLVGLATTANRLSLLLLSSGTATNAAFTLQPLIMGLYRWIVQLPGQTELPFNIILPSFAVQPGVFPLTYVLLTASGACTVFAFSFVDGFFICSCLYICGAFRLVQQDIRRIFADLHGDSVDVFTEEMNAEVRHRLAQVVERHNAIIDFCTDLTRQFTVIVLMHFLSAAFVLCSTILDIMLNTSSLSGLTYICYIIAALTQLFLYCFGGNHVSESSAAVADVLYDMEWYKCDARTRKVILMILRRSQRAKTIAVPFFTPSLPALRSILSTAGSYITLLKTFL

>DmelOR56a

MFKDLLLSPTTFEDPIFGTHLRYFQWYGYVASKDQNRPLLSLIRCTILTASIWLSCALMLARVFRGYENLNDGATSYATAVQYFAVSIAMFNAYVQRDKVISLLRVAHSDIQNLMHEADNREMELLVATQAYTRTITLLIWIPSVIAGLMAYSDCIYRSLFLPKSVFNVPAVRRGEEHPILLFQLFPFGELCDNFVVGYLGPWYALGLGITAIPLWHTFITCLMKYVNLKLQILNKRVEEMDITRLNSKLVIGRLTASELTFWQMQLFKEFVKEQLRIRKFVQELQYLICVPVMADFIIFSVLICFLFFALTVGVPSKMDYFFMFIYLFVMAGILWIYHWHATLIVECHDELSLAYFSCGWYNFEMPLQKMLVFMMMHAQRPMKMRALLVDLNLRTFIDIGRGAYSYFNLLRSSHLY

>DmelOR85f

MEPVQYSYEDFARLPTTVFWIMGYDMLGVPKTRSRRILYWIYRFLCLASHGVCVGVMVFRMVEAKTIDNVSLIMRYATLVTYIINSDTKFATVLQRSAIQSLNSKLAELYPKTTLDRIYHRVNDHYWTKSFVYLVIIYIGSSIMVVIGPIITSIIAYFTHNVFTYMHCYPYFLYDPEKDPVWIYISIYALEWLHSTQMVISNIGADIWLLYFQVQINLHFRGIIRSLADHKPSVKHDQEDRKFIAKIVDKQVHLVSLQNDLNGIFGKSLLLSLLTTAAVICTVAVYTLIQGPTLEGFTYVIFIGTSVMQVYLVCYYGQQVLDLSGEVAHAVYNHDFHDASIAYKRYLLIIIIRAQQPVELNAMGYLSISLDTFKQLMSVSYRVITMLMQMIQ

>DmelOR85d

MLTKKDTQSAKEQEKLKAIPLHSFLKYANVFYLSIGMMAYDHKYSQKWKEVLLHWTFIAQMVNLNTVLISELIYVFLAIGKGSNFLEATMNLSFIGFVIVGDFKIWNISRQRKRLTQVVSRLEELHPQGLAQQEPYNIGHHLSGYSRYSKFYFGMHMVLIWTYNLYWAVYYLVCDFWLGMRQFERMLPYYCWVPWDWSTGYSYYFMYISQNIGGQACLSGQLAADMLMCALVTLVVMHFIRLSAHIESHVAGIGSFQHDLEFLQATVAYHQSLIHLCQDINEIFGVSLLSNFVSSSFIICFVGFQMTIGSKIDNLVMLVLFLFCAMVQVFMIATHAQRLVDASEQIGQAVYNHDWFRADLRYRKMLILIIKRAQQPSRLKATMFLNISLVTVSDLLQLSYKFFALLRTMYVN

>DmelOR85c

MKFMKYAVFFYTSVGIEPYTIDSRSKKASLWSHLLFWANVINLSVIVFGEILYLGVAYSDGKFIDAVTVLSYIGFVIVGMSKMFFIWWKKTDLSDLVKELEHIYPNGKAEEEMYRLDRYLRSCSRISITYALLYSVLIWTFNLFSIMQFLVYEKLLKIRVVGQTLPYLMYFPWNWHENWTYYVLLFCQNFAGHTSASGQISTDLLLCAVATQVVMHFDYLARVVEKQVLDRDWSENSRFLAKTVQYHQRILRLMDVLNDIFGIPLLLNFMVSTFVICFVGFQMTVGVPPDIMIKLFLFLFSSLSQVYLICHYGQLIADASSSLSISAYKQNW QNADIRYRRALVFFIARPQRTTYLKATIFMNITRATMTDLLQVSYKFFALLRTMYIK

>DmelOR85b

MEKLMKYASFFYTAVGIRPYTNGEESKMNKLIFHIVFWSNVINLSFVGLFESIYVYSAFMDNKFLEAVTALSYIGFVTVGMSKMFFIRWKKTAITELINELKEIYPNGLIREERYNLPMYLGTCSRISLIYSLLYSVLIWTFNLFCVMEYWVYDKWLNIRVVGKQLPYLMYIPWKWQDNWSYYPLLFSQNFAGYTSAAGQISTDVLLCAVATQLVMHFDFLSNSMERHELSGDWKKDSRFLVDIVRYHERILRLSDAVNDIFGIPLLLNFMVSSFVICFVGFQMTVGVPPDIVVKLFLFLVSSMSQVYLICHYGQLVADASYGFSVATYNQKWYKADVRYKRALVIIIARSQKVTFLKATIFLDITRSTMTDLLQISYKFFALLRTMYTQ

>DmelOR85a

MIFKYIQEPVLGSLFRSRDSLIYLNRSIDQMGWRLPPRTKPYWWLYYIWTLVVIVLVFIFIPYGLIMTGIKEFKNFTTTDLFTYVQVPVNTNASIMKGIIVLFMRRRFSRAQKMMDAMDIRCTKMEEKVQVHRAAALCNRVVVIYHCIYFGYLSMALTGALVIGKTPFCLYNPLVNPDDHFYLATAIESVTMAGIILANLILDVYPIIYVVVLRIHMELLSERIKTLRTDVEKGDDQHYAELVECVKDHKLIVEYGNTLRPMISATMFIQLLSVGLLLGLAAVSMQFYNTVMERVVSGVYTIAILSQTFPFCYVCEQLSSDCESLTNTLFHSKWIGAERRYRTTMLYFIHNVQQSILFTAGGIFPICLNTNIKMAKFAFSVVTIVNEMDLAEKLRRE

>DmelOR13a

MFYSYPYKALSFPIQCVWLKLNGSWPLTESSRPWRSQSLLATAYIVWAWYVIASVGITISYQTAFLLNNLSDIIITTENCCTTFMGVLNFVRLIHLRLNQRKFRQLIENFSYEIWIPNSSKNNVAAECRRRMVTFSIMTSLLACLIIMYCVLPLVEIFFGPAFDAQNKPFPYKMIFPYDAQSSWIRYVMTYIFTSYAGICVVTTLFAEDTILGFFITYTCGQFHLLHQRIAGLFAGSNAELAESIQLERLKRIVEKHNNIISFAKRLEDFFNPILLANLMISSVLICMVGFQIVTGKNMFIGDYVKFIIYISSALSQLYVLCENGDALIKQSTLTAQILYECQWEGSDRIEIQSFTPTTKRIRNQIWFMILCSQQPVRITAFKFSTLSLQSFTAILSTSISYFTLLRSVYFDDEKKLD

>DmelOR65c

MDIRGNVHRFVKFYIDGWKHFRDPTMESSYSAVYYWREQMKAMFLYTTSKERQMPYRSSWHTLVIIQATVCFLTMCYGVTESLGDKVQMGRDIAFIIGFFYIAFKIYYFQWYGDELDEVVEALETFHPWAQKGPGAVDYRTAKRWYFTLAFFLASSWLVFLCIFILLLITSPLWVHQQILPLHAAFPFQWHEKSIHPISHAFIYLFQTWNVMYFLTWLVCIEGLSVSIYVEITFAIEVLCLELRHLHQRCHGYEQLRLETNRLVQFHQKIVHILDHTNKVFHGTLIMQMGVNFFLVSLSVLEAMEARKDPKVVAQFAVLMLLALGHLSMWSYFGDLLSQKSLTISEAAYEAYDPIKGSKDVYRDLCLIIRRGQEPLIMRASPFPSFNFINYSAILNQCYGILTFLLKTLD

>DmelOR65b

MDIQRFLKFYKVGWKTYRDPLMEASHSSIYYWREQMKAMALFTTTEERLLPYRSKWHTLVYIQMVIFFASMSFGLTESMGDHVQMGRDLAFILGAFFIIFKTYYFCWYGDELDQVISDLDALHPWAQKGPNPVEYQTGKRWYFVMAFFLATSWSFFLCILLLLLITSPMWVHQQNLPFHAAFPFQWHEKSLHPISHAIIYLFQSYFAVYCLTWLLCIEGLSICIYAEITFGIEVLCLELRQIHRHNYGLQELRMETNRLVKLHQKIVEILDRTNDVFHGTLIMQMGVNFSLVSLSVLEAVEARKDPKVVAQFAVLMLLALGHLSMWSYCGDQLSQKSLQISEAAYEAYDPTKGSKDVYRDLCVIIRRGQDPLIMRASPFPSFNLINYSAILNQCYGILTFLLKTLD

>DmelOR65a

MTELRSERKNGNWDRLFGPFFESWAVFKAPQAKSRHIIAYWTRDQLKALGFYMNSEQRRLPRIVAWQYFVSIQLATALASLFYGISESIGDIVNLGRDLVFIITIIFICFRLVFFAQYAGELDVIIDALEDIYHWSIKGPATKEVQETKRLHFLLFMALIITWFSFLILFMLIKISTPFWIESQTLPFHVSWPFQLHDPSKHPIAYIIIFVSQSTTMLYFLIWLGVVENMGVSLFFELTSALRVLCIELRNLQELCLGDEDMLYRELCRMTKFHQQIILLTDRCNHIFNGAFIMQMLINFLLVSLSLFEVLAAKKNPQVAVEYMIIMLMTLGHLSFWSKFGDMFSKESEQVALAVYEAYDPNVGSKSIHRQFCFFIQRAQKPLIMKASPFPPFNLENYMFILKQCYSILTILANTLE

>DmelOR83c

MSTSESPSSRFRELSKYINSLTNLLGVDFLSPKLKFNYRTWTTIFAIANYTGFTVFTILNNGGDWRVGLKASLMTGGLFHGLGKFLTCLLKHQDMRRLVLYSQSIYDEYETRGDSYHRTLNSNIDRLLGIMKIIRNGYVFAFCLMELLPLAMLMYDGTRVTAMQYLIPGLPLENNYCYVVTYMIQTVTMLVQGVGFYSGDLFVFLGLTQILTFADMLQVKVKELNDALEQKAEYRALVRVGASIDGAENRQRLLLDVIRWHQLFTDYCRAINALYYELIATQVLSMALAMMLSFCINLSSFHMPSAIFFVVSAYSMSIYCILGTILEFAYDQVYESICNVTWYELSGEQRKLFGFLLRESQYPHNIQILGVMSLSVRTALQIVKLIYSVSMMMMNRA

>DmelOR83a

MKSTFKEERIKDDSKRRDLFVFVRQTMCIAAMYPFGYYVNGSGVLAVLVRFCDLTYELFNYFVSVHIAGLYICTIYINYGQGDLDFFVNCLIQTIIYLWTIAMKLYFRRFRPGLLNTILSNINDEYETRSAVGFSFVTMAGSYRMSKLWIKTYVYCCYIGTIFWLALPIAYRDRSLPLACWYPFDYTQPGVYEVVFLLQAMGQIQVAASFASSSGLHMVLCVLISGQYDVLFCSLKNVLASSYVLMGANMTELNQLQAEQSAADVEPGQYAYSVEEETPLQELLKVGSSMDFSSAFRLSFVRCIQHHRYIVAALKKIESFYSPIWFVKIGEVTFLMCLVAFVSTKSTAANSFMRMVSLGQYLLLVLYELFIICYFADIVFQNSQRCGEALWRSPWQRHLKDVRSDYMFFMLNSRRQFQLTAGKISNLNVDRFRGTITTAFSFLTLLQKMDARE

>DmelOR49b

MFEDIQLIYMNIKILRFWALLYDKNLRRYVCIGLASFHIFTQIVYMMSTNEGLTGIIRNSYMLVLWINTVLRAYLLLADHDRYLALIQKLTEAYYDLLNLNDSYISEILDQVNKVGKLMARGNLFFGMLTSMGFGLYPLSSSERVLPFGSKIPGLNEYESPYYEMWYIFQMLITPMGCCMYIPYTSLIVGLIMFGIVRCKALQHRLRQVALKHPYGDRDPRELREEIIACIRYQQSIIEYMDHINELTTMMFLFELMAFSALLCALLFMLIIVSGTSQLIIVCMYINMILAQILALYWYANELREQNLAVATAAYETEWFTFDVPLRKNILFMMMRAQRPAAILLGNIRPITLELFQNLLNTTYTFFTVLKRVYG

>DmelOR49a

MEKLRSYEDFIFMANMMFKTLGYDLFHTPKPWWRYLLVRGYFVLCTISNFYEASMVTTRIIEWESLAGSPSKIMRQGLHFFYMLSSQLKFITFMINRKRLLQLSHRLKELYPHKEQNQRKYEVNKYYLSCSTRNVLYVYYFVMVVMALEPLVQSCIMYLIGFGKADFTYKRIFPTRLTFDSEKPLGYVLAYVIDFTYSQFIVNVSLGTDLWMMCVSSQISMHLGYLANMLASIRPSPETEQQDCDFLASIIKRHQLMIRLQKDVNYVFGLLLASNLFTTSCLLCCMAYYTVVEGFNWEGISYMMLFASVAAQFYVVSSHGQMLIDLSTNLAKAAFESKWYEGSLRYKKEILILMAQAQRPLEISARGVIIISLDTFKILMTITYRFFAVIRQTVEK

>DmelOR47b

MNDSGYQSNLSLLRVFLDEFRSVLRQESPGLIPRLAFYYVRAFLSLLCQYPNKKLASLPLYRWINLFIMCNVMTIFWTMFVALPESKNVIEMGDDLVWISGMALVFTKIFYMHLRCDEIDELISDFEYYNRELRPHNIDEEVLGWQRLCYVIESGLYINCFCLVNFFSAAIFLQPLLGEGKLPFHSVYPFQWHRLDLHPYTFWFLYIWQSLTSQHNLMSILMVDMVGISTFLQTALNLKLLCIEIRKLGDMEVSDKRFHEEFCRVVRFHQHIIKLVGKANRAFNGAFNAQLMASFSLISISTFETMAAAAVDPKMAAKFVLLMLVAFIQLSLWCVSGTLVYTQSVEVAQAAFDINDWHTKSPGIQRDISFVILRAQKPLMYVAEPFLPFTLGTYMLVLKNCYRLLALMQESM

>DmelOR10a

MSEWLRFLKRDQQLDVYFFAVPRLSLDIMGYWPGKTGDTWPWRSLIHFAILAIGVATELHAGMCFLDRQQITLALETLCPAGTSAVTLLKMFLMLRFRQDLSIMWNRLRGLLFDPNWERPEQRDIRLKHSAMAARINFWPLSAGFFTCTTYNLKPILIAMILYLQNRYEDFVWFTPFNMTMPKVLLNYPFFPLTYIFIAYTGYVTIFMFGGCDGFYFEFCAHLSALFEVLQAEIESMFRPYTDHLELSPVQLYILEQKMRSVIIRHNAIIDLTRFFRDRYTIITLAHFVSAAMVIGFSMVNLLTLGNNGLGAMLYVAYTVAALSQLLVYCYGGTLVAESSTGLCRAMFSCPWQLFKPKQRRLVQLLILRSQRPVSMAVPFFSPSLATFAAILQTSGSIIALVKSFQ

>DmelOR47a

MDSFLQVQKSTIALLGFDLFSENREMWKRPYRAMNVFSIAAIFPFILAAVLHNWKNVLLLADAMVALLITILGLFKFSMILYLRRDFKRLIDKFRLLMSNEAEQGEEYAEILNAANKQDQRMCTLFRTCFLLAWALNSVLPLVRMGLSYWLAGHAEPELPFPCLFPWNIHIIRNYVLSFIWSAFASTGVVLPAVSLDTIFCSFTSNLCAFFKIAQYKVVRFKGGSLKESQATLNKVFALYQTSLDMCNDLNQCYQPIICAQFFISSLQLCMLGYLFSITFAQTEGVYYASFIATIIIQAYIYCYCGENLKTESASFEWAIYDSPWHESLGAGGASTSICRSLLISMMRAHRGFRITGYFFEANMEAFSSIVRTAMSYITMLRSFS

>DmelOR9a

MSDKVKGKKQEEKDQSLRVQILVYRCMGIDLWSPTMANDRPWLTFVTMGPLFLFMVPMFLAAHEYITQVSLLSDTLGSTFASMLTLVKFLLFCYHRKEFVGLIYHIRAILAKEIEVWPDAREIIEVENQSDQMLSLTYTRCFGLAGIFAALKPFVGIILSSIRGDEIHLELPHNGVYPYDLQVVMFYVPTYLWNVMASYSAVTMALCVDSLLFFFTYNVCAIFKIAKHRMIHLPAVGGKEELEGLVQVLLLHQKGLQIADHIADKYRPLIFLQFFLSALQICFIGFQVADLFPNPQSLYFIAFVGSLLIALFIYSKCGENIKSASLDFGNGLYETNWTDFSPPTKRALLIAAMRAQRPCQMKGYFFEASMATFSTIVRSAVSYIMMLRSFNA

>DmelOR45b

MYPRFLSRNYPLAKHLFFVTRYSFGLLGLRFGKEQSWLHLLWLVFNFVNLAHCCQAEFVFGWSHLRTSPVDAMDAFCPLACSFTTLFKLGWMWWRRQEVADLMDRIRLLIGEQEKREDSRRKVAQRSYYLMVTRCGMLVFTLGSITTGAFVLRSLWEMWVRRHQEFKFDMPFRMLFHDFAHRMPWFPVFYLYSTWSGQVTVYAFAGTDGFFFGFTLYMAFLLQALRYDIQDALKPIRDPSLRESKICCQRLADIVDRHNEIEKIVKEFSGIMAAPTFVHFVSASLVIATSVIDILLYSGYNIIRYVVYTFTVSSAIFLYCYGGTEMSTESLSLGEAAYSSAWYTWDRETRRRVFLIILRAQRPITVRVPFFAPSLPVFTSVIKFTGSIVALAKTIL

>DmelOR45a

MDASYFAVQRRALEIVGFDPSTPQLSLKHPIWAGILILSLISHNWPMVVYALQDLSDLTRLTDNFAVFMQGSQSTFKFLVMMAKRRRIGSLIHRLHKLNQAASATPNHLEKIERENQLDRYVARSFRNAAYGVICASAIAPMLLGLWGYVETGVFTPTTPMEFNFWLDERKPHFYWPIYVWGVLGVAAAAWLAIATDTLFSWLTHNVVIQFQLLELVLEEKDLNGGDSRLTGFVSRHRIALDLAKELSSIFGEIVFVKYMLSYLQLCMLAFRFSRSGWSAQVPFRATFLVAIIIQLSSYCYGGEYIKQQSLAIAQAVYGQINWPEMTPKKRRLWQMVIMRAQRPAKIFGFM FVVDLPLLLWVIRTAGSFLAMLRTFER

>DmelOR7a

MAVSTRVATKQEVPESRRAFRNLFNCFYALGMQAPDGSRPTTSSTWQRIYACFSVVMYVWQLLLVPTFFVISYRYMGGMEITQVLTSAQVAIDAVILPAKIVALAWNLPLLRRAEHHLAALDARCREQEEFQLILDAVRFCNYLVWFYQICYAIYSSSTFVCAFLLGQPPYALYLPGLDWQRSQMQFCIQAWIEFLIMNWTCLHQASDDVYAVIYLYVVRIQVQLLARRVEKLGTDDSGQVEIYPDERRQEEHCAELQRCIVDHQTMLQLLDCISPVISRTIFVQFLITAAIMGTTMINIFIFANTNTKIASIIYLLAVTLQTAPCCYQATSLMLDNERLALAIFQCQWLGQSARFRKMLLYYLHRAQQPITLTAMKLFPINLATYFSIAKFSFSLYTLIKGMNLGERFNRTN

>DmelOR74a

MSFHRYRPRLPGGELAPMPWPVSLYRVLNHVAWPLEAESGRWTVFLDRLMIFLGFLVFCEHNEVDFHYLIANRQDMDNMLTGLPTYLILVEMQIRCFQLAWHKDRFRALLQRFYAEIYVSEEMEPHLFASIQRQMLATRVNSTVYLLALLNFFLVPVTNVIYHRREMLYKQVYPFDNTQLHFFIPLLVLNFWVGFIITSMLFGELNVMGELMMHLNARYIQLGQDLRRSAQMLLKKSSSLNVAIAYRLNLTHILRRNAALRDFGQRVEKEFTLRIFVMFAFSAGLLCALFFKAFTNPWGNVAYIVWFLAKFMELLALGMLGSILLKTTDELGMMYYTADWEQVIHQSDNVGENVKLMKLVTLAIQLNSRPFFITGLNYFRVSLTAVLKIIQGAFSYFTFLNSMR

>DmelOR42b

MVFELIRPAPLTEQKRSRDGCIYLYRAMKFIGWLPPKQGVLRYVYLTWTLMTFVWCTTYLPLGFLGSYMTQIKSFSPGEFLTSLQVCINAYGSSVKVAITYSMLWRLIKAKNILDQLDLRCTAMEEREKIHLVVARSNHAFLIFTFVYCGYAGSTYLSSVLSGRPPWQLYNPFIDWHDGTLKLWVASTLEYMVMSGAVLQDQLSDSYPLIYTLILRAHLDMLRERIRRLRSDENLSEAESYEELVKCVMDHKLILRYCAIIKPVIQGTIFTQFLLIGLVLGFTLINVFFFSDIWTGIASFMFVITILLQTFPFCYTCNLIMEDCESLTHAIFQSNWVDASRRYKTTLLYFLQNVQQPIVFIAGGIFQISMSSNISVAKFAFSVITITKQMNIADKFKTD

>DmelOR42a

MDLRRWFPTLYTQSKDSPVRSRDATLYLLRCVFLMGVRKPPAKFFVAYVLWSFALNFCSTFYQPIGFLTGYISHLSEFSPGEFLTSLQVAFNAWSCSTKVLIVWALVKRFDEANNLLDEMDRRITDPGERLQIHRAVSLSNRIFFFFMAVYMVYATNTFLSAIFIGRPPYQNYYPFLDWRSSTLHLALQAGLEYFAMAGACFQDVCVDCYPVNFVLVLRAHMSIFAERLRRLGTYPYESQEQKYERLVQCIQDHKVILRFVDCLRPVISGTIFVQFLVVGLVLGFTLINIVLFANLGSAIAALSFMAAVLLETTPFCILCNYLTEDCYKLADALFQSNWIDEEKRYQKTLMYFLQKLQQPITFMAMNVFPISVGTNISVTKFSFSVFTLVKQMNISEKLAKSEMEE

>DmelOR71a

MDYDRIRPVRFLTGVLKWWRLWPRKESVSTPDWTNWQAYALHVPFTFLFVLLLWLEAIKSRDIQHTADVLLICLTTTALGGKVINIWKYAHVAQGILSEWSTWDLFELRSKQEVDMWRFEHRRFNRVFMFYCLCSAGVIPFIVIQPLFDIPNRLPFWMWTPFDWQQPVLFWYAFIYQATTIPIACACNVTMDAVNWYLMLHLSLCLRMLGQRLSKLQHDDKDLREKFLELIHLHQRLKQQALSIEIFISKSTFTQILVSSLIICFTIYSMQMSPVLQDLPGFAAMMQYLVAMIMQVMLPTIYGNAVIDSANMLTDSMYNSDWPDMNCRMRRLVLMFMVYLNRPVTLKAGGFFHIGLPLFTKTMNQAYSLLALLLNMNQ

>DmelOR1a

MSKLIEVFLGNLWTQRFTFARMGLDLQPDKKGNVLRSPLLYCIMCLTTSFELCTVCAFMVQNRNQIVLCSEALMHGLQMVSSLLKMAIFLAKSHDLVDLIQQIQSPFTEEDLVGTEWRSQNQRGQLMAAIYFMMCAGTSVSFLLMPVALTMLKYHSTGEFAPVSSFRVLLPYDVTQPHVYAMDCCLMVFVLSFFCCSTTGVDTLYGWCALGVSLQYRRLGQQLKRIPSCFNPSRSDFGLSGIFVEHARLLKIVQHFNYSFMEIAFVEVVIICGLYCSVICQYIMPHTNQNFAFLGFFSLVVTTQLCIYLFGAEQVRLEAERFSRLLYEVIPWQNLPPKHRKLFLFPIERAQRETVLGAYFFELGRPLLVWIFRTAGSFTTLMNALYAKYETH

>DmelOR98b

MLTDKFLRLQSALFRLLGLELLHEQDVGHRYPWRSICCILSVASFMPLTIAFGLQNVQNVEQLTDSLCSVLVDLLALCKIGLFLWLYKDFKFLIGQFYCVLQTETHTAVAEMIVTRESRRDQFISAMYAYCFITAGLSACLMSPLSMLISYQRTGELQPKFPFPSVYPWDNMKLSNYIISYFWNVCAALGVALPTVCVDTLFCSLSHNLCALFQIARHKMMHFEGRNTKETHENLKHVFQLYALCLNLGHFLNEYFRPLICQFVAASLHLCVLCYQLSANILQPALLFYAAFTAAVVGQVSIYCFCGSSIHSECQLFGQAIYESSWPHLLQENLQLVSSLKIAMMRSSLGCPIDGYFFEANRETLITVSKAFIKVSKKTPQVND

>DmelOR98a

MLFNYLRKPNPTNLLTSPDSFRYFEYGMFCMGWHTPATHKIIYYITSCLIFAWCAVYLPIGIIISFKTDINTFTPNELLTVMQLFFNSVGMPFKVLFFNLYISGFYKAKKLLSEMDKRCTTLKERVEVHQGVVRCNKAYLIYQFIYTAYTISTFLSAALSGKLPWRIYNPFVDFRESRSSFWKAALNETALMLFAVTQTLMSDIYPLLYGLILRVHLKLLRLRVESLCTDSGKSDAENEQDLIKCIKDHNLIIDYAAAIRPAVTRTIFVQFLLIGICLGLSMINLLFFADIWTGLATVAYINGLMVQTFPFCFVCDLLKKDCELLVSAIFHSNWINSSRSYKSSLRYFLKNAQKSIAFTAGSIFPISTGSNIKVAKLAFSVVTFVNQLNIADRLTKN

>DmelOR67c

METAKDNTARTFMELMRVPVQFYRTIGEDIYAHRSTNPLKSLLFKIYLYAGFINFNLLVIGELVFFYNSIQDFETIRLAIAVAPCIGFSLVADFKQAAMIRGKKTLIMLLDDLENMHPKTLAKQMEYKLPDFEKTMKRVINIFTFLCLAYTTTFSFYPAIKASVKFNFLGYDTFDRNFGFLIWFPFDATRNNLIYWIMYWDIAHGAYLAGIAFLCADLLLVVVITQICMHFNYISMRLEDHPCNSNEDKENIEFLIGIIRYHDKCLKLCEHVNDLYSFSLLLNFLMASMQICFIAFQVTESTVEVIIIYCIFLMTSMVQVFMVCYYGDTLIAASLKVGDAAYNQKWFQCSKSYCTMLKLLIMRSQKPASIRPPTFPPISLVTYMKVISMSYQFFALLRTTYSNN

>DmelOR67b

MQDQLDHELERIDKLPKLGLLWVEYSAYALGVNIAPRKRSSKYCRLTRILVLIVNLSIIYSLVAFIMENYMISFETYVEAVLLTFQLSVGVVKMFHFQNKVESCSQLVFSTETGEVLKSLGLFQLDLPRKKELLSSVSLILLNNWMIIDRQVMFFFKIVCMPVLYYCVRPYFQYIFDCYIKDKDTCEMTLTYPAIVPYLQLGNYEFPSYVIRFFLLQSGPLWCFFAVFGFNSLFVVLTRYESGLIKVLRFLVQNSTSDILVPKDQRVKYLQCCVRLFARISSHHNQIENLFKYIILVQCSVSSILICMLLYKISTVLEVGWVWMGMIMVYFVTIALEITLYNVSAQKVESQSELLFHDWYNCSWYNESREFKFMIKMMLLFSRRTFVLSVGGFTSLSHKFLVQVFRLSANFFLLLRNMNNK

>DmelOR67a

MDNVAEMPEEKYVEVDDFLRLAVKFYNTLGIDPYETGRKRTIWFQIYFALNMFNMVFSFYAEVATLVDRLRDNENFLESCILLSYVSFVVMGLSKIGAVMKKKPKMTALVRQLETCFPSPSAKVQEEYAVKSWLKRCHIYTKGFGGLFMIMYFAHALIPLFIYFIQRVLLHYPDAKQIMPFYQLEPWEFRDSWLFYPSYFHQSSAGYTATCGSIAGDLMIFAVVLQVIMHYERLAKVLREFKIQAHNAPNGAKEDIRKLQSLVANHIDILRLTDLMNEVFGIPLLLNFIASALLVCLVGVQLTIALSPEYFCKQMLFLISVLLEVYLLCSFSQRLIDASENVGHAAYDMDWLGSDKRFKKILIFISMRSQKPVCLKATVVLDLSMPTMSIFLGMSYKFFCAVRTMYQ

>DmelOR35a

MVRYVPRFADGQKVKLAWPLAVFRLNHIFWPLDPSTGKWGRYLDKVLAVAMSLVFMQHNDAELRYLRFEASNRNLDAFLTGMPTYLILVEAQFRSLHILLHFEKLQKFLEIFYANIYIDPRKEPEMFRKVDGKMIINRLVSAMYGAVISLYLIAPVFSIINQSKDFLYSMIFPFDSDPLYIFVPLLLTNVWVGIVIDTMMFGETNLLCELIVHLNGSYMLLKRDLQLAIEKILVARDRPHMAKQLKVLITKTLRKNVALNQFGQQLEAQYTVRVFIMFAFAAGLLCALSFKAYTNPMANYIYAIWFGAKTVELLSLGQIGSDLAFTTDSLSTMYYLTHWEQILQYSTNPSENLRLLKLINLAIEMNSKPFYVTGLKYFRVSLQAGLKILQASFSYFTFLTSMQRRQMSN

>DmelOR33c

MVIIDSLSFYRPFWICMRLLVPTFFKDSSRPVQLYVVLLHILVTLWFPLHLLLHLLLLPSTAEFFKNLTMSLTCVACSLKHVAHLYHLPQIVEIESLIEQLDTFIASEQEHRYYRDHVHCHARRFTRCLYISFGMIYALFLFGVFVQVISGNWELLYPAYFPFDLESNRFLGAVALGYQVFSMLVEGFQGLGNDTYTPLTLCLLAGHVHLWSIRMGQLGYFDDETVVNHQRLLDYIEQHKLLVRFHNLVSRTISEVQLVQLGGCGATLCIIVSYMLFFVGDTISLVYYLVFFGVVCVQLFPSCYFASEVAEELERLPYAIFSSRWYDQSRDHRFDLLIFTQLTLGNRGWIIKAGGLIELNLNAFFATLKMAYSLFAVVVRAKGI

>DmelOR33b

MDLKPRVIRSEDIYRTYWLYWHLLGLESNFFLNRLLDLVITIFVTIWYPIHLILGLFMERSLGDVCKGLPITAACFFASFKFICFRFKLSEIKEIEILFKELDQRALSREECEFFNQNTRREANFIWKSFIVAYGLSNISAIASVLFGGGHKLLYPAWFPYDVQATELIFWLSVTYQIAGVSLAILQNLANDSYPPMTFCVVAGHVRLLAMRLSRIGQGPEETIYLTGKQLIESIEDHRKLMKIVELLRSTMNISQLGQFISSGVNISITLVNILFFADNNFAITYYGVYFLSMVLELFPCCYYGTLISVEMNQLTYAIYS SNWMSMNRSYSRILLIFMQLTLAEVQIKAGGMIGIGMNAFFATVRLAYSFFTLAMSLR

>DmelOR33a

MDSRRKVRSENLYKTYWLYWRLLGVEGDYPFRRLVDFTITSFITILFPVHLILGMYKKPQIQVFRSLHFTSECLFCSYKFFCFRWKLKEIKTIEGLLQDLDSRVESEEERNYFNQNPSRVARMLSKSYLVAAISAIITATVAGLFSTGRNLMYLGWFPYDFQATAAIYWISFSYQAIGSSLLILENLANDSYPPITFCVVSGHVRLLIMRLSRIGHDVKLSSSENTRKLIEGIQDHRKLMKIIRLLRSTLHLSQLGQFLSSGINISITLINILFFAENNFAMLYYAVFFAAMLIELFPSCYYGILMTMEFDKLPYAIFSSNWLKMDKRYNRSLIILMQLTLVPVNIKAGGIVGIDMSAFFATVRMAYSFYTLALSFRV

>DmelOR94b

MESTNRLSAIQTLLVIQRWIGLLKWENEGEDGVLTWLKRIYPFVLHLPLTFTYIALMWYEAITSSDFEEAGQVLYMSITELALVTKLLNIWYRRHEAASLIHELQHDPAFNLRNSEEIKFWQQNQRNFKRIFYWYIWGSLFVAVMGYISVFFQEDYELPFGYYVPFEWRTRERYFYAWGYNVVAMTLCCLSNILLDTLGCYFMFHIASLFRLLGMRLEALKNAAEEKARPELRRIFQLHTKVRRLTRECEVLVSPYVLSQVVFSAFIICFSAYRLVHMGFKQRPGLFVTTVQFVAVMIVQIFLPCYYGNELTFHANALTNSVFGTNWLEYSVGTRKLLNCYMEFLKRPVKVRAGVFFEIGLPIFVKTINNAYSFFALLLKISK

>DmelOR94a

MDKHKDRIESMRLILQVMQLFGLWPWSLKSEEEWTFTGFVKRNYRFLLHLPITFTFIGLMWLEAFISSNLEQAGQVLYMSITEMALVVKILSIWHYRTEAWRLMYELQHAPDYQLHNQEEVDFWRREQRFFKWFFYIYILISLGVVYSGCTGVLFLEGYELPFAYYVPFEWQNERRYWFAYGYDMAGMTLTCISNITLDTLGCYFLFHISLLYRLLGLRLRETKNMKNDTIFGQQLRAIFIMHQRIRSLTLTCQRIVSPYILSQIILSALIICFSGYRLQHVGIRDNPGQFISMLQFVSVMILQIYLPCYYGNEITVYANQLTNEVYHTNWLECRPPIRKLLNAYMEHLKKPVTIRAGNFFAVGLPIFVKTINNAYSFLALLLNVSN

>DmelOR30a

MELKSMDPVEMPIFGSTLKLMKFWSYLFVHNWRRYVAMTPYIIINCTQYVDIYLSTESLDFIIRNVYLAVLFTNTVVRGVLLCVQRFSYERFINILKSFYIELLQSDDPIINILVKETTRLSVLISRINLLMGCCTCIGFVTYPIFGSERVLPYGMYLPTIDEYKYASPYYEIFFVIQAIMAPMGCCMYIPYTNMVVTFTLFAILMCRVLQHKLRSLEKLKNEQVRGEIIWCIKYQLKLSGFVDSMNALNTHLHLVEFLCFGAMLCVLLFSLIIAQTIAQTVIVIAYMVMIFANSVVLYYVANELYFQSFDIAIAAYESNWMDFDVDTQKTLKFLIMRSQKPLAILVGGTYPMNLKMLQSLLNAIYSFFTLLRRVYG

>DmelOR19a

MDISKVDSTRALVNHWRIFRIMGIHPPGKRTFWGRHYTAYSMVWNVTFHICIWVSFSVNLLQSNSLETFCESLCVTMPHTLYMLKLINVRRMRGQMISSHWLLRLLDKRLGCDDERQIIMAGIERAEFIFRTIFRGLACTVVLGIIYISASSEPTLMYPTWIPWNWRDSTSAYLATAMLHTTALMANATLVLNLSSYPGTYLILVSVHTKALALRVSKLGYGAPLPAVRMQAILVGYIHDHQIILRLFKSLERSLSMTCFLQFFSTACAQCTICYFLLFGNVGIMRFMNMLFLLVILTTETLLLCYTAELPCKEGESLLTAVYSCNWLSQSVNFRRLLLLMLARCQIPMILVSGVIVPISMKTFTVMIKGAYTMLTLLNEIRKTSLE

>DmelOR92a

MLFRKRKPKSDDEVITFDELTRFPMTFYKTIGEDLYSDRDPNVIRRYLLRFYLVLGFLNFNAYVVGEIAYFIVHIMSTTTLLEATAVAPCIGFSFMADFKQFGLTVNRKRLVRLLDDLKEIFPLDLEAQRKYNVSFYRKHMNRVMTLFTILCMTYTSSFSFYPAIKSTIKYYLMGSEIFERNYGFHILFPYDAETDLTVYWFSYWGLAHCAYVAGVSYVCVDLLLIATITQLTMHFNFIANDLEAYEGGDHTDEENIKYLHNLVVYHARALDLSEEVNNIFSFLILWNFIAASLVICFAGFQITASNVEDIVLYFIFFSASLVQVFVVCYYGDEMISSSSRIGHSAFNQNWLPCSTKYKRILQFIIARSQKPASIRPPTFPPISFNTFMKVISMSYQFFALLRTTYYG

>DmelOR19b

MDISKVDSTRALVNHWRIFRIMGIHPPGKRTFWGRHYTAYSMVWNVTFHICIWVSFSVNLLQSNSLETFCESLCVTMPHTLYMLKLINVRRMRGEMISSHWLLRLLDKRLGCADERQIIMAGIERAEFIFRTIFRGLACTVVLGIIYISASSEPTLMYPTWIPWNWKDSTSAYLATAMLHTTALMANATLVLNLSSYPGTYLILVSVHTKALALRVSKLGYGAPLPAVRMQAILVGYIHDHQIILRLFKSLERSLSMTCFLQFFSTACAQCTICYFLLFGNVGIMRFMNMLFLLVILTTETLLLCYTAELPCKEGESLLTAVYSCNWLSQSVNFRRLLLLMLARCQIPMILVSGVIVPISMKTFTVMIKGAYTMLTLLNEIRKTSLE

>DmelOR24a

MLPRFLTASYPMERHYFMVPKFALSLIGFYPEQKRTVLVKLWSFFNFFILTYGCYAEAYYGIHYIPINIATALDALCPVASSILSLVKMVAIWWYQDELRSLIERVRFLTEQQKSKRKLGYKKRFYTLATQLTFLLLCCGFCTSTSYSVRHLIDNILRRTHGKDWIYETPFKMMFPDLLLRLPLYPITYILVHWHGYITVVCFVGADGFFLGFCLYFTVLLLCLQDDVCDLLEVENIEKSPSEAEEARIVREMEKLVDRHNEVAELTERLSGVMVEITLAHFVTSSLIIGTSVVDILLFSGLGIIVYVVYTCAVGVEIFLYCLGGSHIMEACSNLARSTFSSHWYGHSVRVQKMTLLMVARAQRVLTIKIPFFSPSLETLTSILRFTGSLIALAKSVI

>ApisOr1

MGYKKDGLIKDLWPNIRLIQLSGLFISEYYDDYSGLAVLFRKIYSWITAIIIYSQFIFIVIFMVTKSNDSDQLAAGVVTTLFFTHSMIKFVYFSTGTKSFYRTLSCWNNTSPHPLFAESHSRFHAKSLSRMRQLLIIVSIVTIFTTISWTTITFFGESVWKVPDPETFNQTMYVPVPRLMLHSWYPWDSSHGLGYIVAFVLQFYWIFITLSHSNLMELLFSSFLVHACEQLQHLKEILNPLIELSATLDSSVHNPAEIFRANSAKNQSINGIDHDYNGSYVNEITEYGTKGENEPNRKGPNNLTSNQEVLVRSAIKYWVERHKHVVKYVSLITECYGSALLFHMLVSTVILTILAYQATKINGVNVFAFSTIGYLMYSFAQIFMFCIHGNELIEESSSVMEAAYGCHWYDGSEEAKTFVQIVCQQCQKPLIVSGAKFFNVSLDLFASVLGAVVTYFMVLVQLKZ

>ApisOr2

MDVMQKPERFILTPFQKFCIRWSVFFDSSSDRLSRIETVLRTIQFSTIMITSGMTMTSVLIADNKKALESFTYFVICVFMLAIITFAIRTKRFNRAMLLMVVDEFPGYNRPMPDVLKRKMAAIRTSYGDFTMKVIVSYLTLVLFEIPATAMVPLAAASLTDVKLGSQSTQMVVLWFPADTSQVGMYAVSYVIQFLIVVTVKFIITGIMCSFSFFVSQMISEFQILSAYVEHAVEIVEYDQSADKTTEQKLLDHVKNCVMLHDRLIYFKDQLNESYGYIILLELMFSTLYFCLSAFNMIFVGNRFVMIKGLLTLSNYLAELFIFCMYGSMVEDAHMGLLRASYSVAWYAQPVRFRQSLTMVMSRTQTPLQLTVGKVFIANLPLFLSVLKVSYSGVNALRAANAKZ

>ApisOr3

MKTSENITARKMSVYWLTLLLLNGINVYPGHGNRVVRIAAAAYPWMICAFWAFITTSVTTSLALRATSYQEAVEMLTYITGSSSTLALFAIGVHNRPGLHRMLDAVRRDFWDDGRRPAADVLFSRFVRTYGAILPIANVMMCMTPVIWAARNGDIESPAALIFRMWTPWTRLTTARYAVVYAAQFVVSLSVLTSISGMVFAMVLFVTEMQVQVDTLVDAVQDLHVDMWYGDGDGRPDAHPDRRRRAAFDGLVKCIKHHQALITYFNRFKSYFNLLFIVDILYIMVMTCLCASSVLMANGFSAFHIKMMSLLIIVVSQFFFYCLIGEQFSTMNQQIGDCVYFKLVKCKDPMLSRAGLLVILRTQKPLQLTAMGITTYTASLFTFTVTMRSAYAGLNVLYNSSZ

>ApisOr4

MTSIGKKNQRKFYQTLMTLAFFLDTSQYRYISRFVKQFYIFDWMVLVSVAAAFTILEGNYRMPFVMELIQYMIVGFYFTSIFVVFIIKKEAIMSNYNCIQTKFIQWSNKRALHSNAAYKRNIKTVKSLSIPLAILSLSIALGPLISTINDIGKLPLDNRAHFVLFWPTIVDTNKLSMYGIIYTLQVIFTIILYISVLSFNLGYMVFLNELITQFEMLLNGINDAFKYKMDKQFQTLFIDCIRHHQIIIKFLDDLKSYFKWMILIEIIVVQVILAILIYNLTKVNASLGYKVKIAGSILFNLLPICFHCHIGEVVLSLHTRLSNHIYNMPWYDMPNKNKQLIVIMLQRTQRDLTLSSALFSSERASRSLISKVIKQVYTILNVLLKT

>ApisOr5

MQRIDTINMFLQMTGCTDSKAMLYLTYFEFLITFYYLIATYASIVHFEQSVTIQLFALLCMLIECVILLNITFRLYHKNHIREMHQYSRRLGIPDSYRSVINVITKYHLIASNIFVVFPVTYAIFCDSVRVGDPFTFPFLDVLPMHTDNLAIYACKYLVYAISVYIAHVELCFINTTFIYYVGVLKHRLETIVQTIGEAFADNDEQKFKYAIIQHQKLLSYFNTMKKVFSKPILLSMSFNAIYFGLTTSFVIQAIRGYINQAIISICIASSAAAVINITIYTFYGSELMDLHDKILHVLFDNAFFYVSKSFKSSILIMITRVTIPLKFTVGYIFTINLNLLLKILKMSYTVLNVLLSSETIKPHKLSZ

>ApisOr6P

MADIVEIFIQKMICSDDGAGHGAKHISVSLFLAILTCLSLVYSTEDLSIRIYGLLFIIIEIVVCSLIAIRVYHQSQHRDMYQRSQKVGIPENYRWKIAMVIKYHLVMSNVLSVIPLLYTISLDWVRMGDPFTFASVDVLPIKTTNVTVYVCKYIVYALPAYFAHLEICFLNVTFMYNINTINYFIFRFFEDMKTLYEKPLLISIEISTLYIGLSGCTMIQVIQGFVDPIILGFCIVSGVAGFVTISVYCICASNMYDLVLQGMYRTTFFYPGMILWRHSFPGGGQCPGPTRPLPSVAYLGGWSPRPGPIVKLQRTSLTKCVKNHFMMVHDSRCVSTLDVZ

>ApisOr7

MAHAVDNFFQKTGRSDVRRYATIMTCVEFFTYCDLVVTLFFAISAFLSIVYSEEDQLSNRVYGFLWLFVEIHVFGLIVVRLYHQSQCRDMYDRSLLIEQGIPKNYRRTIAMVIAYYCIMSTVHVTVPMLYTISSDSAQVGDPFAFPFADVLPIKTTNPTAYVCKYIVYAFPVYLTHLECCFMNVTFMYFTGVVKRHFQILDQQVQEAVANEDEQKLKIAIEYHQESLKFFKEMETVYEKPLIMTIEFCGLYIGLTGYIMIQCIQGIIHPIILGLCIASSTASLITISIYCICGSNMYDLHDGILNSLFEHQSCYSRNKSLKHLILMMMKRATIPLELKAGSIFKINSNLLVKILKFTYTVFNLLLTSVNRQIKETAIZ

>ApisOr8

MTDIVEIFLQRLGCSDDGDGRGTRRAVFFTYCESAITLFFAVLTYLSLVYSSTGDNLSIRIYGLLCFVIEIYIFAYTAVRVYHQSEHHDMYQRSRQMGIPENYRRKIATVIKYHLVMSNVVLAIPMLCTILLDELRMGDPFTFPFADVMPIKTANVTVYVCKYILYALHTYFAHLELCFLNVTFMYSTGVVKRHFQVLDEQVEEAMVTEDEQKLKIAIIHHQQVLKFFKDMKTVYEKPLLLTIEGFGLYIGLCCCAIIQVIQGFVDQIILGLCMASCVAGFMTISLYCICASNMYDLQNGILNSLYEHRACYSRNKSFKRLNLIMMTRATIPLEFNVYSLFIVNLNLLVKILKLTYSVLNVLLTTINLKFKETATZ

>ApisOr9

MAHIVDIFLQKMVCSHDRGYGMVFFNYCELAITLFFTVSTYPTIADPTENLSIRLYGVLCLLIEVHIFAFIAVRIYHQSQHRDMYQRSQGVEIPGNYRRKIATVIKHYFIISNVFVAVSVLYTILLDWVRIGDPFTFPFIDVLPIKTTNVTVYVCKYIVYALPVYFAHLETCFLNVTFMYSAGVVKRHFQILDEQVEEAVVNEDEQKLKIAIKHHQQVLKYFEDMKTVNEKSILVTIEFCGLYVGLTSCFVIQVMQGFIHQIILGLCIVSSMACLMTIIIYCIYASNMYDLHNGILNALFEHRSCYSRNKSFKRLILIMMTRATIPLEFKAGSVFTINLNLLVKILKFAYTVFNVLLSSINRQFKETRMZ

>ApisOr10

MAHIVDIFFQNMGCSHDHGYGMVFFNCCELAITLFFTVSTYPTIADPTQNLSIRLYGVLCLLIEAHIFAFIAVRIYHQSQHRDMYQHLHGVEIPENYRRKIATVIKHHFIISNVFVAVSVLYTISLDWVRIGDPFTFPFIDVLPIKTTNVTVYVCKYIVYALPVYFAHLETCFLNVTFMFSVGIVKRHFQILNDQVEEAIVNEDEQKLKIAIKHHQQVLKYFEDMKTVYEKPILMTIEFCGLYVGLTSCFVIQVIQGFIHQIILGLCIVSSIACLMTIIIYCIYASNMYALHNGILNALFEHRSCYSRNKSFKRIILIMMTRATIPLEIKAGSVFTINLNLLVKILKFAYTVFNVLLSSINRQFKETAIZ

>ApisOr11

MADIVEIFLQRLGCSDDGAGHDTMRVVFFSCCEFTVSLFLAILTYLSLVYSKEDLSMRFYDLLLLIVEIVVCSLIVIRVYHQSQHRDMYQRSQKVGIPENYRRKITTVIKYHLVMSNVIVVIPVLCTISLDWVRMGDPFTFPSIDVLPIKTTNVTVYVCKYILYALPTYFAHLEMCFMNVTFMYSTGAVKGHFQILEERVEEAMATQDEEKLKIAIKYHQQTLKFFKDMKTVYEKSLLIAIEVSMLYIGLSGCTMIQVMQGFVDPIILGLCMGSCVSTFMTISIYCICASNMYDLHDGILNAIFEQQSCFSRNKSFKQLVLMMMTRATVSLEFRVYSIFTINLNLLVKILKFTYTLLNVLLTSVNRQFKETAKZ

>ApisOr12

MADIVEIFLQLLGCSDDGAGHGTMRVVFFSYCEFAVTQFLAISTYLSLVYSTEDPSIRIYGLLFFIVEIVVCSLIVVRVYHHSQHRDMYQRSQKVGIPENYRQKITMVIKYHLVMSNVLVVIPLLCTISLDWVRMGDPFTFPSIDVLPIKTTNVNVYICKYILYALPTYFAHLEMCFMNVTFMYSTGAVKGHFQILEERVVEAMATQDEEKLKIAIKYHQQTLKFFKDMKTVYEKPLLIAIEVSILYIGLSGCTMIQVVQGFVDPIILGLCIGSCVSTFMTISIYCICASNMYDLHDGILNAIFEQRSCFSRNKSFKRLVLMMMTRATVSLEFRVYSIFTINLNLLVKILKLTYTLLNVLLTSVNRQFKETAKZ

>ApisOr13

MADIVEIFLQKMVCSDDGAGHGTMRLVFFAYFELAVTFFFAISTYLSLVYSTEDLSIRIYGLLFFIVEIVVCSLIVVRVYHQSQHRDMYQRSQKVGIPENYRRKIATVIKYHLVMPNVLLAIPVLYTISLDWAQMGDPFTFPFADVLPIKTTNVTVYVCKYIVYALPTYFGHLEICFLNVTFMYSTGVVKGHFQTLEEMVEEAMVTEDEEKLKIAIKYHQQALKFFKDMKTVYETPILITIEVSILYIGLSGCTMIQVIQGFVNPIILGLCIVSCVSTFITISIYCICASNMYDLHDGILNAVFEHRSCYSRNKSFKRLILMMMTRATVSLEFRVYSMFTINLNLLVKILKLTYTLLNVLLTSVNRQFKETAKZ

>ApisOr14P

MADIVEIFLQKMVRSDDGAGHGTKHVVVFTYCEFAVSLFLAISTCLSLVYSTEDLSIRFYGLLFIIIEIVVCFLIVIRVYHQSQHRDMYERSQKVGIPDNYRRKIAMVIKYHFVMSSVYMTIPLLYTISLDWVQMGDSFTFPSADVLPVKTTNVIIYVCKYIVXSLPANFANLEICFLNATFMYSTGVVKGHFQILEEQVEEAMTAQDEEKLKIAIKHHQQVLKFFKDMITVYEKSLLTAIEVSILYIGLSGCTTIQVIQGFVDPIILGLSMASCVSTFMTISIYCICASNMCDLHDGILNAIFEHRSCFSRNKSFKQLVLIMMTRATVSLEFRVYSIYIIDLNLLVKILKLTYTLLNVLLTSVNRIQLKGTEKZ

>ApisOr15P_NC

NIFTKVRTQRRCKSWHDACGIFTCCEFAVTLFLAISTCLSLVYSKEDLSILFYDFLLFIVETVVCSLIVVRIYHQSQHRDMYQRSQKVGIPENYGRKIVKVIKYHLVMANVLSAIPVLYTISLDWVRMGDPFTFPFADVMLIKTANVTVYVCKYILYALHTYFVHLEICFLNATFMYSTGVVKGHFQIFEEQAEEAMATQGEEKLNIAIKHHQQALKFFKDMKTLYEKSLLIAIRIYTLYAGLSGCTMIQVIQVCGNNNVFDILKLTYTLLNVLTSVNRQFKETA

>ApisOr16P

MKDVDIHASDAMTTTALPGPMPVNANECGEDETAVDLTLFKTIGLHRMLDPGPAGRRLRATYKWIACLIVTIQLMQMVGLYASVNDLQRFASLAVVAFNELTCSFKGILMVTNADRMRAVLDVTLYRYTTCGHRQPANMRLTSATVSTLLRTFTMIGYCMLVVWIIAPLFTGVGYVQVQHSDGTTGAYRKTIDNMWVPGMSETVYNWPPVWATIYVTEVVMMTVDMVIWIMFDCYLITVCFVLNAQFRTLAAGYETIGSQRLILQRGVDFEKSMGGKSDDGDIDSLDYYEELIVHIKDNQNIIEKHDEFLEIVRPVVITQXVSSSISIVGLVFLIEILYFMGEPFTFGPVLRLIFGVITIIIQFYIYCYSFNYIEIAKCTLNFGLYSSNWTEMDLKFKKTLFLGMSMNSTHTKVMKLSPKSIINLEMFAAVMKMSYSVVSVILNSIKKZ

>ApisOr17

MTTTPRVTELTAPASEDLTIVDNRLFKAICLHQILDPTKGGNRYYRLAFMVVMWVSLSVQIIQLVGLYFAVNDLQRFAFTTTVIFNALLCLSKGYVLVVNADRLRASLEVARYEFTSCGARNQRLVRRSRAVLSTILRTFAVLSWVTCFIWALTPLFAMDEYLQVTNADGTVSRYRVTIYNVWLPVPATVYNETTVWSLVYAVEVIACFVNVFSWLLFDSYVVTMCFTFNAQFRTVSASCTTIGHHSDSFRSPPPHAPEGTSDDNNTFNCYDELINRIKDNQSIIKIYDDFFEILQPAILFQIIGGSYSVITLIFLTSLTYLMGFSIISIPVLKVFFGFLSVTFELFLYCYVFNHIETEKCNMNFGLYSSNWTAMDLKFKKTLLFAMNTNSSHRRVMKVTPMSIINLEMFANVMNMSYSIVSVLLNSRVQKZ

>ApisOr18

MTTPRVTAFTVPASSEDLTIVDNKLFKAICLHQILDPTNGGNRFCKLVLMAFMSVSLSVQIMQLVGLYFAVNDLQRFAFTTTTLSYAFLCMTKDYVLLAHADRLRDSLEVARFEFTSCGARDQRVVRRSRAVLSMVLRTFAMLSWSTCVIWALVPLFMMDEYLQVTNADDTVSRYRVTIFNMWLPVPVAVYNATPIWSLIYMVEVIACLFTSFSWLLFDSYVVTMCVTFNAQLRTVSASCATIGHRDCFASLSPHVCTGTHIIKIDDNSILSNCYDELIIHIKDNQNIIKKYDDFFEIIQPVVLFQIIAGSYSVITLIFLTALSYLMGWSIISGPVLKVFFGFLSLTFELFLYCYVFNHIETEMNFGLYSSNWTAMDLKFKKTLLFAMNVNSAHRRVMKVTPTSIINLEMFANVMNMAYSIVSVLLNSRVQKZ

>ApisOr19C

MRSSSATVVDVMLFKAIGLYQLLCPADRGGYSVRSRRALMTALGLSFALHSFQVPYLYYALNDLQRFAYMAAVIIYGMMCSFKGYVLVTNADRLWLVLNAADYGYTGCGHRDPSRLRRCRATLSALLRTFVALSYGTLIVWIVLPFFVDEYTGITNSDGTVTRYRTTIHNMQYPISLAVYNSRPVWALIYVTELYVCIVNVFIWSLFDCYLVTMCFVLNAQFHTMSAGYGTLGIRRTGSSPPDTTFAGVRRIKFDEIESNHYSDLISHIQDNQNLIKMFDVFFEVVRPVVLVQIANGSYSVISLIFLTALMYLMGVPVLSAAFLKFICGLISLTIELFIFCYGFNHIETA

>ApisOr20

MRSSSATVVDVMLFKAIGLYQLLCPADRGGYSVRSRRALMTALGLSFALHSFQVPYLYYALNDLQRFAYMAAVIIYGMMCSFKGYVLVTNADRLWLVLNAADYGYTGCGHRDPSRLRRCRATLSALLRTFVALSYGTLIVWIVLPFFVDEYTGITNSDGTVTRYRTTIHNMQYPIPLAVYNSRPVWALIYVTELYVCIVNVFIWSLFDCYLVTMCFVLNAQFHTMSAGYGTLGIRRTGSSPPDTTFAGVRRIKFDEIESNHYSDLISHIQDNQNLIKMFDVFFEVVRPVVLVQIANGSYSVISLIFLTALMYLMGVPVLSAAFLKFICGLISLTIELFIFCYGFNHIETAKSVLNFGIYSSNWTEMDLTFKKTMLLTMKMNSSHKRAMKVSPNSAVGLEMFARVMNMSYSTVSVLLNSRSZ

>ApisOr21

MIGVHQLLRPDEYGQDNDLYRTAAKVIVGLTLVLQSMQVCRLYLARHDITMFAYIGVMIINGLMCLLKGYMVAAKADQMSATLTAANYAFTKCGGRDPSKLRLCRARLSAILRTFVGLSFGTLIVWLTMPWFMASDYDDQPFIWGVVYVIESIILTVNVFCWTSFDCYLVTMCFVFEAQFCTMSTGYETLGRRRTGAKSSAPQKLGNASTINNVKISDVNYDDLTSHIRDNQNIIKQYDAFFDVVRPMVLIQIANGMYSIIMLIFLTLVTHLSGYSIFSAPILKFVCGLASLTIELYIYCYGFNHIEDGKSTVNFGLYSSNWTEMDLKFKKTLLLAMTLNSAHKRVMKVSPNSIVNLEMFTGVMNMSYSIVSVLLKZ

>ApisOr22

MDVKLFKAIGMYQLLHPVECGLNSDLCRKTAMMVVGLTVGLQLMQVFRLYLARHDIPMFANMAMLVVYGFMCLLKGYTLANHADRICITLEVARYAFTDCGRRDPSLMRRCRARLSTILRTFVGLSFGTLVVWLVMPWFLASEYDGKPLIWAVVYVVESIILTVNVFCWTSFDCYLVTMCFVFEAIFRTMSSGYEKVGQVKANVTLTFPSHYDDLISHIKDNQKIVEKYKTFFEIVTPTVLLQIADGSYTIITMIFLISIAYLNGNSILSPMILKYVCGLVSLTIELYIFCYAFNYIEDGRSTVNFGLYSCDWTDKDLKFKKTVLLAMSMNSANKQVMKLSPNSIVNLAMFSRVMNMSYTIVSTLLSZ

>ApisOr23

MNLNDEQNYIVNLKLMKITGFYHLISPRAPKYFGFNVYKVTAAIEVMTGIFSIIMLFLSSYYYLDNTNELMSHFMLVVAIFFSTLKIFWVSRNSETIWNNMDMTCINFLLYTGHKKEILKKARAKSISTTILFVILWSSVTVAWSISPFFVKDVYLNIKFKDETRRFRYNSLNYVYPISEEFYNEHFLYFYVVEMLSVVFWGHGTVAYDTFVISICITIAFQLKTIAVSYISLNDKKGDIKNLKDNDLEAMFNLKLLIQDQQNMFKKIKEIYKIFEPVTFVQLAAQSMLIILQAYMIFINHYNGFSLLSVPIIKLIVTVAPNIIHLFITCYLYTNINHQQDSMNFALYSSDWTAMSINYKKMLLFTMRMNDAEKLKLKISLRKIVNLEMFASVMHLTYSIISVLAKSYGNTNTKZ

>ApisOr24C

MELQDENSIINLKFMKITGFYQLIRPSDCSKLFNMNIFKMLFIAQILILSITTIMCLYSIYSCVNDVNQVFNYSIMIFATNFAIYKYYFIIKNAKTIWNFAHNMMSTNLLCYKDHTKEVYKVARTRCSTITLISLALWSSILLYWSLLPFSNNNTYLKVKFEDGVHYYRLNALGVVYPVTDIFYNKYFHVFYIIEIVLLILWCKMMWVFDILMISVCISIEYQLKTIAESYRSLGLNHNELMSNKKSTMSVEAISDLEVLIQDQQNIYEKMKNMYQILKPFTFIQVAAESFQIILQSCMILKFYLDGSLSLILFLKFLLPGITYSCHLFLTCYLFSFVNEQKESMNFALYSSNWTDMSIKFKKLLLLTMRMNNAENLKMKISMKRMVNMEMFAD

>ApisOr25

MATGIKTVSKNEDNFMINMRLMKKTGFYQLLDSRSLKVFGHNVFKCMSVVQMSILSSVAFIFVANIYYFSDDINTVMMYSMLITSDVLSILKLYYILQNSDTIWNCIQMTSIDDLSYKYHDRRILEEGRSKSTSYSILIMFMWLNLIVSWSLGPLFVTNYFLIVEQNDEIYRYRFNIMNFAFPATDRFYNDNFMIYYGIEFITLVLWCHCTMNFDVLLLSMNITFKYQLKTISNSFKSMFDFKSLIYDQQRVIENMKNIYRVFRPVVLTQLASESLIIMLLSCIIMLNYFNGISLLSALNLRIFAAISTFLFHIYVICYLFDDVNEQKDSMNLALYSSDWTTSDLQHKILLLHAMRMNNAENLRLQVTRNKIVNFQMFTYIQSYNYSLVVRIAIYIVIKSRVZ

>ApisOr26C

MKLKNEHKFMMDTRLLKIIGFYQLFNPRSPKLFGYNIFKCIAAIQIFIFTMTLFGLIVSIYCCLSDIIEGMRCFWLCVIGMVTIYKHSYIIYYSDIIWKCIQLTFAEGLSYKYYSRRIHENGRKKLELVLEFFIVLWFVNVLFWILTPFAVKNSYTIVKGRNEIYHYHLNIINVIYPVTDKFYNDNFTMFYTMECTIMFVWAHVTLIFDVFIIVICIATAYQLKAIEYSFNTLGN

>ApisOr27C

MVLKNEDKLMANTKLLKTIGLYQILNSSSPKVFGYNIFKCITIIEAFIAAAVVLGCILNAFFCLSDLPETTRYFTIGVMCTITFFKLCCIIGYSDTIWNCLHIITSVKYLTYKYHSKRMLKVGRKKSKSILILFLVVWITVYVNWMFMPIVIQNSYLKVEAGNLIYHFRTNIISLVYPATDKFYNDNFLTFYTMEFIIMLVGIHCTFMFDMLLILMCTTIACYLKTIANSFSTLGNGEHHFMIRYDETKLINAFKIIIQDQQKVIK

>ApisOr28C

MVLKNEDKLMANTKLLKTIGLYQILNSSCPKVFGYNIFKCITIIEAFSAAAVILGCILNAFFCLSDLYVTTRYFTIGVMCAINTFKLCCIIGYSDTIWNCLHIITSVKYLSYKYHSKRMLKGGQKKSKLILIFYLVVWIPVYVNWIFMPIVLQNSYLKVEAGNLIYHYRTNILSLVYPATDKFYNDHFLTFYTMEFIIMLVGIHGAFMFDMFLILMCTTIACYLKTVANSFSTLGNVEDHFLIRYNETKIINAFKIIIRDQQKVIENMNNFCKVIRPVILFQLAAVSSVIILLSIIIIMDYFNGFPLASLKSFTLIATLITYTLELYLICYLLNDVNEQ

>ApisOr29

MVFKNEPKLMENIQLLKTIGLYQILDSHSPKVFGYNVFKCVAVIEAFILTATVYASILNIFYCLSDINEATRYFTLCLIASVPTFKLSYIIGYSDTIWNCLHITSAEYLSYKYHSRCILEVGRKKLKQFLILFVILWIVVFIAWILTPFIVQNSYLRVEARNMTYHYRTNILNLVYPAPDKFYNANFIMYYNIELTISIVWAHSTIIFDILLISMCITIEYYLKTIANTFSTLGNVENQFMIRLDDTKIINDFKIIIQDQQKVIENMKNIYKVIRPVILLQIVAESSIIILLSSITIMNYFNGFSLVSPSNFRFITSIFIYILHIYFICYFLNDVNEQKDSMNFALYSGDWSGKSLKYKKMILYAMQMNSTDQMKLQVTKTRVVNLELFTSVMRTTYTVISVLSEQYAKKTZ

>ApisOr30

MVLKNEPKLMSNIPLLKMIGFYQILNSRSQKVFGCNIFKCIATIEACILIAGVFALILNAFYFLSDINEATSYFTMGVMVSVATCKLFYIIGYSDTIWNCLHITSVEYLSYEYHSRCMIDVGRQKLKSILIISMVVWITTSIGWLLTPLIIQNSYLRVEVKNEIYHYRTNIMNLVYPATDTFYNDHFIMFYIIEFIVPIGFIHCTLIFDILLISMCSTIACYLKTIANSFSTLGNVENHCMGHDEMKTLNNFKIIIRDQQKVIQNMKNIYKVIRPVILLQISAATSIIILLSSMTIMNYFDGLSLISPLNFKFMSTMLTYAMHMYLVCYLLNDINEQIDSLNFALYSGDWTSKSLKYKKMILLAMCMNSAIKLKMQVTMTRIVNLELFAGVMRTTYSIISVFSDQYAKNKIZ

>ApisOr31

METCNDHTCTINLNILKQCGFYQIFDPNSKKIFGWNVYRISFIALTVITQCLIGFGNCGFLFELEDTTDNIDLFLIIFSNSYFCLTEWKVVILIINRKKFLELLDVTDLIFLKSKQCRNNIKILCKHRIRTLQLTNLYFMFCIFVIIEWIIFPIMINSFIAHKTENRRLENVVNRRYPVDVNTYNKYYILFYVFEIIIGVKTVYLVLMVDILLLSIGWAIIIQYEVLAEAFKNIGYNENLQKDHDHDVDDYKYFKSILFDQQQLDSKVKLYFPIVKPIVLMHVAINSVLFIMLSNSFLMVFLSTESFTYKIVNLFKIGTGILYICLQLFLYCHLFDNINLKRKSVNLGIYSCNWTKMDLKFKKLLLLTMQINDANYITIKASTKTIVNLPIFANVLMTSYNIVSVMVKTMSKYRKTZ

>ApisOr32

MNTFKDQDVAINLKLLKQCHFYQIFDSSNRKVFGWNVHQISFVMFAVVVQCFVCYGNAGSVFEKDDVVTNIDYFLIFYTNIHTYLSLWKLIVYLYNAKKILKVFNVTRINFLTSETCCNYSEILHKYRDKTIRFTNWYIIFSIVVIIQWLIFPLVLNMFMISGNSNVRYKNIMNLRYDVSTHTYNQYIIIFVLMETTTLSFAMYFMVMTDLILISFCSAIITQQEVLIHAFKNIGHEDKSQIKYYEQLKSILRDQQHLNLKTQSFYSAMKPIVLLAVAINSTFIIILTYLFILVCLTTESDTSDTALILIKLGSSAVYISLELFLYCYLFENMNIQRERVNLSLYSCDWTKMNLKFKKLLLLSMQMNNANQMLIRASPKKIINLQLFASIISMSYNVVSVMLKTTTPKSSRZ

>ApisOr33C

MSSFKVHDVAINIELYKLLRFYHVFDPSNSKIFGFNFYRFTGILITVFIQSCVLFGLLGCFMEMEDSLDYIELFVFIFVNSSNFLSVMKICVFIYKANDTWDLFEVTRIQFLKSERCRKYRDEILEKVRDKSIKLTNFIFGFAFMTCIIWVIYPLVVNFFMLATDQINNQRYQNVFNMRYPVTINTYNQYYFVFYTIEVILAAFILYNSILIDTFLVSFCWVIIAQYEILTEAFGNIGYEDKFQDQDKDCSIKAYNDLKSLLSEQRRLNLKLKLYYSIVWYIVLTYVVLTSCSIITLTYSFIMV

>ApisOr34C

MDKLRVEEVAINLELMKRSRFYHIFNPNGTKIFNCNAYRLLLFLYGSIVNCIVVFSTLGFFVEMDDTMSFTDLFVAIFVLINFFLCYWRICVFMYNVNAIYDVLSVSRFDLLKSKHCCKNVNVLNDYRDRTIKITNYFFLFSSTVMSQWIIYPLVVIAFTMPEDEYGRFQNIMNLRYPVSTYTYNQYYFIFYLMEVMVAIFTMYAMIFPDILLMSVCWAIIAQQEVLTQAFKNIGHE

>ApisOr35

MGNLKVEEVTINLKLWMLYRFYHMLKTNNYIKIFNCNVYRLILFIYGAIVNCMVAYSIIGFFVEMDDIISNVDVFLVVFVMINFFFCSWRMCIILSKSNTICDVLTVSQLNFLTSKQCVKHSNVLYDYRDRTIKITNYFFVFSVIVLIQWIIFPIMLITFTESDIENIRLPNIMNLRFPVFTYTYNQYYFIFYLMEVTIATFPIYVIIVTDTLIMSFSLAIISQQEVLNRAFRSVGYEENSQSEYYEDLKSILEDQIQLNLKIKSYYSIVRPVILANVAMSSTCFIIVTYVFIVVCFSKEPNQILTIIKLGSSAIFICGQFFLYCYLLDSMNLKREYVNFALYACDWSKMDIKFKKLLLLTMRMNDANNFIIRASPSKVVNLQMFANVISMSYNIISVMLKSMDSNNQISEZ

>ApisOr36

MLYRFYHMLKANNYTKIFNCNVYCLILFIYGAIVNCMVVYSSIGFFIEMDDIISDVDVFLVVFVMINFFFCSWRICIILSKSHIICDVLNVAKFNFLTSKQCFKHLNALYDYRDKTIKITNYFFVFSVIVLIQWIIFPIIVITFMESDVENSRSPNIMNLRFPVSTQTYNQYYFIFYLMEVAIAAFPIYVIMVTDTLILSFSLVTISQQEVINRAFKSIGYEENSQSKYYEDFKSILGDQIQLNLKIKSYYSIVKPIILANVAMSSTSFIIVTYVLIVVSFSKESNQILTIIKLGSSAIFICGQFFLYCYLLDSMNLKKESVNFALYSCDWTKMDIKFKKLLLLTMRMNDANNFMIRASPRKVVNLQMFANVISMSYNIISVMLKSMNSNNQSTEZ

>ApisOr37

MKWLQDHEVAINLALFKRYQFYQIFNPNGSKLLNYDTYKLTNVMFIVAVTTYNIFSAMCFFTDTVDTIDSVDLLLMIFIYSIIIISLLKISVLLFNADQIWELFDLTRFDFLTSRQCRKNVGILCKYRDRSITITNLYQNYSTMVFIIWMITPLVLNTFVVVGGPNQRYHNIFNMQYPVSANIYNQYYYLFYLMEIAMGIFVLNYSMIVDNFLISLCWVIIAQYEVITTAFEKIGNDCELTTLQNEKNNNSFEAYEDLKSILMDQNKLYIKLKSFYRVVWIIVIFLIIIDSVLLIILTYSFVMICSSAESFSIFNILKISTAFFVFVIQLYLYCYLFDVLNDKKESVNFGLYCCDWTKMDLRFKKLLLLATKFNNANTLKIKSTPNKIVNLQLFSSVMTTAFNIVTVMLKTMNGKNZ

>ApisOr38

MSSLKSNEVAINLKLFKVFRFYHIFDPNSGKLCKFNVYHLAWYIINCVIGCILIYGLLGYFTEMEDVIDSIFHIQIMFCYLLYSLSLLKIITFLYKANNIWDLLRVTRINFLTSTQCQAHIGILHKHRNKSIKITNLISGFAIVTTLEWILFPLVLRLLSKTDASHSNKRFENIFNFRFPVTVCEYNNYYFIFYIMESFIAIFMLYAYVVTDVFFISVCYVIIAQYEIIKRAYEIVNCEQTSENNNENKNHNNIIVNDCCDDLISIVMDQQNHYAKLRLFYSTYKLIIVSTVVINSGSIIILTYASVVIFTSPETIPILSIVKLISAFTYMFFVLFFLCYLMECINNKIESVQLGMYSCNWTAMNIKSKKLLLFSMRMHNANKLMIKTTPNNIINLQLFNSVMMTSYNIVSAMVNTRSKZ

>ApisOr39

MFSCDFINRTVNMNSENLFNGGSVAFNLSTYKQLGYYQLLDPKGPHIYGYHLYRTILKIFLLIVQFITIFGVMGFFIEMEDTDPGKSNSFELIIILTNCSLSSLKIYTLISNSKIIWDLFDLTRIDFLRCSRHSKLITKNFVKRCKKSTTITKWIARSFLVGLILWLMGPFIANEEHTEPNTVHRHKNIINIKFPVTMKTYNNYYFVFYLMEVAVGFCIVYGSVLIDAYLMSFCWIISAQYQSVTKAFATFGYNKQGSPKDIYKDFKSIIIDHQNIYLKMKSFYAVVRPITLIHVFAYSCSLIMYAYVIVTIFNSKELFIIAEIMKIVMTVSNVTMEVFIFCYLFELIDNKKEDVNFGLYSCNWTGMDIKFKQLLLMSMKMNNANRFKLKASPDVTINRPFFANVIHTCFKIVSVLIQTQSIDLLNZ

>ApisOr40

MNTTDKKYAFNLTLFKTIGYYQMVDPNSKKIFGFNIYNVINITLVIFTSIMTVIGLSGFFYKTDNITSEENNFKDLQMLFYLSCIGLGNLKIAITVYNADAIWKLFNVAHESFLSNKYCKHDKYKLNNCGKQFARIFPWYFFMFIMTAFAWSVVPIVVNNHAASNETQNNENAYMTNIANMRYPITVKTYNTFYKGFYALEFIMVWYSAYGLVVFDLFIVALLQLLATHYEIISSAYENFKYKAENEDGKLRKEEIQKELVSIIFDCQTIYRKLETLYGFSRPIVLVYMVGDAIGMITMPFLIVMSYVQSGSSIFNTNVMAFSWTLFVVGIQSYMYCSLLQNLNERKEDVNFGLYGCDWTSLDIEIKKLILLAMRMNSSNNLKMKVTSTKFIDLPMFASIVRSSYSVTSVLINSNIDKITKZ

>ApisOr41

MNKQKIYDSNFTLFKLIGVYQMVDPHSQKIFGFNVFHFVSMVFIIFTTSMTILGLSGFFYKVHNTNYNNSDVDTIFIMFYTVCITIGNLKVMIIIFKARQLRNMLEITDESFLSNTFYKRNYYKIVKCGGQLSKFFNLYFSFLLITLTSYAIVPIVLNAHFIDGTTQNTETIQKINIVNLKYPFTVETYNAFFKIFYASECIMLFYIGFGVFALNLFSMTILVIISAQYKLLASAFEVLEYRVNDEDDSLLSDEKLLETFISIVSDNQIIHKKIKMLYDIIRPVGLIQLMADALGMICMPYLIVVYFLEYGSLFNPETMKFVFTFGFAGVQSYMYCSLFQRVTDRREEVNFGLYCCDWTGMDIHMKKLILFTMQMNSSNKLKMNLTTNKCINLPLLSTIIRLSYRISSVMINYNINKZ

>ApisOr42

MPNSSEECVMSSSMAKCTGLHYIIDPEGPTVGGHNVFHVTVMVMIGFTVVCLSMCPFGLYYWANDVTQCIFLLITIVNFSFGCFKAFTLVRHSDDICRCLDVTRFDFSSGAIMSDPDSARFFRKCRDASSTFTGWFAASSHFVLLVWTLLPFVVVGKGVEINNRDGSTSYYHFNPYNMYFLVSSETYNRLHLVFHLVEWAFGLCFVLIMVAFDTFMVTLCVAITCQMRGIGNAYSKLGHDRCATASNVCSDGGIESNKSNNEYLRDLKLIIKDHQAVLGKMNDFYKIVGPVILPQLIVASFTIIFVSFIITRNYFNGMLLTSTQSLKMCCFPIFFYQIYYTCHAFGNLSHQKNVMNFALYSSDWTQMEIKFKKLLLLAMQMHDANKLDMKLTDKLVINLELFTRVINMCYSIFSVLVNSQLKIADKQZ

>ApisOr43

MDSKQEKQYIFNMKLARIMGLYQILFPNSTSFFGYNIYHVVTVFFVSFTFAISMLFPIGLLYLRNDIIAIMYYMGCISNFLLSCFKMVNILYHSKDIWKCIDVTSFNYILYKHYDRNVFKNWQTRSIRITYIYIVIALFAFFCWIFSPCIMNKSVIAIRNIDGSYSKYRMNIFNLYLIASNETYNKNFYIFYVIEIIISICYVYFTIVFDVLMLLVCFAISYQLETISNTIKSLGHEIYTRDNIRSGNSIKLKEKHGILYNDLITIMTDHQNVLKKLNDFYNIFRSITLTQIFIASSSHVFIWFIAAMSIDEGDNADSILSFKLFIVLPLINFQLFMTCSLFGTINEKKDSIIFALYSSNWTNMDLKSKKMILFNLTINNASQLKMKFTNTKIVNLEMFSHTMRFCYSIFSMLINYNKNKMKZ

>ApisOr44

MRFRYIFLQVTKRYITSTLIIMNIRGSNSDSIFNLKLAKIFGFYQILDTETVTFLGRHNVYYGIFVFLIVYQWLLSAIVFLNGLYYPVNNSNIIQDMFYFGFTVNMLYGNYKMYIILNRSKVIWDCLSITKFDFTSYGVQGRHTLNVWRNLSIKYTNIYVMFYITVSILCVGFPVVFSNSFIIIKNHDGLSSAYRLGLVNLFLFISEETYNTHFYVFYIVESLCLIINTLFIIIFDTIVNTLAIALTGQLQMISNAFESVGHKSLHFPNDNVDNKIKLPNENIKYMDHYKDLKTLIIDHQNILKKYDEFLSIFRPTMLLQVFVVSCSIIFLWFIFLTSFIEDDFTQYMALTSMEAIFGIPFCTFQMYMSCFVFNTLNIKKDSITFALYSSNWTEMDMKFKKLILLTMRMNDAHQQKLQYTKTKIINMEIFYHTMRVCYTIVNVMINCKKEKMVQQZ

>ApisOr45

MNIHGSNSDSIFNQKLAKIFGFYQILDTKTVTFLGRHNVYYRIFVFLIVYECLLSAIVILNGLYYPVNNNNIVQAMFYFGFVVNMLYGSYKMYIILSRSKVIWDCLSITKFDFTSYGVQGRHTLNVWRNLSIKYTNIYAIFYLTISILCVASPVVFSNSFIIIKNHDGLSNAYRLGLINLYLFVSEETYNAYFYVFHIVESLGLVINTLFIIIFDTIVNTLAFALIGQLQTISTAFESVGHKSLHFPNNNIDNKNKLPNENIKYMDHYKDLKTLIIDHQNILKKYDEFLSIFRPTMLLQVFVVSSSIIFLWFIFLTSFMEDDFTQYMALTFMEVAIFGIPFCTFQMYMSCFVFNTLNIKKDSITFALYSSNWTEMDMKFKKLILLTMRMNNAHHQKLQYTRTRIINMEIFFQTMRVCYTIVNVMISCKKEKWFQQZ

>ApisOr46P

MQYMYTFLQVTKIYITXTLIIMNIHGNSDRIFNLVLAKIFGFYQILDTETVTFLGRHNVYYKFFVFLIVYDCLISVMVFLNGLYYCINNIVEAMFYMGIVGNAMYANYKMYLILNRSKVIWDCMSVTKFDFTSYGVQGIHTLNKWRNISIKYTNIYMMLFLTLLFFYVLAPVVFSNTFTTMKNHDGSSSAYRLNVINLYLFISEEAYNTYYYVFSIIESFSIVIIVWFVFIFDTIVNTLAIALSGQLQMISSAFESVGHKYLHSPNINIDDKIKLPNENIKYKALNNDLKTLIIDHQSILKKYDEFLSIFRPTMLLQVFVLSYSIIFLWFIFIMCFIEEDVTQYMVMTSVKAAFGIPFCTFQMYMTCNIFNTLEAKKDSITFGLYSSNWTEMDIKLKKTILLTMRMSNAHQKRLQFTRTRIINMELFYETMRVCYTIVNVMLNCKKGKLVZ

>ApisOr47

MDIQNEKHHVFNIRLANLIGLYQTLDPETVKFRGRNVYQIFVAFVALYLLVISLGLFAGCLHLWTYNTATSLLDLLITTNSFYASYKMWIVVYRSNEIWDCLSITRYGFTSLNNRKWNGHDILDRWRARSVRYTSLLAGAYFLTIVFYVGCPLVFGAAVIPIKNQDGSIGSYRLNVINLYLFVSDETYNEYYNTFFFIEALFIVGLIITCLLFDTLLLTLCLGICCQIQMICSAFESVNHNSPSDPHSSAIDNNDEKQIISNEHDLIHDELITIIINHQAVIKKFELFLTIFDRVMLSHIFVSSISLIILWFNLIMSFFNDGTFAISGDTTLKTIVAIPSFLFQIFMVCYLFEDIHNQKDSIVYALYSSNWTEMDMKCKRLILLTMQLNNANQKKLRFTRTKIVNLEMFFKTTGHCYTVVSVLMNYINAKNVZ

>ApisOr48

MDIQNEKHHVFNIRLANLIGLYQTLDPETVKFRGRNVYQIFVAFVALYLLVISLGLFAGCLHLWTYNTATSLLDLLITTNSFYASYKMWIVVYRSNEIWDCLSITRYGFTSLNNRKWNGHDILDRWRARSVRYTSLLAGAYFLTMVFYVGCPLVFGAAVIPIKNQDGSIGSYRLNVINLYLFVSDETYNEYYNTFFFIEALFIAGLVITYLLFDTLLLTLCLGICCQIQMICSAFESVNHNSPSDPHSSAIDNKDEKQIISNEHDLIHDELITIIINHQAVIKKFELFLTIFDRVMLSHIFVSSISLIILWFNLIMSFINDGTFAISGDTTLKTIVAIPPFLFQIFMACYLFEDIHNQKDSIVYALYSSNWTEMDMKCKRLILLTMQLNNANQKKLRFTRTKIVNLEMFFKTMGHCYTVVSVLMNYINAKNVZ

>ApisOr49C

MDKQNEKHHVFNIRLANLTGLYQVLDPGALKCRGRNVYQIFVAFIALYLLVISMVLFVDCLHLSTYNMSTSLLDFLVTTNSFYACYKMWIVIYRSNEIWDCLSITRYGFTSLGNRKLTGHNILDHWRACSVWYTSLLAVAYFLTMVMYVGCPLAFSDTIIPIKNYDGSIGNYRRNVLNLYFFASDKTYNEYYNTFFVIEALFIAGLVIIYLLFDILLVTLCLGICCQIQMICSAFESIYHSSPSDLHSFEIDNNDEKQIISNERDLIYDQLITIIINHQAVIKKFELFLTIFER

>ApisOr50C

MDIQNEKHHVFNIRLANLTGLYQILDPGALKCRGRNVYQIFVAFIALYILVVSMALFVDCLHLWTNNTSMSLLNFFVATNSFYACYKMWIVIYRSNDIWDCLSITRYDFTSLSNRKRIGHGILDRWRARSVWYTSLLAGLYFSTMVIYMGSSLAFCNVLIPIKNHDSSFGNYRLNILNLYFFTSDETYNEYYNTFFVVEAWLSVAVTIFYILFDILFVSLCLAICCHMQMIFTAFSSVNHKSLSDFHSSSIDNTVEKHIISNEHELIYDELITIIIDHQAVIKKFELFLNIFERVMLSHIFVSSISLIILWFNLIMRFFNDSTFAISGDTTIKTIVAIPSFLFQIFMACYLFENVHNQKDSIRYALYSSNWTEMDMKCKKLILLTMQMNNANQKTLRFTRTRIVNLEMFFK

>ApisOr51

MDIRNEKNHVFNIRLAKLTGLYQMLDPGTTKFLGRNVYQMFVALFLLYLLVSAVALMVGCLHLWTYDTSMSILDFFLAINSFYACYKMCIIFYRSDDIWECLSITRYGFTSSSLRKRNGHGDVLDRWRARSVWYTSSMAGAYCFSFVFYIRCHLIFGDAIIPVKNLDGLIGNYRWNVLNLSFLTSDETYNEHYNTFFVIEALFIVVITIFYLIFDILFLTLCMAICCQMQMICDAFKSVNHKSLGDPHSSAIDNTDEKQIITSERDLIHDELITNIINHQAVIKKFELFLTIFERVMLSQIFVSSISLIILGFNLIMSFFNDGTFSISGDTTVKTIVAIPSFLFQIFMACYLFENIHDQKDSIKFALYSSNWTEMDMKCKQLILLTMQLNNANQKKLRFSRTKIVNMEMFFKTMGHCYSVLSVLINYMNAKNDZ

>ApisOr52

MDIWNENNHVFNIRLAKLIGLFQILNPGSIKFLGRNVYHIVVAINMLFVCIVAMVFFASGVYYWSDGVLVGVDYGWKGITALFLTYKMWKVVYHSNDIWDCLTITRYDFTSQNLRDRQILDRWRERSVWITNTMAIAYLMSLVILLSGSLMFRHDTLTVKNHDGSVGNYRQNIMNLYFIVTDETYNAHYKTFYFIEMLFTVGGGTLFTAFDVLLVTLCLAISCQFQVVNAKFESVGYKSLCDSRTKISDNKDEKQNISNEHDLIYDELISIIKDHQEVIKKYYELLTIFKRLMLLHVFYSSISLIVIWFIFIMSFTTEDRFFAWEVTTMKIICLIPSFSFHLYMTCSLFDNLHKQKDSIIFALYSSNWTEMNMKCKKLILLTMKMNNANQKKLKFTRTKIVNLELFYNIIRSCYNVVWFLINYIKVKYELZ

>ApisOr53

MDVWDKNNHVFNIRLAKLTGLFQVLSPESIKFLGQNVYHIVVTVILLYMGIISMILIVSGLYYWADNILLSVDYGWKGITALFSTYKMWNVVYHSNGIWDCLAITRYDFTSHGLRNRHILDLWRERSVWITNTLTIIYVSTTVLFAGSSLMFHDNISTVKNHDGSVGNYHQNLFNLYLIVTDETYNAHYETFYFIETLFAVGLATLFIAFDLLLVTLCLTVSCQMQVVNVAFESVGNKPLNDPHTPSIDNADEKKNISNEHDVIYDELISIIMDHQAVMKKYNDLLRRFKRPMLLQVFYSSTSLIVIWFCFLMSFSTEDRFAASEVTTIKIICSIPSISFQLFLVCSLFDNLHKQKDAIIFALYSSNWTEMNMKCKKLILLTMKMNNANQKKLKFTRTKIVNLELFCIIIHNCYSVVSVLIKCIKVKYEZ

>ApisOr54

MDIRDDQNHVFNVTLAKLIGVYQTLDPKTTKYRGINVHRIVMAFIILYIGVTAVILTLSGAYYWTNNMPLSVDCYWKGIVSYTMCYSMWLIVHYSNDIWNCLSITCYGLTSNSLRDRHILDGWRELSVLITTILTFVYFMSAIIFYISSLALSNDLLPVKNHEGLVRNYRYNLFNLYLFVSEETYNVHYNIFYMVEALGVVSLLISFFVFDILLVTFCLAITCQMQMICAAFESVGHKSLANDLSSIDSRDEKKEITNKHDLIYDELKTIIMDHQEVMKKYDMFLTLFKRVLLIQMVVFSVAFIITWFCFIMSFSNEERFKSPTIFIIKIFCGIPPNVFKLFATCYLFEKLHNQKNSIVFALYSSNWTEMDMKCKKLILLTIKMNNANYKKLKFTTTKIVSLEMFFKTMRDSYSVLSVLINYIKNKDESIZ

>ApisOr55P

MDIRDDQNHVSNMVLAECTGLYQMLDPQTIVRCRGLNVYHIFLLSTTLFMCVVGVIMTISGVYYWPNNMPLCVDYYWKGIIPLYMCYSMWVIVHYSNDMWNCLSITCYCFTLHSIRLXLTTILTVTYIITSVIIYFVSSLAVSNDILQVKNRDGSVSNYRYNVINLYLFVSDETYNXHYNMFYIGEALFIVFITIALFVFDFLMVTLCLAIHCQMQMXCTAFESVGQDDIPLVDCQDENKKSPNEHDLIYDELKTITMDHQAVMKKYDAFLTIFERVLLLQMLFYXIAFIIIWFCFIMNFSNDERFKISRIFTVKICCVIPTNLFWNLLLKDSIIFALYSNNWTGYDMDMKCKKLILLTMNITNANYKELKFTITRIVNLEMFFKTMRDSYSILSVLINYIKNMDKZ

>ApisOr56P

MDIRDDKNHVFNTTLEKCTGPYQMLDLSTLQFRGKNVHHMVLIFITLFMCVISVILSVSVVYYWTENIPLSIDYIWKSFFSLYMCYSMWVIVHCSNDIWNCLSITCYGFTSHSLRDRHILDRWREQSVLLTTILTVTYVTAVIIYVVGSLALSNDIQSVKNHVGSVGSYRHNLINLYLFVSDGMYNAHYNIFYMVEALCTISILIAFFVFDFLLVTLCLAVCCQMEMIXSVFESVGHKSLGDNLSLVDYRDEIKETPNEQDLMYDKLKTIIMDHQAVMRIYDEFISIFERVLLTLVVVLSIMFIVLWFCFIMSFSGDGRFRSSGIFIIKMFCAIPPYLFKLFAVCYLFGNLHDKKDSIIFALYSSNWTEMDMKFKKLTLLTMKMINANHKKLKFTRTKIVNLEMFFKAMGHCYAIISVLVNYIQSNVEZ

>ApisOr57P_C

MDIWNDQNHIFNITLAKLIDLYQTLDPETTKYRGKNVYHIVVAFIRYTLYWCNCGDIAVILNITSVYYWTDNMPLSIDNFWKGIISICICXPMCVIVHYSNGVCRSRGVTSHSLQDRHNILDRWRELSVLSTTILAVAYLTTMIIYFFSSLELSNDMLQVKNRDGSVSNYSFNVINLYLFVSNETYNAHYNMFYMVEALFCACIVIALFVFDFFLVTLCLAIRCQMQMVCTAFESVGHKSFGDDLS

>ApisOr58P_C

MDIREDQNHVFNITLAKCWISRPCNFVKNVYHMVLIFIMLFMCVISVILSVSLVYYXTNNMPLSIDYNIWKSFLSLYTCTYVCCSMWVIVHYPNDILWNCLLITCYGFISHSLXDRHIILDRWRELSVLLMTILTVAYLTKMIIYFVSSLELSSNILQVKNRDGSVSSYRYNLLNLYLFVTSGDMYNAHYNMSYMVEALFCVCIVIALFVFYFLLVTLCLVIHCQXMICTAFESDGHKSFGDNLSLV

>ApisOr59P_NC

LDPSEMDIRNEMNHVFNIKLAKFIGIYQVLDPGSLKYRGRNVYHIVVACLMLFMCLNSVLLDVSDLYYXDNISISVDCFWKADALLYLVYKMWIVFHWNDIWVCLYYDYVIMTYYYVXRYDITSFSFRNRHILNHXRERSVRLTTMFALMYLCFPAFYLGSTLAFRNDILPVKNHDGSVGFYRQNVMNFYLIVSDQTYNANYNTFYIVEALYLAMMVMVFLIFDIVLVTLCIALCSQMQMIFSAFESVGHKSLFDSHSPIGEYDYKVHLKVYCIKKSIHIKFIVFLLINLFNSXTLDRYSHVQHFLMTILFFYXKKDSIIFALYSSNWTKMDMKCKXLILLTMKMNNANQKKLKFTKTKIVNLEMFYKVSNSIITI

>ApisOr60C

MDFRNEKNHLINIKLAKITGLHQLLDPETVKYRGQNIYHVVMSCVSLYMCFISMILLLSGLYYWTGNIPISMNYFFKSVTTFYLIYKMWFLVRHSNYIWNCLSITCFDFTSFSNRHRHILDHWRDRLAWFTTTYATIYFTTTVSYLAITLAFSENKSPVKSHDGSIGYYRQNVLNLYLIVSDESYNAHYYMFYFVEALFGTFIGLFFFIFDFLLVTLCFSMCCQMQIICSAFESVGHKSLRVQHSPIWKGSGLEFFTWAPYYQVTPLYNAQLYM

>ApisOr61C

MDFRNEKNHFFNIKLAKMTGLYQMLDPKTVTYRGRNIYHIGMACVLLYMCLFLMIFILSNLYYWTVNIPISMDYFWKAETTLYVIYKMWFVIHHSNDIWNCLSITRFDFTSFTNRKRHILECWRERLAWFITIYATMYFTATVSYCAITLAFNENKSPGKSYDGSIGHYRQNVMNLYLIVSDETYNAHYYMFYFIEALFGIILGLLIFIFDFLLATLCFSMCCQMQIICSMFESVGHKSLHDHHSPI

>ApisOr62C

MDIRNEKNDFFNIKLAKIVGLYQMLDPKTVKYRGRNIYHIGMACVLLYMCLFLMIYILSCLYYWTVNIPISMDYFWKAEITLYVIYKIWFVVQHSNDIWNCLFITRHDFTSFGNQNRDILDYWRDRLAWLTIVYATMYFMAMFSYLAITLVFSDEKSLVKNHGGSIGYYRQNAMNLYLIVSDQTYNAHYYIFYFVEASFGIFIALLFFIFDFLLVTLCFSMCCQMQIICSAFESVGHKSLHDHHSLI

>ApisOr63P_C

MDIRNVKNHVFNIKLAKLIGLYQLLDPETVKYRGRNIYHIAMVCVLLCMCLISLILILSGLYYWTNNISISMDYFWKSEMTLYVSYKMWFVVCHSNDIWNCLSIARYDFTPINDQNRHILDRWRQRLVWLTTIYVVMYSTATAXYLFITLVFSQDKSPVKSHDGSIGYYRQNAMNFYLIVSDETFNTHYYMFYFVEALFVAFMGLFFLIFDCLLVTLCFCMCCQMQIICSAFESVGHKSLRDLDSQIGNYKSIKMTPNEHDLIYNELKTIIVYHQRLM

>ApisOr64

MDIRNETNHVFNIKLAKLIGLYQMLDPGAAKCRGRNIYHIGMACVLLYMCLVLMILVISGLYYWTVNVPISMDYFWKSESTLYVIYKMWFVVHHSDDIWNCLSITRYDFTSFSNRNRHVLDRWRERVSWSTTIYAIIYFTTCVSYLAITLAFSEVKSPVKNHDGSIGYYRQNAMNLYLIVSDDTYNAHFYTFYFVEALFGNLIGLFFFIFDFLLVTLCFSMCCQMQIVCSAFESVGHISLRDHHPPIDYTDENIKISPDEHELIYNELKTIIKDHQAVMEKYKDFLSLFRRVMLLHIFVSSLLVIAIWNTFIMSFSDDDRFQTSDVIVKKMFCVIPSILFQIYMVCYLFGNIHNQKDSIIFALYSSNWTEMDMKCKKLILLTMKMNNANQKKLKFTRTKIVNLEMFYKTMGDCYTVISVLVNYIZ

>ApisOr65P

MDIRNEKNDFFNIKLAKMTGLYQMLDPKTVKYRGRNIYHIGVACVLLYMCLFLMIFILSNLYYWTVNIPISMDYFWKAESTMFIMYKAWFVVHYSDDIWNCLSITRYDFTSFSNRNRHVLDHWRERVSWSTIIYGIVYFTTCVSYLIMTLAFSQIKSPVKNHDGSIGSYRQNSMNLYLIVSDETYNTHYYMFYFVEALFTIFLGFFPFIFDYLLATLCFSMCCQMQIICSMFELVGHKSLHDRHSPIDYTDENIMISPNEHKLIYDELITIIRDHQAVMKKYEDILTLYRRVMLSHIFTSSLLVILLWFTFIMSFSNDERFNTSDVIVKKLFCVIPSALFQIYMLCYLFGNIHDQMNIYSHCTYSSNRTELEMXKCKKLILLTMKLNNANLKKLNFTRTNIVNLEMFFKTMGYCYTVTSVLVYYIQTKYEYILFZ

>ApisOr66P

MDIRNEKNDFFNIKLAKMTGLYQMLDPKTVTYRGRNIYHIGMACVLLYVCFFLMIFILSDLYYWTVNIPISMDYCWKAETTLFVSYKMWFIVQHSNDIWNCLSITRYDFTSGTNRNKHILEHWRERLALLTTNYAIMYSTTVVSYLVFTLAFSEISIVKNHDGSIEYYRQNIMNLYLIVSDETYNTHYYMFYFVEALFSSFLGLFNFIFDFLLATLCFSMCCQMQIICSMFELVGHKSLHDRHSLIDGNIKISPCEHELIYDELITIIMDHQAVMKKYEDILTLYRXVILSHIFTSSILVILLCFTFIMSFSNDERFKTSDIIVQMFCLIPSILFQLYMLCYLFGNIHDQKDEIIFALYSSNWTEMDMKCKKLILLTMKLNNANQIKLKFTRTRIVNLEMFFKTMGNCYLVISVLVNYIKTZ

>ApisOr67

MDIRNQKNDFFNIKLAKITGLYQMLDPKTVKYRGLNIYRIVMACVLVYMCFISMILLLSGLYYWTGNIHISIDYFFKSVTTFYLIYKIWFVVQHSNDIWNCLSITRHDFTLFGNQNIDMLDHWRDRLAWLTIIYATMYFMGMFSYLAITYVFSDEKSLVKNHDGSFGNYRQNVMNLYLIVSDQTYNAHYYIFYFVEASFGTFIALLFFIFDFLLVTLCFSMCCQMQIICSAFESVGHKSIRDCYSPIDVNLIISPNEHDFIYDKLITIIMDHQAVLKKYEDFLTLFRRVILSHIFVSSFLVIALWFAFIMSFSNDERFKTSDIIVKKMFCAIPSILFQIYMVCYLFGNIHNQKDSIIFALYSSNWTEMDMKCKKLILLTMKLNNCNQKKLKFTRTKIVNLEMFFKTMGNCYSVISVLVNYILKQDEZ

>ApisOr68N

DQTYNAHYYMFYFVEASFGILIALLIFIFDFLVVTLCFSMCCQMKIICSAFESVGHKSLGDHHSPIDGNIMISPYEHELIYDELITIIMDHQLVMKKYEDFLTLFRRVMLSHIFVSSFLVIAVWFTFIMSFSNDERFKTSDIIVKKMFCAIPSILFQIYMVCYLFGNIHNQKDSIIFALYSSNWTEMDMKCKKLILLTMKLNNSNQKKLKFTRTKIVNLEMFFKTMGNCYTVISVLVNZ

>ApisOr69

MDFRNEKNHFFNIKIAKLTALYQMLDPETIKFRGRNIYHIVTACVLVYMCLISMILLLSGVYYWTGNIPISMDYFWKSVSAFYIIYKTWIIIRNSNDIWNCLSITRYDFTSFSDRNRHILDRWRDRLTWFTTIYATMYFTAAVTYLAITLAFGENKSSVKSHDGSIGYYRQNVMNLYLIVSDETYNAHYYIFYFIEALFAAFIGLFFFIFDFLLVTLCFSMCCQMQIICSAFESVGHKSLRDQYSPIVENIKISPKEHDLIYDELKKIIMDHQLVMKKYEDFLKLFRRVMLLHIFVSSLSVILLCFTLIMSFSNDERFKTSEIILKKLFCLIPSILFQIYMVCYLFGNIHDQKDEIIFALYSSNWTKMDMKCKKLILLTMKLNNANHKKLKFTRTKIVNLEMFFKTMGHCYTVISVLVNYISTKDEZ

>ApisOr70

MIVDRLLKDILCCYLFLVFQQYIDIITIDIRNETNHVFNIKLAKLLGLYQILDPGALKCRGRNIYHIVTSCLLLYACLISTILIISGLYYCTNIPVSIDYFWKSVTTIYVIYKTWIIIHYSNDVWNCLSITRYDLTSLTDRNRHILDRWRERLAWLTNIYVIMYCMTLVLYLVITLAFSEVKSTVKNRDGSVGYYRQNALNLYLIATDDTYNVHYYTFYFIEASFVAFITLYFLIFDVLMVTLCFGMCCQMEIICSAFKSVGHKFVTDPHSPIDDIKNQTSNEHDLIYDELKTIIMDHQVVMKKYEDFLTIFRRVMLLHIFVSSFTVILLWFTFIMSFSNDDRFKTSDVIIIRMICEIPSILFQIYMMCYLFGNINDQKDEIIFALYSSNWTEMDMKCKKLILLTMQLNNANQIKLKFTRTKIVNLEMFFKTMGHCYTVISVLVNHIQTKNEZ

>ApisOr71P

MDIRNGTNHFKIKLAKLLGLYQMLDPGAVKFRGRNIYHIVTSCLLLYACLESTILILSGLYYCTNITVIMDYFWKAVSTIYIIYKMRINIHHSNDVWYCLSITRYDLTSLTDRKKHILDRWRERLVWLTNIYVIMYFMTLVIYLVITLTFGEDSIENQTSNEHNLIYDELKTIIMDHQVVMKKYEDFSTLFRRVMLFFVSSFTVILLWFTFIMSFSNDDRFNTSDVIIIKMICEIPSILFQIYMMCYLFCNINDQKDEIIFALYSSNWTKMDMKCKKILLTMQLNNANQMKTKFTRTKIVNLEIIFKAMGHIGYTVILVLVNYIQTKNEZ

>ApisOr72

MDIRNETNHVFNIKLAKLLGLYQILDPVALKFRGRNIYHIVTSCLLLYACLISTILILSGLYYCTNIPVIMDYFWKSVSTIYTIYKMWIIIHYLNDVWNCLSITRYDLTSLTDRNRHILDRWRERLAWLTNIYATTYFTTLVIYFVITLAFSEGKLTVKNRDGSVGYYRQNIMNLYLIASDDTYNAHYYTFYIIEASFIVFMTFYFLIFDILLLTLCFGMCCQMEIICSAFKSVGHKSLCDHHSPINDIKNQTSNVHDLIYDELKTIIMDHQVVMKKYEDFLTLFRRVMLLQIFVSSFSVILLWFTFIMSFFNDDRFKTSEVMVTRMFCLIPSTLFQIYIMCYLFGNLYDQKDEIIFALYSSNWTEMDMKCKKLILLTMQLNNANQIKLKFTRTKIVNLEMFFKTMGHCYTVISVLVNYIKTKNEZ

>ApisOr73P_C

MDIQYETNNVFNIKLAKLLELYQMLDPGAVKCRGRNIYQIVTWCLLLYACLISTSLILSSLFYCTNIPVSMDYFWKTITTXIHYSNDVWNCSSITRYDFTSLTNRKRHILDRWRECLSWLTNIIYAIMYFTSIVIYFVITLAFSECKSTVKDRDGSSGYYRQNPLNLXSIATDDTYNAHYYTFYFIEASFVTFMILYFLIFNILLVTLCFGMCSQMQIICSAFESVGQKSFRGQHSPIIDATDDIKNETSNVHDSIYDELKTIIMDHQVVMK

>ApisOr74P

MDIQNETNNVFNIKLAKLLGLYQMLDPGAVKCRGRNIYQIVTSCLLLYACLISTILILSGLYYCTNIPVSMDYFWRAVTVIYIIYKTWIIIHYSNDVWNCLSITRYDLTSLTDRNRHILDRWRERLSWLTNIYAIMYFTSIVIYFVITLAFSECKSTVENRDGSGGYYRQNVMNLYLIASDDTYNAHYYMFYFIEASFIAFMTLYFLIFDILLVTLCFGMCCQMQIICSAFESVGHKSFRDPHTPIIDSTDDIKNETSNAHDLIYDELKTIIMDHQVVMKKYEDFLTLFXRVMLLHIFVSSFSVIFLWFTFIMSFSNDDRFTTSDVIVTKLFCLIPSILFQIYMMCYLFGNINDQKDEIIFALYSSNWTEMDMKCKKLILLTMQLNNANQIKLKFTRTKIVNLEMFFKTMGHCYTVISVLVNYIKTKNGZ

>ApisOr75P_C

MDVWNETNHVFNIKLAKLLGLYQMLDPGALICREQNIYHIVTSCLLLYARLISTMLILSGLYYCTGITVSMDYFWKSAMTIYVIYKTQIIIHYSNDVWNCLSITRYDFTSLTDRNRHIPDRWRERLAWLTNIYDIPYFTTIVIYFVITLEFSEGKSIVKNRDGSVXYYHEKAVNLYLIVSDETYNTNYYMFYFSGSSSIAFMXIYFLIFDILLVTLCFGMCCQMEIICSAFKSVGHKSLCDHHSPIGE

>ApisOr76C

MDIRNVKNHIFNIKLAKLTGLYQMLDPETTKCWGQNIYHVVMSCILLFMCLIPMTLMLSGLYYWTVNIPISMDFFWKSVSIFYIIYKTWVIIRNSNDIWNCLSITSYDFTSFSNRNRHILDDWRDRLARFTTIYAILYFTGTVSYFASTLALSEGKTPVKNIDGSIGHYRQNVMNFYLIASADTYNSHYYMFYFVEALFLALFATCFLIFDILLVTLCFGMCCQMEIICSAFELVGHKSLRDPHSPIIDENENTAFNEHDLIYDEEIKTIIMDHQVVMKKYEDFLRLFQPMMLLHIFISSFSVISLCFTFIMSFSDDDRFRTSDVIVKKMFCSIPPALFQIYMVCYLFGNIHDQKDSIIFALYSSNWTEMDMKCKKLILLTMKLNNANHKKLKFTRTKIVNLEMFFK

>ApisOr77

MDIQNMKNHIFNVKLARLTGLYQMLDPDTIKCRGRNIYHVVMSCVLLYMCLISMILMISGLYYWMVNVPISIDYFWKSVSTFYIIYKTWIIIRHSNDIWNCLSITRHDFTALTDQNRHILERWRKRLAWLTTIYAIMYTMSVVSYLVFTLAFNEGKTPVKNHDGSIGYYRQNVMNLYLIVSDETYNAHYYTFYFIEALFLGLIGLFYLIFDILLVTLCFGMCCQMQIICSAFESVGHKSVRDPHFPIDYTNGNTNITPNEHDLIWDELRTIIMDHQAVMENFKYNFFYFFFSININKMYKLFSCLNCIKFSTLIANSFSNDYRFKTSEVIVKKMFCSIPPILFQIFMVCYLFGNLHEQKDSIIFALYSSNWTEMDMKCKKLILLTMKLNNANQKKLKFTRTKIVNLEMFFKTMGDCYTVISVLVNYIQTKSEZ

>ApisOr78

MDIQNMKNHIFNIKLARLTGLYQMLDPDTIKCRGRNIYHVVMSCVLLYMCLISMILMISGVYYWTVNIPISIDYFWKSVSTFYTIYKTSIIIRHSNDIWNCLSITRLDFTTFSNRNRQVLDRWRERLSWLTTIYAIIYTMSVVSYLVFTLVFNEGKTPVKNHDGSIGYYRQNVMNFYLMVSDETYNAHYYKFYFIEALFAAFMGFFFFIFDFLLVTLCFSMCCQMRIVCSAFESVGHKSVRDPQSPIDEHDLIWDELRTIIMDHQAIMEKYKDFLSLFRRVMLAHIFISSLSVIALWFTFIMSFSNNDRFKTSELIVRKMFCAIPTILFQIFMVCYMFGKLHEQKDSIIFALYSSNWTEMDMKCKKLLLLTMKLNNANQKKLKFTRTRIVNMEMFFKTMGNCYTVISVLVNQIIKQNEZ

>ApisOr79F

MDIQNMNKHIFNIKLARLTGLYQMLDPNTIKCRVRNIYYVVMSCVLLYMCLISMILMFSGLYYWTVNIPISIDYFWKSVSTFYTIYKTWIIIRHSNDIWNCLSITRHDFTTFSNRNRHVLDRWRERVSWSTTIYAIIYTMSMVGYLVFTLAFDEGKTPVKNHDGSIGYYRQNVMNLYLIVSDETYNAHYYTFYIIEALFVGFLGLFFFIFDFLLVTLCFSMCCQMQIVCSAFESVGHKSVCDPHSPIDYTNGNTKITPNEHDLIWDELRTIIMDHQAVMEKYKDFLSLFRRVMLVHIFISSITVIALWFTFIMSFSNDYRFKTSEVIVIKTFCSTPPVMFQIFMVCYLFGKLHEQKDSIIFALYSSNWTEMDMKCKKLILLTMKLNNANQKKLKFTRTRIVNLEMFFKTMGNCYTVISVLVNQIIKQNEZ

**IR sequences**

>DmelGluRIIC

MWQRILLLGCMWSAFFMCRSRGQQINIGAFFYDDELELEKEFMTVVNAINGPESEQTMRFYPLIKRLKPEDGSVTMQEHACDLIDNGVAAIFGPSSKAASDIVALVCNSTGIPHIEFDISDEGIQAEKPNHQMTLNLYPAQAILSKAYADIVQNFGWRKFTIVYDADDARAAARLQDLLQLREVHNDVVRVRKFHKDDDFRVMWKSIRGERRVVLDCEPNMLVELLNSSTEFGLTGQYNHIFLTNLETYTDHLEELAADNETFAVNITAARLLVNPDPPPYSLPYGYVTQRDNIVYESSDPPRTLIHDLIHDALQLFAQSWRNASFFYPDRMVVPRITCDFAASGGRTWAMGRYLARLMKGTSGVNNTNFRTSILQFDEDGQRITFNIEVYDPLDGIGIAIWDPRGQITQLNVDVKAQKKMIYRVATRIGPPYFSYNETARELNLTGNALYQGYAVDLIDAIARHVGFEYVFVPVADQQYGKLDKETKQWNGIIGEIINNDAHMGICDLTITQARKTAVDFTVPFMQLGVSILAYKSPHVEKTLDAYLAPFGGEVWIWILISVFVMTFLKTIVARISKMDWENPHPCNRDPEVLENQWRIHNTGWLTVASIMTAGCDILPRSPQVRMFEATWWIFAIIIANSYTANLAAFLTSSKMEGSIANLKDLSAQKKVKFGTIYGGSTYNLLADSNETVYRLAFNLMNNDDPSAYTKDNLEGVDRVRKNRGDYMFLMETTTLEYHREQNCDLRSVGEKFGEKHYAIAVPFGAEYRSNLSVAILKLSERGELYDLKQKWWKNPNASCFEEPDPDATPDMTFEELRGIFYTLYAGILIAFLIGITEFLVYVQQVALEERLTFKDAFKKEIRFVLCVWNNRKPIVAGTPISSVRTTPRRSLDKSLDRTPKSSRRVVIGRSSEEMREMAQGSGSSSGSNNAGRGEKEARV

>DmelGluRIIA

MRLCPVVIYAFIIIIGFLEGIIALGGDDRNEITVGAIFYENEKEIELSFDQAFREVNNMKFSELRFVTIKRYMPTNDSFLLQQITCELISNGVAAIFGPSSKAASDIVAQIANATGIPHIEYDLKLEATRQEQLNHQMSINVAPSLSVLSRAYFEIIKSNYEWRTFTLIYETPEGLARLQDLMNIQALNSDYVKLRNLADYADDYRILWKETDETFHEQRIILDCEPKTLKELLKVSIDFKLQGPFRNWFLTHLDTHNSGLRDIYNEDFKANITSVRLKVVDANPFERKKTRLTKVDQILGNQTMLPILIYDAVVLFASSARNVIAAMQPFHPPNRHCGSSSPWMLGAFIVNEMKTISEDDVEPHFKTENMKLDEYGQRIHFNLEIYKPTVNEPMMVWTPDNGIKKRLLNLELESAGTTQDFSEQRKVYTVVTHYEEPYFMMKEDHENFRGREKYEGYAVDLISKLSELMEFDYEFMIVNGNGKYNPETKQWDGIIRKLIDHHAQIGVCDLTITQMRRSVVDFTVPFMQLGISILHYKSPPEPKNQFAFLEPFAVEVWIYMIFAQLIMTLAFVFIARLSYREWLPPNPAIQDPDELENIWNVNNSTWLMVGSIMQQGCDILPRGPHMRILTGMWWFFALMMLSTYTANLAAFLTSNKWQSSIKSLQDLIEQDKVHFGSMRGGSTSLFFSESNDTDYQRAWNQMKDFNPSAFTSTNKEGVARVRKEKGGYAFLMETTSLTYNIERNCDLTQIGEQIGEKHYGLAVPLGSDYRTNLSVSILQLSERGELQKMKNKWWKNHNVTCDSYHEVDGDELSIIELGGVFLVLAGGVLIGVILGIFEFLWNVQNVAVEERVTPWQAFKAELIFALKFWVRKKPMRISSSSDKSSSRRSSGSRRSSKEKSRSKTVS

>DmelGluRIIB

MHGLQFLVLLALAIASGANEDTLVIKIGAIFFDTEMKLADAFSAALEEVNAINPALKLDAIKRYVTVDDSIVLQDISCDLIGSGVAAIFGPSSKTNSDIVEVLCNMTGIPHLQFDWHPQQSNRERMNHQLTVNVAPMELFLSAAFSDILASKTFDWKSFTIAYERSSHLIRLQHILAWKQLHKAGIKMQEFERGDDYRILWKRINNAREKFVLLDCPSDILVDVINASIGYNMTGSFNHLFLTNLDTHLSGIDGFYSRDFTVAVAAVRIRTYVPPPVHDEIDVFDNSVDTRFSSLGSQLVYDSIVLFYNALLEISQRPGFYIPNFSCGRGFWQPGPRLVEQMKQITPKMVKPPFKTQRLQINADGQREDFNLEVYNPIIDRVTHIWNKEFQLVDFEKLRENSTQALKQKRLQNKEDFSQKPIRYTVATRVGKPYFSWREEPEGVHYEGNERFEGYAVDLIYMLAQECKFDFNFEPVRDNKYGSYDANTDEWDGIIRQLIDNNAQIGICDLTITQARRSVVDFTVPFMQLGISILSYKEPPPKADIYAFLNPYNAEVWLFVMIAMMITAFALIFTGRIDQYEWDQPVENVNREMERQNIWHLSNALWLVLGSMLNQGCDLLPRGLPMRLLTAFWWIFALLISQTYIAKLAAFITSSKIAGDIGSLHDLVDQNKVQFGTIRGGATSVYFSESNDTDNRMAWNKMLSFKPDAFTKNNEEGVDRVKLSKGTYAFLMETTNLQYYVQRNCELTQIGESFGEKHYGIAVPLNADFRSNLSVGILRLSERGELFKLRNKWFNSNESTCDSNVPTIDDGQFDMDSVGGLFVVLIVGVVVGLVIGVAEFLWHVQRISVKEKIPPMLALKAEFYFVIRFWLTRKPLHTYRQSRDSTSTGYSSLEQITSASSAKKKKKTRRIEK

>DmelClumsy

MYSLFLTHFLLIALPVLADIDRSQFMVGSIFTSDKDESEIAFRTAVDRANILERNVELVPIVVYANTDDSFIMEKMVCNLISQGVIAIFGPSTGSSSDIIASICDTLDIPHIVYDWIPNESIPDREHSTMTLNVHPDNLLLSQGLAEIVQSFAWRSFTVVYETDKELQQLQDILQVGEPISNPTTVKQLGPGDDHRPFLKEIKLSTDNCLILHCAPDNLLKILQQANELKMLGEYQSVFIPLLDTHSIDFGELSGVEANITTVRLMDPSDFHVKNVVHDWEEREKREGRYFKVDPNRVKSQMILLNDAVWLFSKGLTELGIFEELTAPDLECRRKKPWPFGKRIIEFIKARSEETSTGRIDFNENGQRSFFTLRFMELNSDGFLDLATWDPVNGLDVLNDDEESEKRVGQKLSNKTFIVSSRLGAPFLTLREPQEGEILTGNSRYEGYSIDLINEIAKMLNFKFEFRMSPDGKYGALNKVTQTWDGIVRQLIDGNADLGICDLTMTSSRRQAVDFTPPFMTLGISILFSKPPTPPTDLFSFLSPFSLDVWIYMGSAYLFISLLLFALARMAPDDWENPHPCKEPEEVENIWSIMNTTWLSIGSLMGQGCDILPKAASTRLVTGMWWFFALMMLNSYTANLAAFLTNSRQANSINSAEDLAAQSKIKYGAMAGGSTMGFFRDSNFSTYQKMWTAMESASPSVFTKTNDEGVERVQKGKNLYAFLMESTTLEYNVERKCDLVQIGGWLDYKSYGIAMPFNSPYRKQISAAVLKLGELGQLAELKRKWWKEMHGGGNCEKSDEDGGDTPELGLENVGGVFLVLGLGLLSAMVLGCTEFLWNVKSVAIEEKISLKEAFKSEALFAARIWITTKPVHTSSESGSSNSSSSSSSRSKHSFKSQGLSMKSLKSSGYQDVEASVHSKLKKIGSMFSLKSQKTVTPPPEIGWKLDKSTQIDVVPTSDVDQELIPEVEPHLPHRHHHHHHHRHHHHHHQPDQEHDRNPSPPE

>DmelGluRIID

MHFCWISLIILSLSRVQAQFYGGNAYEASSGQSIRLGLITDDATDRIRQTFEHAISVVNNELGVPLVGETEQVAYGNSVQAFAQLCRLMQSGVGAVFGPAARHTASHLLNACDSKDIPFIYPHLSWGSNPDGFNLHPSPEDIANALYDIVNQFEWSRFIFCYESAEYLKILDHLMTRYGIKGPVIKVMRYDLNLNGNYKSVLRRIRKSEDSRIVVVGSTTGVAELLRQAQQVGIMNEDYTYIIGNLNLHTFDLEEYKYSEANITGIRMFSPDQEEVRDLMEKLHQELGESEPVNSGSTFITMEMALTYDAVRVIAETTKHLPYQPQMLNCSERHDNVQPDGSTFRNYMRSLEIKEKTITGRIYFEGNVRKGFTFDVIELQTSGLVKVGTWEEGKDFEFQRPPQAVNFNDIDDGSLVNKTFIVLISVATKPYASLVESIDTLIGNNQFQGYGVDLIKELADKLGFNFTFRDGGNDYGSFNKTTNSTSGMLKEIVEGRADLAITDLTITSEREEVIDFSIPFMNLGIAILYVKPQKAPPALFSFMDPFSSEVWLYLGIAYLGVSLCFFIIGRLSPIEWDNPYPCIEEPEELENQFTINNSLWFTTGALLQQGSEIAPKALSTRTISAIWWFFTLIMVSSYTANLAAFLTIENPTSPINSVKDLADNKDDVQYGAKRTGSTRNFFSTSEEPIYIKMNEYLNAHPEMLMENNQQGVDKVKSGTKYAFLMESTSIEFNTVRECNLTKVGDPLDEKGYGIAMVKNWPYRDKFNKALLELQEQGVLARLKNKWWNEVGAGVCSAKSDDDGPSELGVDNLSGIYVVLVIGSIISIIISILCWCYFVYKKAKNYEVPFCDALAEEFRIVIRFSENERPLKSAQSIYSRSRNSSQSIESLKTDSEENMPVED

>DmelGluRIIE

MFFNHFVILWSLFSIHISVNWAQYENFGGYDNYQSLESVPIGLLTDQNTEQMNIVFDHAIDVANQEVGTSLTSLKEEVNYGDAYQSYGKLCRMLETGIAGVFGPSSRHTAVHLMSICDAMDIPHIYSYMSENAEGFNLHPHPADLAKALYSLITEFNWTRFIFLYESAEYLNILNELTTMLGKSGTVITVLRYDMQLNGNYKQVLRRVRKSVDNRIVVVGSSETMPEFLNQAQQVGIINEDYKYIIGNLDFHSFDLEEYKYSEANITGLRLFSPEKMAVKELLMKLGYPTDQDEFRNGSCPITVEMALTYDAVQLFAQTLKNLPFKPMPQNCSQRTESVRDDGSSFKNYMRTLRLTDRLLTGPIYFEGNVRKGYHLDVIELQPSGIVKVGTWDEDRQYRPQRLAPTTAQFDSVDNSLANKTFIILLSVPNKPYAQLVETYKQLEGNSQYEGYGVDLIKELADKLGFNFTFVNGGNDYGSYNKSTNESTGMLREIMTGRADLAITDLTITSEREQALDFTIPFMNLGIAILYLKPQKATPELFTFMDPFSEEVWWFLGFSFLGVSLSFFILGRLSPSEWDNPYPCIEEPEELENQFTLGNSIWFTTGALLQQGSEIGPKALSTRTVASFWWFFTLIVVSSYTANLAAFLTIEKPQSLINSVDDLADNKDGVVYGAKKTGSTRNFFMTSAEERYKKMNKFMSENPQYLTEDNMEGVNRVKTNTHYAFLMESTSIEYNTKRECNLKKIGDALDEKGYGIAMRKDWPHRGKFNNALLELQEQGVLEKMKNKWWNEVGTGICATKEDAPDATPLDMNNLEGVFFVLLVGSCCALLYGIISWVLFVMKKAHHYRVPLRDALKEEFQFVIDFNNYVRVLKNSASIYSRSRQSSMSVASVAQESQ

>DmelCG3822

MRSSGVLVLPLLLLQLILNCRKAQSLPDIIKIGGLFHPADDHQELAFRQAVDRINADRSILPRSKLVAQIERISPFDSFHAGKRVCGLLNIGVAAIFGPQSSHTASHVQSICDNMEIPHLENRWDYRLRRESCLVNLYPHPNTLSKAYVDIVRHWGWKTFTIIYENNDGIVRLQELLKAHGMTPFPITVRQLSDSGDYRPLLKQIKNSAEAHIVLDCSTERIHEVLKQAQQIGMMSDYHSYLVTSLDLHTVNLDEFRYGGTNITGFRLINEKIVSDVVRQWSIDEKGLLRSANLTTVRSETALMYDAVHLFAKALHDLDTSQQIDIHPISCDGQSTWQHGFSLINYMKIVEMKGLTNVIKFDHQGFRTDFMLDIVELTPAGIRKIGTWNSTLPDGINFTRTFSQKQQEIEANLKNKTLVVTTILSNPYCMRKESAIPLSGNDQFEGYAVDLIHEISKSLGFNYKIQLVPDGSYGSLNKLTGEWNGMIRELLEQRADLAIADLTITFEREQAVDFTTPFMNLGVSILYRKPIKQPPNLFSFLSPLSLDVWIYMATAYLGVSVLLFILAKFTPYEWPAYTDAHGEKVESQFTLLNCMWFAIGSLMQQGCDFLPKALSTRMVAGIWWFFTLIMISSYTANLAAFLTVERMDSPIESAEDLAKQTRIKYGALKGGSTAAFFRDSKISTYQRMWSFMESARPSVFTASNGEGVERVAKGKGSYAFLMESTSIEYVTERNCELTQVGGMLDTKSYGIATPPNSPYRTAINSVILKLQEEGKLHILKTKWWKEKRGGGKCRVETSKSSSAANELGLANVGGVFVVLMGGMGVACVIAVCEFVWKSRKVAVEERLSAILNE

>DmelCG5621

MISTEASFPLGFILTSLLLAFPGCRGERTNVGLVYENTDPDLEKIFHLAISKANEENEDLQLHGVSVSIEPGNSFETSKKLCKMLRQNLVAVFGPTSNLAARHAMSICDAKELPFLDTRWDFGAQLPTINLHPHPATLGVALRDMVVALGWESFTIIYESGEYLPTVRELLQMYGTAGPTVTVRRYELDLNGNYRNVLRRIRNADDFSFVVVGSMATLPEFFKQAQQVGLVTSDYRYIIGNLDWHTMDLEPYQHAGTNITGLRLVSPDSEQVQEVAKALYESEEPFQNVSCPLTNSMALVYDGVQLLAETYKHVNFRPVALSCNDDSAWDKGYTLVNYMKSLTLNGLTGPIRFDYEGLRTDFKLEVIELAVSGMQKIGQWSGEDGFQENRPAPAHSLEPDMRSLVNKSFVVITAISEPYGMLKETSEKLEGNDQFEGFGIELIDELSKKLGFSYTWRLQEDNKYGGIDPKTGEWNGMLREIIDSRADMGITDLTMTSERESGVDFTIPFMSLGIGILFRKPMKEPPKLFSFMSPFSGEVWLWLGLAYMGVSISMFVLGRLSPAEWDNPYPCIEEPTELENQFSFANCLWFSIGALLQQGSELAPKAYSTRAVAASWWFFTLILVSSYTANLAAFLTVESLVTPINDADDLSKNKGGVNYGAKIGGATFNFFKESNYPTYQRMYEFMRDNPQYMTNTNQEGVDRVENSNYAFLMESTTIEYITERRCTLTQVGALLDEKGYGIAMRKNWPYRDTLSQAVLEMQEQGLLTKMKTKWWQEKRGGGACSDADEDSGAVALEISNLGGVFLVMGVGSFFGIFVSLLEMVLGVKERSDENQEAPDSDASSLGFANLGGVYLVMFVGSCFGSIYGLVNCVVSVYLRARENKVSFKTELLDEIRFILQCSGNTKAVKYPKNSSRSNASSKSKGSSMSVDSLPEDTSEADASGKHNHGKK

>DmelCG9935

MLIASGFLLFQFLSYGLGVPPLVRIGAIFSNQPGMYNSELAFRYAIHRLNMDKSLLPETTVDYYVEYVNRFDSFETVQKVCKLIRVGVQAVFSPTDSVLATHINSICDALDIPNIGRSAHDFSINVYPSKQLVNYAFNDVIQYLNWTRFGILHEKENGIINLHQLSRSFHGEVHMRQVSRDSYVSALNEFKGKEIHNIIIDTNSNGISILLKNILQQQMNEYKYHYLFTSFDLETYDLEDFKYNFVNITSFRLVDTADVGVKQILKDIGLYSHHIFKKPYLNLHIKKSTILESEPALMFDSVYVFAIGLQTLEQSHSLTLLNISCEEENSWDGGLSLINYLNAVEWKGLTGPIQFKDGQRVQFKLDLIKLKQHSIVKVGEWTPHGHLNITEPSMFFDAGSMNVTLVVITILETPYVMMHYGKNFTGNERFYGFCVDILETISREVGFDYILDLVPDRKYGAKDPETGEWNGMVAQLMKYKADLAVGSMTITYARESVIDFTKPFMNLGISILFKVPTSEPTRLFSFMNPLAIEIWIYVLIAYFLVSLCIYIVGKLSPIEWKCINACDLENISIGNQFSLTDSFWFTIGTFMQQSPDIYPRAMSTRIISSTWGFFSLIIVASYTANLAAFLTTERMINPIENAEDLASQTEISYGTLDSGSTMTFFRDSVIETYKKIWRSMDNKKPSAFTTTYEDGIKRVNQGNYAFLMESTMLDYIVQRDCNLTQIGGLLDTKGYGIATPKGSPWRDKISLAILELQERGDIQMLYDKWWKNTDETCTRKNTSKQSKANSLGLESIGGVFVVLIAGIIVAAVVAFFEFWYNFRYNYEATPSQSVVNNKYNQDGILESERNYTPPDRSFWIEIAEELRYASWCMNKQKRPALTRTCSKCTIPKGQRINKL

>DmelCG11155

MVRKKREIVIKENIQGRSYLKKICCSYIILSILVISNALPPVIRVGAIFTEDERESSIESAFKYAIYRINKEKTLLPNTQLVYDIEYVPRDDSFRTTKKVCSQLEAGVQAIFGPTDALLASHVQSICEAYDIPHIEGRIDLEYNSKEFSINLYPSHTLLTLAYRDIMVYLNWTKVAIIYEEDYGLFNLMHSSTETKAEMYIRQASPDSYRQVLRAIRQKEIYKIIVDTNPSHIKSFFRSILQLQMNDHRYHYMFTTFDLETYDLEDFRYNSVNITAFRLVDVDSKRYLEVINQMQKLQHNGLDTINGSPYIQTESALMFDSVYAFANGLHFLNLDNHQNFYIKNLSCTSDQTWNDGISLYNQINAAITDGLTGTVQFVEGRRNIFKLDILKLKQEKIQKVGYWHPDDGVNISDPTAFYDSNIANITLVVMTREERPYVMVKEDKNLTGNLRFEGFCIDLLKAIATQVGFQYKIELVPDNMYGVYIPETNSWNGIVQELMERRADLAVASMTINYARESVIDFTKPFMNLGIGILFKVPTSQPTRLFSFMNPLAIEIWLYVLAAYILVSFALFVMARFSPYEWKNPHPCYKETDIVENQFSISNSFWFITGTFLRQGSGLNPKATSTRIVGGCWFFFCLIIISSYTANLAAFLTVERMISPIESASDLAEQTEISYGTLEGGSTMTFFRDSKIGIYQKMWRYMENRKTAVFVKTYEDGIKRVMEGSYAFLMESTMLDYAVQRDCNLTQIGGLLDSKGYGIATPKGSPWRDKISLAILELQEKGIIQILYDKWWKNTGDVCNRDDKSKESKANALGVENIGGVFVVLLCGLALAVVVAIFEFCWNSRKNLNTENQSLCSEMAEELRFAMHCHGSKSRHRPRKRSCLNCSSVPTYVPSNVSTSNVGVYYNYFN

>DmelGluR1

MHSRLKFLAYLHFICASSIFWPEFSSAQQQQQTVSLTEKIPLGAIFEQGTDDVQSAFKYAMLNHNLNVSSRRFELQAYVDVINTADAFKLSRLICNQFSRGVYSMLGAVSPDSFDTLHSYSNTFQMPFVTPWFPEKVLAPSSGLLDFAISMRPDYHQAIIDTIQYYGWQSIIYLYDSHDGLLRLQQIYQELKPGNETFRVQMVKRIANVTMAIEFLHTLEDLGRFSKKRIVLDCPAEMAKEIIVQHVRDIKLGRRTYHYLLSGLVMDNHWPSDVVEFGAINITGFRIVDSNRRAVRDFHDSRKRLEPSGQSQSQNAGGPNSLPAISAQAALMYDAVFVLVEAFNRILRKKPDQFRSNHLQRRSHGGSSSSSATGTNESSALLDCNTSKGWVTPWEQGEKISRVLRKVEIDGLSGEIRFDEDGRRINYTLHVVEMSVNSTLQQVAEWRDDAGLLPLHSHNYASSSRSASASTGDYDRNHTYIVSSLLEEPYLSLKQYTYGESLVGNDRFEGYCKDLADMLAAQLGIKYEIRLVQDGNYGAENQYAPGGWDGMVGELIRKEADIAISAMTITAERERVIDFSKPFMTLGISIMIKKPVKQTPGVFSFLNPLSQEIWISVILSYVGVSFVLYFVTRFPPYEWRIVRRPQADSTAQQPPGIIGGATLSEPQAHVPPVPPNEFTMLNSFWYSLAAFMQQGCDITPPSIAGRIAAAVWWFFTIILISSYTANLAAFLTVERMVAPIKTPEDLTMQTDVNYGTLLYGSTWEFFRRSQIGLHNKMWEYMNANQHHSVHTYDEGIRRVRQSKGKYALLVESPKNEYVNARPPCDTMKVGRNIDTKGFGVATPIGSPLRKRLNEAVLTLKENGELLRIRNKWWFDKTECNLDQETSTPNELSLSNVAGIYYILIGGLLLAVIVAIMEFFCRNKTPQLKSPGSNGSAGGVPGMLASSTYQRDSLSDAIMHSQAKLAMQASSEYDERLVGVELASNVRYQYSM

>DmelGluR1B

MRFGLKLSCLWPSFLLWLTWSSGGGGGSGVGVSAQPSLTEKIPLGAIFEQGTDEVQSAFKYAMLNHNLNVSSRRFELQAYVDVINTADAFKLSRLICNQFSRGVYSMLGAVSPDSFDTLHSYSNTFQMPFVTPWFPEKVLTPSSGFLDFALSMRPDYHQAIIDTIQFYGWRKIIYLYDSHDGLLRLQQIYQGLRPGNESFQVELVKRISNVSMAIEFLHTLEQIGRFENKHIVLDCPTEMAKQILIQHVRDLRLGRRTYHYLLSGLVMDDRWESEIIEFGAINITGFRIVDTNRRLVREFYDSWKRLDPQMSVGAGRESISAQAALMYDAVFVLVEAFNKILRKKPDQFRNNVQRRSQTLMVAQAAASTSSDGYNYSASGGGGGNGGAGGGFAGSDSGGSGGMASRALDCNTAKGWVNAWEHGDKISRYLRKVEIEGLTGDIKFNDDGRRVNYTLHVVEMTVNSAMVKVAEWNDDAGLQPLNAKYVRLRPHVEFEKNRTYIVTTVLEEPYIMLKQVAFGEKLHGNNRFEGYCKDLADLLAKELGINYELRLVKDGNYGSEKSSAHGGWDGMVGELVRKEADIAIAAMTITAERERVIDFSKPFMSLGISIMIKKPVKQTPGVFSFMNPLSQEIWVSVIFSYIGVSIVLFFVSRFSPHEWRLVQQQPQQSQSPDPHAHHEQLANQQPPGIIGGAPLPAPPGPPTPGAQTAAGAAALQAALSAGSPGSGGSSSAVVNEFSVWNSFWFSLAAFMQQGCDLSPRSVSGRIAAASWFFFTLILISSYTANLAAFLTVERMVTPINSPEDLAMQTEVQYGTLLHGSTWDFFRRSQIGLHNKMWEYMNSRKHVFVPTYDEGIKRVRNSKGKYALLVESPKNEYVNAREPCDTMKVGRNLDTKGFGIATPLGSALKDPINLAVLTLKENGELIKLRNKWWYEKAECSTHKDGETSHSELSLSNVAGIFYILIGGLLVSVFVAILEYCFRSRDSRSASSGSGMGLGMGLGGGMSGGSLGKANGSMMLGPSSAVPGGMPSSHQRSTLTDTMHAKAKLTIQASRDYDNGRVGYLNCASLQYYPPAQLSATPPDAGDSLHMNAHGQV

>DmelNmdar2

MMPSRVKLKRGTDGPTPTPTPMPTTMRKHTPIATLNTASCQHNSTTSRRKRILTPPSGPISLLLLTVLTLLILDTRSCQGLRLTNGGGSLSKGAAANKEQLNIGLIAPHTNFGKREYLRSINNAVTGLTKTRGAKLTFLKDYSFEQKNIHFDMMSLTPSPTAILSTLCKEFLRVNVSAILYMMNNEQFGHSTASAQYFLQLAGYLGIPVISWNADNSGLERRASQSTLQLQLAPSIEHQSAAMLSILERYKWHQFSVVTSQIAGHDDFVQAVRERVAEMQEHFKFTILNSIVVTRTSDLMELVNSEARVMLLYATQTEAITILRAAEEMKLTGENYVWVVSQSVIEKKDAHSQFPVGMLGVHFDTSSAALMNEISNAIKIYSYGVEAYLTDPANRDRRLTTQSLSCEDEGRGRWDNGEIFFKYLRNVSIEGDLNKPNIEFTADGDLRSAELKIMNLRPSANNKNLVWEEIGVWKSWETQKLDIRDIAWPGNSHAPPQGVPEKFHLKITFLEEAPYINLSPADPVSGKCLMDRGVLCRVAADHEMAADIDVGQAHRNESFYQCCSGFCIDLLEKFAEELGFTYELVRVEDGKWGTLENGKWNGLIADLVNRKTDMVLTSLMINTEREAVVDFSEPFMETGIAIVVAKRTGIISPTAFLEPFDTASWMLVGIVAIQAATFMIFLFEWLSPSGYDMKLYLQNTNVTPYRFSLFRTYWLVWAVLFQAAVHVDSPRGFTSRFMTNVWALFAVVFLAIYTANLAAFMITREEFHEFSGLNDSRLVHPFSHKPSFKFGTIPYSHTDSTIHKYFNVMHNYMRQYNKTSVADGVAAVLNGNLDSFIYDGTVLDYLVAQDEDCRLMTVGSWYAMTGYGLAFSRNSKYVQMFNKRLLEFRANGDLERLRRYWMTGTCRPGKQEHKSSDPLALEQFLSAFLLLMAGILLAALLLLLEHVYFKYIRKRLAKKDGGHCCALISLSMGKSLTFRGAVFEATEILKKHRCNDPICDTHLWKVKHELDMSRLRVRQLEKVMDKHGIKAPQLRLASSSDLLNHHHLKERPPLLGNLSLAASAQDLYRWSYKTEIAEMETVL

>DmelNmdar1

MAMAEFVFCRPLFGLAIVLLVAPIDAAQRHTASDNPSTYNIGGVLSNSDSEEHFSTTIKHLNFDQQYVPRKVTYYDKTIRMDKNPIKTVFNVCDKLIENRVYAVVVSHEQTSGDLSPAAVSYTSGFYSIPVIGISSRDAAFSDKNIHVSFLRTVPPYYHQADVWLEMLSHFAYTKVIIIHSSDTDGRAILGRFQTTSQTYYDDVDVRATVELIVEFEPKLESFTEHLIDMKTAQSRVYLMYASTEDAQVIFRDAGEYNMTGEGHVWIVTEQALFSNNTPDGVLGLQLEHAHSDKGHIRDSVYVLASAIKEMISNETIAEAPKDCGDSAVNWESGKRLFQYLKSRNITGETGQVAFDDNGDRIYAGYDVINIREQQKKHVVGKFSYDSMRAKMRMRINDSEIIWPGKQRRKPEGIMIPTHLRLLTIEEKPFVYVRRMGDDEFRCEPDERPCPLFNNSDATANEFCCRGYCIDLLIELSKRINFTYDLALSPDGQFGHYILRNNTGAMTLRKEWTGLIGELVNERADMIVAPLTINPERAEYIEFSKPFKYQGITILEKKPSRSSTLVSFLQPFSNTLWILVMVSVHVVALVLYLLDRFSPFGRFKLSHSDSNEEKALNLSSAVWFAWGVLLNSGIGEGTPRSFSARVLGMVWAGFAMIIVASYTANLAAFLVLERPKTKLSGINDARLRNTMENLTCATVKGSSVDMYFRRQVELSNMYRTMEANNYATAEQAIQDVKKGKLMAFIWDSSRLEYEASKDCELVTAGELFGRSGYGIGLQKGSPWTDAVTLAILEFHESGFMEKLDKQWIFHGHVQQNCELFEKTPNTLGLKNMAGVFILVGVGIAGGVGLIIIEVIYKKHQVKKQKRLDIARHAADKWRGTIEKRKTIRASLAMQRQYNVGLNSTHAPGTISLAVDKRRYPRLGQRLGPERAWPGDAADVLRIRRPYELGNPGQSPKVMAANQPGMPMPMLGKTRPQQSVLPPRYSPGYTSDVSHLVV

>DmelIR8a

MELPLLVLLLALRFAGSEVLKITFWIEPVQRAEFDTDIAMVLKELDALRLDVKVDDTTLTLTRSEDGLDMQRFCEILSTVGASAVIDLTYSHWEEGYNLVRSLGIGYVRLERIMRPFLDMFGDFMRQKRANNVAMVFMNARDAVEAMQQMLVGYPFRTLIMDASQTDPGQHFLERIRSLRPAPTYIALFARAAAMNGIFEKVQKADLFQRPLEWHFVFLDTRDRVFKYRRQAELCTRFTLNPRAICRSMPMPDLYCGSGFTMQRAMLLNVLRSLINAAQVSPGYPLAIYQDCNATASSSEVSDPLEKDDYNWLDMVHWSNFLAYAPPLPHIQDQFQSPVPGLTFAVNISAGYYSSEHEAKTDLAAWSSVGEMRLLNETISPARRFFRIGTAESIPWSYLRREEGTGELIRDRSGLPIWEGYCIDFIIRLSQKLNFEFEIVAPEVGHMGELNELGEWDGVVGDLVRGETDFAIAALKMYSEREEVIDFLPPYYEQTGISIAIRKPVRRTSLFKFMTVLRLEVWLSIVAALVGTAIMIWFMDKYSPYSSRNNRQAYPYACREFTLRESFWFALTSFTPQGGGEAPKAISGRMLVAAYWLFVVLMLATFTANLAAFLTVERMQTPVQSLEQLARQSRINYTVVKDSDTHQYFVNMKFAEDTLYRMWKELALNASKDFKKFRIWDYPIKEQYGHILLAINSSQPVADAKEGFANVDAHENADYAFIHDSAEIKYEITRNCNLTEVGEVFAEQPYAVAVQQGSHLGDELSYAILELQKDRFFEELKAKYWNQSNLPNCPLSEDQEGITLESLGGVFIATLFGLVLAMMTLGMEVLYYKKKQNALEITQVRPVNDSSGSGGNSSTAPPTATSTTKQAWHIPVLEAEEKPAKVSPPPSFETATFRGKKLPARITLGDGKFKPRHGLYARRNLGASDSHSGYME

>DmelIR25a

MILMNPKTSKILWLLGFLSLLSSFSLEIAAQTTQNINVLFINEVDNEPAAKAVEVVLTYLKKNIRYGLSVQLDSIEANKSDAKVLLEAICNKYATSIEKKQTPHLILDTTKSGIASETVKSFTQALGLPTISASYGQQGDLRQWRDLDEAKQKYLLQVMPPADIIPEAIRSIVIHMNITNAAILYDDSFVMDHKYKSLLQNIQTRHVITAIAKDGKREREEQIEKLRNLDINNFFILGTLQSIRMVLESVKPAYFERNFAWHAITQNEGEISSQRDNATIMFMKPMAYTQYRDRLGLLRTTYNLNEEPQLSSAFYFDLALRSFLTIKEMLQSGAWPKDMEYLNCDDFQGGNTPQRNLDLRDYFTKITEPTSYGTFDLVTQSTQPFNGHSFMKFEMDINVLQIRGGSSVNSKSIGKWISGLNSELIVKDEEQMKNLTADTVYRIFTVVQAPFIMRDETAPKGYKGYCIDLINEIAAIVHFDYTIQEVEDGKFGNMDENGQWNGIVKKLMDKQADIGLGSMSVMAEREIVIDFTVPYYDLVGITIMMQRPSSPSSLFKFLTVLETNVWLCILAAYFFTSFLMWIFDRWSPYSYQNNREKYKDDEEKREFNLKECLWFCMTSLTPQGGGEAPKNLSGRLVAATWWLFGFIIIASYTANLAAFLTVSRLDTPVESLDDLAKQYKILYAPLNGSSAMTYFERMSNIEQMFYEIWKDLSLNDSLTAVERSKLAVWDYPVSDKYTKMWQAMQEAKLPATLDEAVARVRNSTAATGFAFLGDATDIRYLQLTNCDLQVVGEEFSRKPYAIAVQQGSHLKDQFNNAILTLLNKRQLEKLKEKWWKNDEALAKCDKPEDQSDGISIQNIGGVFIVIFVGIGMACITLVFEYWWYRYRKNPRIIDVAEANAERSNAADHPGKLVDGVILGHSGEKFEKSKAALRPRFNQYPATFKPRF

>DmelIR21a

MSYYWVALVLFTAQAFSIEGDRSASYQEKCISRRLINHYQLNKEIFGVGMCDGNNENEFRQKRRIVPTFQGNPRPRGELLASKFHVNSYNFEQTNSLVGLVNKIAQEYLNKCPPVIYYDSFVEKSDGLILENLFKTIPITFYHGEINADYEAKNKRFTSHIDCNCKSYILFLSDPLMTRKILGPQTESRVVLVSRSTQWRLRDFLSSELSSNIVNLLVIGESLMADPMRERPYVLYTHKLYADGLGSNTPVVLTSWIKGALSRPHINLFPSKFQFGFAGHRFQISAANQPPFIFRIRTLDSSGMGQLRWDGVEFRLLTMISKRLNFSIDITETPTRSNTRGVVDTIQEQIIERTVDIGMSGIYITQERLMDSAMSVGHSPDCAAFITLASKALPKYRAIMGPFQWPVWVALICVYLGGIFPIVFTDRLTLSHLMGNWGEVENMFWYVFGMFTNAFSFTGKYSWSNTRKNSTRLLIGAYWLFTIIITSCYTGSIIAFVTLPAFPDTVDSVLDLLGLFFRVGTLNNGGWETWFQNSTHIPTSRLYKKMEFVGSVDEGIGNVTQSFFWNYAFLGSKAQLEYLVQSNFSDENISRRSALHLSEECFALFQIGFLFPRESVYKIKIDSMILLAQQSGLIAKINNEVSWVMQRSSSGRLLQASSSNSLREIIQEERQLTTADTEGMFLLMALGYFLGATALVSEIVGGITNKCRQIIKRSRKSAASSWSSASSGSMLRTNAEQLSHDKRKANRREAAEVAQKMSFGMRELNLTRATLREIYGSYGAPETDHGQLDIVHTEFPNSSAKLNNIEDEESREALESLQRLDEFMDQMDNDGNPSSHTFRIDN

>DmelIR31a

MNLLISMFILILAAGEGEIIPSMEESVVTNFVKSLVKTKQAIVFSCLFKDFKEISLALMRINQFVSVVNLNQSYSLTSILTRENYARTSVMVNARCSGSSELLFEASENRYFNKTYQWFLWGVDLEVQSLFPLNLNYVGPNAQITYVNETADGYAYWDIHSKGRHLKSNLEINLIATLINDTLNIARDIFHLQSIDFRGQFNGLTLRGASVIDKEDIISNEQIESILSRPTKDAGVAAFIKYHYELLGLLRERFNFTVNFRNSRGWAGRLGNTTFRLGLLGIVMRNEADIAASGAFNRINRFAEFDTIHQSWKFETAFLYRYTSDLDTHGKSGNFLSPFSDRVWLFCLLTLGAFSIIWVLFEIIDYKILRIRVNSQKLEHLNQKSSVICIKTTCIERILQTFGACCQQGLDPNPVDRSVRFLVMTLFLFSLVMYNYYTSSVVGGLLSSSDQGPSTVDEITASPLKISFEDIGYYKVLFRESQNRSITRLIEKKLSSSRSLNELPIFSHIEDAVPYLKAGGFAFHCEVVDAYPVISEYFDANEICDLREVSGLMEVEILNWILHKNSQYTEIFKTAMCNAQEKGFVERILRRRQIKKPACQSLYTVYPVSLSGVLPGFVILICKSINKFS

>DmelIR40a

MHKFLALGLLPYLLGLLNSTRLTFIGNDESDTAIALTQIVRGLQQSSLAILALPSLALSDGVCQKERNVYLDDFLQRLHRSNYKSVVFSQTELFFQHIEENLQGANECISLILDEPNQLLNSLHDRHLGHRLSLFIFYWGARWPPSSRVIRFREPLRVVVVTRPRKKAFRIYYNQARPCSDSQLQLVNWYDGDNLGLQRIPLLPTALSVYANFKGRTFRVPVFHSPPWFWVTYCNNSFEEDEEFNSLDSIEKRKVRVTGGRDHRLLMLLSKHMNFRFKYIEAPGRTQGSMRSEDGKDSNDSFTGGIGLLQSGQQADFFLGDVGLSWERRKAIEFSFFTLADSGAFATHAPRRLNEALAIMRPFKQDIWPHLILTIIFSGPIFYGIIALPYIWRRRWANSDVEHLGELYIHMTYLKEITPRLLKLKPRTVLSAHQMPHQLFQKCIWFTLRLFLKQSCNELHNGYRAKFLTIVYWIAATYVLADVYSAQLTSQFARPAREPPINTLQRLQAAMIHDGYRLYVEKESSSLEMLENGTELFRQLYALMRQQVINDPQGFFIDSVEAGIKLIAEGGEDKAVLGGRETLFFNVQQYGSNNFQLSQKLYTRYSAVAVQIGCPFLGSLNNVLMQLFESGILDKMTAAEYAKQYQEVEATRIYKGSVQAKNSEAYSRTESYDSTVISPLNLRMLQGAFIALGVGSLAAAALNNTINVRSLNSRDKFICGGPVKIWYYLVLLLWYYFNRGLVGIYQLWHKTSIRNTGKGMPFLGE

>DmelIR64a

MHWWLLVFLPLSCQGLPEHELLELELDYGLAEPQRTSLLQSSLILQFSQDYKHIPRITYFTCQKPHLQTPNQIPNAAEHRDAFAAKNFQLIKSLYESELFVRIVLLDVLAQSPTSGRPNRPGNGPTGGFSQTPSQAQSNSEWLEGVLRMEALRQIAVVDLACGAVSRRFLELASAKMLYSEKFHWLLIEDFAWHGRTQTAEGSGKRDDGEMEEEEPPGQQIQATDDEDLPSIESFLGGMNLYMNTELTLAKRMSEAAHYTLFDVWNPGLNYGGHVNLTEIGSFTPTEGIQLHTWFRTTSTVRRRMDMQHARVRCMVVVTNKNMTGTLMYYLTHTMSGHIDTMNRFNFNLLMAVRDMFNWTFVLSRTTSWGYVKNGRFDGMIGALIRNETDIGGAPIFYWLERHKWIDVAGRSWSSRPCFIFRHPRSTQKDRIVFLQPFTNDVWILIVGCGVLTVFILWFLTTIEWKLVPHDGSALIKPKGGAPPRHHYQQQQQQEQVEAPVRPITAVSVVVSKEKVEEKQEEYEDSTPIDAGTLWQRCYQKLNKYIKDRKAKQKKAPERVGLFLESVLFFVGIICQQGLGFSTSFVSGRCIVITSLLFSFCIYQFYSASIVGTLLMEKPKTIKTLSDLVHSSLKVGMEDILYNRDYFLHTKDPVSMELYAKKITSVPTTKENEADEDEPVDPNPVSTDPAKSYRDIVHSHETGAHAKDNAASNWLDPETGLLRVKHERFAFHVDVAAAYKIIAETFSEQDICDLTEVSMFPPQKTVSIMQKNSPMRKVISYGLRRVTETGILTYHFNVWHSRKPPCVKKIETSDLHVDMDTVSSALLILLFSYAITLMILGTEILYSKWHNRIQLKWVGAT

>DmelIR75a

MQLVQLANFVLDNLVQSRIGFIVLFHCWQSDESLKFAQQFMKPIHPILVYHQFVQMRGVLNWSHLELSYMGHTQPTLAIYVDIKCDQTQDLLEEASREQIYNQHYHWLLVGNQSKLEFYDLFGLFNISIDADVSYVKEQIQDNNDSVAYAVHDVYNNGKIIGGQLNVTGSHEMSCDPFVCRRTRHLSSLQKRSKYGNREQLTDVVLRVATVVTQRPLTLSDDELIRFLSQENDTHIDSLARFGFHLTLILRDLLHCKMKFIFSDSWSKSDVVGGSVGAVVDQTADLTATPSLATEGRLKYLSAIIETGFFRSVCIFRTPHNAGLRGDVFLQPFSPLVWYLFGGVLSLIGVLLWITFYMECKRMQKRWRLDYLPSLLSTFLISFGAACIQSSSLIPRSAGGRLIYFALFLISFIMYNYYTSVVVSSLLSSPVKSKIKTMRQLAESSLTVGLEPLPFTKSYLNYSRLPEIHLFIKRKIESQTQNPELWLPAEQGVLRVRDNPGYVYVFETSSGYAYVERYFTAQEICDLNEVLFRPEQLFYTHLHRNSTYKELFRLRFLRILETGVYRKQRSYWVHMKLHCVAQNFVITVGMEYVAPLLLMLICADILVVVILLVELAWKRFFTRHLTFHP

>DmelIR75b

MNFSVLESHFKEAQIFVDADVTYVTHDPFSKNFLLYDVYNKGRQLGGELNITADREIFCNKTNCRVERYLSELYTRSALQHRKSFTGLTMRATAVVTALPLNVSIKEIFDFMNSKYRIQLDTYARLGYQARQPLRDMLDCKFKYIFRDRWSDGNATGGMIGDLILDKADLAIAPFIYSFDRALFLQPITKFSVFREICMFRNPRSVSAGLSATEFLQPFSGGVWLTFALLLLLAGCLLWVTFILERRKQWKPSLLTSCLLSFGAGCIQGAWLTPRSMGGRMAFFALMVTSYLMYNYYTSIVVSKLLGQPIKSNIRTLQQLADSNLDVGIEPTVYTRIYVETSEEPDVRDLYRKKVLGSKRSPDKIWIPTEAGVLSVRDQEGFVYITGVATGYEFVRKHFLAHQICELNEIPLRDASHTHTVLAKRSPYAELIKLSELRMLETGVHFKHERSWMETKLHCYQHNHTVAVGLEYAAPLFIILLGAIILCMGILGLEVIWHRHCTLH

>DmelIR75c

MTSWPLYRLIVFNLLEINLSNLMVFHCWSIKEAFPLVEMLNQNGIFSQYIDVQNPDNLANVHKEYLDSDLVSLNADVTYVSREDEERFILHDVYNKGSHLGGKLNITVDQTLQCNRSHCQVKEYLSELHLRPRLQHRMDLSSVTFRLAALVSVLPINSSEEELLEFLNSDRDSHMDSISRIGNRLIMHTQEILGFNVQDAFGGAIGMLTNESAELCTTPFVPSWNRLHYLHPMTEQAQFRAVCMFRTPHNAGIKAAVFLEPFMPSVWFAFAGLLIFAGVLLWMIFHLERHWMQRCLDFIPSLLSSCLISFGAACIQGSYLMPKSAGGRLAFIAVMLTSFLMYNYYTSIVVSTLLGSPVRSNIRTIQQLADSSLDVGFDTVPFTKTYLVSSPRPDIRSLYKQKVESKRDPNSVWLSPEEGVIRVRDQPGFVYTSEASFMYHFVEKHYLPREISDLNEIILRPESAVYGMVHLNSTYRQLLTQLQVRMLETGITSKQSRFFSKTKLHTFSNSFVIQVGMEYAAPLFISLLVAYFLALLILILEICWARYAKKKFSTIIPQNQ

>DmelIR75d

MKVQVAHWLPLIFFLLVSGTPRVAGSWRSEYSRQDPDPKTRWGNQLPDMLVAYYRHHGVHSLMLVVCHTDIADFRLWKLWQHFNLNNFYVQVSTESSLRDLQHVDALDEHKDAPPPKSFHANNSTHWETSFLLPALPYKMGILLLEFSSECALNLLRWSAASEHNYFTTNRFWLLLTEDPGDIDLLEDPEIFIPPDSELRVLHYENVGNFSCSLIDLYKVAAWKPLKRTLVGHNIRNSRHVIHALQHFGSAITYRQDLEGIVFNSAIVIAFPDLFTNIEDLSLRHIDTISKVNHRLMLELANRLNMSYNTYQTVNYGWRQPNGSFDGLMGRFQRYELDLAQLAIFMRLDRIALVDFVAETYRVRAGIMFRQPPLSAVANIFAMPFENDVWVSILMLLIITTVVLVLELFFSPHNHDMSYMDTLNFVWGAMCQQGFYVEVRNRSARIIVFTTFVAALFLFTSFSANIVALLQSPSDAIQSLSDLGQSPLEIGVQDTQYNKIYFTESTDPVTKNLYHKKIASKGENIYMRPLLGMEKMRTGLFAYQVELQAGYQIVSDTFSEPEKCGLMELEPFQLPMLAIPTRKNFPYKELIRRQLRWQREVSLVNREERKWIPQKPKCEGGVGGFVSIGITECRYALGIFGCGAAVSFVLFLFEFIFRHFKQVYRIIKGYREVQR

>DmelIR76a

MENLLVESYYFSTVLSFFAQQFFADSHATCIFWHPAFDFRLETVHPMPLIIMDWHRWANRSDQDVYDYKIKEDEFEGKGIPYNDWTLRLTVAIERSHCETFIAFQEQIPEFARYFYHASIYSIWRSLRNRFMFVYTKEFEDKKDSYLSGYIFQDQPNILVITSQYLNSSTFEIKTNRFVGPRNFNKNPEPVEFYILQRFDAKGTKATWETQSAMSSKMRNLKGREVVIGIFDYKPFMLLDYEKPPLYYDRFMNTTDVTIDGTDIQLMLIFCELYNCTIQVDTSEPYDWGDIYLNASGYGLVGMILDRRNDYGVGGMYLWYEAYEYMDMTHFLGRSGVTCLVPAPNRLISWTLLLRPFQFVLWMCVMLCLLLESLALGITRRWEHSSVAAGNSWISSLRFGCISTLKLFVNQSTNYVTSSYALRTVLVASYMIDIILTTVYSGGLAAILTLPTLEEAADSRQRLFDHKLIWTGTSQAWITTIDERSADPVLLGLMEHYRVYDANLISAFSHTEQMGFVVERLQFGHLGNTELIENDALKRLKLMVDDIYFAFTVAFVPRLWPHLNAYNDFILAWHSSGFDKFWEWKIAAEYMNAHRQNRIVASEKTNLDIGPVKLGIDNFIGLILLWCFGMICSLLTFLGELWRGQG

>DmelIR76b

MATGIELLVAAALCVACPPLNDSPPTNLIQMGENGTLSPVTELPMDVDASEAGFDADAPVETLETINRKKPKLREMLDWIGGKHLRIATLEDFPLSYTEVLENGTRVGHGVSFQIIDFLKKKFNFTYEVVVPQDNIIGSPSDFDRSLIEMVNSSTVDLAAAFIPSLSDQRSFVYYSTTTLDEGEWIMVMQRPRESASGSGLLAPFEFWVWILILVSLLAVGPIIYALIILRNRLTGDGQQTPYSLGHCAWFVYGALMKQGSTLSPIADSTRLLFATWWIFITILTSFYTANLTAFLTLSKFTLPYNTVNDILTKNKHFVSMRGGGVEYAIRTTNESLSMLNRMIQNNYAVFSDETNDTYNLQNYVEKNGYVFVRDRPAINIMLYRDYLYRKTVSFSDEKVHCPFAMAKEPFLKKKRTFAYPIGSNLSQLFDPELLHLVESGIVKHLSKRNLPSAEICPQDLGGTERQLRNGDLMMTYYIMLAGFATALAVFSTELMFRYVNSRQEANKWARHGIGRTPNGQSVAPSRWLRGWRRLNSGHGQLLGASTHGQNVTPPPPYQSIFNGGSHGDPLNRWRRPLANGNALGNGVLLGGDSEGGVRRLINGRDYMVFRNPNGQSQLVPVRSPSAALFQYSYTE

>DmelIR84a

MIKLQVKVISWPLIILTAFLRVLQIESINTNFLELAAFEDFLRSEHLSHVLVVRGDDADGDWKIECHQKLLANYRVQFYRPEMSANFEDLMFYGSPRTAVLVLNSEHVLVRRQVFGVASEAGYFNNSLAWFILGSGRESLPVEQLIDQLLSGYRMGIDADITVALRGPDNASMLFYDVYRISRQANTPLIIEKKGLWTHSGGYQKFGNFKNTWVIRRRNFLNVTLIGSTVLTEKPPGFGDMEYLADDKQLQQLDPMQRKTYQLFQLVERMFNLSLAISLTDKWGELLDNGSWSGVMGQVTSREADFAVCPIRFVLDRQPYVQYSAVLHTQNIHFLFRHPRRSHIKNIFFEPLSNQVWWCVLALVTGSTILLLFHVRLERMLSNMENRFSFVWFTMLETYLQQGPANEIFRLFSTRLLISLSCIFSFMLMQFYGAFIVGSLLSESARSIVNLQALYDSNLAIGMENISYNFPIFTNTSNQLVRDVYVKKICKSGEHNIMSLQQGAERIIQGRFAFHTAIDRMYRLLLELQMDEAEFCDLQEVMFNLPYDSGSVMPKGSPWREHLAHALLHFRATGLLQYNDKKWMVRRPDCSLFKTSQAEVDLEHFAPALFALALAMVASALVFLLELFLHWLPDFRRRLGTMST

>DmelIR92a

MLLQPLVMHLSQLLRIIVGQYFAEFPSILIVYNNSASTTPLQLEYLSALELVLRELSKPIRLQWINVAFLKDLNDLEDQVMGALNSSVTEGFITILSQTHHFIHARYYATRNANVRLKDKRYLFLCEDESPAELLCMDILQFYPHHLMVRPGTETAPTGPTGPHPDPRRGGGASVSTKNKDDGEGGAGNKTTSPYRDINFELWTQKFVGAVGNLDALLLDAFLPNETFANRVELYPNKLLNLQRRSLLVGSITYVPYTITNYVPAGQGDVDPIHPQWPNRSLTFDGAEANVMKTFCQVHNCHLRVEAYGADNWGGIYDNESSDGMLGDIYEQRVEMAIGCIYNWYDGITETSHTIARSSVTILGPAPAPLPSWRTNIMPFNNRAWLVLISTLVICGTFLYFMKYVSYRLRYSGTQVKFHHSRKLEKSMLDIFALFIQQPSAPLSFDRFAPRFFLATILCATITLENIYSGQLKSMLTFPFYSAPVDTIEKWAQSGWKWSAPSIIWVHTVQSSDLETEQILARNFEVHDYSYLSNVSFMPNYGFGIERLSSGSLSVGDYVSTEALENRIVLHDDLYFDYTRAVSIRGWILMPELNKHIRTCQETGLYFHWELEFIDKYMDKKKQEVLMDLANGHKVKGAPQALDVRNIAGALFVLAFGVAFAGCALVAELLIHRMDLSK

>DmelIR93a

MNPGEMRPSACLLLLAGLQLSILVPTEANDFSSFLSANASLAVVVDHEYMTVHGENILAHFEKILSDVIRENLRNGGINVKYFSWNAVRLKKDFLAAITVTDCENTWNFYKNTQETSILLIAITDSDCPRLPLNRALMTVECRINAVVFVDQTILEENALLVKSIVHESITNHITPISLILYEINDSLRGQQKRVALRQALSQFAPKKHEEMRQQFLVISAFHEDIIEIAETLNMFHVGNQWMIFVLDMVARDFDAGTVTINLDEGANIAFALNETDPNCQDSLNCTISEISLALVNAISKITVEEESIYGEISDEEWEAIRFTKQEKQAEILEYMKEFLKTNAKCSSCARWRVETAITWGKSQENRKFRSTPQRDAKNRNFEFINIGYWTPVLGFVCQELAFPHIEHHFRNITMDILTVHNPPWQILTKNSNGVIVEHKGIVMEIVKELSRALNFSYYLHEASAWKEEDSLSTSAGGNESDELVGSMTFRIPYRVVEMVQGNQFFIAAVAATVEDPDQKPFNYTQPISVQKYSFITRKPDEVSRIYLFTAPFTVETWFCLMGIILLTAPTLYAINRLAPLKEMRIVGLSTVKSCFWYIFGALLQQGGMYLPTADSGRLVVGFWWIVVIVLVTTYCGNLVAFLTFPKFQPGVDYLNQLEDHKDIVQYGLRNGTFFERYVQSTTREDFKHYLERAKIYGSAQEEDIEAVKRGERINIDWRINLQLIVQRHFEREKECHFALGRESFVDEQIAMIVPAQSAYLHLVNRHIKSMFRMGFIERWHQMNLPSAGKCNGKSAQRQVTNHKVNMDDMQGCFLVLLLGFTLALLIVCGEFWYRRFRASRKRRQFTN

>DmelIR7a

MFHHLWLLMGLRSLAMGALHPPQPEAMTPLVAAALEILAEQVSPSQSTLAVMDLTQDAEHRDERQEQLMTIILRSVGSEMALRTFQKPPAEVPASFVVFLVNSAQAFNTLGFHFTDIHSTREFNFLILLTHRMSSRAERLQVLRDISRTCVRFHTSNVILLTEKRDGVVLVYAYRLLNMDCDLSVNLELIDIYKNGLFRHGHEARSFNRVLSLSGCPLQVSWYPLPPFVSFIGNSSDPEERAQIWRLTGIDGELIKLLASIFDFRILLEEPCNKCLSPDIKDDCSGCFDQVIISNSSILIGAMSGSHQHRSHFSFTSSYHQSSLVFIMHMSSQFGAVAQLAVPFTVIVWLALVVSSLLLVLVLWMRNRLVCGRSDLASHALQVLTTLMGNPLEARSLPRSSRLRILYAGWLLLVLVLRVVYQGKLFDSFRLPYHKPLPTEISELIRSNYTLINQEYLDYYPRELTVLTRNGSKDRFDYIQGLGKEGKFTTTSLIATMEYYNMMHWSTSRLTHIKEHIFLYQMVIYLRRHSLLKFAFDRKIKQLLSAGIIGYFVREFDACQYRKPFEEDYEVTPIPLDSFCGLYYISLIWLSAAVVAFILELLSQRIVWLRRIFE

>DmelIR7b

MKYWLYILSCCSLVASTMESSSDWDLAEALAQVVANSEMGRFKTLYIYTHTNSQSTGGHLEELLDQVLMIVPNNLQARRLLLQQSMEYKPYVHAVLALVDGLPSLSAIYARIRATQDLSHTLIYMSMPTDAYGEEMQATLRFLWRLSVLNVGVVLRPPGDHILMVSYFPFSALHGCQVISANVVNRYQVGTKRWASQDYFPSKLGNFYGCLLTCATWEDMPYLVWRPDGSGSFVGIEGALLQFMAENLNFTVGLYWMNKEEVLATFDESGRIFDEIFGHHADFSLGGFHFKPSAGSEIPYSQSTYYFMSHIMLVTNLQSAYSAYEKLSFPFTPLLWRAIGLVLILACLLLMLLVRWRHHHELPRNPYYELLVLTMGGNLEDRWVPQRFPSRLVLLTWLFATLVLRSGYQSGMYQLLRQDTQRNPPQTISEVLAQHFTIQLAEVNEARILASLPELRPEQLVYLEGSELQSFPALAQQSGSSARVAILTPYEYFGYFRKVHPMSRRLHLVRERIYTQQLAFYVRRHSHLVGVLNKQIQHAHTHGFLEHWTRQYVSAVDEKDESVARIASTSYSTLDGIDGDPSLSESEEDQQVAPVRQNVLSMRELAALFWLILWANLGAVVVFVLELLLPRIKLRKILRKMKKSTRASATTTSTLSSPSTTKDIPFSCKDGFQDSWPKCSLLVS

>DmelIR7c

MLHSAVHNVSLVYALVWAIDNYYGMATSTPLAVVQFPTSRESRRLHNDLIDAALGRSSGTGRIQFLLEDDRVEMTETDTDPPPPSGLTGRPIAIWFLDSLRSYFRLEMYLNQLGSPYKRNGFFLVIYTGLEDQPMESLKIMFRRLLNMYVLNVNVFLQRDGTVHLYTYYPYGPHHCQSSLPVYYTAFQDLAAPANGFGLTKPLFPRKLTNMHGCEMVVATFEHRPYVIIEDDPKTPGGRSIHGIEGLIFRSLAERMNFTIKLVEQKDKNRGEILPDGNFTGILKMMVDGEVNLTFVCFMYSKARSDLMLPSTSYTSFPIVLVVPSGGSISPMGRLTRPFRYIIWSCILVSLIFGFVLICLLKITALPGLRNLVLGRRNRLPFMGMWASLLGGLALYNPQRNFARYILVMWLLQTLILRAAYTGQLYLLLQDVEMRSPIKSLSEVLAKDYEFRILPALRTIFKDSMPTTNFHAVLSLEESLYRLRDEDDPGITVALLQPTVNQFDFRSGPNKRHLTVLPDPLMTAPLTFYMRPHSYFKRRIDRLIMAMMSSGIVARYRKMYMDRIKRVSKRRNLEPKPLSIWRLSGIFVCCAGLYLVALIVFILEILTTNHRRLRRAFNVINRYAA

>DmelIR7d

MDIRCVVALLLGLCKVQAVVWPHQHLLEEQLASQISATLQKIFINGLAVYNFGVFISTSYEEMDRDRVILVHQVLNRNLYPPNFPVAVVLASKMNRKITAQVFTQLLFVQNAEQAIAIAEGVNRNGLCVIVLLTSQPERPIMTKIFTYFMQERYNINVVILVPRLHGVQAFNVRPYTPTSCSSLEPVEIDIKDGDLWDVFPRRLKNLHGCPLSVIVWDIPPYMRINWKSSDPMDGLDGLDGLLLRIVARKMNFTLKLIPNEPNGLIGGSSFMNGTFTGAYKMLRERRANITIGCAACTPERSTFLEATSPYSQMSYIIVLQARGGYSIYEVMLFPFEKYTWLLLSTILGLHWIVGSRWRMPSPILAGWMLWIFVIRASYEASVFNFIQNSPVKPSPRTLDQALSGGFRFITDHASYRMTLKIPSFQGKTLISAGQPVDVFDALLKAPWKTGAFTSRAFLADHLVRHRKHRNQLVILAEKIVDNMLCMYFPHGSYFAWEINKLLFNMRSFGIFQHHSQILAWDNLPTTTDTDTPGKRIHSSTESVATGFAESMSFVVAALNCLMGALCISIVVFGLELLSRRRHWTGLEWLFERV

>DmelIR7e

MNHINEFVARAVLHVVHHYILSVTPSLVLTLCCRSNHTCNFYNKMMSTLFREWGLAPLQIVNVLRGVPWHPVPGRRHFNVIFTDSFAAFEEIRMEYYSREYNYNEHYFIFLQARDRLLQGEMRLIFDYCWRYRLIHCSIQVQKSNGDILFYSYYPFGEHGCSDMEPQLINRYNGSMLVEPDLFPRKLRNFFGCPLRCALWDVPPFLTLDEDQEEVLRVNGGYEGRLLLALAEKMNFTIAVRKVHVNMRDEALEMLRRDEVDLTLGGIRQTVARGMVATSSHNYHQTREVFGVLASSYELSSFDILFYPYRLQIWMGILGVVALSALIQLIVGRMLRERMGSRFWLNLELVFVGMPLLECPRSHTARLYCVMLMMYTLIIRTIYQGLLYHLIRTHQLNRWPQTIESLVQKNFTVVLTPIVQEVLDEIPSVQHMRFRLLEANSELDPLYFLEANHQLRQHVTASALDIFIHFNRLSADKVHQRGEQGSGAHFEIVPEDIISMQLTMYLAKHSFLIDQLNEEIMWMRSVGLLSVWSRWELSESYLRNEQSFQVLGTMELYAIFLMVLVGLIVGLLVFILELVSMRSIYLRKLFT

>DmelIR7f

MQGEDANLYVARALRLVIENVLAQLSTTLVVTISTRHLGTAHWFEYMMNILMDSWRMVAVQLLRIRPDLVVNPVPGRKRVSLLMVDSYQGLLDTNITASNANFDDPDYYFIFLQARDHLIPKELQLILDHCLAHFWLHCNVMIQTAQVEVLVYTYYPYTADACQKAYPIPVNTFDGRKWKASQMFPDKLSQMHGCPLTVLTWHQPPFVELVWDPKHNRSRGSGFEIQLVEHLARRMNFSLELVNIALLRPNAYRLAEGSSEGPIEKLLQRNVNISMGYFRKTARRNQLLTTPMSYYSANLVAVLQLERYRIGSLALLVFPFELSVWMLLLLALLIHLGIHLPSARRGNEEDGGGGLQVVALLLGAALARLPRSWRHRFIAAHWLWASIPLRISYQSLLFHLIRLQLYNTPSFSLDQLLAEGFQGICTANTQRLLLEMPQLARDPDSIQSVDTPFDWDVLNVLTRNRNRKIFAVANQDVTLSFLHSSAHPNAFHVVKQPVNVEYAGMYMPKHSFLYEKMDDDIRRLDASGFIHAWRRASFASVHRKEQVHMTSRRYINHAKLSGIYMVMAGLYLLAGLLFAGEVLLRQRN

>DmelIR7g

MNVTSLLNFESMKYIGAQTQAASINHHVAQALRVFIEDFYQRIAPAFIVVLSCRRPSPMNFYRNIMQLLYESVDTMIVQLVLVELGRPRRIAGPRTHNLLLVDSLDALLDIEIHTYTAQSDTSEYYFIFLQQRDALIPHDMQGVFAYCWRHQLINCNVMTQSSGGQVLLHTYFPYAPGQCNDSQPTRINMFLGESWKHRDYFPSKLHNLNGCPLIVLARKVSPFLDLDEGQRELRGLEGRLLQELSRRMNFSIQFSGLQDQLKNRTTWTEKQLLQKLVQERIAHLAIGYVRKRIQYATNLTPVFPHYSNRVVGCLLLNAHNLTSLEIWSFPFQALTWICLVAGDRLALVLAVYAASLGLPIDPPERPSLQLLFASWLIFGLIVRSMYSALLFFILRYHLHQRLPGNLQDLTHGDYAAVMGRTTLQDLREVPSLQDLLGLKSVIVTSEREEEVLRTLDRCTLREGAGSHPLFFGLISQDALLHLTQRGHRAGAYHIIPQDVLEQQLAIYLQKHSHLASHLDHLVMSIRSVGLVHHWAGQMASERYFRSRFLYREKRIRQPDLWAVYILTAGLYLLSLVVFICELLASRRAGL

>DmelIR10a

MAVLGTVFLLFMLDLKTLNLTRLNGLLVEPTRDLPQLELWLRAGSDHQDAENPYVQWFLLRTEIPLSIVTYQENRYWMDDPFGRRNLVLVMSLDQLLTNRGAAAPIQKASTFFYILADQDKDLSADEQLRLEGSCRQLWTQHKVYNRFFLTRDGVWIYDPFKRRDSAFGRLVRYYGSETLDKLLFRDMAGYPLRIQMFRSVYTRPEFDKETGLLTRVTGVDFLVAQMLRERLNFTMLLQQPEKKYFGERSANGSYNGAIGSIIKDGLDICLTGFFVKDYLVQQYMDFTVAVYDDELCIYVPKASRIPQSILPIFAVGYDIWLGFVLTAFACALIWLTLRVINLKLRIVSLGNQHIVGQALGIMVDTWVVWVRLNLSHLPASYAERMFIGTLCLVSVIFGAIFESSLATVYIHPLYYKDINTMQELDESGLKVVYKYSSMADDLFFSETSPXWNRDLRADVIDEVARFRNKAGVSRYTSLILESSHFTLLRKIWVVPECPKYYTISYVMPRDSPWEDAVNALLLRFLNAGLIVKWIQDEKSWVDIKMRSNILEADAESELVRVLTIGDLQLAFYVVIGGNLLAFLGFLAEHFRWKLQKKGV

>DmelIR11a

MRFAILWLFSGCLLPGIQVGIWVVVRAQPTGRDVLLSRLGNQQNELNTRRLANASSYLTRNYIANRINTLVVREICVECPYELSERQRQLVDQILASLAPELSVLLHKGTAEETTWEYTLFVVNDHTAFTGQVFIFPDELLEREFFCIVVVSEIQSRQFVRQTVGSIVKSNLQMHFVNVVVVAQLEDGTVGTYSYKLFKANCTPGITVRQINHFDRITGKPQQSMPDLYPVRNGHLGDCPFNVGAAHMPPHLIYKRHKDPPPASNVSIPAEDLAGIDWDLLQLLAKALKFRIQLYMPQEPSQIFGEGNVSGCFRQLADGTVSIAIGGLSGSDKRRSLFSKSTVYHQSNFVMVVRRDRYLGRLGPLILPFRGKLWGVIIVILLLAVLSTCWLRSRLGLSHPIEDLLTVIVGNPIPDHRLPGKGFLRYLLASWMLLTLVLRCAYQARLFDVLRLSRHRPLPKDLSGLIKDNYTMVANGYHDFYPLELTCRQPLDFSARFERVQRAAPDERLTTIALISNLAYWNHKHPNISRLTFVRQPIYMYHLVIYFPRRFFLRPAIDRKIKQLLSAGVMAHIERRYMQYENKRKVASNDPVLLRRITKSIMNGAYRIHGLVIVLATGMFILELLAGRSNGRLRRWMEWVHQ

>DmelIR20a

MLASLNRSTGLSAELLDLYGLVVHFLLSGEHTTLVYFNPAGLDCSWGVLWQRNLTAHPQIVWQRNYSYPDLYYQFNAKLLVLACLPMDSRAAIQLEILANSLSHLRTVVRLLIEVAGPDQVTLARQYLSFCLRRSMLHVELYFRDYHHSLILYSFRAFPSFELVMRWISVGQGVKLFLHKLDDLRGHRLRVIPDLSPPNTFFYRDARGDNQVTGYLWDFLATFAGRLNAGLEVVRPSWRAGSASDSSYMLEYSAKGLIDVGLTTTLITKWNLWAIHQYTYPLLVSSWCTMLPVEKPLATPDLFGRIVCPTLAMTLLLIILVTWLVFRQLRCLTRLKNSRPARIVPHLLTLLLLTTCSAQLLSLLIFPPYHVRIASFEDLLRGDQKILGMRNEFYNFDGAFRARYAGVFYLIDDPNELYDLRNHFNTTWAYTMPYIKWLVIKTQQRHFSKPLFRWSKDLCFFDFMPTSVIVAPDSIYWESIKDFTFRIHQAGLMKHWIRKSFYDMIKAGKMSIKDYSDLETLKPLNIGDLEIVWRVCGAAIAVASAIFIMELLYFYINVFFNSL

>DmelIR41a

MFIDLSWSLVLSAIVGKYLNESTICIFWNDKFEFQLLHKSDYISFVGINIKSFDDNGGHYIIDTGLKKKELQNKHLFLDELVIKIIISIEVTHCETFVVFDKDIDRFVNAFNKASVYSIWRSLHNKFVFAHIANESPESRNHFFEDQPNILFVVRDHSSASSFDIKTNKFVGRKAENPSQMILVDRYLASEQRFQFGKSLFADKLNNLQGREVIIAGFDYPPYTVIKHNMSTNAQDMGVSGESDFKNVYIDGTETRIVLNFCEQFNCTIQIDSSAANDWGKVYPNMSGDGALGMLINRKADICIGAMYSWYEDYTYLDLSMYLVRSGITCLVPAPLRLTSWYLPLEPFKETLWAAILLCLCAEATGLVLAYKSEQALYVLPGYREGWWTCTSFGVCTTFKLFISQSGNSKAYSLTVRVLLFACFLNDLIITSIYGGGLASILTIPSMDEAADTVTRLRFHRLQWAANSEAWVSAIRASDEALVKDILYNFHIYSDDELLRLAQDQHMRIGFTVERLPFGHFAIGNYLGPQAIDQLVIMKDDIYFQYTVAFVPRLWPLLDKLNTLIYSWHSSGFDKYWEYRVVADNLNLKIQQQVQETMTGTKDIGPVPLGMSNFAGFIIVWILGSAIATLTFLLELSLTYILKQSNLK

>DmelIR47a

MRQIKLLVWLLVVGVVSSTEQLQFLKNFLEAVHKERSISTILLIQRKVHKNDFLHGLYPIFWPIICLDETKRVELVNNFNKDFLALVYMESEADTLLLSALAADLNHIRDARIMIWLQMSPSENFLDRIVFQASKQKFLNLVVIENTLKTRRFYPFPQPKVQVIDKPFEEKEIYPALWRNFMGKNAIAVPDLVPPRSFNSFDPKTGHRRESGSIYNVFKAFTQRYNITMLLKWPLIRNTTQEEIIGKSVRGEIDLPITGQLISFRHPNGSRSQPLLGMTALSIAVPCGPELPMFDRFFLFYGLATPITITGYYVLLNTIEIILGTLSDRIKRHPRRKKILNLVLNLRVFSCILSLPTPQGNRLRSVKGQLTMVMSITGLILSCIVAAQTSTILTMKPQYRHIKNFQELSDSNITVVCNHLNYLTIKQQMDPKFMAKFMQNIWIVNSIEQMKMIFDLNTSYAYQTFSYKKDPFTLLQMHTTRKAFCRTPGLDLVSGLAYTAVLEKNSIYALALQDYTLKAFSAGLVYYWAEESIRDLISTVGRTQFEKLPIVIGYQSLKLQDYNVCWKILLIGGALAFCVFIVEVVVGLINRRI

>DmelIR47b

MREAQIIIFLLTSAAAVTLKQYEFLXSFLKAGEQEQTITTLLMMQKHVHTKNLLQGLYPXPWPIIHFVETQRIKFIALLYMSSEKDIFLSSLAANLKFERLDKPFGKSNIFPVLWRNYMGXIALTLDHLVEPRSFYWTDPRTNIKRRTGYIYMLITNFAEQHNITLQLXSPPNEDMSQMVIIERTHKGPRSTHNWADDQLETFERXQDSLLPWHGSMAIVVPCGQEMSAYERFHAAHAFRAPIIFFGFHIFLSLIDFLLRTISDRIRCNPRRIQLLQTVLSLCVLRCILSTSLPNSNXLRSRLRDNSPXXXVLQAXSYSALWXLTGTAXQXHNRDFQSHKLHDYXTTDGSXHSIEVPGLLKARNXXIXLFHIFSSLGTKFDLRIGSAGSHTSGVEFRYYELLDRXSSLENNIVSQVFTILKLPYSRFRVLKLEDCRGCWQTLFVGFSIATFVFIVNVLMGFFRNINQKK

>DmelIR48a

MHLLITETYMIIGKTLHDILNELNERLIISTNIIFCKQFDNLIHFEAQTSRFVYSSLEAFNITSLWNHVGNDNKLFVIVGNVPPYELFAKLELSSPENCTQFILNNTVDMCADALVKNSKAFSVSRELRIAPANVIVPHGKPLLSYRYLAAPFNTKVWIALGTYVFLISGFLCLIHWLRSGKWDFSQNLLEVYSSLLFTVFHLKATNGIERYILFGVLFISGFVYSTSYLRLLKSMLIAETFEKQIQTFEELAESNIPLLINPYDRMIFQHHHIPKSLWTAVRTVSSETLLNHRSHGYVRLCPAILTASKIPSHTHRHLFSVCRFSHEQEVVPKGSSXXSLVPCIRKRNREXNHLGCLSGVSWPGISXFFHYGALGGEAFGSILLHDANYFPSPRLFRRLAELHYGSY

>DmelIR48b

MILQQSSNLLKLLLLLAISSVRTQGLNDIIIELNQRLLISNNFLYCNQSDKLNEYEIKYLQHMPPISLMIFTSIESMNFTQVEYNLGADNKLFLIMGNEEPPYDFLHALNLHFQFAEYIIVIDEPVDLKKSTKWLDFVNHLWQQGYVQLLIYTSYDEKLYHKIIFPETVIEETLVEQYISIRGSFNNLYGYPVRVAAYNNAPRSMLYVNRWGKHIFAGFYMRFLRAFIDARNGSFVPVLTPSNSPGNCTLNLVNETVDVCADALAANPAAFSLTHGFRIASANVLVTHAKPLHSYRYLTAPFQWSVWACLVIYVLLVVNFLSFIGWLRSGKWEFSKYLLEVFSSLLFSGFYLKEIRGRERYILFGVLFIAGFVYSTEYLGLLKSMLISEVFEKQIDTFEALVESNITLMVDPYDKILFAKYNMPEILSPIMELVSFETLLKHRNRFDQDYAYILFSDRMALYDYAQQFLKHPKLLRIPIDFSFLYTGIPMRKRWFLKHHLGRAWYWAFESGLTRKLALDADFEAVRVGYLSFLITEHVEAQPLNVDYFVMPAIALAIGYILALLSFVIEMTAWRIREFLGCRKATMTSTGCSEGGHVDVD

>DmelIR48c

MSLLRIILIIIFLRIVSSIPDTIISHLSAELQIKIQIYFGLGNDLYDFSRLDGNYQKIIISHNISEEFKTYHDEPVLIIIRLERDLNLNLATLDVLRSYLTDRQYNDILLIDNDEENLNSYVDIRKAYWNAGFSQVLIYNSQQRTWSIKPYPYLQIRPTSLKEYIENRNTRNLMGYPLRVLVTNDPPHCFVDKDELPGSPNRYKGSIVTMLKIFADQLNATFQANPFREFRRYSTADCVQMVSDDEIDACGSIFIRTYTYATSQPVRLNRVVIMAPFGNPIEKFYYFFRPFDLYVWIGTGIIVVYIAVMGSLLHRWHFKEWNVGQYLLLAVQTLLNRELSLPQSSSGSKFMLLLLLFAIGFILSNLYVALLSMMLTTKLYQRPIENLADLKAANVNILLQTHNIRPNSVYGSSEELRERFLLVEESQHLEKRNGLDPSYAYVDSEDRMDFYLYQQKFLRRRRMKKLSNPVGYTWAVQVIKQNWVLEKHYNDHVQRFFETGLQNKLVDDVHELAVKAGFLHFFPTQTQTIEPLRLEDIVMAAMVLGGGHALAVICFLVELFA

>DmelIR51a

MYNVLVLFLLLFTRAQMEPHRRGHNMTLLRSVLTVIRGRENWKNTPIFLGGHCNSDDLNNLMSWLQNTMEVTCHTVDTSTSAKNENALGHFNINADNSLGLLFCQSSHELIWFNMDKRLRRLRGIRLIVILSDKRSSSSKAIMSTFKRLWHFQFQXNFQGYVVSTPVENDIPRVFFVKDKKTGRKQIRGFGYRTFVEYLHRYNASLHVSNSQQEHAINSSVNMGRIINQIVDGQLEISLHPYVDVPENMGDNSYPLLIASNCLIVPVRNEISRYMYLLLPLNQSSWILLLGSVIYISGVLYYIQPGLLHRTWDQRIGLNILDSISRIINICSPSRIYNPSLRYFIVSVHLSILGFVVTNLYSIMLGSFFTTLVVGEQVDSMQQLIQXQQKVLVKYYEVSTFLRHVEPDLVDGVAQLLVGVNASEQVSALLGFNRSYAYPFTLERWEFFSLQQQYAFKPIFRFSSACLGSPIIGYPMKSDCHLQSSLNMFIMRIQAAGLLRHWVVSDFNDAMRAGYVRLLENFLGFHSLDVDSLRLRWAVLLCGWLLSTLIFLCER

>DmelIR51b

MCKVLTLLVVILLLALTNAAYNVTLLKSVLSLISTREPWINTPIFVGHNTQGGDLNDLIIWLHQTMGVTSLTMNLFLQPEHIRPLGHFKITRYNGIALFFCHDKHDIMWLTLDRNLRKLRRIRLIIILRNQRSGSQGAIKSIFNALWQYQFLNVLVLQRDQLYSYTPYPAMRFFKLDIHTEPLFPHAARNFHGYVVSTPAENDIPRVFHVHDPLTKSRKVLGYAYRTFVEYLDHYNASLRLTNPDENLDPTTSVNMNHIVQLIIDGQLEISLHPYVFTPPTATKSYPLLIYPNCLIVPMRNEIPRHMYLLRPFQLYSWYILLFAVFYITGILYCISPKLNKSSWPQRLGLNFLDAISKILFISPPITIYRPTWRHLIIFLQLSVLGFMSTSWYNIELDSFFTTIVVGEQVNSMDQLVHQQQRVLVKEYEINTFLRHVEPRLVEKVSRLLVPVNASEQVSALLSFNRSFAYPFTEERWQFFAMQQQYAFKPIFRFSSACLGSPHIGYPMRVDSHLETSLNHFILKIQDTGLLNHWVVSDFNDAMRAGYVRFVDNVLGYQSIDVDTLRLGWCVLGIGWILSALVFSCEYWHLYPWRFIA

>DmelIR52a

MALGWSVIILGFIGQLSAQILNYTQSRDLELLEGSLFRVLSRLNLEEEYNTLLIYGKECVFHSLLRKLEISAVTVPSGSTDYDWSFSTAILILSCGYDAENEENSYTLMKLQRTRRLIYLEDNSEPESVCMRYSLKEQHNIAMVKSDFDQSDTFYSCRLFQTPNYVEGHFFKDQPIYIENFQNMRGATIRTVADSLVPRTILYRDEKSGETKMMGYLGHMINTYAQKLNAKLHFIDTSKLGAKKPSVLDIMNWVNEDIVDIGTALASSLQFKNMDSVWYPYLLTGYCLMVPVPAKMPYNLVYSMIVDPLVLSIIFVMLCLFSVLIIYTQHLSWKNLTLANILLNDKSLRGLLGQSFPFPPNPSKHLKLIIFVLCFASVMITTMYEAYLQSYFTQPPSEPYIRSFRDIGNSSLKMAISRLEVNVLTSLNNSHFREISEDHLLIFDDLSEYLVLRDSFNTSFIFPVSVDRWNGYEEQQKLFAEPAFYLATNLCFNQFMLFSPPLRRYLPHRHLFEDHMMRQHEFGLVTFWKSQSFIEMVRLGLASMEDLSRKRNEEVSLLLDDISWILKLYLGAMFISSFCFILEILRCGERCKRLWRCRW

>DmelIR52b

MTWLVILLCFLGYMAAHIADISVQNQSLMDNELINLLLKLRNEEFYDTLLVYGKDCEFHSVIKNVDVAVVLVSDSMNFEWNFSSLTLILSCGPDIDNGGPNSTSIKLQRNRRLVLLKEDFQPSNICNIYTQKEQYNIALVRENFTKSKSIYTCRYFQDPNVDEVNLSGTKPIFIEQFQNMKGKAIRIVPDLLPPRVMLYQDANDGELKMIGYVANLITNFAQKVNATLQLDFLKPSTSITEISRMAKDDELDMGITLEASLNTSNLETSSYPYLLTSYCLMVQVPAKFPYNLVYALIVDPLVLGIIFVLFLLLSVLLIYSQKMSWQDLSVANILLNDKSLRGLLGQSFPFPLNASKKLRLIFTILCFASIMLTTMYEAYLQSFFTNPPSEPEICSFQDVGSYNRRIAMSALEVNGLIKTNNSHFREIRMDDLEIFDNMPECYELRDAFNLSYNYVVTGDRWRSYAEQQTLFKEPVFYFARDLCFSRLIFLSVPLRRHLPYRHLFDEHMMQQHEFGFVNYWMSHSFFDMVRLGLTSLKDLSRPLAYTPSLLMDDISWIMKIYLAAIVLCVFCFLLEIGVDKWKRWMKFRNLQILNTC

>DmelIR52c

MVWLIIILFCLGNSSSQILDVTNNSHLDFDYRLFGLLQRLQVEKSYDTLLVYGEDCAIPSLFERLQVPAVLVSSGSTNFDWNFSSLTLILSCNFQDEREENYRTLMKLQTSRRLILLKGHIKPESVCDFYSKKEQHNVAMVKENFYQLEVVYSCRLFQDQNYEKLNLFDGKSIYKDQFRNMHGAPIRTLSDKEPPRTIPYIDSKTGEEKFKGYVGMLISQFVKKVNATMQIREDLIKDDEEVSFVDITNFTSNDILDIGICEARTLEMSNYDAISYPYLMSSYCFMAPLPDSLPFSDVYMAIVAPSILIMFLIIFCICSVLIIYIQERSYRSLTIRSVLMNDICLRGFLAQPFPFPRQYNRKLKLIFMLVCFSSLISTTMYTAYLQAFLWGPPIEPRLTSFDDVKKSRYTMAINIYEREFLEALNVSLEDVEIYDYGKFSKLRSTFNTNYLFPVTALQWFTINEEQKLFKYKIFYYCDAFCLNQFDILSIPLRRHLPYRDIFEEHMLLQKEFGLTKYWIDQSYRDMIRANLTTFKDFSPLLENDYIEVHNLYWVFTMYFVGMGMGLCFFILEILRPLRYWRNCKIKCEYCYAFLKNFAK

>DmelIR52d

MVRIIIILLCLGYTKARILDATNTNHTDLEERLLSLLLRLQQEQFFNTLLIYGEDCAFSSLSRRLQVPTILVSSGSTSFEWNYSSLALILTCEFKAEREENYQTLKKLQMNRRLILLNGNIKPDSVCDFYSKKDQYNIAMVNNNFHQVGIIYACRLFQERNYEKVYLSEGNPIYVDQFRNMQGALLKSITFNLIPGSMAYRDPKTGQEKHIGYVANLLNNFVEKVNATLDMQVKLHKAGKKTSFYNITKWASEDLVDIGMSYAAYFEMTNFDTISYPYLMTSTCFMVPLPDMMPNSEIYMGIVDPPVLVVLIAIFCIFSVMLNYIKQRSWRSLSLVNVLLNDICLRGFLAQPFPFPRQSNRKLKLISMLVCFFSVITTTMYTSYLQSFMWGPPIDPKMCSFADLENSRYKLAIRRYDIEMLRPFNVSMDHVVVFDESSQLEYLRDSFDDNYMYPMSALSWSAFKEQQKLFAFPLFYYSEKLCLKPISFFSFPIRRHLPYRDLFEEHMLQQNEFGLSTYWIDRSFSDMVRLKLATMNDFSPPRLEDYIEVSDLSWVFGMYFTGLGISCCCFGLELLGLPSWTRRLRLTNWLRVRN

>DmelIR54a

MWTVITGIVLWAPVLVAGSAVDFIFRAAAEHSLSVIMIRIDYCPYNWAKDIFENQTIPVVVLSDSETFINIRMFSRPLHVACLPGHELQKDLALLENFTSSLMDFPSQKKIVYISNNFSDPTRMDYIFETCYHRRIWNIVGLLASDEHRYFYRYHLYPSFRTEYRSLESSTIFDKDFPNMHGHPLTVMPDQWLPRSVLYVDRRTGKQILAGSVGRFFHVLSWKLNATLQLSKKVTTGRFLNATALKELSESFSVDVPASLTIMERVEQLASTSYPMEVTHVCLMVPVARRIPIKDIYFILSSASNMFLAIVIVSSYGLALNLLRNMTHRDVRLVDFVLNDKALRGILGQSFNLPLSRSFSTRLIFLMLGIVGLNVSSIFGAGLDTLMAHPPRQFQARSFAGLRRTKIPLVTTEEDFPTWMKLRVPMLVVNVSEYNHLRNGRNTSNAYFASRLYWNLFSEQQKRFTRELFIYSTDDCLWSLALLSFQWPQNSLFTEPVSQLILEVNANGLYDFWVGMHYYDMTAAGLSGLEDPSLQLKEREHPTSLRIVDFQWMWQAYGTFMVIAILVFLLEVSWHRITSLFVSLVY

>DmelIR56a

MGSRFFIRNLILFGLLASSNMQIPFGELEKKFELDVDFLLGVTELVGHIQGLYSITVYADCIDIHPSIQQRIMDKFMVPVNTIGSNLSRPNYHKLDNSRIRIVLFTGLNDTILVNLNKTDVPYSDNFYMLAYASAIKNKCIELDFIEEVFTLLWKMSIQNAILLIRGEFMMEMWSYLYMGKIHKIKLTKPNSYLESLRKYNYRFSLEVINDPPAIFWYNSSEQADVTGGGNLSVSGPLGLIIINFLRHLNVTIDIVPIPGKQTSQYELFQQPDNLRAENGVNMVGSALLKYSPMVTQSRMCLLVSNRRMIPFSRFLDRLVSPGVHKLTFVSSIGIFVIKYFSHRPRSFVDAIFCTIRFFFAIPLPSIILNRLPVVDRFIEVFIIIFVQILLSSNISITTSALTTGFWEPPIINVETMRASGLHILTEDPTILQAFKENILPSSLADLVILVDEDTYFHHVTTLNNSYVYVVQAHNWQIFRLYQQQMTNEPFEIASEELCSKWRILGIPLNPKSPLRFMFKDYFYRILESGLREQWVHSGFKKFCEFNNLKKLPVDSVDSWQPLSIEFYSNVIRAYIIGLVIATLAFVAELLHNGYRRKNVKKT

>DmelIR56b

MLLDTDLASGVIRSPYSFDIPHAFIFNETQFVVPKFCGPYMEIVKHFAEVYHYQLFLDSLESLPKKSVVEQDIISGKYNLSLHGVIIRPEETSDFFNATQHSYPLELMTNCVMVPLAPELPKWMYMVWPLGKYIWTCLFLGTFYVALLLRYVHWREPGNATRSYTRNVLHAMALLMFSANMNMSVKLKHASIRVIIFYTLLYIFGFILTNYHLSHMTAFDMKPVFLRPIDTWSDLIHSRLRIVIHDSLLEELRWLPVEYQALLASPSRSYAYVVTQDAWLFFNRQQKVLIQPYFHLSKVCFGGLFNALPMASNASFADSLNKFILNVWQAGLWNYWEELAFRYAEQAGYAKVFLDTYPVEPLNLEFFTTAWIVLSAGIPISSLAFCLELFIHRRKQRRPQYERFECYDY

>DmelIR56c

MRSSFRICLFLLTTYHPSHGWNMQHLLNLLAPFGRMNVFQEIVWFVSPHQRLDQLDEFIMRIDEAFGKSATQTVVNNNTEMRMIYSSARRNHMSFVFTTGAEDPIMKVFSKVLLGRHFYVSMVIYVDKVGDMHPIYDLLTFAYNQQFFNSMVHFESMEGVNQLFGVSKFPVMSFENRTDFLKYMGKIWKQVQNARSDVGGFGFTTPLRQDLPHLFQSQGHYDGSTYRIIETFVRFINGSFKELIMPPDSLGGQVINMKDALQLIRERKMEFCAHAYALFMSDEELEKSYPLLVVQWCLMVPLYNSVSTYFYPLQPFDWNVWFFALGALLALVLLELMWLRMFGGWSGYRGAVLNSFCYIINVPIEGQLQQPCLLRFLLLATVFFHGFFLSAYYTSNLGSILTVNLFHAQINTMNDIVSAQLPVMIIDYEMEFLLNLNKELPQEFLELLRPVDSAVFSEHQTSFNSSFAYFVTEDHWEFLDEQQKHLKQRLFKLSSICFGSYHLAFPLQMDSSLWRDIEYFTFRIHSSGLLNFYARSSFGSALHAGLVQRMPDTQEYTSAGLQHLAIAFILLLVMSFLAGIVFVLETLSR

>DmelIR56d

MDNRAAELILRERNIFPTNGSDNITLLNNMFVLEMFYRITQLYHFKNFIFYISERLDLNNKDSQEFFHNFWTYFPMAPNLIITREHHLGIPMMQFISTPSLVMVFTTGKDDPIMELASHNQQGIHWLKTIFVLFPSLQSRDFETNPESLAQFTAEIKDVYDWVWRKQFINTFLITIKDNVFILDPYPTPSIVNKTGVWQAEEFFHKYAKNMKGYLVRTPILYDMPRVFKSDRPTNRYEKNFIHGTSGNLFLGFLEFVNATLMDTSANVTADYLNMTNLLDLVSQGVYETLIHSFTEITTKFVVSYSYPIGINDCCIMVPYRNQSPADQYMHEALQENVWVLISLFTLYITVAIYLCSPLRPRDLSAAFLQSICTLTYSVPTFIIRTPTLRMRYLYILLAIWGIVTSNLYISRMTSYFTTAPPVRQINTVQDVVEANLRIKMLAIEYERMAKSPLQYPESYLNQVDLVDKHMLDLHRDPFNTSFGYTVSSDRWRFLNLQQLHLRKPIFRLTEICEGPFYHVFPLHKDSHMRSVMTEYIMIAQQAGLMNHWERETFWEAVHLHRIHVHLFDDEPMALSLDFFSSLLRTWTLGLILAGLAFAAEMKWHEHVTFKRRPVIRITRKPRSFLRRFMKL

>DmelIR56e

ERXAFRNQWAFCFPRTXAIEVVLSAWSPXCPGQRSKPQPISXPHHXGSCWRKRKWKXKPRLLVVDKRTLVEHLNSLNDGYAYCIIAGHWQVGMM

>DmelIR60a

MWCNNPGLIIIIFLGQILNLCQGIVNLSNETANTVIFMLPEKDLGPDVWKAGVGCLDSFAQIFFFRNPKERFTRAYNLMLVHAFHLSSPADQIQEGFSKLINEAVTNPGPPDREELFQMRVASDYNITNGTEDKGELILADNYVIVVDSVDRLKELMKKKIVEMRSWNPGARFLVLFHNATCRNRPLGVASNIFKDLMEMFYVHRVALLYANSTMNYNLLVNDYYSNVNCRILNVQSVGQCHDGKLYPNNAVVKASMQDYVSGFSPRNCTFFACSSISAPFVEADCILGLEMRILGFMKNRLKFDVNQTCSLESRGEMDGPANWTGLLGKVQNNECDFVFGGYYPDNEVADHFWGSDTYLQDAHTWYIKMADRRPAWQALVGIFEAYTWIGFILILIISWLFWFTLVMILPEPKYYQQLSLTAINALAVTISIAVQERPICETTRLFFMALTLYGLNVVATYTSKMIATFQDPGYLHQLDELTEVVAAGIPFGGHEESRDWFENDDDMWIFNGYNISPEFIPQSKNLEAVKWGQRCILSNRMYTMQSPLADVIYAFPNNVFSSPVQMIMKAGFPFLFEMNSIIRLMRDVGIFQKIDADFRYNNTYLNRINKMRPQFPETAIVLTTEHLKGPFFILVVGSCWAALTFIGELIIHRWRTQLVSTSEQQDRRSDKRRRRRRRRKPEKDNRWQRQVQVAPVVRFTPVKRRKVFQGQTSQK

>DmelIR60b

MRRSLYLIIAIGLVDVHCVSLRYILNALENELQYRAILLVESASEIESCWEQKYIQGAVPILNFNANQSLYLKDALNTNILALVCLNENVESTMQALYENLEDMRDTPTILFVLSDSKVQDVFLECLRRKMLNVLAFKGLDRGFVYSFRAFPTFRVIERNVMDILQYFEQQLEDLGGHTLTTLPDNIIPRTVVYKSPDGSRQLAGYLYPFLRNYVSTINATLKVCWHLVPEDGMIQLGEVVRLSEIHDVDFPLGMHGIEHGSTSQNVPLEVSSWFLMLPMEPSLSRAQFFIMLGFEKVTPVLLLLTILLSTAHRIEMGLRPSWRCYVLGDRVLQGTLGQAFFLPRRLSVKLMLVYSLILLNGFTFSNYSITSLETWLVHPPSGHPIHSWEQMRTLNLKVLIVPSELDSMTKALGKQFTESNSDLFELSKSGNFQDKRLAMDQSYAYPVTCTLWPLLEHAQIRLPKPEFRRSREMVLIPLLIMAMPLPKNSMFHKSLNRYRALTHQSGLYEFWFKRSFNELVALRKIHYKVNGDHQIYRDFEWQDFSYVWLGFVGGTIASILVLLAEIGYHRWQLNQN

>DmelIR60c

MEMRLALFFTFACLAGAHDGSLRNMLKSLEDELGYRTILLLEGFVYSFKAFPTLRVVKRRVKDVRRYFEPQLEDLGGCVLKVVPDGIMPRTMVYQGEDGELQMGGYLSHFIRNYVSTINASLQIRWDLFPEDGDFDMDSLTGSNHVDFPLGLGSLSFQTLHKDVAMEISSWFLMLPMEPSLPRARFFIRFGISLYLIPLIILLAIVLSNAHRFEAGLTPSWRCCSMGNTVLRGVLAQAFVLPKGLSPKLMFVYWLLLVSGFFVSNYVIVYLTAWLIQPPTSDPVTDFDQMRRAKLKILMVPTDMDYLKSIRGAEYVDAHSDVFQTADSTDFQTQRMSMELHFAFSVTGTLWPLLRQAQVKLHRPIFRRSKEMVFLPFVIMGMTMPNNSIFLSSLKQYRLRTSEAGLYLLWFKKSFSELVAIHKISYKEDWVHDSYSDLKWEDFLFAWLGFLGGTTVSCLALLAEIGYHRWLWKRTHQ

>DmelIR60d

MRLAIYVAFLSSIGNRSGFLSSLLMSLGKELHYKTILLVGGSSTCWSLEPFETGVPILNLRGENNAYPQDTFNSQMLALACLQTESEDAVKLLYRSLKDMRDTPTLLFASSEEHIHDTLFLGCFRENMLNVLALTASSKEFIYSYQAFPTFRVIKRKLVEIHRYFEPQLKDLGGHIVSALPGNIMPRTMCYRNAEGERQLAGYLNTFIRNYVESINGTLRISWGLVPEDDMRHLTISRLSKIQHVDFPLGIIPLYNKTDKQHVYMEISSWFLMLPMETSVPRAHLFVKLGLERLLPIIVVVGAVLGNAHRIEVGLGPSWRCYYLADKVLRGALAQPIVLPRRLSPKLMLIYSLLLLSGFFLSNYYMASLTTWLVHPPASDRILEWDQLRYLHLKVLTIPEEFKYMSLILGTDFMTAYGSIFQLTNSTDFQRRRISMDPSYAYPVTTSLWPFLELSQVRLRRPLFRRSYDMVLQPFQVMSLPLPRNSIFHKSLLRYAALTRETGLYYYWFRRSYYELVALGKISYKEEEGNPYCDLKWNDFRIVWLAFLGGTIISCLALLLEVAHYRWHLGNSSL

>DmelIR60e

MVIKMISFLLVSVLLCLVGASDSESMQVQVLQDLNLALQTELNVFIDFECCATSEILHKLDSPRILLSSNSREARDLRIRGNFTESTLIIVSVMDSDLNPLVASLLPRLLDELHELHIVFLSNEEPGFPKQDLYTYCFKEGFVNVILMSGKGLYSYLPYPSIQPISLSNVSEYFDRARIIRNFQGFPVRILRSTLAPRDFEYSNEQGGLVRAGYLFTAVKELTYRYNATIESVPIPDLPEYDVYLAVAEMLHTKKIDIVCYFKDFSLEVAYTAPLSIIREYFMAPHARPISSYLYYSKPFGWTLWAVVISTVLYGTVMLHLAARGARVEIGKCLLYSLSHILYNCHQKIRVAGWRDVAIHGILTIGGFILTNVYLATLSSILTSGLYDEEYNTLEDLARAPYPSLHDEYYRSQMKAKTFLPERLRRNSLSLNATLLKAYRDGLNQSYIYILYEDRLELILMQQYLLKTPRFNMIRQAVGFTLESYCVSNSLPYLAMTSEFMRRLQEHGISIKMKADTFRELIHQGIYTLMRDDEPPAKAFDLDYYFFAFVLXTVGLISSLLVFFAELVSGHL

>DmelIR60f

MRFHLNIANSGLLGLHLCPTRSALPEQNPCFSKAGAVIXNLTLPWRRWRERCLLGALRPXTLPTPELQCXSKYLPXRKSQQENASSGLPGFCXGDXQTELHRGSRAIALPRSPYHDLYYVWIAYLGGTMIGIGMLAVEIACFKWDLLRRPPIXMY

>DmelIR62a

MYLQFLFALFLSRYQIVATENFDRAFELALFLDRIGRVHRLHAITIVNSLGSVDPSYLDDLHRGLMCNSSNHFYMLPQMTATDKDSSHVHFSSLQDEETIYLVFARDSKDAVIYLQAERARGRRYTRTMFLLRKQESQKDIKYFFELLWKLQFRSALVVVAARNFYQMDPYPTVRVIRMRRLSSYDPHHVFPPANRKNFRGYRMRLPVQQDVPNTFWYKNRRTKAWELAGLGGILINQLMMHLNVTMDLFRFEVNGSSLLNMAALTDLIVKGKVELSPHLYDTLQSNTSVDYSYPTQVAPRCFMIPLDNEISRSLYVFLPFSLTMWLCLLFVLLVVHFVYVRRLIPDGHFWAILGVPGAGQVRYGNRKPVRRFSTFLILFGIFILGQTYSTKLTSSLTVTLIRRPDNSLEELFLLPYRILVLPTDVYAIVDSLGHAEQFSTKFSCTDAENFSQKRISMHPEYIYPISTIRWRFFDMQQRFLRKKRFYFSKICHGSFPYQYQLRVDSHLKDALHRFLLHVQQAGLHDLWLDTCYRKAHRMGYLKDFSTLAELEEKLRLRPLALNLLVPAFSLFLCGMLGSGIAFLVEIRHSFGCRQKPPSINRNPGD

>DmelIR67a

MLPILVPVLLLFNETSWINPILTSIYKDRHHETVLLLQHSQHGNASGLERFPWPVFSFNEQMDFYVRGKYNSEMLVLIWQTGNSDWDLDLWQALDRSLLNMRKVRVLLLRKWEKIPTADVAATAEHLLFLHVAVIGQGNRIYRLQPYAPQSWLQVDPIESPIFIKIRNYFGRYIVTLPDQFPPRSIVYRNPKTDEIQMTGYVYKFLLEFIRIYNFTFRWQRPIVQGERMNLILLRNMTLNGTINLAISLCGFETPSXLGVFSDVYDMEEWYIMVPRAQEISIADVYVVMVSGNFLIVLIIFYFIFTILDTCFGPLLLKERVDWSNLMLNERMISGIMGQSFNMSARNTISSKVTNATLFLLGLVLSTLYAAHLKTLLTKRPTSQQISNFKQLRDSPVTVFFEEAERFYLKHAWDRPIRYIKDQLNFRETIEYNALRMGLNRSNAFSALTSEWMIVAKRQELFKQPIFTVQPELRVIQTSVLLSLVMQSNSIYEDHINDLIHRVQSAGIVEYWKHQTLREMITMGMISQKDPFPYVAFREFKVGDLFWIWLLWVSFLFMSFVIFLCELLVDCFISKTLIRNKRPH

>DmelIR67b

MELLYLNTLQSLSLLEGNRLVQTVQELNNIYQTELNVFLEFGNGADILESAQGTFVPTLWIKNPQNQKVMKGNFTSCTLTILYLEDEHLDRGLYYLANWLWEYHHLEVLIFFNGGSYDKLIQIFSRCFNEGFVNVLVMLPGSDELYTFMPYQDLKILNLKSIKEFYSLSRKKMDLNGYNITSGLVIAGAPRWFSFRDRQNRLILTGYMLRMIVDFTNHFNGSVRLMNVLTVNDGLELLANRTIDFFPFLIRPLKSFSMSNILYLENCGLIVPTSRPLPNWVYLLRPYAFDTWIAWLIMLIYCSLALRILSKGQISISAAFLKVLRLVMYLSGSRDMGTRPTTRRLFLFVILTTSGFILTNLYVAQLSSNSAAGLYEKQINTWEDLDKSDSIWPLIDVDIKTMEKLIPDRTKLLKKIVPTLEADVDTYRRNLNTSCIHSGFFDRIDFALYQQKFLRFPIFRKFPHLLYQQPLQISAAFGRPYLQLFNWFVRKIFESGIYLKMKDDAYRHGIQSGLLNLAFRDRHLEVKSNDVEYYYLIAGLWFGGLTLATVCFLLELLIGYAKIKVTISCKMNIM

>DmelIR67c

MFCWLIFLNIILLSDRSESWSAREVIHQFNHDQQLQLNIYLDCNDVELQIGQEVSNLFVNSTADKMKILGRFSSHSLIIACFKDSTRNRTLNGVKELLWGLQYLPILFVVDSNMDFYFQQALRHGFIHVLALNFMNGSLYTYKPYPKVEVHQIKDMQKFYKLTKLRNLQGQAVRTTVETMTPRCFRYRNRHGQLVYAGYMYRMVKEFISTYNGTEEHVFGNVDTVPYKEGLAALKNGEIDMMPRIIHALEWYYFYRSHILYNIKTYIMVPWAEPLPKSLYFIQPFRGTVWITIMVSFVYASIVIWWIRYRQQGNSSLTQSFMDVLQLLFQLPLSKIWHFNMGTHQVVSFIVLFVFGFMLTNLYTAQLSSYLTTGLFKSQINTFDDLFREKRTLLVESFDAEVLHNMTKEKIIQKEFESIILITSIEEVFKHRKSLNTSYAYEAYEDRIAFELSQQRYLRVPIFKILKEVYDQRPVFVALRHGLPYVELFNNYLRRIFESGIWIKLQEDSFLEGIASGEISFRKSKSREIKIFDKDFYFFAYILLGMGWCVSTIALFLELWSFKYSVTNVLHEG

>DmelIR68a

MRCLWILIVAFISLAMATSIPIPIANPAPLSGYEMQLKILLQKILWVANVKRCFAVITDDLHYPIYDRIFFESVGRRVIPFFVMRTNESDDLQRPSRQVELFVKAIKSSDCELNVITILNGWQVQRFLGYIYDNRSLNMQKKFVLLHDLRLFESDMIHLWSVFIDAIFLKRQLDNKYTISTIAFPGILSGVLVMKNIANWELGKGLNGRILFADKTSNLFGTSLPVAISEHVPMVLWANATKSFQGVEVEIMNALGKALNFKPVYYKPNQTENMDWTELDGGASVAYGSGNPDGYAQNGTHIDSMLVDEVAAHSARFAIGDLHLFQVYLKLVELSAPHNFECLTFLTPESSTDNSWQTFILPFSAGMWVGVLLSLFVVGTVFYAISFLNAIINGNVSSEFFRCLRPNRNVPMDPKIYRRISFRIAISRYRSSKGDRMPRDLFDGYTNCILLTYSMLLYVALPRMPRNWPLRVLTGWYWIYCILLVATYRASFTAILANPAARVTIDTLEDLLRSHIPPSTGATENRQFFLEANDEVARKVGEKMEVFGYSDDLTSRIAKGQCAYYDNEFYLRYLRVADESGSALHIMKECVLYMPVVLAMEKNSALKPRVDASIQHLAEGGLIAKWLKDAIEHLPAEALAQQEALMNIQKFWSSFVALLIGYVISMLTLLAERWHFKHIVMKHPMYDVYNPSLYYNFKRIYPQH

>DmelIR68b

MKFLVGLLLQWYLPGIYALAEIACRIAVEQNVQVTYLYRCASCPASFDADYSALELDLYRCVGSRLPVITRNMEAHELEPFRRTDSLSIFQIPAAEKGDSLVRRILDMLNPHQRRKHMHKYLFVWPNAGRHQLLRLFRGSWAKKLLYGLAITGRENGTFDFDPFAWGGLQVIQRLDGEVPYARKVKDLRGYPLRFSMFTDPLMAMPRSPVETAGYQAVDGVAARVVGEMLNASVTYVFPEDNESYGRCLPNGNYTGVVSDIVGGHTHFAPNSRFVLDCIWPAVEVLYPYTRRNLHLVVPASAIQPEYLIFVRVFRRTVWYLLLVTLLVVVLVFWVMQRLQRRIPRRGVIQFQATWYEILEMFGKTHVGEPAGRLSSFSSMRTFLMGWILFSYVLSTIYFAKLESGFVRPSYEEQVDRVDDLVHLDVHIYAVTTMYDAVRSALTEHQYGLLENRSRQLPLGIATSYYQPVVRRRDRRAAFIMRDFHARDFLAITYDSQAERPAYHIAREYLRSMICTYILPRGSPFLHRLESLYSGFLEHGFFEHWRQMDLITRVGASPDAEEFLEDLGDQTDTDSGSNELAIRNKKVVLTLDILQGAFYLWSVGIGISCLGFAVEHAHWFWRRQTLRNAVEARTS

>DmelIR85a

MSIQWLKHILLLAILVNLAGTRENHIPLDLKKSSIVMVKMSQILCKARIKVLFVYFENQTSHEHTGQILKEVTKCDISNQNTPLEAVKDDGILMYMVMITTNISQPLELSLIRKKSAAKHRSHVFLLVRDADTVSDAWMRASFRQFWKIWLLNIVILYWRDGRLNAYRYNPFMDNYLIPVDNKPNEVPTLEQLFPKTIPNMQRKPLRMCIYKDDVRAIFWRQGTILGTDGLLAAYVAERLNATMMITRPHSYNNHNLSSDICFLEVAKEYVDVAMNIRFLVPDTFRKQAESTVSHTRDDLCVIVPKAKTAPTFWNIFRSFGSLVWALILVSVLVANVFCYILKSEVGRVPMQLFAGALTMPMTQIPPNHSIRLFLIFWLYFGLLICSAFKGNLTSMMVFQPYLPDINQLGALARSHYHIIIRPRHVKHIQHFLTLGHKHESRIREQMLEVSDTQMYEMMRNNDIRFAYLEKYHIARFQVNSRVHMHLGRPLFHLMNSCLVPFHAVYIVPYGSPYLGFLDSLIRSSHEFGFERYWDRIMNSAFIKSGVKVVNRRRGSGNDEPVVLKLQHFHAVFALWLVGIGMACIVLAWEHLTHNYNLAVTKRRD

>DmelIR87a

MSTPEQRFWLAALLFLLSQHSEVRGFGINLMKVQTEDKGQEACILALLRKYFDSGDGLSGSVLCINRNYQLPNIEEQLLRGVNNYENYPWSLLITNSREGPSPAKFLMNEKPQCYFLIVDNLEDEDLDEVFEHWKGMVNWNPLAQFVVYLASLEETDEEMNDLMVELLLTFINKKIFNVNVIGQSEENQFYYGKTVFPYHPDNNCGNRVISVELLDACDYPSEETDSEDENDEDEGDGAQEEDDGPQEEGDGEQEEEDGPQEQEDGDQAKGDEGQENDDGGLENKVENEFRIGASDDDELENDLSSNSSEPEAIIEEFFRAKFEDKFPRDLSGCPLTASFRPWEPYIFRNSEEQPVDDYYYGLQGDEDDYNDTSPNYGESDDESYADPGEDGDGAIPDTETQSGGKLKLSGIEYEMVQTIAERLHVSIEMQGENSNLYHLFQQLIDGEIEMIVGGIDEDPSISQFVSSSIPYHQDELTWCVARAKRRHGFFNFVATFNADAGFLIGIFVVTCSLVVWLAQRVSGFQLRNLNGYFPTCLRVLGILLNQAIPAQDFPITLRQLFALSFLMGFFFSNTYQSFLISTLTTPRSSYQIHTLQEIYSNKMTVMGTSEHVRHLNKDGEIFKYIREKFQMCYNLVDCLNDAAQNEHIAVAVSRQHSFYNPRIQRDRLYCFDRRESLYVYLVTMLLPKKYHLLHQINPVIQHIIESGHMQKWARDLDMRRMIHEEITRVREDPFKALTFDQFRGAIAFSGGLLLVASCVFAFELCYVKYVYRTEKRERKTKKITKKVHNIKIQHD

>DmelIR94a

MALPKQLKFINIFLVLLIIYGSSDGTENQHEIFLNRLLQAVHNERSVETLFLLHHSNLANCSLQDWNPPRIPTIRSNELTVFNVEKTFNHNALALVCLMKNSYREILNTLAKSFDCMRQERIILMIHRKSDSKFIEDITHEVKNLQFLHLIVLIVQEKYNGQVFASTLRLQSFPEPHFKRIRNVFAIQRIFYRPINFHGKVLNAIPNDIPILFVALNEMFTEYARRYNSTLRIQNRTIKEDIEITEDNYDIDMKIQLHNSQNFLHHMNIAMDIGSNSLIILVPCATELRGLDIFKELGVRTLTWLALLFYIIFVLVEMLFVFISNRFNGRNFTMRYTNPLINLRAVRAILGQTSPISNRYSLSIQHFFVFMSLFGTLFGGFFDCKLRSFLTKRPYYSQIENFSELRKSGVTVVVDHTTRQFIEQEINANFFRDEVPNVRTTTIQELINHVYSYDRKFAFVANSIPWRTFREEMKSINQKILCDSKNLTILENVPLTFSIRRNAIFSHHLRNFIINAADSGMITCWFKMAGKVIRKHIKTTLRESEQQPSHLPLSFDHFKWLWAVLCIAYVMSFMVFVMEILWSKYQRRTRSVSIV

>DmelIR94b

MSLIFNLLFILILSQAVSQETEFLQLKYLNNIVRSMIKLHKMETLVIVKHHLDNNCSLQNWNAHGMGIIRTNDQGKLIMKDTFNSRTLAIICIGQNSHITLLRNVFETFGKVQQKKIILWTQMELKEKFFQEISKKSRDLKLLNLLVLKAVTKDKLLIYRLNPFPSPHFKRIENIWTPNDTLFMDTKFNFHGMTAVVKHDYNWTIQMGNIRKFPISRIEDKEVIEFALKYNLTLQFFNDVERFDIELRKRIILKSNSTQPIDSGIPMVFSSLLIVVPCGNYLSIQDVIKVSGIEKWIFYIILVYVIFVLIEITFLGVTILISRQSRHQMIPNTLVNLCAFRAILGLPFPETRRTSLSLRQLFLAIALFGMIFSIFINCKLSSMLTNPCPRPQVNNFEELKTSGLTVVMDHDAENFIEKEIGVDFFNQYMPRKVTLTFTERAKLLFSLKGNHAFTLFSESFAIIESYQRSKGLRAHCTSEDLIVAERVPRIYILENNSILDRPLRRFIRQMQESGITNHWLKNIPSSLEKNLMQITIPYDRERVHPLSIEHLTWLWCILILGYSISMIVFFVEMSLKRRKKNLENRAPNICIC

>DmelIR94c

MSKVFKLLVLPLIYLSLTKGSKNPQLKFLRELINVIEEGREIRTIMVIKHSRDEYCHLDQWNPRGSPILRTNEMGSIRISGYFNDQAVILACMGENSDYGLLKSLANAMDNMRQERIILWSEREPTKMLMDYISQQADRYNFAQIIIVTMNEDVDAVPSLHQLNPYPTPRFRQITNISNIRRTSFFGCGLSFQGKTAILKESVVSNIRFKVWSPSGPIPLSELKDYEIVQFAVKYNLSLKLYDQNESKSDHFDIQLGPLFITKDFPTQMAFVSPNTACSLIVIVPCSPKWRFMDVLHKLGVLKLIGCLLIAYAVFVLIETLILWLTHRISGREVRLTSLNQLLNPRAFRGILGLPFPEFRRSSISLRQLFLVISVFGLVYSNFVSCTLSALLTKPAQNPQVRNFKELRDSGLITIMDKYTHSFIEKHIDPEFFDHVLPHYLILQKKEALRMIWNFNDSYSYVMYTTTWKSLNTVQKSFDERVFCESESLTIAWNLPRMYVLGNNSVLKWMLSRYITYMPQTGIPDSWTEQLPKVLKLLYNVTSPRRIKEGAVPLSIQHLSWIWHLLFIGESIATLVFIVEILLQKSNQHTSNMRERSSEDDDFV

>DmelIR94d

MGQLHLLLVALVLLSPGGDSFYHSLIHHLNRELKIEYVLLLGNFDTTWLDILWQLPVSVLQIKEHSRETYSLLENPSHNVLTIAFVNDSPEDILEILYRNLRMLNTQPVLLVIRKSTIRVNSLLEWCWHHQLLKVVAIAQDFMESLIVYSYNPFPVLQFIERRLDNSTVIFEKRLENLHGYEVPIALGGSSPRLIVYRDLEGKLIFSGPVGNFMKSFEQRYNCRLVQPYPFDESAISPARDLIASVQNGSVQIALGAIYPQVPYTGYSYPIELMSWCLMMPVPEEVPHSQLYSMVFSPMAFGITIVAMVLISLTLSMALRLHGYRVSFSEYFLHDSCLRGVLSQSFYEVLRAPALIKAMYLVICLLGLLITSWYNSYFSTFVTSAPRFPQLTSYESIRHSNIKIVIWKPEYEMLLFFSENMEKYSSIFQLQEDYKEFLHLRDSFDTRYGYMMPMEKWSLMKEQQRVFSSPLFSLQDDLCVFHTVPIVFPMVKNSIFKEPFDRLILDVTATGLLSRWRDMSFTEMIKAGQLGLEDRGHPKEFRAMKVGDLIQIWRFVGWMLGLATIVFLLELICFWRHKMWQNMKYMFCRNKNI

>DmelIR94e

MDCPKWILSGLCLISLVSGATVIELLGTLKLELDFEYVLLMKNRNFSLSDQVWNGTSLTKDVMDEVQVPVLQFNENVSYFLHNSISRRLVTLGFMSDANLDEHRGLLTALVANLRHMTTSRVIFLVQSKASTDFLYELFRNCWRKKLLNVIVIFQDFETTSTFYSYSNFPILQIEERIYETSLQTLPIFPDRLRNLHGYEMPVILGGTAPRMIAYRNKKGNVVYDGTVGHFMTAFQQKYNVKFVQPLQAKNPLDFAPSMQTVGAVRNETVEISISLTFPTIPPFGFSYPYEQMNWCVMLPVEADVPPFEYYTRVFELAAFLLTLGTLVLISCLLASALSLHGYATNISEFLLHDSCLRGVLGQSFVEVFRAPTLVRGIYLEICVLGILITAWYNSYFSSYVTSAPKQPPFRTYDDILASKLKVVAWKPEYAELVGRLLEFRKYETMFLVEPDFNRYLALRDTLDTRYGYMITTNRWVLINEQQKVFSRPLFQKRDDFCFFNNIPFGFPLHENSVFMEPVQKLIMELAETGLYYHWITTGFSELIDAGEMHFVDLSPHREFRAMQIQDLQYVWYGYAFMVVLSSLVWLLENLAYTVKSKTIFPTHFMQRNKK

>DmelIR94f

MSGMWQQVLLAETSNWFRSDVLQRFWTHLRVEIRFRTMLNYRLESCDCWFDNVLGSDNSTALLWNDQTYPHYLRRRQDTDILVVSCLRFHQYQEVLLALSLMLDQMRSMPVVLQLCGDEDSMQELNSARLLLKHSQDLKMPNVVLLSSTFFTSATLYSYEMFPEFNVQKLVYQAYLTLFPYKLGNLKGHPIRTVPDNSEPLTIVRKTLNGSIAIDGLVWQFMIEFAKHINATLQLPIEPHPEKSIKLVQILDLVRNQTVDIAASLRPYSLNVQRSSTHIYGSPMMVGNWCMMLPTERVIGSHEALTRLMKSPWTWLILLLFYSVHRFLAQKTRLRSSLIHLIKLLINLSLICFLQAQLSAYFIGPQKVNHISNMQQVEESGLKIRGMRGEFMEYPIDMRSRYASSFLLHDLFFDLAQYRNSLNTSYGYTVTSVKWELYKEAQRHFRRPLFRYSEEICVQKLSLFSLIQQSNCIYCYRSRIFILRMHEAGLIRLWYRRSYYVMVTAGRFPIGDLSTVHRAQPIRWTEWQNVVLLHGVGLLFSVVVFVIELTVHYANVCLNNL

>DmelIR94g

MSTAVNSVHSKLVSLISRGQELTSIFFYAPAKEKCHLEDTISSATWGLPLVIWRTDRTVILNGFIGEGLLVLACLPGFHWRALLGSLARSLKYLRQARILIELMQDRDEFLVSEVLQFCLSQDMINVNAIFDDFPETENLSSFEAYPSFEVVNQTFTPDTQVSDLYPNKMLNLRGGVIRTMPDYSEPNTILYQDKEGNKEILGYLWDLLEAYAHKHNAQLQVVNKYADDRPLNFIELLDAAQSGIIDVGASIQPMSMGSLSRMHEMSYPVNQASWCTMLPVERQLHVSELLTRVIPYPTLALLLLLWIFYEVLRGRWRRHSRLQSIGWLVLATLVSSNYVGKLLNLFTDPPSLPPVNSLAALMESPVRIISIRSEYSAIEFTQRTKYSAAFHLALHASILIGLRNAFNTSYGYTITSEKWKIYEEQQKRSSKPVFRYSKDLCFYEMIPFGLVIPENSPHRAPLHSYTLLLRQAGLHDFWVNRGFSYMVKAGKINFTAVGERYEAKTLTITDLRNVFIIYVSVLLISLILFTCELFVSWVNYWLGF

>DmelIR94h

MLSNISFSSAPELVDLYGLVLKFLVSSETTLFYFNPTGQKCSWETLPRTILSNHPQIIWFREETYPGLYKRHSSNLFVMACLSSTSYDGQLQLLAESLTRYRSVRVLIEVQDKEGSFLASQILLLCQQHSMLNVVLYFSRWTRTLNVFSYLAFPYFKLLKQRLSGSLRPKIFINQLKDLQGYKIRVQPDLSPPNSFSYRDRHGECQVGGFLWRIVENFSKSLKGDTQVLYPTWAKAKVSAAEYMIQFTRNGSSDIGVTTTMITFKHEERYRDYSYPMYDISWCTMLPVEKPLSVEILFSHVLSPGSALLLILAFILFFLIVPQLIKCLGITFRGRLIGMASRIFALVMLCSSSAQLLSLLMSPPLHTRIKSFDDLLTSGLKIFGIRSELYFLDGGFRAKYASAFHLTENPNELYDNRNYFNTSWAYTITSVKWNVIEAQQRHFAHPVFRYSTDLCFSSETPWGLLIAPESFYREPLQHFTLKINQAGLITQWMTQSFHEMVRAGRMTIKDYSRTNLMKPLRIQDLRKCWVIFAVGLGTSTVVFTIELLLIYTNVFLNSL

>DmelIR100a

MATTLQLIMLALVGGTLGQANNTDHKQVLTSIVKQLEGGLELHLRTSEDGGNDLVQFLMQEKSSIIISAKQEEVPSRAKIMRHHFFIFDGVHQMQEIRTSLFNTDGFYILALENNTIEDDVLLMEFAADVWLQHGHSRIYYVQLSKKSVLLFNPFLQRLVVVQDSKTYSRIYKDLEGYHLRIYIFDSVYSSVIGDGENKVLSVTGADAKLAKTVARQLNFTADFVWPDDEFFGGRLANGEYSGGVGRAHRGEVDIIFAGFFIKDYLTTHIQFSAAVYMDELCLYVKKAQRIPQSILPLFAVHMDVWLCFLLVGLLGALVWLILRAVNLILGIEGVPDGSRATRISYFGAARRIFVDTWVIWVRVNVGRFPPFHSERIFVASLCLVSVIFGALLESSLATVYIRPLYYRDVNTLRELDESGQPIYIKHPAFKDDLFYGHNSEVYRRLDAKMMLVAEGEERLIEMVSKRGGFAGVTRSASLQLSDIRYVMTKKVHKIPECPKNYHIAYVLPRPSPYLEEVNRIVLRLVAGGIVGLWTGEAKERAKWSIQRFPEYLAELDVGRWKVLTLSDVQLAFYALTIGCLLSAIVCMAEILLGRQR

>Apis_XP_001946588

MAGPRITRSFVLYCVAFAAAATASRADGIVYEKIPLGAIFEQGSDEVQTAFKLAVFKHNQNNTERRFELQAYVDVIKTADAFKLSKLICIQFSRGVYSMLGSVSPDSFDTLHSYSNTFQMPFVTPWFPEQVLSPSSGMLDYAVSLRPDYHRAILDTVRYYGWTNIIYMYDSHDGLLRLQQLYEALDVGPNSLKVDMVKRIQNVSDALNFIHQVERVNRWGSKRIVLDCPTLMAKQIVVSHVKDIQLGKRTYHYLLSGLIMDDHWETEVIEYGAINITGFRLLDMNQWSVKHFLSEWKNLDPLTSPGAGKDTISAQAALMHDAVLVLVETFNKLLWKKPDMFKANTKRTLSNSNSSMSGISSSQILGLDCNSGRTSGNQWEHGEKISRFLRKVVMEGLTGHIMFNDDGKRYNYTLHVVQMTIDSTITKIAEWSDTDGFKTVVSKPERVHTLGHSHKGNATLIVATLMEEPFTMFKKPKYGESLAGNDRFEGYCKDLAILLADKIGVK

>ApisIR21a_XP_001950972

MHPKLDYRQLRPWSCQLPLYHRVPDRLLPPGAPVKDNGIETSLSVVSLSLPRDVLQTMLPSLDTISYKLFNVYEYTSKRTRVSESKCVNYIIFCQDPEITATKFGNAIDSRVFIVTIGSKWQIQEFFKSPASQNIMNLLIASAGPALQEKNKNIKSRSYHVLTGVKANRQNSIGNTKEILPVSVMRHLYDTTEPDIEVADIKLYTHEMYIDGLGSSAQTILTTWKLNKFTRPEVDLYPVKLVNGFRGHRFILSAIENPPLVFRSPLSVDKVLTQEQSTSWDGVEIRLLQIISRVLNFTLGIHDATFSKSRGEGDDRIIGDLVASKADIGISGLYMTNARYSLVDFSPVILQDCGTFVSLGSFALSKYRAIFGPFHWSIWVMVVITYMVAIFPIAFTNNRNVGTLCKSPKQLESMCCYMFGTYTNLFTFKDVKSWTNTKMGSTRLFIGTYWIFTIIITTAYTSSIIAFITLPEQPVIVDKSYQLVDQGYRVMTLDKGGWQHYLNITNDTMSRRLLSNIKLMKNLDDAIDYIVRTRFILDYAFLGSKISLTYLSQNNFIQKYNNKKIFLHVASECYVPFNIGIAYKKNFVFRNIFSNFILRAQQSGLVTKIIKDIEWEITQKSGVRNPNLIIAPEDRQLALDDVQGMFVLLGGGIFLAAFTLSIEFVKCKRENRKIAQIKIEKHLKKKKRSKSLTVRGNTTVEPFRPLTAF

>ApisIR76b_XP_001952331

MALTNTLLLALCANWPPSNITETTLPPPWKWISEHKAKFINSSEYQCDLKNNFQIQYEILRGKRLKIATFPNSKPLSWVTKEINGTLIGHGIAFEIVETLRQRYGFTYDVVVPTRETLLNENGSIIYMLVNGEVDMAAAFIPVLPGLDDIVKWGIDLTQFQYVVLMKRPKESATGSGLLAPFEMEVWLLILMSLIAVGPIIYGIMMLRHKLCGHDSGIIYSIPTCVWFVYGALMKQGSSLNPDTDSARLIFATWWIFIMILTSFYTANLTAFLTLSKFTLPIKRIDDIASNEYRWISSEGSAVEYIVKVDNDLKPLKQSMFEGNGTLLLHDKNWLNFLMLRIERSITDDKERCRYVLTTDPYLTRSMSFVYPKHSILPPLFNPIMLSYMESGIVRHLQTKDLPEAVICPLNLGSKERQLRNSDLYTTYAVVVCGFSMAAAVFTLELLSTRTGWFVAADLARRSHDPLRSGAVRCEISVFGQPSKAMYPFSHRDNVMNAASATHFPFRGSSSSTSLPSSSSSPFLLLDGKSSLRRFQY

>Apis_XP_001943685

MCCCGRYPPSSRWPSVGRADASAVRSGGGGGEKDRSGASGGAVGGGRWKHKGVGVGLGGGGGRSSHRRPSAGDRRPEMALNVGMLMPKTSFGVRGYLRAIHDAIQGINKAYKKNHSTNFSKIYDFEERNVRSQMMSLTPSPTAILDSLCKEFLNMNVSAILYLMNYEKYGRSTASAQYFLQLAGYLGIPVIAWNADNSGLERRASRSSLQLQLAPSLEHQTAAMLSILERYKWHQFSVVTSQIAGHDDFIQAVRERVADMQDRFKFTILSSVLVTKNSDLQQLVASESRVMLLYCTREEALRILQAARGFKITGENYVWVVTQSVMENLQAPQLFPVGMLGVHFDTSSDSLVKEITTAIRVFAHGVEDFVNDPENINETLTTQLSCEGEGESRWVTGEKFYKYLRNVSVEHTEPAKPKVEFTADGVLKSAELKIMNLRPGVGNSLVWEEIGVWKSWEREGLYIKDIVWPGDSHTPPQGVPEKFNLRITFLEEAPYITLAPPDPITGKCSMNRGVLCRIARGKDTQGLDVIEAKRNSSYYQCCSGFCIDLLEKFAEDLGFTYELVRVEDGKWGTLENGKWNGLISELVNRKTDMVMTSLMINSDRESVVDFSLPFMETGISIAVTKRTGIISPTAFLEPFDTASWMLVGVVAIQAATFSIFAFEWMSPSGFDMKVWFYESVSQAPNHRFSLFRTYWLIWAVLFQAAVQVDIPKGITSRFMTNIWAMFAVVFLAIYTANLAAFMITREEYYDFMGIDDYRLSKPHSHKPMFKFGTVPHSHIDSTIHKYFPEMFSYMKSFNRSTVQEGLSSLLTAELDAFIYDGTVLDYLVTQDDDCRLLTVGSWYAKTGYAIAFQRNSKYVQMFNKRLLDFRENGDLERLQRFWMTGTCKPDDHDQNATSDPLALEQFLSAFLLLMGGTLLAALILLLECLYFNFFRKKLAKTDQGGCCALISLSMGKSLTFRGAVFEAQDFLKFRRCKDAVCDSQYQLLRNQLMSTYSKMKQLEKELELRKLKSEKRYQFSPASPLRQRDLVRPSIISTEYTNRYDHEPV

>ApisIR93a

LYIVLRLKYHKNITDGKPTIFLPLKFKFNNISIYFYNKFKRXRRRRVRICCLRLTHLSFRQISFTNFNSILVCYTDCPRLPTDEAITIPLTVHHSELSQMILDLRMSNAFSWKSAVLMHDNSIGDSVLQHIVTSLTKYYPSNIMSPSITIFEIYTQGSEWKRRKLFMEDLQHFLKMSEINSNYICIVSILYVPLILDVAKSLNLMTAENSWLIIIPDIDSSRNNTSSFTNLLSEGENISFIYNSTKTGSKCIVRILCLVDELMSVFIMAFSALIQQEIELSQRVSEEEWDEIRPSKIDRRQSMVSFIKFRLNESGVCETCPLWQIDSGVTWGQEHFGQGCYILPVGNWNTKTGLKLTEPLFLHLANGFRGIALPIATFNFPPWQIVNFNRSGHLIGYSGLVFDIINQLAKTLNFTYNVIVISNTEQMNTTRTLFMQNNVLGEHDAVVSKPLWDKMIDLVRSEKVFIAAAAFAVKEANQILVNYTTHISLEPHQILVARPKELSRALLFTAPFTLLTWLCIAIVVGLMGPLLNVFHVLSPYYEYHNIPRRGGLNSPLNCFWYVYGALLQQGGAHLPDADSGRLVVGTWWLFVLVIVTTYSGNLVAYLTFPQMDSMVSNVADLMARKPQGYSWGIPKTSNLHSLLTTLPDDTMVKELIKNAEHHEELSRSIIERVRSGKHAFIHRRTNLMYIMKNDFLKTNRCDFAIGNEDFAEEKLAMMLSKESPYLSRINREIEKMHKVGLINKWLVDTLPKKDQCWTNTQLEVTNHKVNLDDMQGSFIVLLLGVLSSLVSFVFEYILHKYINRRQIVITPFIN

>ApisNMDAR1

SPNLWIAIFVSLHVTTVAVALYEWFSPFGLNPSGRQRSKNFGMPSALWAMWGLLCGALVNFKAPKSWPNKFLINVWGGFCVIFVASYTANIAALIASLLFQNADVDYNDRNVNTQLNQTLSLRFKNIYHFDFALCKTVMNIIKSVLNLFAFVLHFRNGSLDIFIGDKPILDYYSGTDHDCKLQPYGDPLYDDVYAVGMTKNFVLKEKVSAAVSTYTNNGFMDILQNKWFSDLPCVNRELETSDIGQPTPLGVDAFLGVFLMLGCGILAGAVILCLE

>Apis_XP_001949860

MKPVFSVFLVFLFSIFYCKSEITEKENTDNNPYEFHIGGVLSSNASEGYFKQTIEHLNFNTPFMKPGNTFYAHSIKMDPNPIKTALNVCKQLIVRRVYAVIVSHPQIGDLSPAAVSYTSGFYHIPVIGISSRDSAFSDKNIHVSFLRTVPPYSHQAEVWVELLKYFNYLKVIFIHSSDTDGRAFVGRFQTTSQNQGDDIEKKVQVEAVIEFEPGLFHFNNQLNEMKNAQARVYLMYASKIDAEVIFRDAAHRNMTEAGYVWIVTEQALDANNVPEGTIGLKLVNASNELAHIYDSIYILASAITDMNRTKTITPPPADCDNSGAIWDTGKTLFEYIKKQVYKDGHTGKVAFDNNGDRIYAEYDIVNVKEVANKDAIGKYYFNNDLNKMKLRLNESNIIWPGRLKKKPEGFMIPTHLKVLTIEEKPFVYVRPLKKDDGNSCKSDEIMCPLYNTSSKVYCCKGYCIDLLVELSETINFTYSLSLSPDGQFGNYEIRNNSASGKKEWNGLIGEIVYERADMILAPLTINPERAEFIEFSKPFKYQGITILEKKPSRSSTLVSFLQPFSHTLWVLVMGSVHVVALVLYLLDRFSPFARFKLINADGTEEDALNLSSATWFAWGVLLNSGIGEGTPRSFSARVLGMVWAGFAMIIVASYTANLAAFLVLERPKTKLSGINDARLRSTMENLTCATVKGSAVDMYFRRQVELSNMYRTMEANNYETAEEAIRDVKNDKLMAFIWDSSRLEFEAAQDCQLVTAGELFGRSGYGVGLQKGSPWSESVTLSILDFHESGFMESLDDKWIFQGRVEQCEDQEKTPNTLGLKNMAGVFILVAVGIVVGMVLIVIEIGYKKHHVRKQNRLQLARNYGQTWRAIVQKRKMMRMQGRTSGVGALSLSVEALPRSVLNIQSPIRSIETLPRTCRNSPSPTRAWSGRQMVHRKSDDIPLRSPIGGPPQLHFHNNLM

>ApisIR25a_XP_001945524

MYKPIRGITILLWINTLFNIGTSQNVQTVNILFINDRTNEVAEDTLNVALNYIRRNPRVGLMIDGLYSVKIGGDDASAILETLCVNYNASIRNNKPPHLVIDTTINGVASEAVKSFTAALALPTVSASYGQTGDIRQWRNLDGEQQKYLIQISPPADLIPEIVRSIVVAQNITNAGIMFDDTFVMDHKYKSLLQNIPTRHIIAAIDDTTSIKLHLTRFRDVDIVNFFVLGKLSIIKSVLDHANSNKLFGRKYAWHVITQDKGSLKCGCSNATILFVKPEPDAGSRERLSNLRTTYGLTSTPELKAAFYFDFYYRSLLAIRSMMNSGEWPTNVTYTTCDEYNEENPLPRRNVDLRRYLKDMTEPPSYAPFLIDTNGHSYEEFTMRLEKVTVLNSQSVSAENVGSWKASLNSPIIVKDAANMTHFSAVTVYRVVTVLQNPFMIQIDDEDGKGVKFKGYCIDLIEEIRKLIGFEYEIYIAPDNNFGNMDENGQWNGMVKELVEKRADIALGSLSVMAERENVVDFTVPYYDLVGITILMKKPQTPTSLFKFLTVLENDVWMCILGAYFFTSFLMWVFDRWSPYSYQNNRIKYKDDEEKREFNLKECLWFCMTSLTPQGGGEAPKNLSGRLVAATWWLFGFIIIASYTANLAAFLTVSRLDTPVESLDDLSKQYKIQYAPLNGSSAMTYFQRMADIETRFYEIWKDMSLNDSLSEVERAKLAVWDYPVSDKYTKMWQAMKEAKLPNTLEEAIERVQSSKSSSEGFAYLGDATDIRYQVMIDCHLQMVGDEFSRKPYAIAVQQGSPLKDQFNNAILLLLNKRKLEKLKETWWNLNPERIQCEKQDNQSDGISIHNIGGVFIVIFVGIGLACFTLAFEYWWYKYKKSSRIIDIAMVINENCIYPIGYISVGTNLMANIQRVMHIGY

>ApisIR8a_XP_001942846

MLQNLGLQIEHSTLAFGPTLGANTTNNKCRTRRVWALGVYENKNEFQYDIDTNLMTYNERAMILKVSPHGVPNQLGTWTLNGGLEMKYDASTVVNGRRFFRVGTAKSIPWTFMEDVWKGYCIDLIEKLSKEMNFKYELVVKDKFGSLDPVTNQWNGLIGGLVEGELDIVIAALTMTSEREEVIDFIAPYFEQTGISIVIRKPSRKTSLFKFMTVLKPEVWLSIVAALAMTAVMIWVLDKYSPYSAQNNKTKYEQFRHFTLVESFWFALTSFTPQGGGETPKAISGRVLVAAYWVFVVLMLATFTANLAAFLTVERMQTPVQSLQQLARQSRINYSVIDGSDAHHFFRNMKMAEDILYNVWKEIALNQTNNRKDFRVWDYPIKEEYGQILAAIERTGTVPNRSVGYQMVLDNEQGEFALIHDSSDIEYEVYNNCNLTEVGEIFAERPYSIAVQQGSLIQEEISRKILDLQKDRFFELLNAKYWNASKVSMCPNADDSEGITLESLGGVFIATLVGLLIALITLAFEVVYFKHKRAKVAEVSVVNNTVHKDKLMYELSNILYITKLKYKIKIQLKNSICRYHKTYDIPCFPKKFIIVSKSQ

>Apis_XP_001952842

MGYDTEVDLAIAPLTITAEREKVVEFSKPFMSTAINILIKKPSKHKPGAFSFLSPLSREVWISVGFAYVAVVIVMYVVSRFSAHATAAAHVRPVHNSYGGGGGIGIGSGGGGLGHELQQTIPPPPMFVHNPFTLANSMWFAVAAITQQACDIAPRTTPGRIVGAVWWFFTVILISSYVSNFTAYLIVQKMSTPISTPEDLAAQTEIEYGVLSHGSTWDFFKRSHSSLYSKMWQFMNSRTHVFVDTYDEGIQRVRNSNGKFALLIESPKNDFVNMRKPCDTMKVGRNLDVKGYGIAMPLGSPLRNRINAAIVQLTDSGELTRLENKWWVDECGYEAKESFRNAELTLNNIAGIFYILVLGLIVAIAVALFQFCYRTHSDQANSKDELEQAQSSTNASVVSKSMFVDVGVQTKLTGEELDKTIQDSRREKKILQQKLIRRNNRVNVALIYDAMSIRDGFWPDQSGKVYGYCDLCGIAHTDTEELAKESLVFIISSLGKKFKCPVAYFFINKINATVQSQLVLAVISALYEAGIIVRSLTSDGTSTN

>Apis_XP_001943239

MQSAATAVVVLFLLSGFADCAAEDVERITIVGLFPSEDSVEQMAFELAIHKVNLDPTLSNDVKLEGRVEIVDIDDGYQTSKIVCESLESGVGAIFGPAGYESSVIVQSICDSMEIPHIETHWKMNLRQQPNYYINVYPDPVVLSRGYTAIVRDMDWTSFTLLYQRDEGLARLQHLIQDYSGLTKLSDTELSAITIIKLSENYDYRPMLKEVKKSLESHIVLDCDTDIILTVLGQAEDVGLMDDYHSFIITSLDAHTIDYGHLQFKRTNITAVKLIDPSSPTVTNIMADLEFVQQRMNLNMEVFRAETITVNALLMFDAVNVYAKALRGIGGTKAIKAEPNSCANRSITGWSSGFSLINFMRVETDGLTGKLRFDQDSGYRNYFTLEMVELTNTGFKKIGVWDPQKEMSYTRTRNQMLDDLVNANMNKTFIVASKITEPYMMLKEDHKNRVGNDKYEGYVVDLIHMISEEINITYEFKLRNDGNGKKDKKTGKWDGLIGEVHELRADLAICDLTITFDRRNAVDFTTPFMTLGISILYAKPEKKKPQLFWFLNPLSFSVWMYTATAYLGVSLFLFMLARMTPFEWELPHPVKPGDDTLENSMTLLNCLWFSIGSVLCAGCEVLPKAVSTRLVAGMWWFFALIMTSSYTANLTASITSGRLDTPIKNVDDLSKDSNIEYGCYEEGSTASFFQKSNLSLYQRMWSVMEASNPTVFTKSNQEGVDRVLKGKGRYAFLMESSSIEYQTERNCNLMEIGNTLDSKGYGIAMPMNSPYRTLISESVLRLQESGFMRELKDKWWKVQGDNKCEEEDESDELGFTKIGGVFVVLVLGCLIAFMFSILEFLWNIRKVAIEEEITPKEALILEWKFAMKCDGGVKPLRRRHHIDTGTNNSDTS

>Apis_XP_001943184

MFVSYDFVVIICLCNVVAQENDNIPLSIVGFFDKENSIEQQTFELSIQKVNIDPKFANVYLKAEIKIIDSTDTYMTGLKVCETMESGIGAVFGPSHLESSNIVQSLCETMEVPRIDTSWENTPVENFNFYFNPHPEPTLLAKGYTAIVHDMDWKSFTLLYQRPECLQRLQDLIQDYSGRTKPNDKQAAAISIIQLPEGNNFRPILKDIKKSLEGHIVLDCDADLILTVFKQAKEVNLLDDYHSFIITSLDAHTVDFSSIVQNLRTNITTVRLIDPLSPFVENIVRDLNFVQQRMNLNMEPLKADKLTVNAILIYDAVNVYAKALKGLGMTNKIITEPLQCTDSPFIPWSNGFKLINFMRIETEGLTGLLRFDNKTGHRSYFTLEMVELVDTGFKKIGLWDPERGMTYTRTSLEMLRDLYAGSKNKTFIVSTKILDFFDNFVRLLVDIYYLFLYKMDNNPGPSGISKSPPIKKKRLTDQEIFNLIENPLSSDEDFDLSSDTDLGEGDSADDDVVFEEADNDVDEMVSDDIDDNNVNVLDDAVIEEVSPLRLEEENSETGTLSPSLFSYSPPTIRDRVIRQKTIRVPTTNYTWLDNPPNLDTIDFNENVGLKINPDGDQPIDFFNLLLTNDFFEFVVKETNNYATDLYMNRSSDKSRIVKWIDIDVNELKIFFGLLLHTGTIKMPKMSDYWKTDELFNLNCFREKMSRNRYMLIMRTLHFCKNPEAGEKAPSRIYKVEKVLDYFNKRMQDVYQPSKNLSLDESMVLWRGRLVFRQYIKNKRHKYGVKLYMLTENQGFVQQIMIYSGQGTGVLPGVSHTEYVVDQLMNNYFDKGYSLFMDNYYNSVQLAHKLLQRKTYCTGTLRSNRKGNPKEVVTKNLKSGESVGKYTKDGICVVKWKDKRDVLSISSEFKHEMVETANRHGKKKIKPLQISEYNKNMSGIDRQDQMMSYYPAELIKALVSNKADPVPRLCPQIPERHFPCKVEKNHKKRYIRKRCRLCASRADLGVCDLTITHERRSAVDFTMPFMNLGISILFSKPEEPQTNLFSFTQPLSFQVWIFTATAYLGLSLVLFFLARITPNEWQNPHPCNPHPVELENSLSLLNCLWFSMGSILCQGSDILPRAFPTRLCAAMWWFFALIMTQSYTANWTAFLTSNRMETTIKNVDDLDKKGGTDGIKYGCVTDQSTASFFQNSDVNLYQKMWSVMELNGDSVMVSDNKQGVDRVKKERNHYAFFMESSSIEYEVQRNCDLTEVGYWLDNKAYGIAMPFNAPHRTLVNMAVLKLSESGALMNLKNRWWSVSDDKRCKDLKKDSAELDVNEVGGMFVILILGCLIAFLFSILEFLWNIRKVAVEEKLTLWEAFMVELKFVLKCHGTSKPVRHVEDSSSDVTTK

>Apis_XP_001942991

MKFEIFVWIIHFASRVSTLPDTIRIGGLFHPNDVNQENVFKHAIHDVNANRNILSRSNLSGQVEKVSPQDSFHASKRVCSLLRLGVAAVFGPQSAQISSHVQSICDTMEIPHLETRWDYKLRRESCLVNLYPHPTVLSKAYLDLVKKWGWKSFTIIYESNEGLVRLQELLKARNGALSAYPITIRQLGSGRDHRPLLKQIKNSAESHVVLDCSTEKIYDVLKQAQQIGMMSDYHSYLITSLDLHTIDLDEFKYGGTNITGFRLVNPDTPVVQKVLKQWGENFTVMSTETALIYDAVHLFARALHDLDSSQKIDIKPLSCDASDTWSHGYSLINYMKIVEISGLTGVIKFDNQGFRTDFELDVVEVNKEGLSKIGTWNSSQGINFTRSFVEAYSSIVDNLHNKTLVVTLILSSPYTMRRESSQKLVGNDQFEGYAIDLIYEISKLLGFNYTLKLVPDGRYGSYSEDTKEWDGMMGELLQQRADLVVADLTITYDREQAVDFTMPFMNLGISILYRKPIKQPPNLFSFLSPLSLDVWIYMATAYLGVSVLLYILARFTPYEWYNPHPCNPDPDNLENQFSLMNCMWFAFGSLMQQGCDILPKAVSTRIVAGMWWFFTLIMISSYTANLAAFLTVERMDSPIESAEDLAKQTKIKYGALRGGSTAAFFRDSNFITYQRMWSFMESSRPSVFMASNNEGVERVVKGKGNYAFLMESTSIEYVIERNCELTQVGGLLDSKGYGIAMPPNSPYRTAISGAVLKLQEIGKLHKLKTKWWKEKRGGGACRDDTSKSNSAANELGLANVGGVFVVLMGGMGVACVVAVFEFVWKSRKIAVEERTKAYIALYSKFAHSNSNTLTLGQQF

>Apis_XP_001942939

MNAIVGLLVPFVLILDGGKLCAGDTTITLGILYNEENSMLETAFKSSVDVAKAKMIASGVELDVISKIVPLYDSFETQHHVCEMLAEGVTGMFGPSAGDTAPIVQSICDYKEIPHIQTRWDINQKRGSCQINLYPHPSTLAEALIDIIIAVDWKSFTIIYENNESLMQITNILKSPPTNHPIRIRQLSPGPNYRKELREIKDSGETKILLDCSFGILSEVLLQAQQVGLMGSEHNFIIASLDMHTLDLDAFKYSGTNITGMRLVKPLDSEFQDTVSQWTDNLSPLEPDDKTVLPETIQLESALIYDAVQLFTTSIYNLSKEFEINETPTPCNSSLSWKHGFTLINYMKMAVNFKGLTGKIKFDQEGFRTDIELELVDLTQNGLRVTGTWNTKTGINVSATPKSQTVPGGKEFDLRNMSFVVITALTKPYGMVKLSSNTLEGNDRYEGFGIDLIKELSEMSGFNYTFIIQEDFNSGYPDEKTKKWNGMIGEVINGKADLAIADITITRQREHDVDFTSPFMNLGISILYKKSTKSSPSLFSFLAPFSSFVWLWVITAYCGVSVLLFIMARISPYEWTNPYPCIEEPEYLENQFSLSNAFWFTIGSLMQQGSDIAPIAVSTRLVAGIWWFFTLIMVSSYTANLAAFLTVESVSEPFKNVEDLVNNQNIITFGLKKKGSTEEYFRESTNPTYKKIFDILQKNQAWYTTSNDEGVDKVLRENYAFFMESTSIEYMVERNCKLAQIGGLLDNKGYGIVMKKNSSFRNVLSANILSLQEKGKLTALKNKWWKEKRGGGACQDTDNNEASELSMKNVGGVFIVLCSGVVVAAILAAMEMFWTLWKTTSKEKVSFKSEFKDELKFIAKCRGSTKPARRKQNSSADNSNPTGSNQYMNSDRHYDYES

>Apis_XP_001945266

MFRAIFTQEDKDSTTEVAFKYAVYRINKDKAILPNTTLVYDIEYVSPHDSFKASKKVCRLLESGVHAIFGPSDSHLASHIVSICESVSMPLLLTIADRVTDLNRRNFVTDMFPARNHLGQAFRDLINFLNWTKIAIVYDDEEGLLLVQNLMKMSKTDFYVRQVDQHTHRQVMREIKDKHIFNIIVDVHPRNINGFFRSILQLQMNDFRYHFLFTTFDLETFDLEDFKYNSVNITSFRIVDDENHRVVNILREMERFQRVGQNMLHKSGIIRAEPALMFDAVNVFAHSIGSLDGSSNSIQSSNVSCKTSDRWANGTFLYDRLNAVSIEGLTGKVHFDEGRRSDIKLDLLKLHQEQVKKVGFWTPSTGINITKHSVFYGHQSSNVTLIVVTRVEKPYVMIKEDKNLTGNSKYEGFCIDLLHRIASQVGFHYAITLVPDNKYGAYDPTTKQWNGIVRELMDKKADLAMGSMTINYARESVVDFTKPFMNMGIGILFKVPSNEPSKLFAFLNPLATSVWSFMFLAYMAVSFSLFFLARFSPYEWRPHTEENYREGRFTISNCFWFVAGVSLKQDAGITPKATSARILGGIWWFFTIIIIPSYTANLTALRTVERLQKPIQNVAELSSQEKITYGTLEGGSTMSFFRDSKIPIYQKMWKFMEKHQSVFVSTYDEGTKKVLGGNYAFLMESTMIDYAVQRDCNLTQISGLLDSKGYGIATPKGSVWRDKLSLAVLELQEKGVIQMLYDKWWKNAADICIKDEKVKEFKPKPLDLNDLGGVFVFVLCGLTVAVIIAILEFCWHSKKKSTDQQLPVCTEMAEELRYAIRCDGTKHKATLKRTCNGCSPITTYVPAPMHINHVTSQIDNVPMIELTRPSISVDHEDK

>Apis_XP_001949419

ALFTHDQINSSLEIAFKFAVDQINRDAHLLPHTQVSYDIQYVPHDDSFYTIKTACSQLDLGIAALFGPSNSYLASHLQSMCDALDLPHIEARLDLEMDPKRFSINLYPKQSLLNKAYSDVISFLNWTKMAIIYEHDYDLFRMKDFMKTVPDRKTVEVYLRHATPETYRSVLNEIKNMEIYNMLVDTKSEHVDHFLRGILQLQMNDYKFHYLFTSFDMEMFDLEDFKYNFVNLTTFRMVDIEDVGVREVLKHMEQYSTEKNQSINKFMLTQCEPALMYDSVQVLAVGLGALQQSHTLHYPNISCKDEVPWSNGSSLRNFINSVEIKGLTGPIQFKNGQRTDFKLDLMKLKQHALAKVGEWTPGLGVNITDMSAFHDTAPYNITLVVVTIVEFPYVMMHLQKNYTGNARFYGFCVDLLEIIAKQVGFDYILDLVPDNKYGAQDPVTLEWNGIVEQLIKHKADLAVGSMTINYVRESVIDFTKPFMNLGISILYKVPSNQPAQFFSFMNPLALEVWIYWLVAYVVVSVTLYIVAKFSPYEWEDQTSGKLFSSPNIATNVPQLNRFTLSNSFWFTIGSLMQQGSDISPKAFSTKIIGCVWWSFTLIIVSSYTANLAAFLTVERMVSPIENAEDLAAQTNIAYGTLDSGTTMTFFRDSMIETYKKMWAYMQKKKPSVFVTSYDDGIERVLQGNFAFLMESTMLDYAVQRNCNLTQINGLLDSKGYGIATPMGSPWKDQISLSILDLQEKGEIQMLYNKWWKPPNDMCVPEESGAGAKTNTLDFKNICGVFAVLLVGLIISIMVSMFEFYHYRKKIKKIEQVVDVCFLSLI

>ApisIR68a_XP_001945663

SYKEIKWNSEFENLAVDITYKWKDTATCLNLILDNFHNGILDKAFYRAVSGIPLFKTLVDESEDLMSPNFQTWQILNHVRKEGCNMNIIFILNADQTMRLLKFSDKHRMLDSRTKFILLHDRRLFTKQHHTIWTKIINVVFIRKYRLKDMYELSTVPYPAPIKGALVTLRLDIWNKRNFQKKTDLYTDKVSDLQGNLLKVVTFNYIPSAIKNSVINENEENSGYKKGLEIEVLKSLGSAMNFIPVIYEPINWRTEQWGKKQINGTISGLLGEVWSARADLALGNLHYTPYHLNILDLSIPYNTECLTFLTFESKTDNSWKTLILPFRLNMWVGVLITLLIGGFLFYALATAHKHIEDSENSIKMIQCDTMKTKILEKKPELLTKNKGITKRNSILYTFGMLVAVSLPKVPSGWAIRILTGWWWMYCLLVVVAYKASMTAILANPDTRVTIDTLDALADSNINCGGWGEQSKEFFMTSLDKTGQRVGQKFQEVYEVDKAIDLVSKGQFAYYDNIHFLRYVKVMQNTKTYEKNSQFINDFTLHIMSKCIINMPISLGLQKNSPLKPAVDRFLRRVIEAGLVKKWLNDVMLDTVILEEPQQIEEVKALMDLKKLYGAFVVLVAGYILSILVLLIEIGYWYGVVKKDPLFDEYSLNCYYAQQ

>ApisIR40a

IYIFFLIRSTIYYVSFSGRDIFKNTVASAICNEYSIVVLTNXNANIIMLILINIIFISLYLSSSIILIDYNTYLIIQNLLVNVTIYINIYRLLGLHRDGDFLFFTQIRRSNLMSRNVVYVFLWLRSSVSRTFKADILEAMRVCVITSPRPGFYQIYYSQASARPGYGSSLKMVNWWSAMDGLVRFPLLPPPKQVYKNFEGRYFNVPVLHKPPWTFVEYLNDSFRVEGGRDDKLINLLADKLHFQFKYIDPPDRTQGSGLDRGSSMQGVLGLIWQREADWFVGDLSITYERNLVVDFSFLTLVDNEAFLTHAPGRLNEAFSLIRPFHWSVWPLLLITVIFAGPILYILVDTTDGHPQGKSMLYWKCVWWSVTVFLQQAAIIPSENNKIRFVAGLLMLSVTYVIGDMYSASLTSILARPPKEPPINTLKELSEAMRDSGLQLLVEVQSASQAMLENGTGVYEELSQLVTRQREYLIGSTEKGMQLVRDNKNYAVIGGRETFYYDIKRFGAQHFHLSEKLNTRYSAIAFQRACPYRDNFDDVLMRLFEGGILSKITEEEYQKLNDKLMGSEEFDSTSVVIEPVLEGSEPRQEDDDKQLTIAMSMKTLQGAFYVLAIGSILAGLLLLIEMRSHDKLEKDKVIKLVEAPFVYKRKVPNKFQNRLYDLK

>ApisIR100a

IQYTTPVMFDELCFVVPKAQQMSQWRAIFTAFDVRLWYCLAGSFVVALGAWTLIRRTTNTPYTLTLLSINLFRVFLMVPLNRVPSNISERLMVVSTVLFGFIMATSLQAVMVKYLSYPKYERDVATVQELYDTGMPVLSASNNLILLFESDERPLYVKMSDRFSIVNNKSLDILAAIGRKNDLVEKIASSYVENNEVLLHIVDECPRSFHIVYLVRRDVPFIHVVDRIITTFVESGIVNSWFMHAKPFRPISDYHHRKDPHKVFTFKNVVIAFLCLMIGLIASGIAFVAE

>ApisIR75j

IVLKYHYKSISLIIFDSEKYYAKYEYLLKFLYINKLPFQKCSSFIIKMLIVYVKFSESWLWNASYHWLMVMSKKSNNNIQSFLSFLSTKKINLSISSEILLAYPNEMDDIITNWHIFDIYRTAFEPRGKLMIDVVNKTFGKWGNRLWKFDKRSNLENLTLNVVTIVTKKNGTDIRPSSEELVKYLQTINLDRLNSVQKFSFAIMYPLTKILLNITLNIQTTNSWGYKINNKWDGIVGMLISGEVDFSISLNALRSERYDVVDYSAISTWKHLPCFIFRHPRSSTASTNIFLRPFETAVWFSIICILILSGFVYYTIIHYESNQMFSLKVLSDIVLLKIGTLCQQGTAMEPTKLSNRLIVLLMFVFSLVVYQFYSSSIVSGLLRPTVINIDSVQKLEESGLDVGVEDFNIVTKVIKVYGEINPYLKKIIDNKIKPKSEYLLAKDGVKKIKKGHYAFFTDPATSYWLINDIFTEKEKCDLTELPLHRPETTGYLVRKESPYRKLINYANCILRETGIMERELRIWHAEKPKCTTGKTTTEESLVSVEIKDVASLIAFLIIGFVISFTVLILEIIVHRHKTNTPIIIKK

>ApisIR75d2_XP_001951573

MFKNMSIHTVWTNTCWDTNTITCLEKSGVNLGISLELVQNVENKLSEGGRGIEFAKVKLSEASKRRLFNLQNDWVLFDQHGNPNKTDTLEIALHSFRTHMNQAYVMPDSRVYLLIEVDRNVWEIWDGFRLSVAEEVQVSRVGVMTPDRVDIKLSDRTNFKRVVLSASTVILEPNSFRGWNKPILTHNLDAFAHMHYAICTVLAEQLNFTLNLTLSADYGWNYGNGTYSGLIGQLQKEEIDFTAAGGLMRSDRMDVGDLTVGTFVARTVAIFKQPPLSSVNNVFTLPFVLNVWIVMFTVMSVFTVALIFLIWTSNWLHGVETRLSIALDMVTLVLAAICQQGLSVAAAQYSGPARLATFTLFLCAVFLFTSYAACIVVMLQLPSQTINTVRDLADKPITFSAQDTKHNYIYFNETVDENIKKIYREKMKPQGARAFTSPEIGVERVRNEFHSYLVEITSAYYIIAKTWQEHEKCSLSETELYKIPLLTLYVVKKSGYKDVFKQKMIQQHEIGLKDRTVKSIMPQKQKCVSANSGVARFDSVSMKEIYPALLFLGYGIFTSWLLLLFEHMYKTHVDKKNT

>ApisIR75d1_XP_001942610

MRNPMILLWLCLVASTFRTSVEYDSLINALTRVFVHKHVQTVTATTCWPTDANGRLLLALSSADISVNFRPSLIPVHKTWYKYGILVDVSCSVQIPTTLFRKASKHRLFNMQNDWILINSRRNESHVLNTSVIALETFEMHLSEAYVLPESNVFLFIDTPSGWEIWEGFKVGKMERIQVKRHGMVTSDGIYFEKNDSISFKSNLRGITLKATTVISEPSKFKGFYPFADTDLDTFAHMHYDLNMILQDQLNFKIDLGIVNSFGWDMGNASFSGLTGQLQREECDFSGIGVFIRNDRMTVIDYTVGTFYRQAAALFKQPPLSSVHNICILPFKFEVWMVTLFTFIGFTILIAFLSRMTRRLKKDEEETLNVLDSVTIVHGAICQQGYTMNLNAGSIRVAIFVLFLTAVFLFTSYSASIVALLQSPSNSIKTINDLVESSMTFSAQDNPYNDVYFGETDDPLLRKLYDKKMRPHGTQKFTLASAGIARIRTEFHGFMIDFVSAYKLISQLWLEEEKCGLSEIQLFKLPMLALAVVKRSGYKDILKQKLIHQQEVGLKNRIIRRWIPAKPMCDSSNRANQFVSVSIKEIYPMLQIYGFGLCISIVILFVEIVYNTYTDRSENLHSNTYFSIFRFMYNK

**SNMP sequences**

>DmelSNMP1

MQVPRVKLLMGSGAMFVFAIIYGWVIFPKILKFMISKQVTLKPGSDVRELWSNTPFPLHFYIYVFNVTNPDEVSEGAKPRLQEVGPFVFDEWKDKYDLEDDVVEDTVSFTMRNTFIFNPKESLPLTGEEEIILPHPIMLPGGISVQREKAAMMELVSKGLSIVFPDAKAFLKAKFMDLFFRGINVDCSSEEFSAKALCTVFYTGEIKQAKQVNQTHFLFSFMGQANHSDSGRFTVCRGVKNNKKLGKVVKFADEPEQDIWPDGECNTFVGTDSTVFAPGLKKEDGLWAFTPDLCRSLGAYYQHKSSYHGMPSMRYTLDLGDIRADEKLHCFCEDPEDLDTCPPPKGTMNLAACVGGPLMASMPHFYLGDPKLVADVDGLNPNEKDHAVYIDFELMSGTPFQAAKRLQFNLDMEPVEGIEPMKNLPKLILPMFWVEEGVQLNKTYTNLVKYTLFLGLKINSVLRWSLITFSLVGLMFSAYLFYHKSDSLDINSILKDNNKVDDVASTKEPLPSANPKQSSTVHPVQLPNTLIPGTNPATNPATHHKMEHRERY

>DmelSNMP2

MIHWSLIVSALGVCVAVLGGYCGWILFPNMVHKKVEQSVVIQDGSEQFKRFVNLPQPLNFKVYIFNVTNSDRIQQGAIPIVEEIGPYVYKQFRQKKVKHFSRDGSKISYVQNVHFDFDAAASAPYTQDDRIVALNMHMNAFLQVFEREITDIFQGFANRLNSRLNQTPGVRVLKRLMERIRGKRKSVLQISENDPGLALLLVHLNANLKAVFNDPRSMSVSTSVREYLFDGVRFCINPQGIAKAICNQIKESGSKTIREKSDGSLAFSFFGHKNGSGHEVYEVHTGKGDPMRVLEIQKLDDSHNLQVWLNASSEGETSVCNQINGTDASAYPPFRQRGDSMYIFSADICRSVQLFYQTDIQYQGIPGYRYSIGENFINDIGPEHDNECFCVDKLANVIKRKNGCLYAGALDLTTCLDAPVILTLPHMLGASNEYRKMIRGLKPDAKKHQTFVDVQSLTGTPLQGGKRVQFNMFLKSINRIGITENLPTVLMPAIWVEEGIQLNGEMVAFFKKKLISTLKTLNIVHWATLCGGIGVAVACLIYYIYQRGRVVEPPVK

>DpseSNMP1

MKLDRMKLLFVSAGTLVFAILFGWVMFPKILKFMISKQVTLKPGTDVRELWSNTPFPLHFYFYVFNVTNPEDVSQGGRPRLQEVGPFVFDEWKDKIDLVDDVVEDSVTFTMRNTFIFNAEASYPLTGEETITLPHPIMQPGGITVQRERAAMMELIAKAMSLVFPGAKAFLSAPFMDLFFRGIDVDCSPDDFAAKALCTVFYTGEVKQAKQVNQTHFLFSFMGQANHSDAGRFTVCRGVKNNKKLGKVIRFAEETEMDVWPGDECNQFEGTDSTVFPPGLKKEEGLWAFTPDLCRSLGATYVRKSSYHGMPSTRYTLDLGDMRSEEKLHCFCDDPEDLDTCPPRGTMNLAPCVGGPLLASMPHFYNGDPKLVAAVDGLHPNEKDHAVYIDFELMSGTPFQAAKRLQFNLDMEPVEGIEALKNLPKLILPLFWIEEGVHLNKTYTNMVKYTLFLGLKFNSGLRWTLITLSLVGLMSAAYLFYQNSDSLDITLPPKILKEVNKVADAAMNSKMFPEKAPTTPQTTIPGTNPPTNHGAQPPPAVASVPGIIPPLSLKMEQAQRY

>DpseSNMP2

MLHWSLIVSALGVCVAVLGGYCGWSLFPNMVHKKVEQSVILADGSEQYKRFVNLPQPLNFKVYIFNVTNPDMIQHGAIPIVEEIGPYVYKQYRHKKVKHFSRDGSKITYVQNVHFDFDADASAPYTQDDRIVALNMHMNAFLQVFEREITDIFQGFANRLNSRLNRTPGVRILKRLMERIRGKRKSVLQISENDPGLALLLVHLNANLKAVFNDPRSMFVHTSVREYLFDGVRFCINPQGIAKAICNQIKESGSKTIREQSDGSLAFSFFGHKNGSGHDVYEVHTGKGDPMKVLEIQKLDDSHNLQVWLNASTEGETSVCNQINGTDASSYPPFRQRGDSMYIFSADICRSVQLFYQADIQYEGIPGYRYSIGENFINDIGPEHDNECFCVDKLANVIKRKNGCLYAGALDLTTCLDAPVILTLPHMLGASNEYRKMIRGLNPDAKKHQTFVDVQSLTGTPLRGGKRVQFNMFLKSINRIGITENLTTVLMPAIWVEEGIQLNGEMVAFFKKKLINTLKALNIVHWAALCGGAGVALISLLYYLYQKGRGEEAPLK

>AaegSNMP1

MLIKNRKNLMLKPGTQMRGMFEKIPFPLDFKLYLFHVTNPDVVMKGGKPHVREIGPYFFEEWKEKYDTVDNEEDDTLTFTLKNTWIFRPDLTKPLTGDEMITIPHPLILGALLMVQRDREAMMPLVSKGMDIIMNPLTTGFLTTRVMDLLFDGILIDCSSQEFSAKALCSGLESEGAVMPFNETHFKFSMFGLKNGTDAGRWVVYRGVKNIMDLGRVVSFNDETEMDIYDGDECNRYIGTDSTIFPPFLTTKDKLWAWSPEICRSIGAEYGGKSKYAGLPMSFFKLDFGDARNEPEHHCFCRDPPDICPPKGTIDLAPCLGAPIIGSKPHFYDSDPKLLAAVDGLTPNEKDHDVYIHFQLLSGTPVSAAKRLMFSMEIEPIRDHAVLGNLPTVILPLFWAEEGASLNKTWTNQLKYTLFLGLRFNTAVKWLTIIIGTIGTIVGGFMHYKRTTKTVNVTPVQSVNGSSAKGNGAGMTVVGHQPDSKGGSVTTPVIPSAKDLLQNSRNLPTVIEGLDRPQKVTVTEMQERY

>AaegSNMP2

MMVMNTELRQDTPQFKRWEAVPQPLDFKVYIFNVTNPYEVQMGRRPRVVEVGPYVYFQYCHKDNIRFSRDRSKVHFSQQQMYVFDAESSYPLTENDQLTVLNMHMNSILQIIDTQAKETITNFRSDVNNTLEKIPVVRVIKRIIEKTTPIQSILQLAEDETYDSLRLINAELNRIFGRPDSMFLRTTPREFLFEGVPFCVNVIGIAKAICKEIEKRNTKTIRVQPDGSMKFSFFNHKNMTNDGTYTINTGIKEPALTQMIEYWNGRNTLDRWINQSAGSSSKCNKIVGTDGSGYPPFREGVERMTIFSSDICRTVDIKYVGPSSYEGIPALRFETDSHFLNEIGPEYGNDCYCVNRIPKAIVKNNGCLYKGALDLSTCFDAPVVLTHPHMMGAAQEYTSLIDGLYPDPEKHQIFVDVEPLTGTPLNGGKRVQFNMFLRRIDSIRLTDRLQTTLFPVLWIEEGIALNEDMVKLIDDSLMKVLTLLDIVQWVMIGSGLLLAIIMPIVYFIKRKPSSGSITPTLTTTTSTVSISDGGGLGGNPQK

>AgamSNMP1

MELKERNFKKIGLICVAVLLCGMVFSYGIFPSILRFMIKQNVLLKPGTQIRDMFEKIPFPLDFKLHIFNVTNPDEIMRGGKPRVNDIGPLYFEEWKEKYDTVDNVEEDTLTFTLRNTWIFRPDLSALTGEEIVTIPHPLIMGVLLMVQRDREAMMPLVKKGVNILFDPLESAFLKVRIMDLLFDGIYVDCSSQDFAAKALCSGMDSEGAVMPHNETHYKFSFFGMRNHTEAGRWVVYRGVKNIRDLGRVVSYNEETEMDIWDGDECNQYIGTDSTIFPPFLTAQDRLWAWSPEICRSLGAHYVHKSKYAGLPMSYFELDFGDLKNEPHNHCFCRDAPDDCPPKGTMDLSPCLGGPIIGSKPHFYGADPKLVEAVDGLAPNKAAHDVYIHFELASICWVSPVSAAKRLQFSMELGPIRDHELFGQLPDVILPMFWAEEGASLNKTWTNQLKYQLFLGLKFNATVKWLTIIIGTVGAVGSAYMYFRKETKTTDVAPVDVSTPDTNPSSAKDGVVNVSLGRNLPPVIDGLDKPPKLRATELQQERY

>AgamSNMP2

ATELRQGTDQYKRWEALPQPLDFKVYIFNVTNPYEVMQGRRPKVVEVGPYVYFQYRQKDNVRFSRDRSKVHFSQQQMYVFDAESSYPLTENDELTVLNMHMNSILQIIDNQAKETITNFRSDVNNTLEKIPVVRVIKRIIERTTPIQSILQIAEDETYDSLRLINVELNRIFGRPDTMFLRTTPKQFLFDGVPFCVNVIGIAKAICKEIEKRNTKTIRTMPDGSLRFSFFSHKNMTDDGMFTINTGIKDPSRTQMIELWNGRTTLDVWNNRSSGLSSSCNKIHGTDGSGYPPFRTGVERMTIFSTDICRTVDIKLTGSSSYEGIPALRYEIDNNFLHEIGPEYGNDCYCVNKIPKSIVKSNGCLYKGALDLSNCFDAPVVLTLPHMLGVAEEYTALIDGMDPEPERHQIFVDVEPYTGTPLNGGKRVQFNMFLRRIDAIKLTDRLQPTLFPVIWIDEGIALNEDMVKLIDDSLMKVLSLLDVVQWVLIGVGLLLAVLMPTVYFVKRCRGEGSRTVSPAVTATTSAASLSTVAGVTGDRSK

>CpipSNMP1a

MVVGLTVSMAILPELVNLMLRQNLRLKPGSDLRKMYEKVPFGLDFKVHIFNITNPQEIMQGGRPRVKDIGPFYFEEWKEKYDIEDNDGEDTMTFDMKNTWIFRPDLTAPLTGNEMITVPYLLVIGVLLAIQRDKEAMLPLISKGLDIIFEPLESAFVTVRVMDLLFDGIPVDCSSEEFAAKALCSGLDSEGAVAPLNDTHVKFSMFGLRNGTSIGRFKVYRGIKNVADLGRVITYNDETEMDFYDGDECNKYVGTDSTIFPPFLTKKDRLWAWSPEICQSLGAVYAGKSSYQGFPTSFFTIDFGDLRDDPVHQCYCRDPPDGCPPKGTIDLGPCVGAPILGSKPHFIGGDPKLLRDVDGLEPDPKEHDIFIHYDLQTGTPFSAAKRLQFNLELEPIRGHEVFGKLPKMVLPMFWAEEGASLNKTWTKQLKPLFMIRKFNATVKWLSIVLGTLGTIGAGFMHYKLHIKPVNVRPMEVQKTTVKEVEPSVETNGTGKEPPEKIEPRVVESAHRNLPPLFDGGLAGKQKPVPSDQRER

>CpipSNMP1b

MKLEELNFKKIAIICACTLVGGLTFCYGIFPPILKFMLKQNVLLKPGTQMRGMFEKMPFPLDFKIHLFNVSNPEEIMKGGKPKIKDVGPYYFEEWKEKFDTEDDLEEDTLSFTLRNTWKFRPDLSSPLTGDEMITLPNMLLMGVFLMVQREREAMMPLIRKGAKIIFDPLESAFMTVRVMDFLFDGLPVDCSSQDFASKALCSGMESEKVVLPLNDTHYQFSIFGGRNATDAGRWVVYRGVKNIKDLGRIVSFNGETEMDTYDGDECNQFVGTDSTIFPPFLTKEDRLWAWSPEICRSMGATYGGKSKYAGMPMSYFELDFGDLKNEPENHCFCRDPPEDCPPKGTMDLAPCLGAPLLGSKPHFIDADPKLLEEVQGLEPNREDHDMFINFELISGTPVSAAKRLQFNLEMEPVRDHEVLGNLPNVILPVFWVQEGVSLNKTWTNQLKYQLFLGLKFNATVKWLTIIIGTVGSIGAGIMHYKRSTKSVNVTPVEAVSNGSGRIISVSSAGKDREAINNSKNLPAVLDGLGERIPKMAPVEQRY

>CpipSNMP1c

NVLLKPGTQMRWMFEKIPFPLDFKIHLFNVTNPDEVMKGGKPKIRDVGPYYFEEWKEKFDTEDDLEEDTLSFTLRNTWIFRPDISAPLTGDEMITVPHLLVLGVFLSVQRDREEMMPLISSGMKIIFDPLESAFMTVRVMDLLFDGIPVDCGSEEFAAKAVCSGMESEGAVAPLNETHVKFSMFGMRNATDAGRWVVYRGVKNIRDLGRIVSYNGEPEMDIYDGDECNQYIGTDSTIFPPFLTKQDRLWAWAPEICRSLGAHYIGKSKYAGMPMSLFKLDFGDLKNEPENHCFCRDPPEDCPPKGTMDLSMCIGVPILGSKPHLLDADPKLLEGVDGLEPNEAEHDVFIHFELLSGTPVSGAKKLQLNLEVEPIRDHEVLGNLPTVVLPMIWVEEGVSLNKTWTNQLKYQLFLGLKFNATVKWLTIIVGTLGSIGAGFMHYKRTSQVTQVEQVAAGAKSEGGGRFISVSAATTEEGKNGGGGNLPAVLDGLDPSGISKRMSDPAQKERY

>CpipSNMP2

MKARNLNPPAPSQGGKLIKGKVLCSSPWVKAKAGCGAARLAKDNTELRQGTPQFKRFEALPQPLDFKVFIFNVTNPYEVQMGKRPRVVEVGPYIYFQYRQKDNIRFSRDRSKVHFSQQQLYVFDAESSYPLTENDPLMVLNMHMNSILQIIDNQAKETITNFRSDVNNTLEKIPIVRVIKRIIEKTTPIQSILQIAEDETYDSLRLINVELNRIFGRPDSMFLRTTPKEFLFDGVPFCVNVIGIAKAICKEIEKRNTKTIRVLPDGSMKFSFFNHKNMTEDGIYTINTGVKNALETQMIEFWNGKNMLDKWSNSSRGSSMTCNKIEGTDGSGYPPFREGVQRMTIFSSDICRTVDIKYVGSSSYEGIPAARYVTDDNFLNKIGPEHNNDCYCVNRIPKAIVKANGCLYEGALDLSTCFDAPVVLTLPHMMGAAEEYTSLIDGMHPDPEKHQIFVDVEPLTGTPLNGGKRVQFNMFLRRIDSIRLTDRLPTTLFPVLWIEEGIALNEDMVKLIDDSLMKILTILDIVQWTMIAIGLFLAISMPILYFTKRRPSSGTITPTLTTTTSAASIPERGGLGGNPDK

>BmorSNMP1

MQLAKPLKYAAISGIVAFVGLMFGWVIFPAILKSQLKKEMALSKKTDVRKMWEKIPFALDFKIYLFNYTNAEDVQKGAVPIVKEVGPFYFEEWKEKVEVEENEGNDTINYKKIDVFLFKPELSGPGLTGEEVIVMPNIFMMAMALTVYREKPAMLNVAAKAINGIFDSPSDVFMRVKALDILFRGIIINCDRTEFAPKAACTTIKKEAPNGIVFEPNNQLRFSLFGVRNNSVDPHVVTVKRGVQNVMDVGRVVAIDGKTKMNVWRDSCNEYQGTDGTVFPPFLTHKDRLQSFSGDLCRSFKPWFQKKTSYNGIKTNRYVANIGDFANDPELQCYCDSPDKCPPKGLMDLYKCIKAPMFVSMPHYLEGDPELLKNVKGLNPNAKEHGIEIDFEPISGTPMVAKQRIQFNIQLLKSEKMDLLKDLPGTIVPLFWIEEGLSLNKTFVKMLKSQLFIPKRVVSVVCWCMISFGSLGVIAAVIFHFKGDIMHLAVAGDNSVSKIKPENDENKEVGVMGQNQEPAKVM

>BmorSNMP2

MLAKYTKTIFSVSVAFLVVSIVLATWGFPKIIRKQIQKNVQISNTSKMYDKWVKLPMPLDFKIYVFNVTNRDAINQGEKPNLKEIGPYVYKQYREKIILGYGDNDTIKYNLKKTFVFDPVASGDLREDDELTVINFSYMAAIISVQEMMPAAVGMINRALEQFFTNLTDPFQTVKVKDLFFDGLFLNCEGDNTALGLICGKIRAEKPPTMRISKSANGFYFSMFSHMNRTVSGPYEMVRGTENLSDLGHVISYQGKRIMSAWDDQYCGQLNGTDSTIFPPLEDGNIPEKLYTFEPDICRSLFASLVGKDTLFNISTYYYEISDMTLGSKSANPDNKCFCKRNGSVKHDGCLLMGVLNLAPCQGAPAIASLPHFYLGSDELADFFGDGIKPDKEKHNTYVHLDPITGVVIKGVKRLQFNIELRNVPSVPQLKEVPSGLFPLLWIEEGAEIPEWLRKEIMDSHTMLWYVDAARWLVLAVAVVAVLVSATLVARSAALIPWPRNSNSISFILGNSVNTSKVHS

>AmelSNMP1

MKPKKLGIIGGSLLAFGILICAIAFPPFLRSQVKKQIALKDGSEMRELWSNFPVPLDFKIYLFNVTNPMEITAGEKPILEEVGPFFYDEYKQKVDLVDREEDDSLEYNLKATWFFNPSRSEGLTGEEELIVPHVLILSMIKLTLEQQPAAMGILNKAVDNIFKKPESVFVRAKAREILFDGLPVDCTGKDFASSAICSVLKEKDDALIADGPGRYLFSLFGPKNGTVLPERIRVLRGIKNYKDVGKVTEVNGKTKLDIWGEGDCNEFNGTDSTIFAPLLTEQDDIVSFAPDICRSMGARFDSYTKVKGINTYHYKADLGDMSSHPEEKCFCPSPDSCLTKNLMDLTKCVGAPLIASLPHLLGAEEKYLKMVDGLHPNEEEHGIAMDFEPMTATPLSAHKRLQFNLYLHKVAKFKLMKNFPECLFPIFWVEEGILLGDEFVKKLKTVFKTISIVGFMKWFTIVSGTCVSGAAAALFFKNKDKNKLDITKVTPQKGEEKKWPNQMTISTIQSAAVPPNLDAD

>AmelSNMP2

MPDSTLFDMKPSDGLFSIKNAILKNLPLIKGKDMYDEWILPVNLIFKCYFFNVTNPDEVMEGNNPNLVEYGPFTYREVFEKQIVDVDEELDEIIYDVKSTFTFDKYASLNISKRDTVTILNPAYIGTISMLTTLPPSYIEKFVKLTCNERKFPELSTICKTLKALRSPVLKEGEKEGVYYLSIFQRVNGTIRGRFSVNRGVNNISELGNIGSYNGRRVQTIWRTEKCNTVRGSDTITWAPLINPMPSVLSFIPDLCRSIEADYDKEVSIYGLIGSRFVMRERTWFLNQSQCYCLERNKVPNCLPQGLIDVSDCLKVPIIMSEPHFLHGDPQLLMYALGLNPSEDLHETFIVIEPYTGTPLSGQKKIQLNLKLERQPVDLLSNISEGYFPLLWCANGNTPDLSVIILTFQLLRLVKLIKFIDVIPLIIGIHMTIVAMLYCNCKKRKRQPTISIADSLLISSNSNNAHRST

>MsexSNMP1

MRLARGIKYAVIGAGVALFGVLFGWVMFPAILKSQLKKEMALSKKTDVRKMWEKIPFALDFKIYLFNYTNPEEVQKGAAPIVKEVGPYYFEEWKEKVEIEDHEEDDTITYRKMDTFYFRPELSGPGLTGEETIIMPHVFMMSMAITVYRDKPSMMNMLGKAINGIFDNPSDVFMRVNAMDILFRGVIINCDRTEFAPKAACTAIKKEGAKSLIIEPNNQLRFSLFGLKNHTVDSRVVTVKRGIKNVMDVGQVVAMDGAPQLEIWNDHCNEYQGTDGTIFPPFLTQKDRLQSYSADLCRSFKPWFQKTTYYRGIKTNHYIANMGDFANDPELNCFCETPEKCPPKGLMDLTKCVKAPMYASMPHFLDADPQMLENVKGLNPDMNEHGIQIDFEPISGTPMMAKQRVQFNMELLRVEKIEIMKELPGYIVPLLWIEGGLALNKTFVKMLKNQLFIPKRIVSVIRWWLLSFGMLAALGGVIFHFKDDIMRIAIKGDSSVTKVNPEDGEQKDVSVIGQSHEPPKINM

>MsexSNMP2

MLAKHSKLFFTGSVVFLIVAIVLASWGFPKIISTRIQKSIQLENSSMMYDKWVKLPIPLIFKVYFFNVTNAEGINEGERPILQEIGPYVYKQYRERTVLGYGPNDTIKYMLKKNFVFDPEASNGLTEDDDVTVINFPYMAALLTIQQMMPSAVAMVNRALEQFFSNLTDPFMRVKVKDLLFDGVFLNCDGDSPALSLVCAKLKADSPPTMRPAEDGVNGYYFSMFSHLNRTETGPYEMVRGTEDVFALGNIVSYKEKKSVSAWGDEYCNRINGSDASIFPPIDENNVPERLYTFEPEICRSLYASLAGKATLFNISTYYYEISSSALASKSANPDNKCYCKKDWSASHDGCLLMGVFNLMPCQGAPAIASLPHFYLASEELLEYFEDGVKPDKEKHNTYVYIDPVTGVVLKGVKRLQFNIELRNMPRVPQLQAVPTGLFPMLWIEEGAVMTPDLQQELRDAHALLSYAQLARWIILAAAIILAIIATITVARSTSLISWPRNSNSVNFIIGPMVNDKMR

**CYP sequences**

>ApisCYP_XM_001942470

MALQKIIRKIWTSIKVTCFIVLACVTALVKFVSKNTLGIYRKFRKPADAQRRIYKTVADIPGPRSFPIIGTRWIYWKFGSYKLNAVHLGFEAMFLCFGDIIREETLWNSPVISVINRDCIEKVLRQSGKYPIRPPNEVIANYRRSRPDRYTNTGVSNEQGVIWNSLRKRLTSKMTSPDVVQGVFPEIKSMVDDFIHLLCQARNKNNIVKGFEGLSNRMGLESSCMLILGRRNRFLDRVVNETAMRLTDAVTTQFRASQKTFYGHPFWKIIPTKLYKEFIASEETFYEIMSEIIDFALSDETQSGISENSVFGSILRAPNMDMKEKKAAIIEFIGAGIKTFGNTLVFVLYLIAKHPEVQEKLYNEISRLAPADTPITNEHLKQAKYLNACIMEAHRYSPTAPCIARVLESQIIYDGYCLPKGTTVLMQTGLACLDERNFKDATSYIPERWMNKETYDSLFLVAPFGCGKRICPGKKFVELALKIVLAKMVKQFHIGYEGQLETVFEFILTPVNANFILRDRIN

>ApisCYP_XM_001943115

MISFLIDCLVNNVTCLSLIVIFTGSFYYYSTSTYNKWRKLKIPYVPPVPLFGNTFRMLARLEHPIDTFDKIYNHFPDFKLFGFYQMREPMLLVRDPELINMILVKDFLYFTDHGVDIDPSMSTLAKSLFFANGQKWRTMRQKLSPGFTSGKLKGTYCQINECSDEMVSSIVEAIGKKTDRIELKTITGRFSTDVIATCAFGLKLDSIKNGDSEFRRYVKILFQTTTKQAIILILSLICPRVVKILRLQFFSLEATNFFSKVFADVIKYREDHNVSRNDITQTLIEARKELVLKEISTTEDKFTDDDIIGNAIFLFSAGSETISSLVCFCLYELALNKEIQDKLRAEIYSMKAKHNGKLNNDYLVDLRYTNMVLEETGRKYSIAFNITRVATKTYTLPDESFVIEKGQKLIIPMFNIHRDPKYYPDPLRFDPERFSMEQKSQRPNGTYIPFGDGPRLCIGKRFAEAEMKLVLSKVLSKFEVQPCEQTEIPLDIRSGSGLLSPKNGLVLKFKPIIEH

>ApisCYP_XM_001943388

MDSSTLILTCIAVALIAIAIIRRLVFVASFRSIAGPPALPIFGNALQLNGSPSGKSVRLYYKLVYIMVQQSSFNFKLLFQEILKSSVHIDKNLEYELLLPFIGTGLVTSSGSKWHTRRKLLTPTFHQNILEEFLPLIEKQIKTLVKVLRKEVNNVNGFDIKPYAKLAALDTIGNTAMGCELNSQENSQLEYVKALDELTAIMQKRFITPWLKPNLLFNLTSLSKRQKACIDVIHTFTRKVVKERKDNFKLFNGQTSDANKKKTHYEKKPNRALLDLLIEVSEDGKVLSDEDIQEEVDTFMFAGVDTTSVTLSWVMYVLGKHPHVQDKIVEELNEKIPNFGDEKLTVNILSSLDYLGRTIKEVLRLYPSVPFIGRQIYKPLTIGDHTILPGTSIFINVFALHRNEKHFENPEKFDPDRFLEENKKDRHRFAFVPFSAGSRNCIGQKFAMIVLKIAVATLIKTYRVKSIDPEEKLGLVGEIVLNALNGIHVTLEERA

>ApisCYP_XM_001943535

MFTANWWINVITPCTIIVTIAYYFCVSTFKKWEKLNVPYIKPIPLFGNFLNVAVGKDHPLEFYGKIYNEFAAHKYGGLYQMRTPYLMVRDPEIINDMLIKDFSSFPDRGIYSDFVANPLSNGLFFMENPQWKIIRNKLTPAFTSGKLKTMYDQIKECGDELMKTIDIELIKNGKEIEVRDIMGKYSTDVIGTCAFGLKLNAINDDESPFRKHGKSIFTPSLRSLFRELCLMVTPALLKVVRVKDFPTDATDFFHAVFKETITYRLENKIVRNDFVQCLIQARNDLVLNADLPNHEKFTESQIVANAFGMFAAGFETVSSTISYCLYELALNKSIQDRLRKEIQLKLSKNDGQINPEFLMDLNYLDMVIAETLRKYPPLVALFRKASQKYRLPNDSLIIEKGQKIIIPIYALHYDNKYFTDPENFIPERFSAEEKAKRPNGIYLPFGDGPRICIGKRFAEMEMKLAFVEMLTKFEVFPCDKTDIPLKYSNNVITLVPKHGIWLTFKRIN

>ApisCYP_XM_001943595

MFTDNWWIYVITPCTIIVTIVYYFCVSTFKKWENLNVPYIKPVPLFGNFLNVALGKEHHIDFYNKFYHKFAGHKYAGVFQMRLPILMIIDPEIINDVLIKDFSSFPNRGFSVDFKANPLSNNLFLMENPQWKIIRNKLTPAFTSGKLKVMYDQIKECGEELMKNIDIDLKKSGDEIEVRDIMGKYSTDVIGTCAFGLKLDAINDDESPFRKHGKSIFAPSLRQLFREMCMLISPVLVKVVRVKDFPKDATDFFHAAFKETMKYRHENKIVRNDLVHCLMQARNDLVLNTDLPKHEKFTESQIVANAFIMFAAGFETVSSAISYCLYELALNKSIQDRVREEIQLKLSKNDGQINHEFLMELHYLDMVLAETLRKYPPLVFLMRKALQTYRLPNDSLTIEKDQKVIIPVYAIHHDSKYYPEPENFIPERFSTEEKAKRPNGTYMPFGDGPRICIGKRFAEVEMKLAMVEMLTKFEVFPCEKTEVPLKYSHKTITLMPKHGIWLKFKKIN

>ApisCYP_XM_001943888

MIFSNVIGALTSDSNTQWMALLSLVVLGVYFLFSDRFSENRGRQISLLPSITRSQWTSLILSLKLASFGPRDILPYFDNVIKKYGSLIHLKIIARHYIIINDPDDIKVLLSSVQHITKGPDYEMLEPWLNKGLLTSTDQKWHSRRKLLTNTFHFKILETYVPSLNKHSRSLVKNLINASDNGKSIADIDSHVTLCALDIVCETIMGVNLRTQEGKSMNYVKAIKNVSQILIKRIFTFWYWNEIVFNLSSIGREFRKSLKLLHDFTENVIRERRKILENVEQKKVDENGKKRIYSFLDLLVGVSKENPGAMTDKDIREEVDTFLFEGHDTSSIAITMAIIHLGLDQNIQNLVRDELYEIFGDSDRDATMEDLKAMTNLERVIKETMRLYPSVTGITRTLKQPLHLDKYTIPSKSVMVVVPHLLHRDKNIYPNPEKFDPDRFLPEQCNGRHPYAYIPFSAGPRNCIGQKFAMYQMKTVLSTILRYTNVETLGTQKSIVISTQLILRADYLPSVKITPITNTTRHIIL

>ApisCYP_XM_001943946

MISYLTNLLFDYIFLSLIIVCTFLYYYTTSTYDTWRKLNVPFAKPVPFFGNIFKMFTGLERQVDAFGRIYQQFPDEKFCGFYQMSTPFLMLRDPELINTVIIKDFSYFTDHGIDMNPSVNVMARSLFFATGQKWKTMRQKLSPGFTSGKLKGTHEQIRECSDQLTNCIYEKSQKTDAIEVYELVGNTATDVIGTCAFGMKLDTINNDNSSFRQNVKKVFKPSGKVIFAQILGVLFPKIVKFLKLQTSPVDVDAVNFFHSVFGEVIEYRTKNDVVRNDLTQTLMKARQDLVVSSDYKGEEKYCELDIIANAMLLFTAGSETVTATASFCFYELALNKVIQDRLRDEIISSKIKHGGQLNNEFLEDLHYADMVLDETHRKYTIITALLRGATQNYNVPGESLLIEKGQKILIPIYSIHHDPKYYPNPETFDPERFTAEEKSKRPNGTFLPFGDGPRHCIGKRFAELELKLILSKILTKFEISPCEKTEIPLQMNKERGITSPKNGIWLNFRPIVE

>ApisCYP_XM_001944008

MIEVNFYSVVLVPLAGLISYAIWSRLRMPVEYRQISSHVPSVTKSFWSEMVLSWKLAMLQPKDILPFVTDLFKENGPVVHFNLSGRSYVLLNDPDDLKVLLSNTQYIKKGPEYEMLKPWLNEGLLLSSGQKWHNRRKLLTNTFHFKTLDMYNPSINKHSRILVDKLFEASANDDKEISIAEYVTLCSLDIICETIMGTEMNAQKGKSAEYVHSIKSACKSVIERIFKFWLWNDLVFRMSGSGQSFFKSIKILHEYTDNVIKSKRASLNNSGIEKIRSDSKFEKTKKKSFLDLLLNVLNDTPDQMSDRDIREEVDTFLFEGHDTSSIAMTMILVLLGMHPEIQDRARDELRSIFGYSTRDATMEDLNAMKYLEAVIKESLRMYPSVPAFTRELDKPLQLNKYIIPPMTTITVYPFILHRNEDIYPDAEEFIPERFLDEENKAKFIFGYLPFSAGARNCIGQKYAMNQMKIVVSTILRNAKFESLGRKEDIQISTQLIIRIESLPKMKFYKL

P450_XM_001944016

MFAQMRMAIHNAAHALPMTKSELYFYASIVIFVVLWCRMRWQYRQFYRLADKLKGPPSYPLKGSIFDLSTTPEKLMYNFKESAEKYNYEPVKLWVGPFFFVGVYKPEDVQIVLNSSKALEKGMIYHIIRHAVGEGVFTAPMGKWKKHRRVIASIFSSKFLDQLYPIFNENNKKLVENISKHVGETQPFDIWDYIISCNLNNVSQAAMGYNLNDQRTLSEFVLAMKKVSELSKCIVKPWLYIDQIFAVYTYLTGLNVYMSQLNRVSLQIIRDKKLEFKSIKLQQSTDKSHEVVPEKKRNSTKVFLDKLLKLNDEGADFTDEDLKDEVITMTVAGSDTSAISECFCILLLAMHQDIQDKVYDEIYSVLGDSDREVIPEDIFRFKYLEMVLKESLRLFPPGAIFSRKINENVKLTNFELPKGSNVFVSPYVTHRCPQLYPNPDTFNPENFSAENEANRHKFSFLAFSGGPRGCLGVKYAMISMKLMMVAVLRRYSVHTDCKLSEIEMQIDLLAKKANGYPITIRPRERTQDR

>ApisCYP_XM_001944057

MIEVNFYSVVLMPLAGLISYAIWSRLRMPVEYRQISSHVPSATKTFWSEMVLSWKLAMMQPKDILPFLTDLIRNNGPVVHFNLSGRSYVLLNDPDDLKILLSNTQNIKKGPEYEMLKPWLNEGLLLSSGQKWHNRRKLLTNTFHFKTLDMYNHSINKHSRILVDKLLDASANSNKEISIADYVTLCSLDIICETIMGTEMNAQEGKSVQYVHSIKCACKSVIERIFKFWLWNDLIYKISGSGQSFFKSIKALHEFTDNVIKSKRALLNNSGIEEMQSDSKFEKTKKKSFLDLLLNVLNDTPDQMNDRDIREEVDTFLFEGHDTSSISMTMTLVLLGMYPDIQDRARDELHSIFGDSDRNATMEDLNAMKYVEAVIKESLRLYPSVPGITRELQTPLQLKNYIIPPMTTIAVYPFILHRSENIYPNAEEFIPERFLDEENKAKFQFGYLPFSAGARNCIGQKYAMNQMKIVVSTILRNAKFESLGSKEDIQISTQLVLRIESLPKMKFFNL

>ApisCYP_XM_001944148

MANRYCSLVLVNSTKKRFMSTSNLKTVITESKKEIPIVKGLPLVGTMFSILAAGGGRKLHEYIDKRHQKYGSVFREKLGSVDAIWISNPLDMKLLFAQEGKFPKHILPEAWLLYNDTYGQKRGLYFMNGKEWWKYRQIFNKVMLKDLNVNFIKSYKVVINDLLNEWELSNGQVIPNLIADLYKISISFMVAHLVGRVYDDCKNDLSNDINCLAQCIQKVFQCTVKFTVIPAKTSKLLKLNIWNDFVIAVDNSIESANNLVSKLMSLNGDGLLNSVLNVHDIPIDMIKRLMIDFIIAAGDTTAYSTQWSLYTLGLHKSIQNNLRHSLLKTDFLECDYLNNILKEVLRMYPLAPFITRIPPSDIYLTDHKIPANSLVIMSMFTSSRNGKYFNSPNEFIPDRWNRLKNNKYNGVNEPFATLPYGFGARSCIGQKMAHVQMCLTLSECIKRYNIHTMQPVEIALDLITVPDDQINIKIHKL

>ApisCYP_XM_001944170

MVTNVQGVNPLFALSAFNLFFYLLTPAIVLWYIYFRMSRKQLYDLASKIPGSEGLPLLGNALDFMQDPHTIFEKIYERSFEFEKNSPIKMWIGPRLLVFLTDPRDVEVILSSNVYIDKSPEYRLFEPWLGNGLLISTGDKWRAHRKLIAPTFHLNVLKSFVTLFNVNSRDTVSKLRKMGSSTFDIHDFMSECTVEILLETAMGVSKKTQKKSGFEYAAAVMKMCDILHMRHTNLWLKPDFIFNFTKYAKEQVGLLDLIHGLTNNVLAKKKEEFLKKKSLMKEVSDIPAASEEIVETSSTLEVEEVPYGNSFGQSAGLKDDLDVEDDGIGEKKRVAFLDLLIECSENGVVLSDEEVREQVDTIMFEGHDTTAAGSSFFLCLMGAHQDVQQKVVDELYSIFGDSDRPVTFQDTLQMKYMERCIMETLRMYPPVPIISRQIKEKVKLASRDITLPVGATIVIATFKIHRNEDVFPNPEVFNPDNFLPEKSASRHYYAYVPFSAGPRSCVGRKYAMLKLKIILSTILRNFKINSNLTEKDWKLQADIILKRTDGFKLSLEPRKSLAKTAA

>ApisCYP_XM_001944396

MWCYFKWHNRPFEKLAARMPGLPAYPFIGSLYTCIGVTSEQLRSRILDLVKDYNLGPIKCWMGPYFGVFIVRPEDIQIVLNSSNALQKGFVYNFFKVILGEGLFTAPIDKWRIHRRMISPFFNGKLLEQFFPVFIEKNRILIRNVAKQLNETQVFDLWDYIAPFAFDTICQNTLGYNIDTQTNKNECEFAKAIVKTLDLEGMRIYKPWLYPEFVFSMYLKLTGQQRVFETVRKFPLQVIKEKKAEFDQRKKLIDAKIDVTNSNEHQSKLFLDTLFELNNGGGNFSDSDIRDEVITMLAAGSETNAITICFCLLMLAIHQDIQDKVYDEIYDILDDSDHMISIEDTTRLVYLEQVLNETLRLFPAGPMQLKEIQEDLKISSSDYVLPKGTMCVISPLVTHISPDLYSNPRDFNPENFSPENIAKRHRYSFIPFSGGPRGCIGSKYVMMIMKVTVSTFLRHFSVHTNIKLTDIKLKLDVLMRSVDGYPVTIQPRHKRPTYKRNKKPLR

>ApisCYP_XM_001944452

MIEIIVVIFVMMWCYIKWHNRPFEKLAARMPGFPAYPFIGTGFQFIGLTPEQIMNRILDYEKDYNLEPFKIWIGPYFGVFIVKPEDLQIVLNSSKALQKGCVYDFFKHVTGEGLFTAPVDKWRIHRRMISPLFNGKLLEQFFPVFIEKNRILIRNVANQLNETQVFDLWDYIAPFALDTICQNTLGYNLDTQTNKNGCEFAEAIVTTTDLEGMRIYKPWLYPEIVFSMYLKLTGQQRVFETVRKFPLQVIKEKKAEFDQRKKLIDAKIDVTNNNEHQSKLFLDTLFELNNDGGNFSDSDIRDEVVTMLTGGSETSAITVCFCLLMLAIHQDIQDKVYDEIYDIFDESDHMISIEDTTRLVYLEQVLKETLRLFSVGPLLLREIQEDLKIFSSDYVLPKGTTCVLAPIGTHLSPNLYSNPRDFNPENFSPENIAKRHRYSFIPFSGGPRGCIGSKYAMMSMKVTVSTFLRNFRVYTDIKLTDIKLKLGLLMRSVDGYPVTIRLRDKRPTYKRNKKPPR

>ApisCYP_XM_001944495

MRIFKPWLHVEIVFSMYLNLTGQQRVFETVRKFPLQVIKEKKAEFDQRKKLNDAKMDVTNSNEHQSKLFLDTLFELNNGGGNFSDSDIRDEVITMLIAGSETSAITVRFCLLMLAIHQDIQDKVYDEIYDIFDESDHMISIEDTTRLVYLEQVLKETLRLFSVGPLLLREIQEDLNLVSSDYVLPKGTMCIISSIATHHSPDLYPNPWSFNPENFSPENVVKRHKYSFIPFSSGPRGCIGSKYAMMSMKVTVSTFLRHFSVHTDIKLTDIKLKLGLLMKSVNGYPVTIRPRDKRPTYKRNLKPLR

>ApisCYP_XM_001944564

MFPAVVIIVACCTTVILFLYKYTTYTYKYWKSKSVTFATPVPLFGNIKDHVTLKMTQGECLKNIYNDFPREKFVGMYQLQTPTLLLRDPETIRLFLVKSFAHFTDRGFSYDGHREPLTKHLVNLEGDTWKILRQKLTPTFSSGKIKSMLGLLQGCGVQLIEYMDATIESGKTEFEIRDLTAKFTTDVIGTCAFGLECNSLKDSQSEFRRMGCAVLNSSASLALAKMVRVFFPKLFKALKLRTFPAEVQQFFMGIVKQTIDFRNTNRVRRNDFIQLLLEIKNQNHNQENAIKSIELTEELIAAQVFVFFLAGFETSSTTLSFCLHEMAVNQDIQNRVYDEINETANMYGLPFSYEAISSMNYLEQCLKETMRKYPPVQALARVCTKQFRVPGTDLDLDVGTAVLIPVYAIHHDPQYYPEPDTFNPDRFAKDGDGGGGDNGRPSGVFLPFGDGPRICIGMRFAMLEMKLALAQFLHRYLVTLSDKSCTRIEFEPASFLSCPKGGIWLNVNKRKA

>ApisCYP_XM_001944991

MIEIIAYIIGIVLVMVWCYFKWQNRRFEKLAAIMPGPPAYPIIGIGYTFFGSSEHVMSKIIDLVKEYNLSPIKLWLGPYFAVSISKPEDLQIILNNSKALQKDRMYDFFKYAVGEGLFTAPVDKWKRHRRMITPAFNAKLFEQFFPVFNEKNKILIKNVTKELNKTQMFDLWHYVAPAALDTICQTTMGYNLDTQSNNKECEFGEAIGIGGSSDANLQTMDISRNGVFNVFKTHWTPKGFRNSEKFPLQVIKEKKDEFDQRKKAINAKVDLANNKDENQSKLFLDILFELNNTGGNFSDSDIRDEVVTMMTGGSETSAITICFCLLMLAIHQDIQDKVYDEIYDIFGGSEETITIEDTTKLVYLEQVLKETLRLYPVRPVLLRELQDDVKIFSNDYVLPKGTTCVLCPITTHHCPVIYPNPWSFNPENFTPENVAKRHRYSFIPFSGGPRGCIGSKYAMLSMKVTVSTFLRHFS

>ApisCYP_XM_001945065

MSASQLLVDLAAGWWTVAVLALLAATVYHFCTSTFGYWRDRGVPYVRPTVPLFGNIGGLALGVEHQARMFGRIYDGFRGQRYGGFFQMRTPHLMVCDPALVNRVLIGDFAHFTDHGMYTAGPDENPLANGLFNMNGAQWKIMRQKLSPVFTAGKLRHMRGQVTECSEQLMRNVAADVPTGGGQMEIRDVLGKYSTDVIGTCAFGLHLNAINDERSSFRKHGKAVFAPSFRVLLKELAWMVTPALRRALRIGDMPPDAAQFFTAAFTDTMKYREEHGIVRDDFMQSLIQARTDLVVNKTEPSVEFLETDIVANAFILFAAGFETVSTAMSFCLYELALKKPIQDKVREEMNTTKKKHNAEIDNDFLKDLHYLEMVLAETLRKYPPLLTLFREATQDYQVPDDTFVIEKGTKVLIPAYAIHHDYRYYPDPETFDPERFSPEEKAKRPNGTYMPFGDGPRLCIGKRFAEMEMKLALTELLTTYEVEPCEKTDIPMRFSKRSLIITPENGIWLKFKPIHTSK

>ApisCYP_XM_001945326

MKGPKGYPFIGSSFDFIGTPEQVMEKVLKIDDKYSPGPIKIWVGPYFGVIVIKPEDVQAVLNNSKALQKDRVYDFIKNIFGEGLLTAPVHKWRKHRRLITPSFNASLLNQFFPVFNEKNKILIRNLKKELGKTTPFDLWDYIAPTTLNLICQTAMGYNLDTQSEYGTEFENAMIKASELDSLRMKTPWLYLSFMFKLYLKLKGHSDVFNTLYKLPIKMIQEKKEAFAQRKILNKPSAVDVTDNEREKLKVFLDTLFELNEAGANFSDDDIKDEVVTMMIGGSETSAITICFSLLMLAIHPDIQDKVYDEIYEVFHDDNETITIEDTNKLVYLEQVLKETLRLFPVLPLVFRKLEDDIKIDDLVLPKGTTCIISILGTHHFSESYPNPWTFNPENFNPENITNRHKYSFIAFSGGPRGCIGSKYAMMSMKVAMSTFLRNYSVHTHYTFDDIKLKIDLLLRSANGYPVTIQLRDRRPTYIRNKKL

>ApisCYP_XM_001945510

MLETNVYNFVLIPLAILISYAIWSRLRKPLEYRQISSHVPSVTKNLWSELLFSCSIAMKHPRDLLPFFMEIFLNNGPVVHCNITGRSYVLLNDPDDIKILLSSTQYINKGPEYKMLKPWLNDGLLLSSGLKWQNRRKLLTNTFHFKTLDMYNPAVNKHAKVFTKKLLEACEDDKEISVMEYVTLCSLDIICETIMGTEMNAQKGKSIQYVYSIKSACRSVIDRVFKFWLWNDLIYRISESGRSFFKSIRVLHDFTDSVIKRKQSLLKTSGNTIVQPESKPAEKRKTKSFLDLLLDVLKDNPDQMTIKDIREEVDTFLFEGHDTSSISMTMTLLLLGMHQDIQDRAREELHSIFGDSDRDATMEDLNAMRYLDAVIKESLRLYPSVPSFTRELETTLQLENYKIPPMTTMVIFPYILHRNENIFPKPEDFIPERFLDEDNKSKFLFGYIPFSAGARNCIGQKYAMNQMKTVVSTVLRNAKIVSSGCKEDIKISMQLLIRIESLPKVIFRPL

>ApisCYP_XM_001945726

MDTAKGVVAAAADNVTVVLLLLLSVVLLILAVKSASGRGPWTSRRRPGKSTAAVALTAVPDGPTAYPVIGALHAMDGHRDKPFHRFTELSHKYGPVFSMTMGSMPCVIVNDFDSIKEVLITNGSKFGGRPDFSRYNVLFAGDRNNSLALCDWSWLQETRRKIARKYCSPKVCSSNYGLLDSISSDELDVFLESLAAVTIRGFECEVQLKKQLLMACANMFIRFMCSTQFEYGDPKFQNMVRTFDEIFWDINQGYAVDFLPWLKPFYAGHMRKLSKWSTQIRRFIMDTVVSKRISYAKAAALGSPGDYNDYAADDVDEQEPIDFTDALLMSLRKEPGLKMNHVLFELEDFIGGHSAVGNMIMLALSMVATRPHVAQAIRDEAEQVTGGQRLVRLYDKPDMPYTEATLFETLRFISSPIVPHVATEDTTIKGFKISKGTCIIINNYEINTSPAYWDNPEVFDPNRFVHRESGTKPCIRKPEYFLPFSTGKRTCIGQQLVSGFGFVLLAGILQRYEVKATAQLAIPEARLALPPDTYPLILKPLDGSR

>ApisCYP_XM_001945798

MMYFLTDWLLDNFTYLSLIAVFTGFYYYSTSTYGKWQKLNIPYIPPVPLFGNAFRMVTKLECPMDMYDRLYKQFPDVKLLGFYQMTEPMLLIRDPELINAILIKDFPYFTDHGFVMDPSTTVMAKSLFFSNGQRWRKMRQKLSPGFTSGKLRDTYLAINECSNQMVSSIVEKLGKTDRLAIRSIISGFSNDVIGMCAFGIQLDSMNNEDSDFRRYSERIFEKTTKQIIVQAVTTIFPFVINLFKIQMFSAEATNFFRKVFADVINYREKNNIVRNDLTQTLLQARKELVLKENSTAEDQFTDDDIIGNAIVLFAAGAETISSIVSFCLYELALNKEIQDKMRAEICSMKAKHDGQFNNDFLMDLRYTNMVLEETGRKYSIASILMREATKTYTLPDESFVIEKGQKLIIPMFSIHRDPKYYPDPLIFDPERFSKEQKSQRPNGIYMPFGDGPRMCMGKRFAELEMKLVLSNVLSKFEVLPCEETEIPLEITDETGVIAPKRDLVLKFRPIIED

>ApisCYP_XM_001945899

MLNALEDWRAECWTIPIYHPRAKPVECHNQDLKKGLRAQLVVGHHKSWDTKLLSILFSIRNKCNEQTSYTHTVFVLGKESKRPRDWALPKSAPVDFEKSQEERVVRKQRIRERDSGHTEAEHQGPSRIPFIGNYLEIRKLRNELGFYHLVWHQLAKCYGQVFSVKLGRIEAVVVSGYDAVRQVLCKDDFDGRPDGFFFRFRAFYKRLGIVFVDGPTWNDQKKFCMQHLRKMGFGGDLMEKIIIEEVHDLMVDITIKSENGKPIKVHGLFDISILNGLWAMLAGQRFALNDSRLARLMELVHVSFRMLDMSGGILNQMPFIRFLAPNSSGYEHIKQILNEFYTFLKESVEEHKCGENYQEDFISAFLMEIEKNKESPESFSEEQLLVILLDLFLAGSETTSSMLSFAVLLLLKHQDIQDKVHAELNAVVGDREIQLADKKKLNYLEAVLMEVQRHSNVAPLAIAHRTIRKTSLQEYIIPKDTLVLASIWSVHMDEHHWGDPEVFRPERFLDSTGNIIKDSWLMPFGIGRRRCLGEILAKANVFMFIANLIQNFEIRIPNGVQLPDRPQDGVTISPSPFSAIFIPRR

>ApisCYP_XM_001946260

MKKIEKKHYNKKEQLIEDYKTIFSKDKYDVGTLKDYEARKDLLVDKFCSKRPYRCTIEDRKEIEQQVAKLLEENLIEESYSPFAAPVTLALKKAFWSKPLRIKDRNKTGFITQEGHYQWTCLPFGLKTASAIFQRILSSILRNNNLKNFTENYIDDILIFLETFEDHIIHIKQVLEAIIKEVRPIKDNLVSIHNFPSPKTQKNIRQFLGKINSYHEYIPEISTIIESLHRLLRKDVKFNWSVDCEKSFTEIKKLLCSQPVLEIFDKDLPIKIFTDASIEEPLCRTKLVLVLSNCYDAHPDNHNVVACIIYLVFCFRRKIKIQTIKTVAMDTTNGIVAGADTVTVALSLLLPVVLLMLAVAWACGPLAAHRRPGTSTAAVLDGPKSFPIIGSLHAMDGHQDSPFRRFTELSHQYGPVFAMTMGSMPCVVVNDYDSIKEVLITNGSKFGGRPDFTRYNALFAGDRNNSLALCDWSSLQETRRKIARTYCSPKVYSSNYCLLDSISSNELDVFLDSLATVSVRGSECEVQLKQLLLMASANMFIRFMCSTQFEYGDPEFQNMVRTYDEIFWDINHGYAVDFLPWLKPFYAGHMRKLSKWSTQIRQFIMDMVVRENNDYAADDDDVDEQEPTDFTDALLMSLRKEPGLKMNHVLFELEDFIGGHSAVGNMVMLALSMVATRPHVAQAIRDEAEQVTGGQRLACLYDKPDMPYTEATLLETLRFISSPIVPHVATEDTTIKGFKISKDTCIIINNYEINTSPAYWDNPEVFDPNRFVHRKFGAKPCIRKPEYFLPFSTGKRTCIGQQLVSGFGFVLLAGVLQRYEVKATAELAIPEARMALPPDTYPLILKPLDGSR

>ApisCYP_XM_001946349

MQHPIDFYRKIYYELAGYKYGGLFQMRTPYLMIRDPEIINNVLIKDFSNFPNRGIYSDFSANPLSNQLFFMENPQWKIIRKILSPAFTSGKLKLMYDQIKECGDELMKNIHKNLTKTDNKMEVRDILGKYSTDVIGTCIFGLKLNAVSDDNSTFRKYGKSLFLPSLRTHLRELSLMITPALLNILRFKDFPADATEFFHSAFHETITYREKNNIVRNDFVQTLIQARNDLVLNKNIPQRERFLESQIVANAFVMFAAGFETVSTAISFCLYELSLKKHIQDKVREEINLKLSKNNGLINNDLLIDLNYLDMVLAETLRKYPPTFALFRKASQTYHVPNDSLTIEKDQKVIIPIYSLHYDPKYFADPEVFDPERFSPEEKSKRISGTYLPFGDGPRICIGKRFAELEMKLALVEILTKFETEPCERTEVPIRFSKKALITMPENGIWLTFKKITNQ

>ApisCYP_XM_001946393

MFEFVYELFDLKMLLVTAFLGAIYVYSTWTHSHWSKLGISSPSAPVPLFGHAMPSMLGQMHFMDVLHNLYKELGDQRFGGIYTMRTPQLLVKDPELIGHILIKDFNNFTDRGLYAGTHTNPLNNNIFFTRGERWKTMRQKLSPTFTANKLKYMNEQVKECSDGLLSTIGKNLDDDAGRIEIREMMAKYSTDVIGSCAFGLKLDAINDPDSEFRKHGKTVFQPSLRSKIRVAVIFMQPSLLSIFRVHHYSHRTIRFFHDAFQQTIEYREKHNEDRKDFVQHLMKAREDLVLNPNLKPEEKFTEMDIVANAYILFIAGFETVSTSMSFCMYELALRKDVQDKVRKEILEVKSKYNGQMNSECLNELHYMGMVIKETLRKYPPLVTLNRVVTKPYVIPGTQIKLKIGTKIVVPVHAIHYDPKYYSDPEAFEPDRFSDENIHNIQPNTYMPFGDGPRFCIGKRFAEFEMKMALSEVLTNYEVMACDKTQIPIKYVIGSFVNIPESVWLKFRKVNT

>ApisCYP_XM_001946709

MSVLARRLRNLRITVDHANKSTEVFTSVSQGDVDFVKDYSELPGPKSLPLLGNNWRFMSYIGDYKVTEIDKLSLRLWKEYGDIVKIEKLLGRPDMVFLYDADEIEKVFRNEELMPHRPSMPSLNYYKHVLRKDFFGDLAGVIAVHGEKWYEFRTKVQQPMLQPRTAKFYIGTIEDTATAFVNRIKKIKNKDQEVPDDFLNEIHKWSLESIAKVALDQKLGCLEDEHAVDSDTQNLIDAINTFFANVPELELKIPFWKLFSTPTWRKYINALDTITNVTSKHINRSMDRLLSQKSFCPDSQSSLLQRVLSLDPSNPKLAQILSLDMFIVGIDTTSAALASILYQLSRHPDKQKKLREEIRTVLPNADSKLTSSKLEQLQYLKACIKETLRMYPVVIGNGRCMTKETIISGYKIPKGVQVVFQHYAISNSSKYFSQPDQFLPERWLKGSGYKHHPFASLPFGYGKRMCLGRRFADLELQTVVSKIFQNFEVKYEYGDLEYTVHPIYMPDGPLKFKMIED

>ApisCYP_XM_001947219

MYTLIQKVIDLNELDMWYLTVITPIAILVVIIMILRLEGRRKRVLANKIPGPDGSIFIGMLPLFLQGPEQLILKGLKVYQKYEKSLFKVWVLNNLYIVLTRPEDIEMVLTNPKLQKKSKEYLVLQESIMGQGIFSIDDIKKWKSNRKMVSGGFNFTIIKSFIPIFYEESNVLNDILKQKCDLKSNECDISVPVSMATMEMIGKTALGVKFNAQNGGRHRFVENLQTAMHAWEYRISHPWYLSKTLFQLSSVKKKHDQSQKIINEFTDEIINKKLDELNQNANNKNKVETDDEDVCRKTKTVIEILLGNYHEMSHEQIRDELVTIMIGKYKNIYNVQSNK

>ApisCYP_XM_001947733

MESGEDIVRSEDGHCYETFETDIKDYITTYGSPRCVIRITVSNSPAKFGRLVIGKKEFPNADFGLPPNRTKARENVFERIVIKEKKAEFDQRLKATNDKVDVTNNDDEKYSKLFLDILFELNNNGGNFSDSDIRDEVVTMMTGGSETSAITICFCLLMLAIDQDIQDKVYDEVYDIFGESDHIITIEDTTRLVYLEQVLKETLRLYPVGPVLLREIREDLKIFSNDYVLPKGTTCVISPIATHHSPDLYPNPWSINPENFSPENVAKRHKYSFIPFSGGPRGCIGKEIFYEIIDPNMP

>ApisCYP_XM_001947787

MFQDRKPTREDRQQTIPVPEFVLFDLQVHERSWRKIGINQQTPVAKNKFKTFIDTLLEASENDPDFTNADIRDEVITMMFAGSDTNATTECFCLLLLAIHQDIQDEVYDEIYNVVRDSDRELTPEDTANFSYLEQVIKETLRMYPTISVFTRQLVEDVKVTNYVLPRGASVTISPIVTHHCPHLYPNPEAFNPDNFSIENVAKRHKYSYIAFSGGPRGCIGMKYAMISMKLMITEILRNFSVHTDIKLSDVRIKMNDAFTRKVGGYPITIRPRDRRPSYVRRNTRVA

>ApisCYP_XM_001947839

MFWIIGVILFGVLCAGYLWRSNRNLPPGPWGVPIFGYLPWLNPTEPYKTLTALASKYGPIYSIQMGKHFAVVMSDPTLVRMALARNELADRTNFEVVNEIMQEHGLIFTHGPLWKEQRKFVCNWLKVIGVTKFGDKKNNLQLLIADAVSTTISKLRQSNNRPIDTGTFFLVHIGDFINLIVLGKAWPEDDPNWIYLRNLAEDGSKKFAIATPLSVLPILKIIPKYRNTVFEVIEGVKNTHLIYKTLMEKRGNEIHESDDLMAMFMKEMTKRKNDKDSHYFTEKQCCFLLSDLFGAGVETTVNTLRWFLLYMALNQEIQNDLQKLLDSACTDGGLIGLEQIESIPLLKACVSETMRLRPVAPSGIPRSVNTEITISGYRIPKGTMVLPLQWAMHHDEKYWTDPETFRPKRFLDDEGNMINHKAFMPFQAGKRACVGDTLSYWILYLFGANIIHNFNVSAEQGLSEKEINTIMDGEFGITLSPATHKVVFKSRI

>ApisCYP_XM_001947885

MIDVISCSIIGLLSSVYILYATVFLSIAYYLCTSTHDKWRKLNVPYTKPLPLFGNSMNLVLAREHPMDFFTGLYNRFPDEKLCGFYQMTTPFLMIRDPKLINNIMVRDFSYFTDHGFDTDPSVNILANSLFMLNGDRWRTMRQKLSPGFTSGKLKDTHDQIKECTDQLINIVDDNLKVSDHFEIRELVGNFSTDVIGMSAFGLKLDTIRNGNLDFRKFGKKIFQSDFKQLFVQAMMLFCPKLVTILKLKQFPDDAADFYGSMFRDVLEYRDRNNVIRNDVTQTLIQAKKDLVTNNDGDDSTSKNKWTEMDIVGNAILMFVAGAETVSITICFCLYQLALNKDIQDKLREEIVTTNAKHGGQLNNDFLTNLHYMNMVLEEVSRMYSITMILFRQATKNYEVPGQSLVIEKGQKIIIPAYCIHNDPKYYPNPGTFDPERFSTEEKAKRLNGTYIPFGDGPRLCIGKRFAELEMKLVLSKILLKYEVLPCEKTEVPINIRGAGSIVNPKNGVWLSFKPIVAN

>ApisCYP_XM_001947888

MTAETMSDGDGYSRELWLNAVAAALGLTYSAYRQLRAARTLPPGPWGVPFLGYAPFLSNHCTYLKYNELARRYGPICSFTQRGNTVILLSDHKLIKTAFDMKQITGRPNDGYMDIIGGYGAVNSTGKLWESQRKFLHLVLRHMGMTFTGHNRLNMENRIMIEVSTLTETFHKTCGKPIDLNAGSLCLAITNVISSLTMSVRFEPNDPRFERYMHMVDEGFKLFGMLRPVSLFLPRRHITDERNIQEKIKNNHQEIAKYFQSIIEEHRSTFDPNSIRDLVDAYLLEIKRSQEAGTMDQLFQGLDPNRQVQQILGDLFSAGMETIKNTILWAMVYMLHYPDVMTKVQDEIDSVVGQYKSPVLDDYPNLPYTQATLYEVLRKSSITPLGTTHATTSDVTLNGYHIPTGAQIIPLQHFVHNDPNLWDEPEAFKPERFINAEGKVKKPDCFLPFGVGRRKCLGETLAQMELYLFFSTLLHEFDVCLPDGDELPSMDGQVGITLTPQSFKVVMKARNK

>ApisCYP_XM_001948106

KMVSGGFNFTITKSFIPIFYEESNVLDDILKQKCDLKSNECDISVPVSMATMEIIGRTALGVKFNAQNGGRHRFVENLQTVMHAWEYRVTHPWYLSKTLFQLSSVKKKHDQSQKIINEFTDEIIKKKLDELNQNANNKNKVETDDEDVCRKTKTVIDILLENYHEMSHEQIRDELGTIMIGGQETTAMANACAIFMLAHHPDVQNKVFEELQSIFSTGDGDHSRPLTYEDLQQMEYLERVIKETLRIFPPLPVFCRSLDEEMKIGEHMCPAGSTLLVSPLFIHSSGQYYTDPEKFNPDNFLPDTCHSRHPYSFIPFSAGYRNCIGIKYSMLQMKTVISTLVRKNTFSPSERCPTPKHLRVMFLATLKFVDGCYVKIVPRTS

>ApisCYP_XM_001948264

MVFLNGSDWWRLRKAFQKHLSKVQCIKRYVDSTNTVVGEFIDRRIKRAELRDDFGPELSRLFLELTYYVAFDERLQRFKDEEWDSDSECSKLIKAAHDINSAIMKTDNGPQLWRKFDTPMYKSIQKGHEQIEKIALRVVNEKLISIKTTDSKTSLLGEYLSSDDTDFKDVIGMTVDTLLAGIDTATYSCCFGLYHLSSNPDVREKMFDESRALLPDNHTPVTDRVLERAVYAKAVVKEMFRMNPISVGVGRILPEECVFSGYRVPAGTVVVTQNQVSCRLEEYFRRPNEFLPERWIKGSAEYEPVSPYLVLPFGHGPRTCIARRLSEQFLQVVLIKIVRNFEMTWTGPKLDSESLLINKPDGPISIIFKTRD

>ApisCYP_XM_001948386

MISFMTDWLHDNVTCLSLIAVLASFYYYSTSTYGKWRILNIPYVPPVPLFGNTTRMMLRLEHPIDMFERFYNSFPDVKLFGFYQMRDPVLLVRDPELINAILVKDFSYFTDHGIDLDSSTSVLANSLFFANGQKWRTMRQKLSPGFTSGKLKDTHGQINECSDEMVSGIVESIKKKTDQIDVKTITGGFSTDVIGTCAFGMKLDTIKNDDSDFRRYVKIMFQSTPKQMIVQVLLMICPWVIKVLKINMFSVEATNFFHNVFTDVFKYREEHNVIRNDLTQTLMQARKELVLKENSSIEDKFTDADIIGNAILMFTAGSETISSMLSFCLYELALNIEIQDRLRSEICSMKAKHDGHLNNDYLMDLYYTNMVLEETARKYSIAFNLMRVATKTYTLPDESFVIEKGQKLIIPMFSIHRDPKYYPDPLRFDPERFSTEQKSQRPNGIYMPFGDGPRLCIGKRFAESEMKLVLSNVLSKFEVLPCEKTEIPVNIRSMSGFITPKNGIVLKFRPIVEH

>ApisCYP_XM_001948408

MTMFTASWWINVITPCTIIVTITYYFCVSTFKKWEKLNVPYIKPIPLFGNFLNVALGKNHPLEFYNKIYHEFAGQKYAGVFQMRTPYLMVRDPEIINDVMIKNFSSFPDRGIYSDFVAEPLTNNLLLMENPQWKIIRNKLTPAFTAGKLKTMYDQIKECGDELMKNIDIDLNRTSNEIEVKDIMGKYSTDVIGTCAFGLKLNAINDDESPFRKYGKLIFKPSLRVLMRELCVMITPALLKVVRLKKFPTAATDFFHAAFNETMTYRLENNIVRNDFVHYLMQARNDLVLNTDLPKHEKFAESQIVANAFVLFAAGFETVSSAISYCLYELALNKSIQDRVRKEIQLQLSKNNGQINHELLIDLNYLDMVIAETLRKYPPLVALFRKASQTYRVPNSSLIIEKGQKIIIPIYAIHYDNKYYSDPEKFIPERFSAEEKAKRPSGVYLPFGDGPRICIGKRFAEMEIKLAFVEILTKFEVFPCEKTEIPLKYSKKVLTLVSKHGIWLRFKRIN

>ApisCYP_XM_001948453

MYPSSSSATDWWIYIVTPCLVAVTITYYFCISTFNKWEKLNVPYIKPIPLFGNFLKVALAKDHPLEFYDKIYYKFSGLKYGGLFQMRTPYLMVRDPEIINNMLIKDFSSFPNRGIYSDLAANPLSDNLFFMENPRWKTIRSKLTPAFTSGKLKIMYDQIKECGDKLMKNIDNDLKGKNDEIEVRDIMGKYSTDVIGTCAFGLKLNSISDDESPFRKYGKSIFIPSLRTLFRELCLMVSPALLKVVRVKDFPTDATAFFNAAFKETITYRLENKIVRNDFVNCLMQARNDLTLNTNLPKHERFSESQIVANAFVMFAAGFETTSTTLSYCLYELALNIHIQDKVRQEIQLKLSKSDGQIDNEFLMGLNYLDMVIAETLRKYPPLIALFRKASQTYRLPDNLILEKGQKIVIPIYSIHFDSKYFEDPLKFNPERFSSEERAKRPNCVYLPFGDGPRTCIGKRFAELEMKLALVEMLTKFEVLPCGKTEVPLKYSNKALTLMPKHGIWLRFKKIV

>ApisCYP_XM_001948520

VKNKSGIVRHDVAQSLIEARKDLVLGSTNENGFTEQDIVANAILMFFAGFEPVSSTLSFCLYQLALNQHNQEEMRDEMNSKVKEHGKINNDFLVDLHYSDMVLAETGRMYGVTNALFRETVKTYQVPGESLVIKKGTRIMIPLNSIHHDSEYYPDPYKFDPQRFSPEKTKRMSDTYLPFGDGPRFCIGKRFAELEMKMVLSQILTTFRVLPGEKTEVPLKFKSGLPLLVSKNGIWLRFQSISK

>ApisCYP_XM_001948546

MFTANWWINVITPCTIIVTIAYYFCVSTFKRWEKLNVPYIKPIPLFGNFLNIALGKDHPLEFYNKIYYEFAGRKYGGLFQMRTPYLMVRDPEIINDVMIKDFSSFPDRGIYSDFTANPLSNNLFFMENPQWKTIRNKLSPAFTSGKLKTMYDQIKKCGDELMKNIDIDLNKNGNEIEVRDILGKYSTDVIGTCAFGLKLNAISDDESPFRKYGKSIFTPSLRMLFRELCLMITPALLKVIRVKDFPTAATDFFHAAFKETMTYRIENKIVRNDFVHCLMQARNDLVLNTDLPKHEKFTETQIVANAFVMFAAGFETVSTTVSYSLYELALDKSIQDRAREEIQLKLSKNDGQINHEFLMDLNYLDMVIAETLRKYPPLVALFRKASQTYRIPNDSLIIEKGQKIIIPIYAIHYDTKYYPEPEKFIPERFSVEEKAKRPSGIYLPFGDGPRMCIGKRFAEMEMKLAFVEILTKFEVFPCEKTEVPLKYSNKVLTLMPKHGIWLRFNRIN

>ApisCYP_XM_001948572

MVQKNFWTKIGGACCIVVACITALVKLVLKYVVGTYSNVEYPSEAQQKIYKTIADIPGPRSLPVFGTRWIYWKFCLYKLNAVHLAYEDMFNRYGDIIREEALWNIPVISVKNRDFIERVLRQSGKYPIRPPNEVTANYRKSRPDRYTNTGLVNEQGEVWAMLRNKLTPELTSPRTIRRFLPEVNQLADDFNNLISLARDGNNVVKEFEAYCNRMGLESTCTLILGRRFGFLDGEISETATRLADSVTSQFRASQEAFYGLPLWKLIPTKAYKDFVASEDALYNIVSEIVDSALIDEQQSCTDVRSVFVSILQTSELDNRDKKAAIIDYIAAGIKTLGNTLVFLLYLVAKHPEVQEKIYNEISRLAPAGTSVTAEHLHKATYLRACITEAHRLKPTAPCIARVLESEIEYDNYRLPPGSVVLLHTGLACLDENNFKDATSYRPERWLDELTKKSPFLVAPFGCGKRMCPGKRFVDLELQIVLAKMVKQFEIDFEGQLKTEFEFLLTPVDSNFILRDRIC

>ApisCYP_XM_001948633

MVQKNFWTKIGSACCIVIACITTLVKLVLKYVVGTYSNHENPSDAQQKIYKTIADIPGPRALPFFGTRWIYWKFCLYKLNAVHLAYEDMFNRYGDIICEEALWNIPVISVKNRDFIERVLRQSGKYPIRPPNEVTANYRKSRPDRYTNTGLVNEQGEVWAMLRNKLTPELTSPRTIRRFLPEVNQLADDFNNLISLARDGNNVVRGFEGYCNRMGLESTCTLILGRRIGFLDGEVSETATRLADSVTSQFRASQEAFYGLPLWKLIPTKAYKDFVASEDALYDIVSEFVESALIDEQQSFTDVRSVFVSILQASELDNRDKKAAIIDYIAAGIKTLGNTLVFILYLVAKHPEVQEKIYNEVSLLAPAGTPITSEHLHKATYLNACIIEAHRLKPTAPCIARVLESEIEYDNYRLPPGTVVLLHTGLACLDENNFKDATSYRPERWLDELAKKSPFLVAPFGCGKRMCPGKRFVDLELQIVLAKMVKQFQIDFEGQLKTEFEFLLTPVDSNFILRDRIY

>ApisCYP_XM_001948658

MALQKIIRKIWTSIKVTCFIVLACVTALVKFVSKNTLGIYRKFRKPADAQRRIYKTVADIPGPRSFPIIGTRWIYWKFGSYKLNAVHLGFEAMFLCFGDIIREETLWNSPVISVINRDCIEKVLRQSGKYPIRPPNEVIANYRRSRPDRYTNTGVSNEQGVIWNSLRKRLTSKMTSPDVVQGVFPEIKSMVDDFIHLLCQARNKNNIVKGFEGLSNRMGLESSCMLILGRRNRFLDRVVNETAMRLTDAVTTQFRASQKTFYGHPFWKIIPTKLYKEFIASEETFYEIMSEIIDFALSDETQSGISENSVFGSILRAPNMDMKEKKAAIIEFIGAGIKTFGNTLVFVLYLIAKHPEVQEKLYNEISRLAPADTPITNEHLKQAKYLNACIMEAHRYSPTAPCIARVLESQIIYDGYCLPKGTTVLMQTGLACLDERNFKDATSYIPERWMNKETYDSLFLVAPFGCGKRICPGKKFVELALKIVLAKMVKQFHIGYEGQLETVFEFILTPVNANFILRDRIN

>ApisCYP_XM_001948680

MEFVFSSLTYLLLFVLTAVLLFLIRDELKTKQVDHRAGLVDPPAPKAWPIIGHLYLMARYKVPYRVFDEIMADLGSVFRLDLGSVPCVVVNGLNNIREVLMIKGDHFDSRPSFRRFNQLFKGDKNNSLAFCDWSQLQKTRRELLRAHTFPNTTSNMYTRLDTCLKTELADLTDTLDTMANTECVDIKNMLLHTCANVFMSYFCSTRFSRSYDKFREFIRNFDDVFYEVNQGAPCDFLPSLMPLYHWHFKKIRSWSSKIRNFMETEIFNKRKAAWVPGTKPVDFVDNLLDAVTQPDRDDGFDMDIGLFSLEDIIGGHSAITNFIVKTLGFLVDRPDVQRRIQEESDAVVRASGSVGLSDRSQMPYTEAVVYESLRLIASPIVPHLANRDTSVDGVRIRKGTTVFLNNYSLHMSPELWNNPEHYSPERFINAEGRLEKPEYFIPFSGGKRSCMGYKLVQLLSFCTISTLLNKYTLLPVEDVSYAVPKGNLALPFVTFPFRLRPRNFRKQ

>ApisCYP_XM_001948854

MQSVSGFRLTTTEVFAYPTICFVVILWCRYKWNRRHLDKLAAGLKGPPAYPIIGSALQFIGTPEEIMDNIIKLIKDYSPEPFKICMGPYFGVAIVKPEDVQIVLNSSRALQKDRFYNFVKNIFGEGLLTAPVDKWRKHRRLITPSFNSILLNEFFPVYNEKSKMLIRNLKSELNKTQPFDLWDYIAPITLNLICQNAMGYNLDSQSKSGSEFEKAMIKASELDSIRVSKPWLYPSIMFSLYLKLKGYSNVFNSLYKLPLKMIHKKREEFAQKKIGNESNYLDVTDNERKHSKVFLDTLFELNEAGANFSYDDIRDEVVTMMIGGSETNAITLCFCVLLLAIYPSIQDKVYDEIYDVLGDGDQTITIEDTSKLLYLDQVLKETLRLFPVIPLILRQLQGDVKIISNNIVLPKGSTCYLSPLATHRDSDSYPNPTSFDPENFSPENIAKRHKYSFIGFSGGPRGCIGSKYAMLSMKVLVATFLRNYSVHTDCKFNDIKLRLDLLLRSSNGYPVTIRTRDRRPVYIKLEYI

>ApisCYP_XM_001948871

MNKYEKSMFKAWLFNKLYIVLTRPEDIEFVLASPKFLRKAKEYMVLQQSIMGQGIFTIEDINKWKINRKLVTKGFSFKLLKEFIPIFYEEALVLAEILGDNSDSTSKECDISVPVSMATMEMIGKTALGVTFNAQKGGCNRFVENLLTAMHAWEYRITHPWYLSSTLFQFSSIKQKHDHSQKIINEFTDEIIKSKIVEINNSGSENGVNADDDDIGRNTKTLTKIFLENPHENMTLEQIRDELVTVMIGGQETTAMANACVVFMLAHHQDVQDKVFKEQESIFSIGDRNRPITYNDLLQMEYLERVIKETLRLFPPLPVFGRDLNEDTTIGDHLCPAGSTLIICPLFLHSSPQHYGSTAHGPDAFDPDNFLPEACHERHAYAYIPFSTGPRNCIGIKYAMLQMKTVASTLVRHHRFLPSDRCPTPDQLRLVFLTTLKLADGCYVKVEPRRPQ

>ApisCYP_XM_001948899

MISWMFNCLIDSFTLICTTVIGLLFYYYSTSTYKKWRKANVPHTKPVPFFGNFFRSTLGFETINDTYHNIYKQFPDKKFCGFYQMRTPTLMIRDPELINNVLIKDFSHFTDHGLDMDPSVNFLASSLFFTRGQKWKIMRQKMSAGFTSGKLKLMHSQIKDCSKEMIDYIDRKSKTTDQFDMHDIMNKYATDVIGTCAFGLKLGSMKDEDNEFRKFTKLLFKPSFRLIFTNILSLISPKTSNILKIKTSSPEVMEYFTTSFQNVIEYREKNNMDRNDVAQTLMRARKELKFTEMDIISNAILMYLAGAEPVSDTLGFCLHELAINKHVQDKLRKHINTKRKEHGGEFTNDYLMDLHYADMVLTETLRKCNGTIVLFRKATKAYQVPDSSLVIEKGQQIIIPTYSIHHDPKYYTNPDVFDPERFSPEEKSKRPSSTELLFGDGPRFCIGKRLAELEMKLGLSEIISKFEILPCEKTENPVQLANAGGAIKPKNGIWLI

>ApisCYP_XM_001948952

MXXXXXXXXXXXXXXXXXILVITIVILILKGRKNRILANRIPGPNGWFLVGMLPLFLQGPEKLIKNILREYRIYEKHIVKFWLFNNLYIVLTRHQDIELVLGNPKFLRKSKDYMVLQESIMGQGIFSIDDIEKWKNNRKMVMKGFNFTPTKSFIPIFYQEANVLAEILQEKCVLKSNECNISGPVSMATMEMIGKTALGVTFNAQTGGCNQFVEHLQTAMHAWEYRVTHPCEKKIESDNEECCQKSKTVLEILLGSSHKMDHEQIRDEIVTVMIGGQETTAMAITCTIFMLAHHQDVQNKVFEELQSIFVNGDRNVPPTYKDFQQMKYVEMVIKETLRLFPPLPFLGRRLDEDMKIGEYMCPAGAALIICPIFVQSSPLYYTDSEKFNPDNFLPDACGSRHSYAYIPFGAGLRNCIGIKYAMLQIKTVISTLVRKIKFSPSERCPTPEDLRLMFLMTLKLVDGCYIKMEPRT

>ApisCYP_XM_001949467

MFIAAATITIFIIFCFLDIITSPKYPSGPTRVPLLGNFLEIQKLKNKLGFYHLVWDKLAKCYGQVYSVKFGPIETVVVSGYDAVREVLSKDDFDGQADGFFFRTRAFYKKLGIVFVDGPMWTEQRKFCMRHLQKLGFCGDVMEKIVIEEVNDLVLDITRKYENGKSIEVRGLFEVSVLNGLWAMLAGGRFSLNDSRLARVVELIHESLRILDMPGGILNQWPFIRYLAPKLSRNKHLKQIINELYILLKESVEEHKCSENDQEDFISAFLMDIEKNKKSLGSFSEEQLVVILLDLFLAGSETTSITLSSVILHLLMNQDIQTKVRAELDAVIGDREILPSDRKRLNYLEAVFMEVQRHSNVVPLAIATNRTIRKTTLQDYIIPKDTLVLASIWSVHMDEQHWGDPEVFRPERFLDSKGKIINDSWLMPFGVGKRRCLGEKLAKTYIFMFIAKLIQHFEIRIPTDIQLPDKPQNGVNISQTPVSVFFIPRRCLKAN

>ApisCYP_XM_001949505

MIGAVSKEFFRLLLEWHSKFGDTYQLWIGLRPFIAMADADHIQGVDTTSVTLSWVMYVLGKHPHVQDRIVEELNEKIPNFGDGKLTVNILSSLDYLGRTIKEVLRLYPSVPFIGRQIYKPLTIGDHTILPGTSIFINVFALHRNEKHFENPEKFDPDRFLEENKKDRHRFAFVPFSAGSRNCIGK

>ApisCYP_XM_001950191

MDSSTLILTCIAVALIAIAIIRRLVFVASFRSIAGPPALPIFGNALQLNGSPSDFFRLLLEWHSKFGDTYQLWIGLRPFIAMADADHIQEILKSSVHIDKNLEYELLLPFIGTGLVTSSGSKWHTRRKLLTPTFHQNILEEFLPLIEKQIKTLVKVLRKEVNNVNGFDIKPYAKLAALDTIGNTAMGCEFNSQENSQLEYVKALDELTAIMQKRFITPWLKPNLLFNLTSLSKRQKACIDVIHTFTRKVVKERKDNFKLFNGQTSDANKKKTHYEKKPNRALLDLLIEVSEDGKVLSDEDIQEEVDTFMFAGVDTTSVTLSWVMYVLGKHPHVQDKIVEELNEKIPNFGDGKLTVNILSSLDYLGRTIKEVLRLYPSVPFIGRQIYKPLTIGDHTILPGTSIFINVFALHRNEKHFENPEKFDPDRFLEENKKDRHRFAFVPFSAGSRNCIGQKFAMIVLKIAVATLIKTYRVKSIDPEEKLGLVGEIVLNALNGIHVTLEERA

>ApisCYP_XM_001950260

MSLLLMAWYFVCFVTVILLLIALRTCRKPKNYPPGPKWIPFVGNTYQLSKLAATKNGQYLAFEELRQRYKSDIIGLKLGREYVVIVFGNDLLNETFHRDEFQGRPDNFFMRLRTMGKRRGITMTDGDLWKVHRSFAVRHLKLLGLGQRRVDELIHDEYQLMVDRLFDATKSVTPTLYLQSAVMNVLWELTAGTKFEDPKLLTLMRKRSSAFDMAGGLLNQIPWLRYLAPTRTGFSLITEINQQLYSLISNIIVEHKKTITHTTRDFIDAYLNQMKKEEIYNTMFTEEQLIAVCLDLFIAGSSTTSSTLDFAILAMARWPDVQAKVQSTLDEIQPPGTYITAEQILKNRYVEAVLLETKRLNHVTPIIGPRRVLRNTNLNGYNIPKNTTILMSLYSVHQDQLKWGDPEVFRPERFMDTNGKINTTEDMYFFGFGKRRCPGEALAQRFVNLAFANLIHDFTIEIDQLPDGVNCGILLTPKPYKIKMTKRK

>ApisCYP_XM_001950505

MICFSCWLEIVPIAAIASALLTYVYCTRYYGHWTALGVPHTKPAPLLGHFAGPTMGRESGTITVDTLYRRFVGHRYFGVYQLRHPMLVVRDPVLVHAVLATEFGSFHDRVMSRTSFEHDGLFNSLVNLRGDKWKAVRAKLSPTFTVAKLKAMFASLHVCTGQLTDKLLLLTSGGQGIVNVTDVSSKFTIDTIGRCAFGINCNTLFDSNTEFQRAGQAVFTPTLKSSVLNFMRLIDLGWLVDLFRLRSMPDLVYEFYLNLFQDTLELRKNEKEDRNDFVSILVKLRNDEKINNSRVGNLQERHFIDVLASNAFIFFAAGFETTASAMSYCLYELALNQDIQVELRKQIQHTLNENGGILTYDVLKDMKYLDMVLNETLRMHPPGPGLLRVCTKKFKIPDSDITLDTGMKVLIPTYSLHHDPAYYPNPELFDPLRFTEDNKALRPNGTFLPFGDGPRICIGLRFALMEAKTGLAEIISKFEIFPCKYTKIPIKLNPRSILLTPNEPISLLFKQIA

>ApisCYP_XM_001950557

MTFRQFKPFSSIPEPKRWPLLGHTHLFIPKIGPYDSQHLTEAMGDIERMLGPVFKLMLGGKTMVVTTRVEEAKTLFAHEGKHPARPIFPALNLLRKKPFGTGGLVSENGVEWYRLRKAIAPLMSKNIYESYIPQHKKAAVDFIDYIKLNRNKDKCLKDMFYHLTKFSVEAISIVSPGLRIKCLNTTMSECFVEAGNKFMDGLYNTLKEPPIWKFYKTNAYRNLESSHSTCKNFIDEYLKQTHEHNALVNAINTNSNLTNTDINLLVLEIFFGGIDATATTLAMTLFYISQDESVQKACEEDVLQGTNAYIKACIKETLRLSPTAGANARYLPKTTVIGGYEIPANTLVMAFNSLTSTKEKYFKAPLEYQPSRWLRNSNIQKFDPYASLPFGHGPRMCPGRHVAMQEMTILLSELIKNFKISLPAEHAKNIGMIYRMNRIPDSRIDIIFNNK

>ApisCYP_XM_001950999

MTSTELTAYGVISFIVVLWCHYKWNRRHFERLASKMTGPPAYPIIGAGLEFVGTPQQVIERIIKLFDIYGSEPFKVWMGTSLGVTISKPEDVQIVLNSSKALEKDQFYKFFKNTVGEGLFSAPVHKWRRHRRLITPVFNANLLDQFFPVFNEKNRILTRNLKKELGKTQPFDLWDYIADTTLDIICQTAMGYNLDTQLNNESEFAEALTKASELDSMRIYKPWLHPDIIFSIYGKLTGLHNVYKTLHKLPNQVIKEMKETYAQRKIDNKSNTIDVNDDDKKRLKVFLDTLLDLNEAGANFSDEELRDEVVTMMIGGSETSAITLCFCLLLLAIHPEIQDKVYDEIYEVLGDGDQTITIEDTTKLVYLEQCLRETLRLYPIGPLLLRQLQDDVKIFSGDHTLPKGTTCIISPICTHHIPELYPNPWSFNPDNFDAENVSKRHKFSFIAFSGGPRGCIGSKYAMLSMKVLVSTFLRNYSVHTNVKLSDIKLKLDLLMRSANGYPVTIRPRDRRPTYKKNTHCSTVNL

>ApisCYP_XM_001951058

MWILVLVLFSVVVALLSYLDMRKPKNYPPGPKWLPILGSALTVNSLRKQTGYLYRATICLAESYGPIVGLKVGKDRQVVCCGYNAIKEMLTKEEFDGRPQGPFYETRTWGTRRGLLLTDEEFWVEQRRFVLRHLREFGFGKRTMAELVQDEAVQLVEDFKEKIAMSKNGNGEIFEMRDAFSVGVLNTLWSMMASKRYNADDIELKNLQALLTELFANIDMVGALFSQFPVLRFIAPEASGYKSFVNIHQQVWKFLKAELDDHKETFIINQPRDLMDVYLQMLHSEDKKESYSESQLLAICMDMFMAGSETTSKSLGFGFLYLLLNPEVQKKAQEEIDRVVGRDRLPTLNDRPNMPYLEALVLESVRVFMGRTFSIPHRALKDTTLQGYHIPKDTMVIANFAALLNDDDVWDHPDRFWPERFIGCDGKLIVPDEYLPFGYGKHRCMGQTLARSNIFLFSACLLQNFDFSVPDGQAPPSTLGVDGVTPSPGEFNAYVSLRPR

>ApisCYP_XM_001951431

MLIFANFWIDFIILITVLFSIIYYYCTSTFNVWKKLNVPYIRPIPLFGNYLRVALGIENPMETYRKIYCELAGFKYGGMFQMRTPYLMIRDPEIINNILIKDFSYFTDRGIYVDFKTEPLSEVLFLMNNPRWKKFRSKLSPAFSSGKLKQMFNQIEKCGHDMINNIFAELKKNPNDIDMRDVVSKYSMDVIGSCAFGLTLNVASDDTSLFRKYGKTAFGPSILYFIREICVMISPAILKILRLTFFPSKTTAFFGSVFKETKTYREQNNVLRNDIVHALIQAHQSDENSSKDETLMESQILSNAFGFFAAGFDTTSTSISYCLYELALKKNIQDRVREEIKLTKSKYNGVIDNEFLNDLHYLDMVIAESLRKYPLMFALFRVATKTYRVPNDSLIIEKGQKIIIPTFSLHYDPKYFSDPEVFNPERFSPKEKAMRPNGVYLPFGDGPRHCIGKRFAEMEMKLALVEILSKFEVEPSEKTMIPVQFSKLSVVVIPRDEKILLKLNPLSE

>ApisCYP_XM_001951556

MIEIIVYIIVVIFVVMWCYFKWHNRPFEKLAARMPGPPAYPFIGTLYGCIGLTSGQIVSRILDYVKDYNLEPFKFWMGPYFGVFIVKPEDLQIVLNSSNAFQKGFVYDFFKVILGEGLFTAPVDKWRIHRRMISPFFNGKLLEQFFPVFIEKNRILIRNVGKQLNETQVFNLWDYVAPFALDVICENTMGYNLDTQTNKNECEFAKAIVK

>ApisCYP_XM_001951794

MIEQIAYIIGIVLVWSYFKWQNRRFEKLAAIMPGPTAYPIIGIGYKFFGSSEDVMSKIIDLVKEYNLSPIKLWLGPYFAVSISKPEDLQIILNNSKALQKDQMYDFFKYAVGEGLFTAPVDKWKRHRRMITPAFNAKLFEQFFPVFNEKNKILIKNVTKELNKTQMFDLWHYVAPAALDTICQTTMGYNLDTQSNNKECEFGEAIVMASEVAALRIYKPWLYPEMVFSMYLKLTGHQRVFETVKKFPLQVIKEKKDEFDQRKKAINAKVDLANNKDENQSKLFLDILFELNNTGGNFSDSDIRDEVVTMMTGGSETSAITICFCLLMLAIHQDIQDKVYDEIYDIFGGSEETITIEDTTKLVYLEQVLKETLRLYPVRPVLLRELQDDVKIFSNDYVLPKGTTCVLCPITTHHCPVIYPNPWSFNPENFTPENVAKRHRYSFIPFSGGPRGCIGSKYAMLSMKVTVSTFLRHFSVHTDIKLTDIKLKIDLLMRSVHGYPVTIRPRVKLPTKY

>ApisCYP_XM_001951870

MNINTKLLIYKSLLRPLWTYGIQLWGAAKPSNTHQFTDDDIIGNAILLFAAGAETISSTVSFCLYELALNKEIQDKMRAEICSMKAKHDGQFNNDFLMDLRYTNMVLEETGRKYSIASILMREATKTYTLPDESFVIEKGQKLIIPMFSIHRDPKYYPDPLIFDPERFSKEQKSQRPNGIYMPFGDGPRMCMGKRFAELEMKLVLSNVLSKFEVLPCEETEIPLEITDETGVIAPKRDLVLKFRPIIED

>ApisCYP_XM_001951948

MSGVWSIPFVQLCAAAVLLVTFLGYMYLTYHYGKWTGLGVPHAAPSPPFGSLRDVVMGRVPLVDAIHSLYRRFDGQRYFGIYEGRQPLLVVCDPQLVHTIMVKDFRSFVDRNAGKVSFVHDKLFDHLVNLRGEQWKAIRAKLSPTFSAAKLKSMLGDINVCTARLIDNLNGQITKNSGIVDVSEASAQFTTDTIGSCAFGLDCNALSNPDSEFRRTGRAIFTPSLRSNLLNITRLVGFGRLLDVFRIRGMSGNIYDFFDNLLDTTMEQHKSGENTRNDFIALLVKLKDEEKQKEHGQKLFTDDILAANSFVFFVAGFETTASTISYCLYELAMNPEIQVKLRENIKKTLDANDGKLAYDTLKDMKYLDMVINETFRLHPPVPVLNRVCTQKYTITDSNITLNVGDKLIIPTYSLHHDSKYYSDPEIFDPERFTEENISSRPHGTFLPFGDGPRICIGLRFAMMEAKTGLAEILSKFEVSPCKETQTPIKIKPRSILLTPNESIRLSFKSIDQ

>ApisCYP_XM_001952075

MDSSTLILICIAVALIAIAIVRRLEFVASFRSIAGPPSLPIFGNALQLNGSPSDFFRLLLEWHSKFGDTYQLWIGLRPFIAMANADHIQQILKSTVHIDKNLEYNLLLPFIGTGLVTSSGSKWHTRRKLLSPTFHQNILEGFLPLIEKQMKTLVKVLRKEVNNVNGFDIKPYAKLAALDTIGNTAMGCEINSQENSQLDYVKALDELTAIMQKRFITPWLKPNLLFNLTSLSKRQKACIDVIHTFTRKVIKERKDNFKLFNNQTSDANKNEIHYEKKPNRALLDLLIEVSEDGKVLSDEDIQEEVDTFMFAGVDTTSVTLSWVMYVLGKHPHVQDKIVEELNQKIPNFGDGNLTLNILSSLDYLGRTIKEVLRLYPSVPFIGRQIYQPLTIGDHTILPGTSIFINVFALHRNEKHFENPEKFDPDRFLKEKKNDRHRFAFVPFSAGSRNCIGQKFAMIVLKIAVATVIKTYRVKSIDPEEKLGLVGEIVLNALNGIHVTLEERT

>ApisCYP_XM_001952149

MDKHPSPKDKVYDEIYDVLGDGDQTITIEDTSKLLYLDQVLKETLRLFPVIPLILRKLQGDVKIISNNIVLPKGSTCYLSPLATHRDSDSYPNPTSFDPENFSPENIAKRHKYSFIGFSGGPRGCIGSKYAMLSMKVLVATFLRNYSVHTDCKFNDIKLKLDLLLRSSNGYPVTIRTRDRRPVYKFKLEYI

>ApisCYP_XM_001952404

MFHEHAPERNKESILSSLKNCMDPSGGSLLEISSGSGQHISYFAAHFPNIEFQPTEINRRLFETINACTHNLSNVLPAKYLDVSSDPSVWLGGQLMNTQYDYILNINTLHVSNFKCTEGLFRGSCCALKPKETGAIIMQSVGEFRLAVSEVLLYSAIISVVVFWCSCKWNNRHINKLDSKMKGPPAYPIIGSALELLGTPEQVINVLLGFYNNYGSEPFKVWLGPFFGVYIIKPEDVQIVLNNSKALQKDRFYEFIKNIFGEGLLTAPVDKWRKHRRLITPLFNANLLSQFFPVFNEKNKILIRNLKKELGKTQPFDLWDYIAPTTLNLICQNAMGYNLDSHSQCGSEFEKAMIKASELDSIRIYKPWLFPNIFFSLFLRLQGQSNVFKTLKKLPLKMINEKKEVFAQKKIVKETIVMNNTDGEKKNLKVFLDTLFELNETGANFSDNDILDEVVTMMIGGSETSAITLCFSLLLLAIHPDIQNKVYDEIYDVLGDGDQTITTEDTIKLVYLEQVLKETLRLFPVLPLVIRKLQDDVKIISGNHLLPKGTTCYIAPLFTHRDCDSYPNPLNFNPENFSQENISKRHKYSFIAFSGGPRGCIGSKYAMLSMKVMMSMFLRNYSVHTNCKFNDIKLKLDLLLRSANGYPVFIQSRDRRPSYKLNKT

>ApisCYP_XM_001952415

MISCLIYVLFGTPAIAAVAVLAAILYYYTTNTYDKWLKLKVPHDPPWPLVGNTAKMMTLIEHQLTTIDGIYKRFSGEKYCGFYQMKTPFLMIRDPELINNILIKDFSNFADRGFHKDPALNIIANGLFFMEGPKWKMMRQKLSPGFTSGKLKLAHNQIAECSDELMRFIAAKMKENDQIEVKETMSKYSTDVIGTCAFGLKLDTVKNEGSDFRLYGRKILKLSFRFLLAEMVSPKILKLLGVAEFPPDASAFYESAFKEVIRYREENGIVRHDVAQSLIEARKELVLDSTDENGFTEQHIIANAILMFLAGFETVSSTLSFCLYHLALNQDVQEKIRDEMNSKLKQHGKINNDFLVNLHYTDMVLAETERMYVVTNALFREAVKTYHVPGDTLVIEKGTKIMIPIYSIHHDPTYYPEPYIFDPQRFSPEEKAKRQSSTYLPFGDGPRFCIGKRFAELEMKMVLSQIITTFRILPCEKTEVPLKLQNGLPMMVAKNGIWLRFQSISE

>ApisCYP_XM_001952585

MFFVAVVISVFIIVCILDIITPHKYPIGPTRVPLLGNYLEIRKLRNKLGFYHLVWDHLAKYYGKVFSVKLGRIEAVVVSGYDAVRQVLCKDDFDGRPDGFFFRFRAFYKRLGIVFVDGPTWTEQRKFCMQHLRKMGFGGDLMERIIIEEVNDLMLDISRKCENGKPIEVYGLFDVSVLNGLWAMLAGHRFALNDSRLARLMELVHVSFRMLDMSGGILNQMPFIRFFAPKCSGYKYLKQIINEFYTFLKESVEEHKCRANDQEDDFISAFLKEIEKNKESPGSFSEEQLLVILLDLFLAGSETTSSMLSFVILLLLKHQDIQAKVHAELDAVVGDREIHLADKNRLNYLEAVLMEVQRHSNVAPLAIAHRTIRKTSLQEYTIPKDTLVLASIWSVHMDEQHWGDPKVFRPERFLDSSGKIINDSWFMPFGVGRRRCLGEILAKTNIFMFIAKLIQHFEIRIPQGAQLPDKPQDGVTISPSPFSAIFIPRRCLSQ

>ApisCYP_XM_001952686

MPYLEALVLESVRVFMGRTFSIPHRALKDTTLQGYHIPKDTMVIANFAALLNDDDVWDHPDRFWPERFIGCDGKLIVPDEYLPFGYGKHRCMGQTLARSNIFLFSACLLQNFDFSVPDGQAPPSTLGVDGVTPSPGEFNAYVSLRPR

>ApisCYP_XM_001952692

MTMFTANWWINFITPCTIIVTIAYYFCVSTFKKWEQLNVPYIKPIPLFGNLLNVALGKDHPLDFYNKIYHKFAAHKYAGVFQMRTPYLMVRDPEIINDMLIKDFSSFPDRGIYSDFVAEPFSNHLFFMENPQWKIIRNKLTPAFTSGKLKMMYDQIKECSDELMKTIDIELIKNDDEIEVRDIIGKYSTDVIGTCAFGLKLNAIKDDESPFRKHGKTLFEPSLRALFKELCLMIAPALLKVIKVKDFPTDATDFLHTVFKETITYRQKNKIVRNDIFQCLIQVRNDLVLNADLSKNEKFTETQIVANAFAMFAAGFETVSSAISYCLYELALNKSIQDRVREDIELKLSNNDGQINHELLIDLNYLDMVIAETLRKYPPVVALFRKASQTYRVPNDSLIIEKGQKIIIPIYALHYDSKYYTDPEKFIPERFSAEEKAKRPSGIHLPFGDGPRICIGKRFAEMEMKLAFVEILTKFEVFPCEKTEIPLKYSNKVFTLMPKHGIWLRFKRIN

**校对报告**

当前使用的样式是 [BMC Genomics]

当前文档包含的题录共18条

有0条题录存在必填字段内容缺失的问题

所有题录的数据正常
